# Common miRNAs, Genes, and Regulatory Pathways in Alzheimer's Disease and Type 2 Diabetes Mellitus: An Integrative Analysis of Systematic Reviews, Bioinformatics and Data Mining

**DOI:** 10.1111/jnc.70196

**Published:** 2025-08-18

**Authors:** Lívia Cristina Ribeiro Teixeira, Jessica Diniz Pereira, Izabela Mamede, Paulo Caramelli, Vítor Corrêa Silva, Adriano Alonso Veloso, Marcelo Rizzatti Luizon, Karina Braga Gomes

**Affiliations:** ^1^ Faculdade de Farmácia Universidade Federal de Minas Gerais Belo Horizonte Minas Gerais Brazil; ^2^ Instituto de Ciências Biológicas Universidade Federal de Minas Gerais Belo Horizonte Minas Gerais Brazil; ^3^ Faculdade de Medicina Universidade Federal de Minas Gerais Belo Horizonte Minas Gerais Brazil; ^4^ Instituto de Ciências Exatas Universidade Federal de Minas Gerais Belo Horizonte Minas Gerais Brazil

**Keywords:** Alzheimer's disease, artificial intelligence, microRNAs, systematic review, type 2 diabetes mellitus

## Abstract

Alzheimer's disease (AD) and type 2 diabetes mellitus (T2DM) are frequent conditions affecting older adults, with evidence suggesting a higher predisposition for AD in diabetic patients. MicroRNAs (miRNAs) are proposed as regulators of gene expression in the mutual pathways among these diseases. This study aimed to investigate circulating miRNAs found to be expressed both in AD and T2DM, as well as their target genes and associated molecular pathways, using systematic reviews (SRs), bioinformatics analyses, and data mining. Two independent SRs were conducted to identify differentially expressed miRNAs in AD and T2DM compared to their respective controls. Searches covered major databases (EMBASE, PubMed, Cochrane, Scopus, Cinahl, Web of Science), gray literature, and reference lists, following the Joanna Briggs Institute (JBI) and PRISMA guidelines. Results were combined to identify miRNAs shared by both AD and T2DM, with target genes extracted from miRTarBase. Pathway enrichment analysis was performed using EnrichR, and relevant pathways were ranked based on gene involvement frequency with artificial intelligence tools. From the SRs (AD: 49 studies; T2DM: 104 studies), 21 miRNAs were identified as commonly expressed (10 upregulated, and 11 downregulated). 337 and 233 genes are potential targets for these down‐ and upregulated miRNAs, respectively. The key pathways identified from those genes were the TCR‐RAS signaling cascade for downregulated miRNAs and the extracellular matrix pathway for upregulated miRNAs. Our findings highlight shared biological pathways between AD and T2DM and provide insights into their shared pathophysiology and potential therapeutic targets.

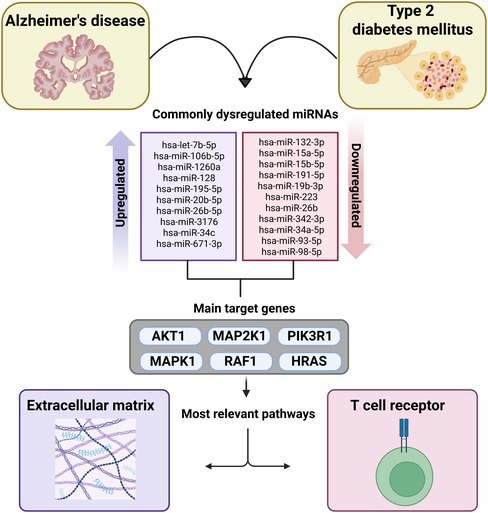

## Graphical abstract

Alzheimer's disease (AD) and type 2 diabetes mellitus (T2DM) are frequent conditions affecting older adults, with evidence suggesting a higher predisposition for AD in diabetic patients. MicroRNAs (miRNAs) are proposed as regulators of gene expression in the mutual pathways among these diseases. In this study, the T‐cell receptor–RAS signaling (TCR‐RAS) signaling cascade for downregulated miRNAs and the extracellular matrix pathway for upregulated miRNAs were pathways shared by both diseases. Our findings highlight biological pathways between AD and T2DM and provide insights into their pathophysiology and for potential therapeutic targets.

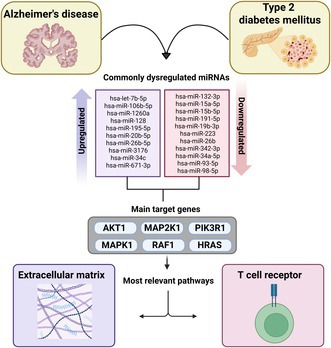

**Abbreviations**
AD: Alzheimer's disease
ADA: American Diabetes Association
ADAS‐Cog: Alzheimer's disease assessment scale‐cognitive
AGEs: advanced glycation end products
AKT1: AKT serine/threonine kinase 1
APP: amyloid precursor protein
Aβ: amyloid‐beta
BACE1: beta‐site APP cleaving enzyme 1
BBB: blood–brain barrier
BM: basement membrane
CNS: central nervous system
CSF: cerebrospinal fluid
DGIdb: Drug–Gene Interaction Database
DSM‐IV: Diagnostic and Statistical Manual of Mental Disorders—Fourth Edition
DSM‐V: Diagnostic and Statistical Manual of Mental Disorders—Fifth Edition
ECM: extracellular matrix
ERK: extracellular signal‐regulated kinase
GO: Gene Ontology
GSK3β: glycogen synthase kinase 3 beta
GWAS: genome‐wide association study
HP: heparin
HRAS: Ras proto‐oncogene, GTPase
IDF: International Diabetes Federation
iNKT: invariant natural killer T cell
IPCs: insulin‐producing cells
IWG: International Working Group
JBI: Joanna Briggs Institute
Kegg: Kyoto Encyclopedia of Genes and Genomes
KSPGs: keratan sulfate proteoglycans
MAIT: mucosal‐associated invariant T cell
MAP2K1: mitogen‐activated protein kinase kinase 1
MAPK1: mitogen‐activated protein kinase 1
MeSH: Medical Subject Headings
miRNA: microRNA
miRTarBase: microRNA Target Database
MMSE: Mini Mental State Examination
MTI: miRNA–Target Interaction
NF‐κB: nuclear factor kappa B
NI: not informed
NIA: National Institute on Aging
NIA‐AA: National Institute on Aging—Alzheimer's Association
NIA‐R: National Institute on Aging—Revised
NINCDS‐ADRDA: National Institute of Neurological and Communicative Disorders and Stroke—Alzheimer's Disease and Related Disorders Association
NINDS‐AA: National Institute of Neurological Disorders and Stroke—Alzheimer's Association
p38: p38 mitogen‐activated protein kinase
PI3K: phosphoinositide 3‐kinase
PIK3R1: phosphoinositide‐3‐kinase regulatory subunit 1
PNNs: perineuronal nets
PRISMA: Preferred Reporting Items for Systematic Reviews and Meta‐Analyses
PROSPERO: Prospective Register of Systematic Reviews
qPCR: quantitative polymerase chain reaction
RAF1: Raf‐1 proto‐oncogene, serine/threonine kinase
RAGE: receptor for advanced glycation end products
SR: systematic review
STAT3: signal transducer and activator of transcription 3
T2DM: type 2 diabetes mellitus
TCR: T‐cell receptor
TCR‐RAS: T‐cell receptor–RAS signaling pathway
Th17: T helper 17 cell
Tregs: regulatory T cells
WHO: World Health Organization

## 1 Introduction

Alzheimer's disease (AD) is the most common type of dementia, accounting for 60%–70% of the 55 million global cases (WHO 2023). Several pathophysiological processes have been associated with the development and progression of AD, mainly the accumulation of amyloid‐beta (Aβ) plaques and tau protein hyperphosphorylation, resulting in tau aggregation and neurofibrillary tangles (NFTs). Oxidative stress, mitochondrial dysfunction, and neuroinflammation also contribute to AD development (Sehar et al. 2022). Neurodegeneration, including synaptic dysfunction and neuronal loss, occurs as a consequence of these pathological processes and contributes to the clinical manifestations of AD, which include progressive memory decline, deterioration of other cognitive functions, behavioral changes, and functional impairment (Guo et al. 2020; Yang 2019).

Type 2 diabetes mellitus (T2DM) is a prevalent chronic metabolic disorder characterized by persistent hyperglycemia due to impaired insulin secretion, insulin resistance, or a combination of both (Galicia‐Garcia et al. 2020). T2DM results from the progressive dysfunction of pancreatic β‐cells, alongside decreased sensitivity of peripheral tissues to insulin (Artasensi et al. 2020; Chatterjee et al. 2017; American Diabetes Association Professional Practice 2024). The development of T2DM is influenced by a combination of genetic predisposition and environmental factors, including obesity, physical inactivity, and high consumption of sugars and fats (Artasensi et al. 2020; Chatterjee et al. 2017). According to the International Diabetes Federation (IDF), 589 million people aged 20 to 79 were living with diabetes in 2024, with over 90% of these cases attributed to T2DM (IDF 2025). This condition can trigger complications that contribute to its significant morbidity and mortality burden worldwide (Zheng et al. 2018).

The increasing incidence of T2DM in the 21st century, along with the evidence of the higher risk of developing AD in diabetic patients, has stimulated research into pathways linking glycemic disorders and neurodegeneration (Rorbach‐Dolata and Piwowar 2019). T2DM has been increasingly recognized as a significant risk factor for the development of AD (Livingston et al. 2024), with epidemiological studies showing that diabetic patients have over a 50% increased risk of developing AD compared to non‐diabetics (Carvalho and Moreira 2023). Experimental studies have shown that high‐fat diets, which induce metabolic disturbances such as insulin response, can also lead to brain insulin resistance, mitochondrial dysfunction, and cognitive impairment in animal models (Machado et al. 2024; Kothari et al. 2017).

Several molecular mechanisms underpin the connection between AD and T2DM, supporting the concept that AD may represent a brain‐specific form of insulin resistance (Chen et al. 2019). Both conditions share pathophysiological features, including impaired insulin signaling, glucose metabolism dysregulation, and chronic inflammation (Chen et al. 2019). In particular, insulin resistance in the brain leads to reduced activation of the PI3K‐AKT pathway and overactivation of GSK3β, contributing to tau hyperphosphorylation and Aβ accumulation—two hallmark features of AD (Chen et al. 2019). These molecular disruptions are further compounded by oxidative stress and AGE‐RAGE signaling, common in both diseases, exacerbating neuroinflammation and synaptic dysfunction (Chen et al. 2019).

Given the shared pathophysiological features and the complex interplay between AD and T2DM, identifying molecular mediators that contribute to both conditions has become a crucial area of research. Among these mediators, microRNAs (miRNAs) have emerged as key regulators of gene expression, offering valuable insights into the mechanisms underlying the pathogenesis of these diseases. miRNAs are small endogenous non‐coding RNAs, approximately 23 nucleotides in length, that play a crucial role in gene regulation as post‐transcriptional silencers, inhibiting the translation of target messenger RNAs (Bartel 2009; Pasquinelli 2012). miRNA‐mediated control of gene expression influences several cellular functions, such as proliferation, differentiation, and apoptosis, and is involved in both physiological and pathological processes (Reid et al. 2011). The involvement of miRNAs in physiological processes is essential for development, as they attenuate the formation of aberrant transcripts and help to suppress random fluctuations in transcript copy numbers (Ebert and Sharp 2012). MiRNAs are also associated with various pathological conditions, such as cancer, inflammatory, and cardiovascular diseases (Smolarz et al. 2022; Colpaert and Calore 2019).

Investigation of differentially expressed miRNAs in diseases is used to elucidate their molecular mechanisms, as well as to identify associated genes and metabolic pathways, thereby indicating potential biomarkers for diagnosis or treatment targets (Backes et al. 2016). Several miRNAs have been described as differentially expressed in AD (Miya Shaik et al. 2018; Wang, Qin, and Tang 2019) and T2DM (Yaribeygi et al. 2018; Dehwah et al. 2012). However, few studies have focused on identifying miRNAs common to both diseases (Alamro et al. 2022). In this context, these miRNAs may serve as potential biomarkers for screening T2DM patients who are at risk of developing AD.

Therefore, the present study aims to investigate miRNAs as a molecular link between T2DM and AD, focusing on the identification of miRNAs commonly expressed in both diseases, as well as the genes and molecular pathways regulated by these, using systematic reviews, bioinformatic analyses, and data mining. Finally, potential drugs targeting genes involved in these molecular pathways are suggested.

## 2 Material and Methods

### 2.1 Systematic Reviews

Two independent SRs were planned and conducted in accordance with the Joanna Briggs Institute (JBI) Manual for Evidence Synthesis (Aromataris and Munn 2020). The research protocol was registered in the International Prospective Register of Systematic Reviews (PROSPERO) (https://www.crd.york.ac.uk/prospero/), under the number CRD42021268596. We selected observational case–control studies that reported miRNA expression levels in patients with AD compared to cognitively healthy controls for the first review, and studies comparing miRNA expression in patients with T2DM and individuals without T2DM for the second review. The results were reported according to the Preferred Reporting Items for Systematic Reviews and Meta‐Analyses (PRISMA) guidelines (Page et al. 2021).

#### 2.1.1 Research Sources and Search Strategy

The search strategy aimed to retrieve the largest possible number of articles on the topic, without restriction regarding the publication date and language. The searches were carried out in the electronic databases EMBASE, Medical Literature Analysis and Retrieve System Online (Medline via PubMed), Cochrane Central Register of Controlled Trials (CENTRAL), Scopus, CINAHL, and Web of Science. The research question for the first SR was: ‘What are the differentially expressed miRNAs in patients diagnosed with AD when compared to cognitively healthy individuals?’ The search strategy was defined using the PECO acronym—Population (P): adults or older individuals; Exposure (E): Alzheimer's disease; Comparison (C): cognitively healthy individuals (without AD); and Outcome (O): differentially expressed miRNAs. The research question for the second SR was: ‘What are the differentially expressed miRNAs in patients diagnosed with T2DM when compared to individuals without T2DM?’ The search strategy, in turn, was defined as follows: (P): adults or older individuals; (E): type 2 diabetes mellitus; (C): healthy individuals (without T2DM); and (O): differentially expressed miRNAs.

The searches related to the AD and T2DM reviews were completed on May 1, 2024. The searches were carried out using Medical Subject Headings (MeSH) terms such as “Adult,” “Aged,” “Diabetes Mellitus, Type 2,” “Alzheimer Disease,” and “miRNAs,” combined with Boolean operators “OR” and “AND.” Gray literature searches were performed in OpenGrey (www.opengrey.eu/); Open Access Theses and Dissertations (https://oatd.org/); Turning Research into Practice (www.tripdatabase.com); Theses and Dissertations Catalog (CAPES) and in the databases of Federal University of Minas Gerais and São Paulo University/Brazil. Other resources included manual searches in the reference lists of four systematic review studies already published on the topic of each systematic review: AD (Wu et al. 2016; Takousis et al. 2019; Swarbrick et al. 2019; Hu et al. 2016) and T2DM (Gonzalez‐Sanchez et al. 2021; He et al. 2017; Liang, Li, et al. 2020; Zhu and Leung 2015). The complete search strategies are available in Supporting Information S1.

#### 2.1.2 Criteria for Study Evaluation

The studies were included according to inclusion and exclusion criteria, which are summarized in Supporting Information S2 and S3.

##### 2.1.2.1 Inclusion Criteria

Observational studies that compared the expression profiles of circulating miRNA in patients with AD or T2DM and their respective controls (case–control studies) were included. Given that miRNA expression is cell and tissue‐dependent, only studies that analyzed circulating miRNAs in serum or plasma were included, as these are analytes obtained in a less invasive way compared to other tissues. Moreover, studies focused on miRNAs from extracellular vesicles, exosomes, peripheral blood cells, and whole blood were excluded to ensure standardization.

##### 2.1.2.2 Exclusion Criteria

Studies that did not meet the eligibility criteria were excluded, including such as animal and in vitro studies, systematic reviews, meta‐analyses, editorials, and interventional studies. Studies analyzing miRNAs in extracellular vesicles, exosomes, peripheral blood cells, whole blood, or other tissues were excluded. In addition, studies were excluded if they failed to describe the diagnostic criteria for AD or T2DM, if they did not report the age of the patients, or if they lacked a control group.

#### 2.1.3 Study Selection

After retrieving studies from electronic databases, duplicates were removed using EndNote reference manager software. Two reviewers (LCRT and JDP) screened titles and abstracts, with disagreements resolved by a third reviewer (KBG). Articles were selected based on predefined inclusion and exclusion criteria. The selected articles were then assessed in full text based on the established eligibility criteria.

#### 2.1.4 Data Extraction

The extracted data included the diagnostic criteria for AD or T2DM; sample size; country of origin of the patients; age and sex of the patients with statistical analysis; year of publication; type of sample used; method of miRNA quantification; miRNAs measured and differentially expressed; miRNA expression in relation to the control group (downregulated or upregulated); and fold change values and/or *p*‐values.

#### 2.1.5 Quality Analysis – Risk of Bias

The assessment of the methodological quality of the included studies followed the criteria of the Joanna Briggs Institute's Critical Appraisal Checklist for Case Control Studies. To analyze the quality of the studies in the review, 10 questions regarding the design and analysis of the results of each study were answered and scored. The questions formulated by the JBI and the scoring criteria can be found on the following website:

https://jbi.global/sites/default/files/2020‐08/Checklist_for_Case_Control_Studies.pdf

Low‐quality studies or those studies with a high risk of bias received a score of ≤ 4. Medium‐quality studies or those studies with a moderate risk of bias received scores between five and seven. High‐quality studies or those studies with a low risk of bias received scores of ≥ 8 (Moola et al. 2020; Munn et al. 2020; Rawal et al. 2022). The scoring system was applied according to the criteria presented in Supporting Information S4. The process of risk of bias assessment was conducted independently by two reviewers. Any discrepancies were resolved through a third reviewer.

### 2.2 Identification of Commonly Expressed miRNAs in Alzheimer's Disease and Type 2 Diabetes Mellitus

Based on the systematic reviews conducted separately for AD and T2DM, two tables were compiled listing the differentially expressed miRNAs identified in each condition, in comparison to their respective control groups. These tables included information such as the full name of the miRNA, direction of regulation (upregulated or downregulated), *p*‐values, fold change, and the reference to the original study.

To identify miRNAs commonly expressed in both diseases, a cross‐referencing of the data was performed. Only miRNAs with identical names in both tables and consistent directions of regulation were selected. miRNAs showing discrepancies in their regulation patterns, either across studies of the same disease or between the two diseases, were excluded from the analysis to ensure greater robustness and reliability of the findings.

### 2.3 Pathway Enrichment Analysis

The target genes associated with each miRNA identified from the systematic reviews were extracted using the miRTarBase (https://mirtarbase.cuhk.edu.cn/~miRTarBase/miRTarBase_2022/php/index.php), a database with experimentally validated miRNA‐target gene interactions (Chou et al. 2018). The molecular pathways regulated by these target genes were then analyzed using EnrichR, a functional enrichment tool that links selected genes to metabolic pathways, drawing from databases including Reactome (Fabregat et al. 2017), Kyoto Encyclopedia of Genes and Genomes (Kegg) (Kanehisa and Goto 2000), and Gene Ontology (GO) (The Gene Ontology 2019). In these analyses, we considered only target genes of miRNAs that have been validated by experimental methods providing strong evidence according to miRTarBase, such as reporter gene assays, Western blot, and quantitative polymerase chain reaction (qPCR) (Chou et al. 2018). The relevant molecular pathways related to the pathophysiology of T2DM and AD were explored, while pathways related to other conditions were manually excluded, such as infectious and rheumatic diseases, cancer, and those associated with cell cycle regulation.

### 2.4 Selection of the Most Relevant Biological Pathways

#### 2.4.1 Data Processing

The data processing was performed in three steps: (1) Creation of two variables—ID (pathway name) and GeneID (associated genes); (2) The GeneID variable was converted into a list where each gene represents an item in a pathway; (3) The processed data was stored in a DataFrame for further analysis.

#### 2.4.2 Gene Combination Analysis Using Apriori

The Apriori algorithm was employed to identify frequent gene combinations. First, we conducted the identification of Individual Genes: the frequency of each gene was counted. After, new gene combinations were formed from frequent sets, discarding those with infrequent sets. Support count was conducted—support indicates the frequency with which a gene appears in pathways. The support of gene X in relation to a set of pathways P is defined as the proportion of pathways in P that contain gene X. It can be expressed by the following formula:

$$\text{Support}\;\left(\text{gene}\;x\right)=\frac{\text{Total number of pathways containing gene}\;X}{\text{Total number of pathways}}$$

A minimum support threshold of 0.15 for upregulated miRNAs and 0.4 for downregulated miRNAs was applied, removing genes below these thresholds. These values were defined based on exploratory analysis: lower thresholds generated redundant gene combinations (e.g., larger sets that were simply overlaps of smaller, already identified groups), which added little interpretative value and could clutter the results. For iteration, steps 2 and 3 were repeated until no new frequent gene sets were generated. From the frequent gene sets, association rules (“If X, then Y”) were created with a minimum support threshold of 0.01 to maximize information.

#### 2.4.3 Pathway Ranking

The ranking of pathways was performed using the average support of the genes within the pathway. The support of each gene was calculated individually, and the average support of all genes was used as the score for the pathway. Pathways with a high score have a higher quantity of important genes compared to pathways with lower scores.

$$\text{Pathway Score}=\frac{\mathrm{Sum}\;\text{of supports of}\;\mathrm{all}\;\text{genes in the pathway}}{\text{Number of genes in the pathway}}$$

It is important to note that the pathway score does not represent the support of the pathway. The support of the pathway is represented by the frequency with which the set of genes representing the pathway occurs in the dataset (Saaty 1987).

### 2.5 Identification of Drug‐Gene Interactions for Therapeutic Drug Suggestions

To identify potential pharmacological interventions, a drug‐gene interaction analysis was performed using the Drug‐Gene Interaction Database (DGIdb, https://dgidb.org/). DGIdb compiles information on known drug‐gene interactions, making it a valuable tool for identifying compounds that may inhibit or modulate disease‐associated genes (Cannon et al. 2024).

The most frequently occurring genes, previously identified through data mining, were individually queried in DGIdb. For each gene, only U.S. Food and Drug Administration (FDA)‐approved drugs were selected, and an interaction score threshold of > 0.10 was applied. The interaction score is a ranking metric calculated by DGIdb to prioritize drug‐gene interactions based on supporting evidence. This score considers the number of known drug and gene partners, the strength of their associations, and the number of supporting publications and databases (Cannon et al. 2024). As the interaction score depends on the number of interacting partners and the available literature, it may change over time as new sources are incorporated. However, between updates, the score remains static regardless of the search set, ensuring consistency in the analysis (Cannon et al. 2024).

**FIGURE 1:**
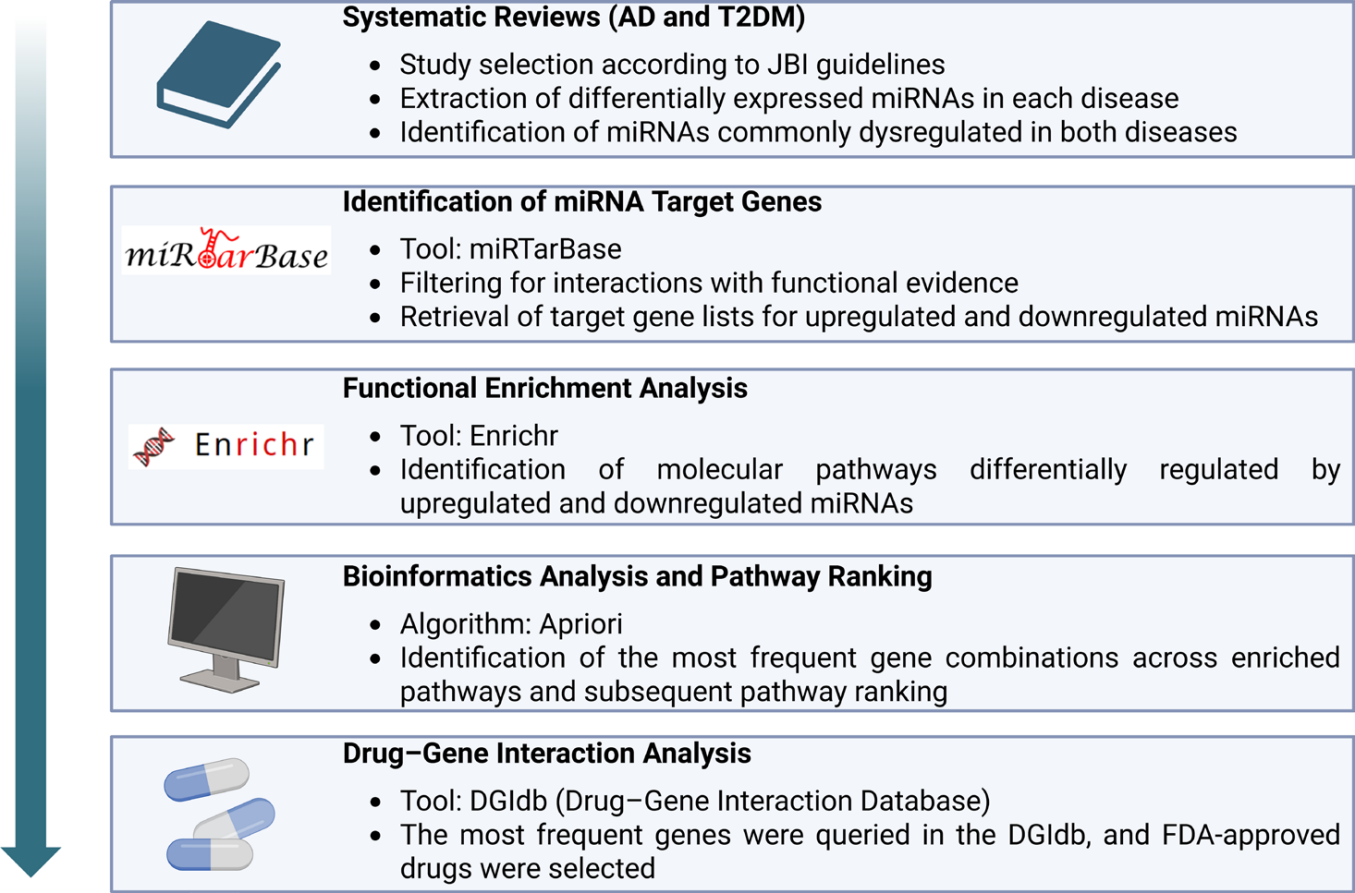
Study workflow. Schematic diagram summarizing the methodological steps used in this study, including systematic reviews, identification of miRNA target genes (miRTarBase), enrichment analysis (Enrichr), bioinformatics‐based pathway ranking (Apriori algorithm), and drug–gene interaction analysis (DGIdb), focusing on FDA‐approved drugs.

Figure 1 summarizes the methodological pipeline, including systematic reviews, miRNA target identification, pathway enrichment analysis, gene combination, pathway ranking, and identification of potential drug–gene interactions.

## 3 Results

### 3.1 Alzheimer's Disease Systematic Review

#### 3.1.1 Selection of Studies

The flowchart used for the selection of studies included in the systematic review of AD is illustrated in Supporting Information S2. The initial search identified 2025 studies, distributed as follows: 286 in the PubMed database, 24 in Cochrane, 209 in EMBASE, 210 in CINAHL, 828 in Scopus, and 468 in Web of Science. Additionally, seven studies were found in the gray literature, and three through manual reference searches in four review articles on the same topic. Out of the total articles/studies found, 719 were excluded as duplicates. The remaining 1306 articles were subjected to a more detailed assessment by reading titles and abstracts, resulting in the exclusion of 1116 articles that did not meet the inclusion criteria. Thus, 190 references were included for full‐text evaluation, but only 186 were effectively assessed, as the full text of four references was not available. Regarding gray literature and manual search, 10 studies were included for full‐text evaluation. Of the 196 articles/studies fully evaluated, 147 were excluded for the following reasons: 3 used animal or in vitro models; 12 did not quantify the miRNAs of interest or were intervention or only bioinformatics studies; 97 did not present a control group or did not use plasma/serum samples; 16 did not perform comparisons between the control and AD groups; 10 did not report diagnostic criteria for AD or the basic characteristics of the participants; and 9 were duplicates identified later. Therefore, 49 studies that met the established inclusion criteria were included in the review.

#### 3.1.2 Characteristics of the Included Studies

The characteristics of the participants in the studies included in the AD review are described in Supporting Information S5. Of the studies reported, two involved patients from Canada (Bhatnagar et al. 2014; Zirnheld et al. 2016), 19 from China (Cao et al. 2020; Dong et al. 2015, 2021; Guo et al. 2017; Han, Guo, et al. 2020; Jia and Liu 2016; Li et al. 2019; Liu and Lei 2021; Tan, Yu, Liu, et al. 2014; Tan, Yu, Tan, et al. 2014; Wang et al. 2020, 2023; Wang and Zhang 2020; Wu et al. 2017; Zeng et al. 2017; Zhang, Liu, et al. 2021; Zhang, Han, et al. 2021; Zhu et al. 2015; Zhang et al. 2022), two from Egypt (Sabry et al. 2021; Abuelezz et al. 2022), one from Germany (Denk et al. 2018), five from Iran (Abolghasemi et al. 2021; Hajjri et al. 2020; Heydari et al. 2021; Poursaei et al. 2022; Biglari et al. 2022), one from Iraq (Abed et al. 2023), seven from Italy (Barbagallo et al. 2020; Galimberti et al. 2014; Giuliani et al. 2021; Maffioletti et al. 2019; Mancuso et al. 2019; Ragusa et al. 2016; Piscopo et al. 2023), two from Japan (Hara et al. 2017; Kiko et al. 2014), one from Korea (Kim, Choi, et al. 2021), one from Montenegro (Dragović et al. 2022), two from Poland (Nagaraj et al. 2017; Prendecki et al. 2019), one from Spain (Cosin‐Tomas et al. 2017), one from Turkey (Guven et al. 2021), one from the United Kingdom (Zhao et al. 2020) and three from the USA (Burgos et al. 2014; Geekiyanage et al. 2012; Kumar et al. 2017).

Regarding the diagnostic criteria used in the studies, one study applied the Diagnostic and Statistical Manual of Mental Disorders—4th Edition (DSM‐IV) (Poursaei et al. 2022) and three employed the 5th edition of the same manual (DSM‐V) (Abolghasemi et al. 2021; Sabry et al. 2021; Abed et al. 2023); 33 used the National Institute of Neurological and Communicative Disorders and Stroke—Alzheimer's Disease and Related Disorders Association (NINCDS‐ADRDA) (Barbagallo et al. 2020; Bhatnagar et al. 2014; Cao et al. 2020; Denk et al. 2018; Galimberti et al. 2014; Guo et al. 2017; Guven et al. 2021; Hajjri et al. 2020; Hara et al. 2017; Heydari et al. 2021; Jia and Liu 2016; Kiko et al. 2014; Kim, Choi, et al. 2021; Kumar et al. 2017; Li et al. 2019; Liu and Lei 2021; Maffioletti et al. 2019; Mancuso et al. 2019; Prendecki et al. 2019; Ragusa et al. 2016; Tan, Yu, Tan, et al. 2014; Tan, Yu, Liu, et al. 2014; Wang and Zhang 2020; Wang et al. 2020; Wu et al. 2017; Zhang, Han, et al. 2021; Zhang, Liu, et al. 2021; Zhao et al. 2020; Zhu et al. 2015; Zirnheld et al. 2016; Abuelezz et al. 2022; Biglari et al. 2022; Zhang et al. 2022). One study adopted NINDS‐AA: National Institute of Neurological Disorders and Stroke—Alzheimer's Association (Dong et al. 2015) and another one employed the National Institute on Aging (NIA criteria) (Geekiyanage et al. 2012); three studies adopted National Institute on Aging—Alzheimer's Association (NIA‐AA) criteria (Zeng et al. 2017; Wang et al. 2023; Dragović et al. 2022) and one adopted the National Institute on Aging—Revised (NIA‐R) (Burgos et al. 2014). Other studies described a combination of two or more criteria, such as DSM‐IV+ NINCDS‐ADRDA in three studies (Giuliani et al. 2021; Han, Guo, et al. 2020; Piscopo et al. 2023), DSM‐IV+ NINCDS‐ADRDA+NIA‐AA in one study (Nagaraj et al. 2017) and International Working Group (IWG) + NIA‐AA in one study (Cosin‐Tomas et al. 2017); finally, one study used the National Institute of Neurology and the Institute of Alzheimer's Disease (Dong et al. 2021).

Among the included studies, 11 did not report whether there was a significant difference in age between patients with AD and the control group (Geekiyanage et al. 2012; Han, Guo, et al. 2020; Heydari et al. 2021; Kiko et al. 2014; Kumar et al. 2017; Prendecki et al. 2019; Sabry et al. 2021; Zhu et al. 2015; Zirnheld et al. 2016; Biglari et al. 2022; Piscopo et al. 2023). In addition, two reported a significant difference between the groups, indicating that AD patients were older than the control participants (Barbagallo et al. 2020; Maffioletti et al. 2019). Regarding sex, two studies did not provide information on the participants' sex (Bhatnagar et al. 2014; Han, Guo, et al. 2020) and one study reported a significant difference between the groups, with the AD group consisting exclusively of men, while the control group included only women (Kumar et al. 2017). Another 13 studies did not report whether there was a significant difference in sex distribution between the groups (Barbagallo et al. 2020; Bhatnagar et al. 2014; Geekiyanage et al. 2012; Han, Guo, et al. 2020; Heydari et al. 2021; Kiko et al. 2014; Kim, Choi, et al. 2021; Prendecki et al. 2019; Sabry et al. 2021; Zhu et al. 2015; Zirnheld et al. 2016; Biglari et al. 2022; Piscopo et al. 2023).

Only 34 studies applied the Mini Mental State Examination (MMSE) cognitive test in both groups (Bhatnagar et al. 2014; Cao et al. 2020; Cosin‐Tomas et al. 2017; Dong et al. 2015, 2021; Galimberti et al. 2014; Geekiyanage et al. 2012; Giuliani et al. 2021; Guo et al. 2017; Guven et al. 2021; Hajjri et al. 2020; Hara et al. 2017; Kiko et al. 2014; Kim, Choi, et al. 2021; Kumar et al. 2017; Li et al. 2019; Liu and Lei 2021; Maffioletti et al. 2019; Mancuso et al. 2019; Prendecki et al. 2019; Sabry et al. 2021; Tan, Yu, Tan, et al. 2014; Tan, Yu, Liu, et al. 2014; Wang and Zhang 2020; Wang et al. 2020; Wu et al. 2017; Zeng et al. 2017; Zhang, Han, et al. 2021; Zhang, Liu, et al. 2021; Zirnheld et al. 2016; Abuelezz et al. 2022; Biglari et al. 2022; Zhang et al. 2022; Dragović et al. 2022). Five studies applied the MMSE only in the AD group (Barbagallo et al. 2020; Nagaraj et al. 2017; Han, Guo, et al. 2020; Jia and Liu 2016; Piscopo et al. 2023). One study applied the Alzheimer's Disease Assessment Scale‐Cognitive (ADAS‐Cog) cognitive test (Abed et al. 2023). Finally, nine studies did not cite the cognitive test applied (Abolghasemi et al. 2021; Burgos et al. 2014; Denk et al. 2018; Heydari et al. 2021; Poursaei et al. 2022; Ragusa et al. 2016; Zhao et al. 2020; Zhu et al. 2015; Wang et al. 2023).

#### 3.1.3 Assessment of the bias Risk

Two studies presented a high risk of bias (Han, Guo, et al. 2020; Heydari et al. 2021). The first one did not mention the origin of the patients, even though the authors' hospital affiliation was provided. It also did not specify whether cognitive tests were performed in the control group, nor did it mention the sex of the patients (Han, Guo, et al. 2020). The second did not mention if the cognitive test was performed or whether there were differences in age and sex between the control and AD groups (Heydari et al. 2021). Also, two presented a moderate risk (Barbagallo et al. 2020; Bhatnagar et al. 2014); the first one did not perform the MMSE test on the control group (Barbagallo et al. 2020). The second did not mention the sex of the patients (Bhatnagar et al. 2014). Finally, the remaining 45 studies presented a low risk of bias. Evaluation of the articles is presented in Supporting Information S6 and S7.

#### 3.1.4 Differentially Expressed miRNAs in Alzheimer's Disease Group

A total of 124 miRNAs, which were quantified using qPCR with Reverse Transcription (RT‐qPCR) or sequencing, showed differential expression in AD patients compared to controls. Data can be found in Supporting Information S8.

### 3.2 Type 2 Diabetes Mellitus Systematic Review

#### 3.2.1 Selection of Studies

The flowchart used for the selection of studies included in the systematic review of T2DM is illustrated in Supporting Information S3. In the initial phase of the search, 2380 studies were identified across different databases: 510 in Pubmed, 148 in Cochrane, 213 in EMBASE, 153 in Cinahl, 1058 in Scopus, and 298 in Web of Science. Additionally, seven studies were identified in gray literature, and 12 were obtained through manual searching of the reference lists of four review articles related to the topic. From the total set of identified studies, 953 were excluded due to being duplicates. The remaining 1427 articles were subjected to detailed evaluation through title and abstract analysis, resulting in the exclusion of 1172 that did not meet the inclusion criteria. Consequently, 255 references were initially considered for full‐text evaluation, of which only 250 were actually analyzed, as the full text of 5 references was not available. Regarding gray literature and manual search, 19 studies were included for full‐text evaluation. Among the 269 studies evaluated in full, 165 were excluded for the following reasons: five used animal or in vitro models; 24 were reviews, intervention studies, or observational studies that did not quantify the miRNAs of interest or bioinformatics studies; 89 did not have a control group or did not use plasma/serum samples; 31 did not perform a comparison between the control and test groups; 13 did not report diagnostic criteria for T2DM or the basic characteristics of the participants, and 3 were duplicates identified later. Thus, 104 studies were included in the review, as they met the established inclusion criteria.

#### 3.2.2 Characteristics of the Included Studies

The characteristics of the participants in the studies included in the systematic review of T2DM are described in Supporting Information S9. Among the included studies, 47 involved patients from China (Yang et al. 2016, 2014, 2017; Wan et al. 2020; Kong et al. 2011, 2022; Li et al. 2016, 2020; Liu et al. 2014; Wang et al. 2018, 2016; Wu et al. 2015; Zhang et al. 2013, 2017; Lu et al. 2021; Wang, Zheng, et al. 2019; Zhou et al. 2020; Wan et al. 2017; Rong et al. 2013; Wang, Wang, et al. 2019; Liang, Li, et al. 2020; Ma et al. 2016; Sun et al. 2014, 2023, 2022; Yan et al. 2016; Nie et al. 2020; Luo et al. 2020, 2019; Shao et al. 2017; Lv et al. 2015; Huang et al. 2018; Liang et al. 2018; Guo et al. 2023; Hu et al. 2022; Lin et al. 2021; Liu, Chen, et al. 2022; Liu, Wang, et al. 2022; Meng et al. 2023; Pan et al. 2022; Ruan et al. 2023; Su et al. 2022; Wu, Xie, et al. 2021; Wu, Li, et al. 2021; Yun et al. 2022; Zhao et al. 2023; Zou et al. 2017), two from Ecuador (Baldeon Rojas et al. 2016; Baldeon et al. 2014), 11 from Egypt (Rezk et al. 2016; Amr et al. 2018; Motawi et al. 2018; Abdelaty et al. 2020; Seleem et al. 2019; Motawae et al. 2015; Shaker et al. 2019; Abdel‐Tawab et al. 2023; Abdou et al. 2022; Ezzat et al. 2023; Saleh et al. 2022), one from Hungary (Fejes et al. 2017), seven from India (Sucharita et al. 2018; Ali Beg et al. 2020; Khan et al. 2020; Prabu et al. 2015, 2020; Alfaifi et al. 2020; Banerjee et al. 2022), one from Indonesia (Tursinawati et al. 2022), 14 from Iran (Alipoor et al. 2018; Akhbari et al. 2018; Parsa et al. 2020; Monfared et al. 2020, 2022; Sadeghzadeh et al. 2020; Zeinali et al. 2021; Samanian et al. 2019; Aghaei Zarch et al. 2024; Ghoreishi et al. 2022; Mokhtari Ardekani et al. 2023; Ghaneh et al. 2023; Shahouzehi et al. 2021; Yazdanpanah et al. 2022), seven from Italy (de Candia et al. 2017; Olivieri et al. 2015, 2014; Mensa et al. 2019; La Sala et al. 2019; Del Cuore et al. 2023; Greco et al. 2023), one from Japan (Higuchi et al. 2015), one from Lithuania (Simoniene et al. 2022), one from Mexico (Garcia‐Jacobo et al. 2019), one from Peru (Espinoza 2018), one from Romania (Nemecz et al. 2023), one from Russia (Tonyan et al. 2023), one from South Africa (Dias 2016), two from Spain (Pescador et al. 2013; Ortega et al. 2014), one from Turkey (Al‐Hayali et al. 2019), two from United Arab Emirates (Elemam et al. 2021; Aljaibeji et al. 2022), one from the USA (Seyhan et al. 2016) and one from Vietnam (Dzung et al. 2023).

The diagnostic criteria for T2DM varied among the studies. Thirty‐eight studies followed the guidelines established by the American Diabetes Association (ADA) (de Candia et al. 2017; Yang et al. 2017; Rezk et al. 2016; Sucharita et al. 2018; Ortega et al. 2014; Amr et al. 2018; Motawi et al. 2018; Abdelaty et al. 2020; Olivieri et al. 2015, 2014; Akhbari et al. 2018; Yan et al. 2016; Shaker et al. 2019; Garcia‐Jacobo et al. 2019; Zeinali et al. 2021; Shao et al. 2017; Lv et al. 2015; Samanian et al. 2019; La Sala et al. 2019; Al‐Hayali et al. 2019; Abdel‐Tawab et al. 2023; Abdou et al. 2022; Aghaei Zarch et al. 2024; Banerjee et al. 2022; Del Cuore et al. 2023; Dzung et al. 2023; Greco et al. 2023; Lin et al. 2021; Liu, Wang, et al. 2022; Meng et al. 2023; Mokhtari Ardekani et al. 2023; Nemecz et al. 2023; Ruan et al. 2023; Saleh et al. 2022; Ghaneh et al. 2023; Shahouzehi et al. 2021; Wu, Li, et al. 2021; Yazdanpanah et al. 2022), while 30 studies applied the criteria of the World Health Organization (WHO) (Yang et al. 2014, 2016; Li et al. 2016; Liu et al. 2014; Wang et al. 2018, 2016; Zou et al. 2017; Lu et al. 2021; Zhou et al. 2020; Wan et al. 2017; Rong et al. 2013; Liang, Li, et al. 2020; Ma et al. 2016; Sun et al. 2014, 2023, 2022; Prabu et al. 2015, 2020; Monfared et al. 2020, 2022; Luo et al. 2020, 2019; Huang et al. 2018; Liang et al. 2018; Dias 2016; Ezzat et al. 2023; Ghoreishi et al. 2022; Kong et al. 2022; Tonyan et al. 2023; Pan et al. 2022). Also, one study described the use of both WHO/IDF criteria (Pescador et al. 2013) and another used both ADA and WHO criteria (Yun et al. 2022). Additionally, in two studies, patients were diagnosed by The Expert Committee on the diagnosis and classification of DM guidelines (Baldeon Rojas et al. 2016; Baldeon et al. 2014), one by the American Association of Clinical Endocrinologists (Nie et al. 2020). In addition, one study used Standards of Medical Care in Diabetes (Zhang et al. 2017). Finally, 30 studies used laboratory tests such as fasting glucose, hemoglobin A1C, and/or the oral glucose tolerance test, without explicitly referencing specific guidelines (Kong et al. 2011; Alipoor et al. 2018; Ali Beg et al. 2020; Wu et al. 2015; Zhang et al. 2013; Seyhan et al. 2016; Wang, Zheng, et al. 2019; Seleem et al. 2019; Motawae et al. 2015; Higuchi et al. 2015; Fejes et al. 2017; Wang, Wang, et al. 2019; Wan et al. 2020; Parsa et al. 2020; Li et al. 2020; Khan et al. 2020; Elemam et al. 2021; Mensa et al. 2019; Sadeghzadeh et al. 2020; Alfaifi et al. 2020; Espinoza 2018; Aljaibeji et al. 2022; Guo et al. 2023; Liu, Chen, et al. 2022; Simoniene et al. 2022; Su et al. 2022; Tursinawati et al. 2022; Wu, Xie, et al. 2021; Zhao et al. 2023; Hu et al. 2022).

Among the included studies, seven did not report whether there was a significant difference in age between patients with T2DM and the control group (Yang et al. 2017; Ali Beg et al. 2020; Wang et al. 2018; Wan et al. 2020; Akhbari et al. 2018; Abdou et al. 2022; Ghaneh et al. 2023). Also, 14 reported a significant difference between the groups, with T2DM patients being older than the control participants (Baldeon Rojas et al. 2016; Pescador et al. 2013; Sucharita et al. 2018; Ortega et al. 2014; Seyhan et al. 2016; Higuchi et al. 2015; Baldeon et al. 2014; Aljaibeji et al. 2022; Kong et al. 2022; Pan et al. 2022; Sun et al. 2023; Tonyan et al. 2023; Tursinawati et al. 2022; Wu, Li, et al. 2021). Regarding sex, six studies did not provide information on the participants' sex (Wu et al. 2015; Zhou et al. 2020; Seleem et al. 2019; Ma et al. 2016; Monfared et al. 2020; Yang et al. 2016). Another 25 did not report whether there was a statistically significant difference in sex between the groups (Baldeon Rojas et al. 2016; Yang et al. 2014, 2017, 2016; Ali Beg et al. 2020; Wang et al. 2018; Wu et al. 2015; Motawi et al. 2018; Zhou et al. 2020; Seleem et al. 2019; Higuchi et al. 2015; Ma et al. 2016; Akhbari et al. 2018; Yan et al. 2016; Baldeon et al. 2014; Garcia‐Jacobo et al. 2019; Monfared et al. 2020; Luo et al. 2019; Abdel‐Tawab et al. 2023; Abdou et al. 2022; Aljaibeji et al. 2022; Nemecz et al. 2023; Ghaneh et al. 2023; Tonyan et al. 2023; Wu, Li, et al. 2021).

#### 3.2.3 Risk of Bias

Only 21 studies showed a moderate risk of bias (Pescador et al. 2013; Wu et al. 2015; Ortega et al. 2014; Lu et al. 2021; Zhou et al. 2020; Higuchi et al. 2015; Fejes et al. 2017; Alfaifi et al. 2020; Olivieri et al. 2014; Yang et al. 2016; Espinoza 2018; Abdou et al. 2022; Banerjee et al. 2022; Ezzat et al. 2023; Kong et al. 2022; Monfared et al. 2022; Nemecz et al. 2023; Simoniene et al. 2022; Pan et al. 2022; Sun et al. 2023; Wu, Li, et al. 2021) due to differences between groups or incomplete information regarding age, sex, or BMI. The remaining 83 studies exhibited a low risk of bias. The evaluation of the articles is detailed in Supporting Information S10 and S11.

#### 3.2.4 Differentially Expressed miRNAs in T2DM Patients

The method used for detecting miRNAs in these studies was only RT‐qPCR. A total of 286 miRNAs were differentially expressed in T2DM patients compared to controls. Data can be found in Supporting Information S12.

### 3.3 miRNAs Commonly Expressed in Alzheimer's Disease and Type 2 Diabetes Mellitus

**FIGURE 2:**
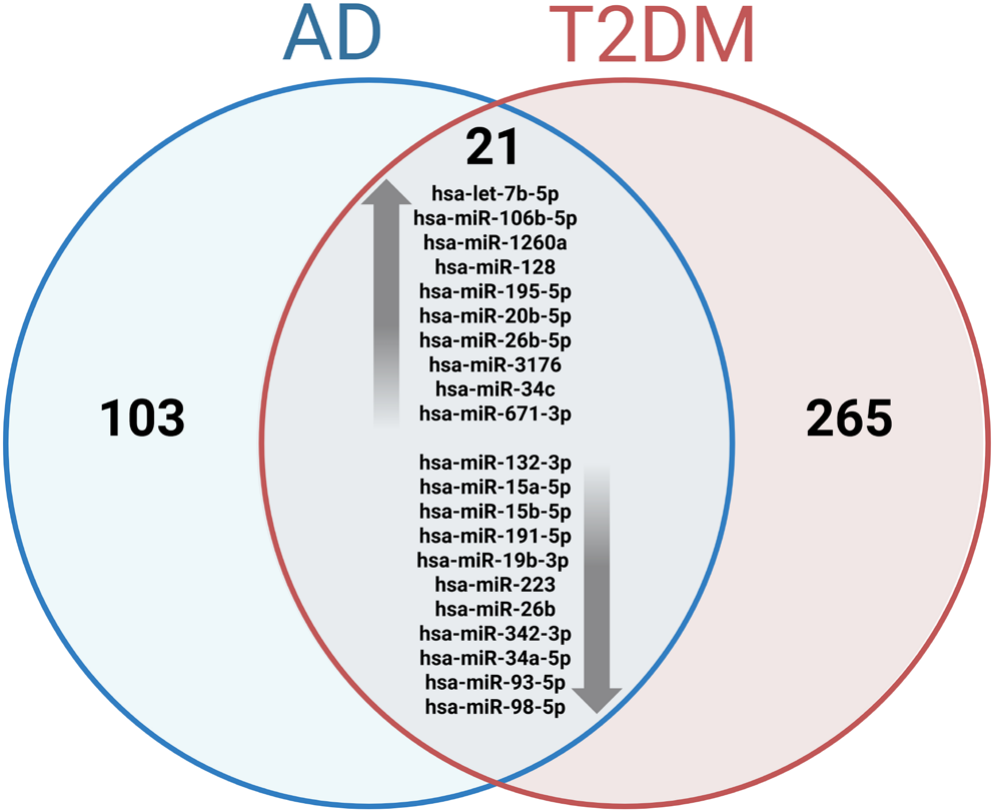
Venn diagram of differentially expressed miRNAs in Alzheimer's disease (AD) and type 2 diabetes mellitus (T2DM). A total of 124 miRNAs were identified in AD and 286 in T2DM. Among these, 21 miRNAs were commonly dysregulated in both conditions. Upregulated miRNAs in both diseases are shown above the arrow, while downregulated miRNAs are shown below.

When analyzing data on miRNAs differentially expressed in patients with AD and T2DM, we identified 21 miRNAs commonly dysregulated in both conditions—10 upregulated and 11 downregulated—considering the family or the specific isoform when related (Figure 2). To ensure consistency, we excluded data with conflicting findings regarding the expression of miRNAs, specifically those cases where the same miRNA was reported as upregulated in some studies and as downregulated in others under the same condition. Additionally, miRNAs that were found to be upregulated in one disease and downregulated in the other, and vice versa, were also excluded from the analysis.

### 3.4 Bioinformatics Analyses

#### 3.4.1 Identification of Target Genes of the Selected miRNAs

**FIGURE 3:**
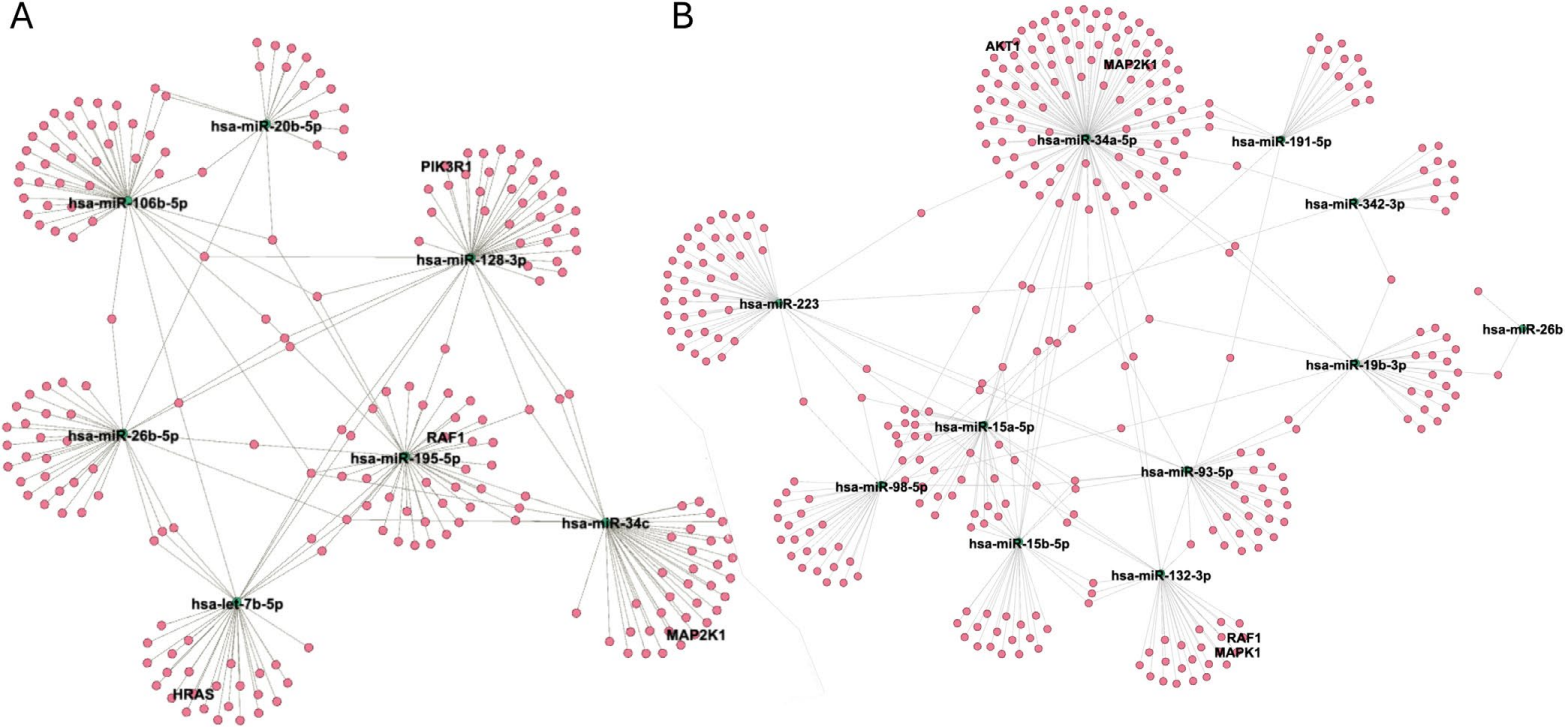
Network of miRNA‐gene interactions between the microRNA targets. Green dots represent microRNAs, and pink dots represent their predicted gene targets. Among the target genes, those that are also part of the enriched pathways are specifically highlighted. The network layout was generated using the ForceAtlas2 algorithm in Gephi. (A) Network of downregulated miRNAs. (B) Network of upregulated miRNAs.

The target genes of the miRNAs were extracted from miR‐Tar‐Base, and the following miRNAs were not found in the latest version of this database with “Functional miRNA–Target Interaction (MTI)” as Support type: miR‐1260a, miR‐3176, miR‐671‐3p and miR‐181c‐3p. Therefore, it was wound a total of 233 targets for upregulated miRNAs and 337 targets for downregulated miRNAs. Figure 3 shows network of miRNA‐gene interactions between the microRNA targets.

#### 3.4.2 Pathway Analyses

Functional enrichment analysis was performed and two tables were generated: one containing all pathways associated with the targets of downregulated miRNAs, and another including all pathways associated with the targets of upregulated miRNAs. Pathways with the lowest adjusted *p*‐values up to “E‐05” were included. The pathways in the final model are listed in Supporting Information S13.

#### 3.4.3 Selection of the Most Relevant Pathways

A total of 265 pathways targeted by downregulated miRNAs and 243 pathways targeted by upregulated miRNAs were identified. Given the large number of pathways, an analysis using algorithms was performed to determine which pathways were the most relevant (Supporting Information S13).

##### 3.4.3.1 Analysis of Gene Combination Frequency in Pathways

Each gene was represented as an item in a set corresponding to the pathway. The Apriori algorithm was applied to determine the frequency of specific gene combinations appearing in the pathways. From the analysis of pathways targeted by downregulated miRNAs, the most frequent gene combination was mitogen‐activated protein kinase kinase 1 (*MAP2K1*)/mitogen‐activated protein kinase 1 (*MAPK1*), with the highest support value (0.2943). Additionally, the genes *MAP2K1*, *MAPK1*, AKT serine/threonine kinase 1 (*AKT1*), and Raf‐1 proto‐oncogene (*RAF1*) frequently appeared in various combinations, with support values equal to or greater than 0.2000: *AKT1/MAPK1*: 0.2830; *MAP2K1/RAF1*: 0.2528; *MAPK1/RAF1*: 0.2453; *MAP2K1/MAPK1/RAF1*: 0.2226; *AKT1/MAP2K1*: 0.2000; and *AKT1/MAP2K1/MAPK1*: 0.200 (Supporting Information S14).

For pathways targeted by upregulated miRNAs, the most frequent gene combination was *MAP2K1/RAF1*, which had the highest support value (0.2840). Other frequent combinations included the genes *MAP2K1*, *RAF1*, HRas proto‐oncogene (*HRAS*), and phosphoinositide‐3‐kinase regulatory subunit 1 (*PIK3R1*): *HRAS/PIK3R1*: 0.2757; *HRAS/RAF1*: 0.2716; *HRAS/MAP2K1*: 0.2634; *MAP2K1/PIK3R1*: 0.2510; *HRAS/MAP2K1/RAF1*: 0.2469; and *PIK3R1/RAF1*: 0.2305, also support values equal to or greater than 0.2000 (Supporting Information S15).

##### 3.4.3.2 Pathway Ranking Based on the Average Support of all Items

Pathway ranking was performed using the average support of the genes within each pathway. For this, the support of each gene was calculated individually, and the average support of all genes in the pathway was used to assign a score. According to the results, the most significant pathway targeted by downregulated miRNAs was T‐cell receptor–RAS (TCR‐RAS) signaling cascade (PID_TCR_RAS_PATHWAY), while the most significant pathway targeted by upregulated miRNAs was the extracellular matrix (ECM) signaling pathway (BIOCARTA_ECM_PATHWAY) (Supporting Information S16).

### 3.5 Suggested Therapeutic Drugs Based on Drug‐Gene Interaction Analysis

The drug‐gene interaction analysis identified FDA‐approved drugs targeting genes associated with differentially expressed microRNAs. For target genes of downregulated microRNAs, *AKT1_HUMAN* is inhibited by Capivasertib (interaction score: 0.51) and Nelfinavir (0.12). *MAPK1*, another downregulated microRNA target, was found to be regulated by benzalkonium chloride (0.14).

As described before, the genes *MAP2K1 and RAF1* were identified as targets of both downregulated and upregulated microRNAs. *MAP2K1* is regulated by Cobimetinib (0.56), Trametinib dimethyl sulfoxide (0.54), Selumetinib (0.47), Binimetinib (0.4), Dabrafenib (0.34), Vemurafenib (0.33); Cobimetinib fumarate (0.31), Selumetinib sulfate (0.31), Encorafenib (0.27), Panitumumab (0.18) and Cetuximab (0.16). *RAF1_HUMAN* is inhibited by Tovorafenib (0.66), Encorafenib (0.22), Regorafenib (0.18) and Sorafenib (0.15).

**FIGURE 4:**
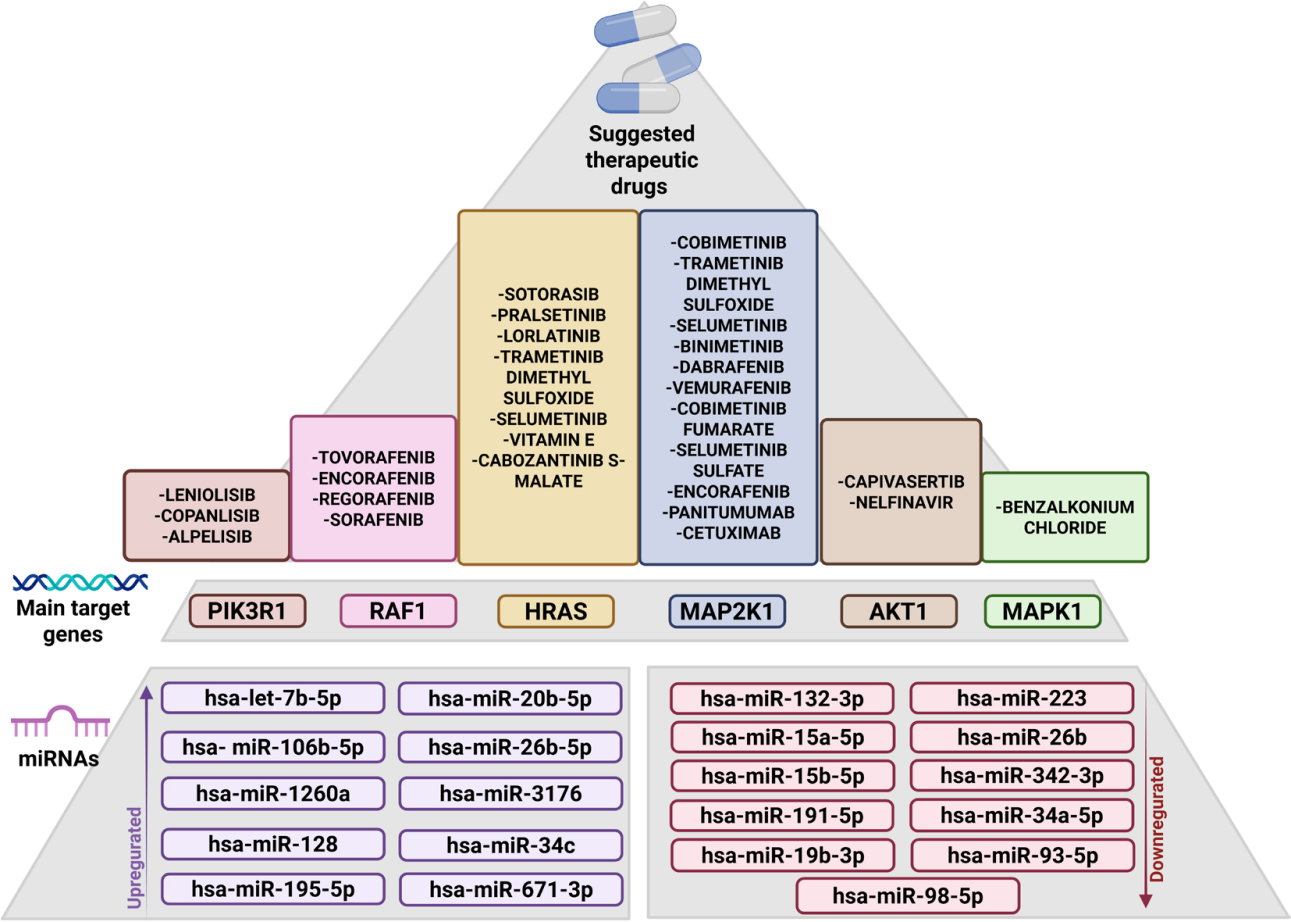
Predicted therapeutic drugs targeting key genes regulated by commonly dysregulated miRNAs in Alzheimer's disease (AD) and type 2 diabetes mellitus (T2DM). This integrative analysis identified six genes (PIK3R1, RAF1, HRAS, MAP2K1, AKT1, and MAPK1) as potential therapeutic targets, which are regulated by miRNAs found to be upregulated (left) or downregulated (right) in both AD and T2DM. The diagram displays the respective miRNAs (bottom) and the drugs predicted to interact with these targets (top), based on drug–gene interaction databases.

For target genes of upregulated miRNAs, *PIK3R1* was found to be regulated by Leniolisib (0.33), Copanlisib (0.18), and Alpelisib (0.11). *HRAS* is regulated by Sotorasib (0.41), Pralsetinib (0.17), Lorlatinib (0.14), Trametinib dimethyl sulfoxide (0.14), Selumetinib (0.11), Vitamin E (0.11), and Cabozantinib S‐malate (0.11). The data is shown in Supporting Information S17 and Figure 4.

## 4 Discussion

Alterations in microRNA expression profiles have been reported in both AD and T2DM, suggesting that these small non‐coding RNAs may play a role in the shared molecular mechanisms underlying both diseases. However, the identification of circulating miRNAs that are commonly dysregulated in both conditions, along with their target genes and pathways, remains limited. To address this gap, the present study investigated serum/plasma miRNAs simultaneously associated with AD and T2DM, and identified shared genes and regulatory pathways through a combination of systematic review, bioinformatics, and artificial intelligence approaches. We found that 21 miRNAs were identified as commonly expressed in both diseases, and 337/233 genes are potential targets for these down‐ and upregulated miRNAs, respectively. The biological pathways identified from those genes were the TCR‐RAS and the extracellular matrix. Therefore, our findings provide insights into their shared pathophysiology and for potential therapeutic targets.

### 4.1 Target Genes

The genes ***MAP2K1*** and ***MAPK1*** encode proteins of the MAP kinase family, also known as extracellular signal‐regulated kinases (ERKs), which integrate biochemical signals involved in processes such as proliferation, differentiation, transcription regulation, and development (Slattery et al. 2012). MAPK1 leads to the activation of several kinases that are known to be involved in the hyperphosphorylation of tau, such as AKT1, cyclin‐dependent kinase 5 (CDK5), casein kinase II subunit alpha (CSNK2A1), BR serine/threonine kinase 1 and 2 (BRSK1 and 2), and microtubule affinity‐regulating kinase 1 and 2 (MARK1 and 2) (Alves et al. 2022; Gerschutz et al. 2014). In AD, miRNAs are known to modulate the MAPK signaling pathway, thereby contributing to Aβ and tau pathology, oxidative stress, neuroinflammation, and neuronal death (Raffaele et al. 2023; Zhang et al. 2014; Bazrgar et al. 2020). Notably, miR‐132, identified as downregulated in both AD and T2DM in the present study, stands out for its neuroprotective role by reducing Aβ and tau accumulation and mitigating oxidative stress through the modulation of ERK/MAPK1 signaling (Raffaele et al. 2023). Moreover, the upregulation of miR‐132 in a mice model of AD improved cognitive function, reduced oxidative stress, and decreased cell apoptosis. In T2DM, the glucose variability, a known contributor to diabetes complications, was associated with *MAPK1* through its role in insulin secretion, glucose metabolism, and glycogen biosynthesis, emphasizing its central position in diabetes‐related gene networks (Saik and Klimontov 2020). MiR‐34c was identified as upregulated in both AD and T2DM in the present study, and it was shown to downregulate *MAP2K1*, among other targets, to enhance proinsulin synthesis during the formation of insulin‐producing cells (IPCs). However, prolonged dysregulation by miR‐34c led to suppression of additional targets, such as acyl‐CoA synthetase long‐chain family member 4 (*ACSL4*) and secretion‐associated Ras‐related GTPase 1A (*SAR1A*), ultimately impairing insulin secretion in IPCs (Bai et al. 2017). Finally, network pharmacology analysis revealed that *MAPK1* is one of the key targets associated with both T2DM and AD, reflecting the shared pathological mechanisms between these pathologies (Wang et al. 2021). Our findings highlight *MAPK1* and *MAP2K1* as important targets of dysregulated miRNAs shared by AD and T2DM, reinforcing their relevance in the overlapping pathophysiology of these diseases.

The gene ***AKT1*** encodes one of the three members of the AKT serine–threonine protein kinase family, which undergoes phosphorylation via phosphatidylinositol 3‐kinase activity (Shi et al. 2019). The interaction between AKT and PI3K is crucial in various signaling pathways, including those involving ligand interaction with tyrosine kinase receptors, G‐protein‐coupled receptors, and integrin‐linked kinases (Sugiyama et al. 2019). Consequently, AKT proteins regulate a wide range of cellular functions, including cell proliferation, survival, metabolism, and angiogenesis (Nicholson and Anderson 2002). Hyperphosphorylation of *AKT1* in the brain has been associated with increased amyloid burden, tau tangle density, and cognitive decline in AD patients (Arvanitakis et al. 2020). Dysregulated *AKT1* signaling contributes to neuroinflammatory processes and insulin resistance, key mechanisms linking AD and T2DM (El Idrissi et al. 2021; Liao and Xu 2009; Arous et al. 2020). Therapeutic activation of *AKT1* has shown promise in restoring memory recall and synaptic protein synthesis deficits in AD mouse models, suggesting its importance as a target for intervention (Kommaddi et al. 2024). In our study, *AKT1* emerged as a key target of miRNAs dysregulated in both diseases, supporting its involvement in insulin resistance and neurodegeneration.

The gene ***RAF‐1*** encodes RAF‐1, a MAP‐type protein (Yeung et al. 2000). *RAF‐1* has been implicated in AD through its role in the MAPK/ERK signaling pathway. In AD brains, *RAF‐1* shows increased phosphorylation at key sites (Ser338, Tyr340/341, and Ser259), which correlates with its activation and association with downstream MEK1/ERK signaling (Mei et al. 2006). Additionally, bioinformatics analyses identified *RAF‐1* as a key autophagy‐related gene, suggesting potential involvement in AD pathogenesis through dysregulated cellular processes (Li, Liu, Sun, et al. 2023). *RAF‐1* kinase plays a crucial role in the regulation of pancreatic beta‐cell mass and function, key factors in the pathogenesis of diabetes. Studies show that *RAF‐1* mediates insulin‐stimulated beta‐cell proliferation through the MAPK pathway, highlighting its importance in maintaining beta‐cell survival and replication at physiological insulin levels (Johnson and Alejandro 2008; Beith et al. 2008). Furthermore, in diabetic conditions, *RAF‐1* signaling has been implicated in retinal neurodegeneration, with its regulation potentially mitigating apoptosis and glial activation in hyperglycemic environments (Wu et al. 2022). In our analysis, *RAF‐1* was identified as a miRNA target, showing that it plays an important role in cellular processes mediated by insulin.

The gene ***HRAS*** (HRas proto‐oncogene, GTPase) encodes proteins from the GTPase family related to the regulation of growth, differentiation, and survival of many cell types (Pazik et al. 2021). The role of HRAS in AD was previously explored using APP/PS1 transgenic mice, and it was shown that the absence of HRAS improved spatial memory deficits. Moreover, *HRAS* deletion reduced cortical amyloid deposition, astroglial activation, and the loss of dendritic spines linked to amyloid plaques (Qu et al. 2023). Finally, *HRAS* was recently identified as a key hub gene in immune‐related mechanisms of AD pathogenesis through transcriptomic analysis of immune‐related differentially expressed RNAs and protein–protein interaction network construction (Xu and Jia 2020). Regarding T2DM, *HRAS* was identified as a key driver within the gene‐regulatory networks associated with the insulin‐like growth factor I/insulin resistance axis, highlighting its potential role in the metabolic pathways underlying T2DM (Jung 2021). Also, another study demonstrated that targeted *HRAS* expression in pancreatic beta cells induces beta‐cell degeneration, leading to hyperglycemia, reduced plasma insulin levels, and diabetes, with associated structural damage to the islets of Langerhans and stress in the endoplasmic reticulum (Efrat et al. 1990). The identification of *HRAS* as a target of dysregulated miRNAs in our study reinforces its role in immune activation and cell degeneration, a condition commonly observed in AD and T2DM.

The gene ***PIK3R1*** (phosphoinositide‐3‐kinase regulatory subunit 1) encodes phosphatidylinositol 3‐kinase, which plays a critical role in insulin metabolic actions. Pathogenic variants of ***PIK3R1*** have been associated with insulin resistance (Tsay and Wang 2023). Also, *PIK3R1* is a key gene in the neurotrophin signaling pathway, and genetic variants have been associated with the progression from mild cognitive impairment to AD, with evidence suggesting that these variants may regulate *PIK3R1* expression and contribute to disease development (Li, Liu, Lutz, et al. 2023). Moreover, *PIK3R1* was identified as one of the key immune hub genes closely associated with tau and Aβ pathology in AD, with its involvement linked to abnormal peripheral immune cell infiltration and pathways such as axon guidance, long‐term potentiation, and cytokine–cytokine receptor interactions (Mei et al. 2006). *PIK3R1* also plays a critical role in insulin signaling by regulating phosphoinositide 3‐kinase (PI3K) activity, which is essential for glucose uptake and metabolic homeostasis. Variants in the *PIK3R1* gene have been associated with increased susceptibility to T2DM (Mir et al. 2021). Reduced expression of *PIK3R1* improves insulin sensitivity and glucose tolerance, as shown in animal models, suggesting its potential as a therapeutic target (Mauvais‐Jarvis et al. 2002). Furthermore, meta‐analyses and network‐based studies identified *PIK3R1* as a hub gene in T2DM‐related pathways, including PI3K‐Akt signaling, linking it to key metabolic and cellular processes (Zhu et al. 2020; Rasche et al. 2008; Zhang et al. 2024). Our results support these findings, identifying *PIK3R1* as a key target gene, underscoring its integrative role in insulin resistance and abnormal immune response pathways, connecting AD and T2DM.

### 4.2 Biological Pathway

#### 4.2.1 TCR‐RAS Pathway

The most significant pathway targeted by the downregulated miRNAs was the TCR‐RAS signaling cascade. The TCR signaling pathway is essential for T‐cell activation and immune response regulation. This cascade begins with the recognition of antigens by the T‐cell receptor. This recognition triggers the activation of several intracellular pathways, including the RAS one, which plays a critical role in transducing signals that regulate T‐cell proliferation, differentiation, and effector functions (Hwang et al. 2020). T cells play a key role in neurodegenerative diseases such as AD, particularly when the blood–brain barrier (BBB) is compromised, allowing their infiltration into the central nervous system (Zeng et al. 2024). Under physiological conditions, regulatory T cells (Tregs) suppress effector T cell‐mediated inflammatory responses, maintaining immune balance (DeMaio et al. 2022). In neuroinflammation, brain‐resident Tregs mitigate astrogliosis by producing amphiregulin, polarize microglia toward neuroprotective states, and release interleukin‐10 to suppress inflammation (Liston et al. 2022). However, diminished Treg function in AD contributes to a pro‐inflammatory environment, exacerbating neurodegeneration (DeMaio et al. 2022).

Emerging research on microRNA let‐7b, upregulated in both AD and T2DM according to the present study, has identified its increased expression in CD4+ T cells within the cerebrospinal fluid (CSF) of AD patients (Liu et al. 2018). This elevation correlates with tau protein markers (t‐tau and p‐tau), thereby implicating let‐7b in AD progression (Liu et al. 2018). These findings suggest that let‐7b could serve as a valuable biomarker, improving diagnostic precision when integrated with traditional AD markers (Liu et al. 2018). Additionally, beta‐site APP cleaving enzyme 1 (BACE1), a known driver of amyloid plaque formation, regulates TCR signaling in CD4+ T cells, enhancing interleukin‐17A and CD73 expression in T helper 17 cells (Th17) and regulatory T cells (Dai et al. 2021). Peripheral antagonism of prostaglandin E2 signaling mediated by BACE1 partially reduces T cell overactivation and neuroinflammation, underscoring its role in AD‐related immune dysfunction (Dai et al. 2021).

The adaptive immune response is not only critical in AD, but also influences the pathogenesis of T2DM. Oxidative stress and inflammation in T2DM are associated with increased activation of CD4+ and CD8+ T lymphocytes and elevated proinflammatory cytokine levels, contributing to the activation of inflammatory pathways (Stentz and Kitabchi 2003). Proinflammatory CD4+ Th1, Th17, and CD8+ T cells are expanded, while innate T cell populations, such as mucosal‐associated invariant T cell (MAIT) and invariant natural killer T cell (iNKT), are reduced. These changes disrupt T cell homeostasis, potentially driving tissue and systemic inflammation (Touch et al. 2017).

In T2DM, immune dysregulation contributes to various complications through T cell‐mediated mechanisms. CD8+ T cells accumulate in ischemic tissues, impairing vascular regeneration through their negative impact on angiogenesis (Liang, Yang, et al. 2020). In addition, a deficiency in Tregs contributes to retinal neurodegeneration, gliosis, inflammation, and vascular leakage associated with diabetic retinopathy (Llorian‐Salvador et al. 2024). Notably, systemic Treg expansion in a db/db mouse model effectively reduced these neurodegenerative and inflammatory retinal changes without affecting glycemic or insulin levels (Llorian‐Salvador et al. 2024). Additionally, in diabetic kidney disease, elevated glycemic variability promotes pro‐inflammatory Th17 and Th1 cell expansion and reduces Treg numbers and function, amplifying inflammatory processes (Gu et al. 2023). Finally, a clinical study showed that short‐term intensive insulin therapy could modulate T cell subpopulations in patients with newly diagnosed T2DM (Cheng et al. 2019).

#### 4.2.2 ECM Pathway

Conversely, the ECM pathway was the most significant pathway targeted by upregulated miRNAs, which involves interactions between ECM components, such as collagen, fibronectin, and laminin, and integrin receptors on the cell surface (Valiente‐Alandi et al. 2016). The ECM provides mechanical support to tissues through its interconnected components and acts as a storage site for growth factors and signaling molecules (Yue 2014). Particularly in the central nervous system (CNS), neurons, astrocytes, and oligodendrocytes are responsible for ECM production (Testa et al. 2019), and its composition is distinct from that of other tissues (Anwar et al. 2022). The ECM in the CNS is organized into three primary compartments: the basement membrane (BM), perineuronal nets (PNNs), and the neural interstitial matrix (Anwar et al. 2022). PNNs stabilize synaptic connections through components like lecticans, hyaluronic acid, and tenascins. The neural interstitial matrix, covering 15%–20% of the brain, connects neurons and vasculature via a network of hyaluronan, proteoglycans, and adhesive glycoproteins (Anwar et al. 2022). Additionally, the ECM facilitates bidirectional communication between cells, mediated by integrins, and interacts with growth factors to influence inflammation and neurodegenerative processes (Anwar et al. 2022).

In AD, ECM proteins play a dual role, influencing both disease progression and neuroprotection (Ma et al. 2020). Elevated levels of tenascin‐C in AD contribute to stabilizing perineuronal nets, reducing Aβ clearance, and participating in inflammatory pathways that drive disease progression. PNNs also shield neurons from Aβ toxicity. Heparan sulfate proteoglycans promote Aβ fibril formation, inhibit amyloid hydrolysis, and enhance Aβ production, while dermatan sulfate proteoglycans regulate Aβ plaque size and structure. High molecular‐weight heparin (HP) facilitates β‐sheet formation, increasing Aβ production, whereas low molecular‐weight HP inhibits this process. Reelin, which is reduced in AD, aids in Aβ clearance and suppresses tau phosphorylation. Additionally, changes in hyaluronan metabolism, such as increased short‐chain HA due to tau hyperphosphorylation, disrupt myelination and decrease oxygen and glucose supply to the brain. Tenascin‐R and chondroitin sulfate proteoglycans are elevated in AD, stabilizing PNNs and contributing to synaptic dysfunction, while keratan sulfate proteoglycans (KSPGs), which are decreased, may impair synaptic plasticity (Sun et al. 2021).

Also, collagen IV, laminin, and fibronectin are closely linked to AD pathogenesis, playing crucial roles in basement membrane stability, blood–brain barrier function, and the progression of neurodegenerative processes (Anwar et al. 2022; Reed et al. 2019; Ma et al. 2020). Laminin, a heterotrimeric glycoprotein essential for basement membrane assembly, is predominantly secreted by astrocytes and endothelial cells, forming diverse isoforms such as laminin‐111 and laminin‐421 in the brain (Anwar et al. 2022; Reed et al. 2019). Astrocytic laminin regulates pericyte differentiation and maintains blood brain barrier integrity (Yao et al. 2014). In AD, laminin expression is altered, with reductions observed in the brain microvasculature of transgenic models and BBB dysfunction linked to impaired capillary density (Anwar et al. 2022; Ma et al. 2020). Fibronectin, an ECM protein dimer secreted by endothelial cells, astrocytes, and pericytes, plays a key role in cell attachment and ECM organization through integrins (Anwar et al. 2022). In AD, fibronectin levels are elevated (Bogdan et al. 2022), particularly in association with Aβ plaques and reactive astrocytes, reflecting its involvement in the disease's pathological processes (Moreno‐Flores et al. 2001). Studies in both human and transgenic models have demonstrated increased fibronectin levels in brain tissues and microvessels, correlating with Aβ aggregation and amyloid precursor protein (APP) secretion (Ma et al. 2020; Anwar et al. 2022). Plasma fibronectin has emerged as a promising biomarker, with higher molecular‐weight forms more frequently detected in AD patients than in healthy controls (Lemanska‐Perek et al. 2009).

In the context of T2DM, the ECM undergoes significant alterations that contribute to the development and progression of various complications (Law et al. 2012). Chronic hyperglycemia in T2DM promotes the formation of AGEs, which accumulate in the ECM and induce structural and functional changes (Law et al. 2012; Rojas et al. 2018). These modifications result in increased stiffness and reduced functionality of ECM components, impairing tissue homeostasis (Giri et al. 2018). Additionally, AGEs quench endothelial nitric oxide and generate oxidative stress, exacerbating endothelial dysfunction observed in T2DM (Hayden et al. 2005).

### 4.3 Potential Therapeutic Drugs

In addition to identifying dysregulated miRNAs and their target genes, this study explored potential pharmacological interventions by analyzing drug‐gene interactions using DGIdb. Among the identified drugs, Nelfinavir, an HIV protease inhibitor, has been shown to influence Aβ metabolism by enhancing the secretion of undigested Aβ after phagocytosis and inhibiting endogenous Aβ40 production in primary cultured human cortical neurons (Lan et al. 2012). These effects were associated with a reduction in BACE1 and γ‐secretase activities (Lan et al. 2012). Sorafenib, a multi‐kinase inhibitor approved for cancer treatment, has demonstrated potential neuroprotective effects by modulating key inflammatory pathways in AD (Echeverria et al. 2009). Sorafenib has also been shown to regulate neuroinflammatory responses by suppressing the expression of cyclooxygenase‐2 and interleukin‐1β in microglial cells and modulating the AKT/P38‐STAT3/NF‐kB signaling pathway (Kim, Park, et al. 2021). In lipopolysaccharide‐stimulated wild‐type mice, Sorafenib reduced microglial and astroglial activation, while in a 5xFAD mouse model of AD, it significantly decreased astrocytosis, reinforcing its therapeutic potential in neuroinflammatory conditions (Kim, Park, et al. 2021). Similarly, Regorafenib, another multi‐kinase inhibitor, has a potential role in neuroinflammation (Fiscon et al. 2022). Beyond its kinase inhibition properties, Regorafenib was also found to modulate aberrant glycosylation, a process implicated in AD progression. By targeting glycosylation‐associated mechanisms, Regorafenib improved microglial responses, phagocytosis, and neuroinflammatory balance, ultimately leading to cognitive improvements in AD models (Wang et al. 2024). Moreover, Regorafenib has shown direct effects on AD pathology by reducing Aβ plaque accumulation, decreasing tau phosphorylation, and enhancing dendritic spine density in 5xFAD mice. These effects were associated with the downregulation of glycogen synthase kinase 3 beta (GSK3β) activity (Han, Kang, et al. 2020).

Vitamin E has also been explored for its potential neuroprotective effects in AD, primarily due to its antioxidant properties (Browne et al. 2019). Studies suggest that vitamin E may help reduce oxidative stress, mitigate lipid peroxidation, and slow cognitive decline in AD patients, although its efficacy remains debated, particularly regarding its impact on disease progression and optimal dosage (Lloret et al. 2019). Additionally, Cabozantinib, a tyrosine kinase inhibitor originally approved for cancer treatment, has emerged as a potential therapeutic candidate for AD. A drug screening study based on behavioral profiling identified Cabozantinib within a functional cluster that includes calcineurin inhibitors, which have been suggested for AD prevention due to their immunosuppressive and neuroprotective properties (Tucker Edmister et al. 2022).

### 4.4 Limitations

The present study has some limitations, primarily related to the inherent constraints of the included articles, as well as different diagnostic criteria. Moreover, it is important to consider that more than 50% of individuals diagnosed with Alzheimer's disease have mixed dementia, with more than one identifiable pathological cause, which may impact the specificity of the findings (Reuben et al. 2024).

The absence of subgroup or stratified analyses in most of the included studies, considering variables such as age, sex, comorbidities, and geographic background, is another limitation. Since environmental and genetic factors can influence both diseases and molecular signatures, it is unclear whether the identified miRNA profiles are consistent across different demographic strata. Similarly, no analyses were conducted to address potential subtypes within the AD and T2DM cohorts.

Determining miRNAs in peripheral blood may present variability, since several factors can influence normal circulating miRNA levels, including collection and quantification methods, blood sample processing, and characteristics of individuals such as age, sex, and other comorbidities (Takizawa et al. 2022); which were not fully controlled for in the included studies.

Another limitation is the lack of cross‐validation with transcriptomic datasets or independent experimental cohorts, which restricts the biological validation of the identified targets. Although the use of miRTarBase ensures that only experimentally validated targets were included in pathway enrichment, this database may not yet contain all relevant interactions, especially those from studies that are more recent. Although this limitation may influence the detection of the molecular pathways identified in the present study, it is important to note that miRTarBase remains a valuable tool, as it contains experimentally validated miRNA target genes. MiRNAs are also soft regulators of gene expression and, in many cases, pharmacological and genetic approaches to their silencing do not change much of the desired phenotype (Linsen et al. 2008; Kilikevicius et al. 2022).

### 4.5 Future Directions

Despite the limitation, advances in detection methods and improved techniques have facilitated the more reliable measurement of these miRNAs in bodily fluids, enabling their potential application as disease biomarkers (Mariner et al. 2018). Building upon the exploratory nature of this study, future research should focus on validating the identified miRNAs and target genes through in vitro and in vivo experiments. These validations will be crucial to determine their mechanistic roles in shared molecular pathways and to assess their viability as therapeutic targets.

Additionally, the integration of transcriptomic datasets could strengthen the robustness of future findings by providing cross‐validation of expression patterns. Multi‐omics approaches—including proteomics, metabolomics, and epigenomics—alongside network biology tools, could offer a more comprehensive view of the molecular landscape connecting AD and T2DM.

Another promising direction involves exploring the diagnostic and prognostic value of the identified miRNAs, particularly in individuals with T2DM who are at higher risk of developing AD. Longitudinal studies are needed to determine whether these miRNAs appear in early disease stages and how their levels fluctuate with aging and disease progression. This could clarify whether they act as early biomarkers or reflect downstream pathological changes.

Understanding how these common miRNAs regulate key pathways involved in insulin signaling, neuroinflammation, and synaptic function may provide important insights into how T2DM contributes to the onset and progression of AD. Future studies exploring the modulation of these miRNAs–through pharmacological or genetic approaches–could support the development of targeted therapies aimed at delaying or preventing neurodegeneration in diabetic individuals.

Lastly, the shared miRNAs may not only inform the risk and progression of AD in the context of T2DM but could also modulate key biological processes relevant to both diseases. Understanding how these molecules contribute to or protect against neurodegeneration and metabolic dysfunction could pave the way for personalized and preventive therapeutic strategies.

## 5 Conclusion

This study provides novel evidence that the TCR‐RAS signaling cascade and the ECM pathway are potentially modulated by microRNAs in both AD and T2DM. By uncovering these shared regulatory mechanisms, our findings offer original insights into the molecular crosstalk between these two complex diseases and highlight previously underexplored pathways that may serve as promising targets for future therapeutic interventions.

## Author Contributions

**Lívia Cristina Ribeiro Teixeira:** conceptualization, methodology, software, data curation, writing – original draft, visualization. **Jessica Diniz Pereira:** methodology, software, data curation, visualization. **Izabela Mamede:** methodology, software, data curation, visualization, writing – review and editing. **Paulo Caramelli:** visualization, writing – review and editing. **Vítor Corrêa Silva:** methodology, software, data curation, visualization. **Adriano Alonso Veloso:** methodology, software, visualization. **Marcelo Rizzatti Luizon:** conceptualization, methodology, visualization, writing – review and editing. **Karina Braga Gomes:** conceptualization, methodology, supervision, visualization, writing – review and editing.

## Ethics Statement

This is an observational study. The COEP‐UFMG Ethics Committee has confirmed that no ethical approval is required.

## Consent

The authors have nothing to report.

## Conflicts of Interest

The authors declare no conflicts of interest.

## Peer Review

The peer review history for this article is available at https://www.webofscience.com/api/gateway/wos/peer‐review/10.1111/jnc.70196.

## Supporting information


**Data S1:** jnc70196‐sup‐0001‐Supinfo.pdf.

## Data Availability

The data that supports the findings of this study are available in the Supporting Information of this article.
