# Supplementary material for: Common miRNAs, Genes, and Regulatory Pathways in Alzheimer's Disease and Type 2 Diabetes Mellitus: An Integrative Analysis of Systematic Reviews, Bioinformatics and Data Mining
Source: J Neurochem. 2025 Aug 18;169(8):e70196. doi: 10.1111/jnc.70196 (PMC12359298; doi:10.1111/jnc.70196)
Supplement: Supplementary file 1 — Data S1: jnc70196‐sup‐0001‐Supinfo.pdf. [file JNC-169-0-s001.pdf]

Supplementary Information (SI)

Review Article

TITLE: Common miRNAs, Genes, and Regulatory Pathways in Alzheimer's Disease and Type 2 Diabetes Mellitus: An Integrative Analysis of Systematic Reviews, Bioinformatics and Data Mining

Author information:

Lívia Cristina Ribeiro Teixeira[1]

Jessica Diniz Pereira[1]

Izabela Mamede[2]

Paulo Caramelli[3]

Vítor Corrêa Silva[4]

Adriano Alonso Veloso[4]

Marcelo Rizzatti Luizon[2]

Karina Braga Gomes[1]

Affiliations:

1- Universidade Federal de Minas Gerais, Faculdade de Farmácia, Belo Horizonte, Minas Gerais, Brazil.

2- Universidade Federal de Minas Gerais, Instituto de Ciências Biológicas, Belo Horizonte, Minas Gerais, Brazil.

3- Universidade Federal de Minas Gerais, Faculdade de Medicina, Belo Horizonte, Minas Gerais, Brazil.

4- Universidade Federal de Minas Gerais, Instituto de Ciências Exatas, Belo Horizonte, Minas Gerais, Brazil.

Corresponding author:

Karina Braga Gomes

Faculty of Pharmacy, Federal University of Minas Gerais.

Antônio Carlos Avenue, 6627, Pampulha, Belo Horizonte, Minas Gerais, Brazil.

Zip Code: 31270-901 / Tel: +55 31 3409-6895 / Fax: 55 31 3409-6985.

E-mail address: karinabgb@ufmg.br

ORCID: https://orcid.org/0000-0002-6870-2063

Supplementary material 1: Search strategy.

- **Alzheimer's disease systematic review**

1. **CENTRAL or COCHRANE**

Adult OR Adults OR Aged OR Elderly AND Alzheimer Disease OR Alzheimer Dementia OR Alzheimer Dementias OR Dementia, Alzheimer OR Alzheimer's Disease OR Dementia, Senile OR Senile Dementia OR Dementia, Alzheimer Type OR Alzheimer Type Dementia OR Alzheimer-Type Dementia (ATD) OR Alzheimer Type Dementia (ATD) OR Dementia, Alzheimer-Type (ATD) OR Alzheimer Type Senile Dementia OR Primary Senile Degenerative Dementia OR Dementia, Primary Senile Degenerative OR Alzheimer Sclerosis OR Sclerosis, Alzheimer OR Alzheimer Syndrome OR Alzheimer's Diseases OR Alzheimer Diseases OR Alzheimers Diseases OR Senile Dementia, Alzheimer Type OR Acute Confusional Senile Dementia OR Senile Dementia, Acute Confusional OR Dementia, Presenile OR Presenile Dementia OR Alzheimer Disease, Late Onset OR Late Onset Alzheimer Disease OR Alzheimer's Disease, Focal Onset OR Focal Onset Alzheimer's Disease OR Familial Alzheimer Disease (FAD) OR Alzheimer Disease, Familial (FAD) OR Familial Alzheimer Diseases (FAD) OR Alzheimer Disease, Early Onset OR Early Onset Alzheimer Disease OR Presenile Alzheimer Dementia in Title Abstract Keyword AND MiRNAs OR MicroRNA OR miRNAs OR Micro RNA OR RNA, Micro OR miRNA OR Primary MicroRNA OR MicroRNA, Primary OR Primary miRNA OR miRNA, Primary OR pri-miRNA OR pri miRNA OR RNA, Small Temporal OR Temporal RNA, Small OR stRNA OR Small Temporal RNA OR pre-miRNA OR pre miRNA

2. **PUBMED/MEDLINE**

(((("Adult"[Mesh]) OR (adult[Title/Abstract] OR adults[Title/Abstract])) OR (("Aged"[Mesh]) OR (aged[Title/Abstract] OR elderly[Title/Abstract]))) AND (("Alzheimer Disease"[Mesh]) OR (Alzheimer Disease[Title/Abstract] OR Alzheimer Dementia[Title/Abstract] OR Alzheimer Dementias[Title/Abstract] OR Dementia, Alzheimer[Title/Abstract] OR Alzheimer's Disease[Title/Abstract] OR Dementia, Senile[Title/Abstract] OR Senile Dementia[Title/Abstract] OR Dementia, Alzheimer Type[Title/Abstract] OR Alzheimer Type Dementia[Title/Abstract] OR Alzheimer-Type Dementia (ATD)[Title/Abstract] OR Alzheimer Type Dementia (ATD)[Title/Abstract] OR Dementia, Alzheimer-Type (ATD)[Title/Abstract] OR Alzheimer Type Senile Dementia[Title/Abstract] OR Primary Senile Degenerative Dementia[Title/Abstract] OR Dementia, Primary Senile Degenerative[Title/Abstract] OR Alzheimer Sclerosis[Title/Abstract] OR Sclerosis, Alzheimer[Title/Abstract] OR Alzheimer Syndrome[Title/Abstract] OR Alzheimer's Diseases[Title/Abstract] OR Alzheimer Diseases[Title/Abstract] OR Alzheimers Diseases[Title/Abstract] OR Senile Dementia, Alzheimer Type[Title/Abstract] OR Acute Confusional Senile Dementia[Title/Abstract] OR Senile Dementia, Acute Confusional[Title/Abstract] OR Dementia, Presenile[Title/Abstract] OR Presenile Dementia[Title/Abstract] OR Alzheimer Disease, Late Onset[Title/Abstract] OR Late Onset Alzheimer Disease[Title/Abstract] OR Alzheimer's Disease, Focal Onset[Title/Abstract] OR Focal Onset Alzheimer's Disease[Title/Abstract] OR Familial Alzheimer Disease (FAD)[Title/Abstract] OR Alzheimer Disease,

Familial (FAD)[Title/Abstract] OR Familial Alzheimer Diseases (FAD)[Title/Abstract] OR Alzheimer Disease, Early Onset[Title/Abstract] OR Early Onset Alzheimer Disease[Title/Abstract] OR Presenile Alzheimer Dementia[Title/Abstract]))) AND (("MiRNAs"[Mesh]) OR (MiRNAs[Title/Abstract] OR MicroRNA[Title/Abstract] OR miRNAs[Title/Abstract] OR Micro RNA[Title/Abstract] OR RNA, Micro[Title/Abstract] OR miRNA[Title/Abstract] OR Primary MicroRNA[Title/Abstract] OR MicroRNA, Primary[Title/Abstract] OR Primary miRNA[Title/Abstract] OR miRNA, Primary[Title/Abstract] OR primiRNA[Title/Abstract] OR pri miRNA[Title/Abstract] OR RNA, Small Temporal[Title/Abstract] OR Temporal RNA, Small[Title/Abstract] OR stRNA[Title/Abstract] OR Small Temporal RNA[Title/Abstract] OR premiRNA[Title/Abstract] OR pre miRNA[Title/Abstract])

3. EMBASE

('adult'/exp OR 'adult' OR 'adults'/exp OR 'adults' OR 'grown-ups'/exp OR 'grown-ups' OR 'grownup'/exp OR 'grownup' OR 'aged'/exp OR 'aged' OR 'aged pacient' OR 'aged people'/exp OR 'aged people' OR (('aged'/exp OR aged) AND people) OR 'aged subject'/exp OR 'aged subject' OR 'elderly'/exp OR 'elderly' OR 'elderly pacient' OR 'elderly people'/exp OR 'elderly people' OR 'elderly person'/exp OR 'elderly person' OR 'elderly subject'/exp OR 'elderly subject' OR 'senior citizen'/exp OR 'senior citizen' OR 'senior') AND ('alzheimer disease' OR 'alzheimer dementia' OR 'alzheimer dementias' OR 'dementia, alzheimer' OR 'alzheimers disease' OR 'dementia, senile' OR 'senile dementia' OR 'dementia, alzheimer type' OR 'alzheimer-type dementia' OR 'alzheimer type dementia' OR 'dementia, alzheimer-type' OR 'alzheimer type senile dementia' OR 'primary senile degenerative dementia' OR 'dementia, primary senile degenerative' OR 'alzheimer sclerosis' OR 'sclerosis, alzheimer' OR 'alzheimer syndrome' OR 'alzheimer diseases' OR 'alzheimers diseases' OR 'senile dementia, alzheimer type' OR 'acute confusional senile dementia' OR 'senile dementia, acute confusional' OR 'dementia, presenile' OR 'presenile dementia' OR 'alzheimer disease, late onset' OR 'late onset alzheimer disease' OR 'alzheimers disease, focal onset' OR 'focal onset alzheimers disease' OR 'familial alzheimer disease' OR 'alzheimer disease, familial' OR 'familial alzheimer diseases' OR 'alzheimer disease, early onset' OR 'early onset alzheimer disease' OR 'presenile alzheimer dementia') AND ('miRNAs' OR 'microrna' OR 'mirnas' OR 'micro rna' OR 'rna, micro' OR 'mirna' OR 'primary microrna' OR 'microrna, primary' OR 'primary mirna' OR 'mirna, primary' OR 'pri-mirna' OR 'pri mirna' OR 'rna, small temporal' OR 'temporal rna, small' OR 'strna' OR 'small temporal rna' OR 'pre-mirna' OR 'pre mirna') AND ([embase]/lim OR [pubmed-not-medline]/lim)

4. CINAHL

(TX Adult OR Adults OR aged OR elderly) AND (TX Alzheimer Disease OR Alzheimer Dementia OR Alzheimer Dementias OR Dementia, Alzheimer OR Alzheimer's Disease OR Dementia, Senile OR Senile Dementia OR Dementia, Alzheimer Type OR Alzheimer Type Dementia OR Alzheimer-Type Dementia (ATD) OR Alzheimer Type Dementia (ATD) OR Dementia, AlzheimerType (ATD) OR Alzheimer Type Senile Dementia OR Primary Senile Degenerative Dementia OR Dementia, Primary Senile Degenerative OR Alzheimer Sclerosis OR Sclerosis, Alzheimer OR Alzheimer Syndrome OR Alzheimer's Diseases OR Alzheimer Diseases OR Alzheimers Diseases OR Senile Dementia, Alzheimer Type OR Acute Confusional Senile Dementia OR Senile Dementia, Acute Confusional OR Dementia, Presenile OR Presenile Dementia

OR Alzheimer Disease, Late Onset OR Late Onset Alzheimer Disease OR Alzheimer's Disease, Focal Onset OR Focal Onset Alzheimer's Disease OR Familial Alzheimer Disease (FAD) OR Alzheimer Disease, Familial (FAD) OR Familial Alzheimer Diseases (FAD) OR Alzheimer Disease, Early Onset OR Early Onset Alzheimer Disease OR Presenile Alzheimer Dementia) AND (TX MiRNAs OR MicroRNA OR miRNAs OR Micro RNA OR RNA, Micro OR miRNA OR Primary MicroRNA OR MicroRNA, Primary OR Primary miRNA OR miRNA, Primary OR pri-miRNA OR pri miRNA OR RNA, Small Temporal OR Temporal RNA, Small OR stRNA OR Small Temporal RNA OR pre-miRNA OR pre miRNA)

5. SCOPUS

TITLE-ABS-KEY (("MiRNAs") OR ("MicroRNA") OR ("miRNAs") OR ("Micro RNA") OR ("RNA, Micro") OR ("miRNA") OR ("Primary MicroRNA") OR ("MicroRNA, Primary") OR ("Primary miRNA") OR ("miRNA, Primary") OR ("pri-miRNA") OR ("pri miRNA") OR ("RNA, Small Temporal") OR ("Temporal RNA, Small") OR ("stRNA") OR ("Small Temporal RNA") OR ("pre-miRNA") OR ("pre miRNA")) TITLE-ABS-KEY (("Alzheimer Disease") OR ("Alzheimer Dementia") OR ("Alzheimer Dementias") OR ("Dementia, Alzheimer") OR ("Alzheimer's Disease") OR ("Dementia, Senile") OR ("Senile Dementia") OR ("Dementia, Alzheimer Type") OR ("Alzheimer Type Dementia") OR ("Alzheimer-Type Dementia ") OR ("Alzheimer Type Dementia") OR ("Dementia, Alzheimer-Type ") OR ("Alzheimer Type Senile Dementia") OR ("Primary Senile Degenerative Dementia") OR ("Dementia, Primary Senile Degenerative") OR ("Alzheimer Sclerosis") OR ("Sclerosis, Alzheimer") OR ("Alzheimer Syndrome") OR ("Alzheimer's Diseases") OR ("Alzheimer Diseases") OR ("Alzheimers Diseases") OR ("Senile Dementia, Alzheimer Type") OR ("Acute Confusional Senile Dementia") OR ("Senile Dementia, Acute Confusional") OR ("Dementia, Presenile") OR ("Presenile Dementia") OR ("Alzheimer Disease, Late Onset") OR ("Late Onset Alzheimer Disease") OR ("Alzheimer's Disease, Focal Onset") OR ("Focal Onset Alzheimer's Disease") OR ("Familial Alzheimer Disease ") OR ("Alzheimer Disease, Familial ") OR ("Familial Alzheimer Diseases ") OR ("Alzheimer Disease, Early Onset") OR ("Early Onset Alzheimer Disease") OR ("Presenile Alzheimer Dementia")) AND TITLE-ABS-KEY (("Adult") OR ("Adults") OR ("Aged") OR ("Elderly"))

6. WEB OF SCIENCE

(Adult) OR (Adults) OR (Aged) OR (Elderly) AND (Alzheimer Disease) OR (Alzheimer Dementia) OR (Alzheimer Dementias) OR (Dementia, Alzheimer) OR (Alzheimer's Disease) OR (Dementia, Senile) OR (Senile Dementia) OR (Dementia, Alzheimer Type) OR (Alzheimer Type Dementia) OR ((Alzheimer-Type Dementia) (ATD)) OR (Alzheimer Type Dementia (ATD)) OR (Dementia, Alzheimer-Type (ATD)) OR (Alzheimer Type Senile Dementia) OR (Primary Senile Degenerative Dementia) OR (Dementia, Primary Senile Degenerative) OR (Alzheimer Sclerosis) OR (Sclerosis, Alzheimer) OR (Alzheimer Syndrome) OR (Alzheimer's Diseases) OR (Alzheimer Diseases) OR (Alzheimers Diseases) OR (Senile Dementia, Alzheimer Type) OR (Acute Confusional Senile Dementia) OR (Senile Dementia, Acute Confusional) OR (Dementia, Presenile) OR (Presenile Dementia) OR (Alzheimer Disease, Late Onset) OR (Late Onset Alzheimer Disease) OR (Alzheimer's Disease, Focal Onset) OR (Focal Onset Alzheimer's Disease) OR (Familial Alzheimer Disease (FAD)) OR Alzheimer Disease, Familial (FAD) OR Familial Alzheimer

Diseases (FAD) OR (Alzheimer Disease, Early Onset) OR (Early Onset Alzheimer Disease) OR Presenile Alzheimer Dementia in Title Abstract Keyword AND MiRNAs OR (MicroRNA) OR (miRNAs) OR (Micro RNA) OR (RNA, Micro) OR (miRNA) OR (Primary MicroRNA) OR (MicroRNA, Primary) OR (Primary miRNA) OR (miRNA, Primary) OR (pri-miRNA) OR (pri miRNA) OR (RNA, Small Temporal) OR (Temporal RNA, Small OR stRNA) OR (Small Temporal RNA) OR (pre-miRNA) OR (pre miRNA in Title Abstract Keyword - (Word variations have been searched)

Manual Search

ID	Title
**(Wu *et al.*, 2016)**	Circulating miRNAs as Biomarkers of Alzheimer's Disease: A Systematic Review
**(Takousis *et al.*, 2019)**	Differential expression of miRNAs in Alzheimer's disease brain, blood and cerebrospinal fluid: a systematic review and meta-analysis
**(Swarbrick *et al.*, 2019)**	Systematic Review of miRNA as Biomarkers in Alzheimer's Disease
**(Hu *et al.*, 2016)**	Diagnostic value of microRNA for Alzheimer's Disease: A Systematic Review and Meta-Analysis

- **Type 2 diabetes mellitus systematic review**

1. **CENTRAL or COCHRANE**

Adult OR Adults OR Aged OR Elderly AND Diabetes Mellitus, Type 2 OR Diabetes Mellitus, Noninsulin-Dependent OR Diabetes Mellitus, Ketosis-Resistant OR Diabetes Mellitus, Ketosis Resistant OR Ketosis-Resistant Diabetes Mellitus OR Diabetes Mellitus, Non Insulin Dependent OR Diabetes Mellitus, Non-Insulin-Dependent OR Non-Insulin-Dependent Diabetes Mellitus OR Diabetes Mellitus, Stable OR Stable Diabetes Mellitus OR Diabetes Mellitus, Type II OR NIDDM OR Diabetes Mellitus, Noninsulin Dependent OR Diabetes Mellitus, Maturity-Onset OR Diabetes Mellitus, Maturity Onset OR Maturity-Onset Diabetes Mellitus OR Maturity Onset Diabetes Mellitus OR MODY OR Diabetes Mellitus, Slow-Onset OR Diabetes Mellitus, Slow Onset OR Slow-Onset Diabetes Mellitus OR Type 2 Diabetes Mellitus OR Noninsulin-Dependent Diabetes Mellitus OR Noninsulin Dependent Diabetes Mellitus OR Maturity-Onset Diabetes OR Diabetes, Maturity-Onset OR Maturity Onset Diabetes OR Type 2 Diabetes OR Diabetes, Type 2 OR Diabetes Mellitus, Adult-Onset OR Adult-Onset Diabetes Mellitus OR Diabetes Mellitus, Adult Onset AND MiRNAs OR MicroRNA OR miRNAs OR Micro RNA OR RNA, Micro OR miRNA OR Primary MicroRNA OR MicroRNA, Primary OR Primary miRNA OR miRNA, Primary OR pri-miRNA OR pri miRNA OR RNA, Small Temporal OR Temporal RNA, Small OR stRNA OR Small Temporal RNA OR pre-miRNA OR pre miRNA

2. **PUBMED/MEDLINE**

(((("Adult"[Mesh]) OR (adult[Title/Abstract] OR adults[Title/Abstract])) OR (("Aged"[Mesh]) OR (aged[Title/Abstract] OR elderly[Title/Abstract]))) AND (("Diabetes Mellitus, Type 2"[Mesh]) OR (Diabetes Mellitus, Type 2[Title/Abstract] OR Diabetes Mellitus, Noninsulin-Dependent[Title/Abstract] OR Diabetes Mellitus, Ketosis-Resistant[Title/Abstract] OR Diabetes Mellitus, Ketosis Resistant[Title/Abstract] OR Ketosis-Resistant Diabetes Mellitus[Title/Abstract] OR Diabetes Mellitus, Non Insulin Dependent[Title/Abstract] OR Diabetes Mellitus, Non-Insulin Dependent[Title/Abstract] OR Non-Insulin-Dependent Diabetes Mellitus[Title/Abstract] OR Diabetes Mellitus, Stable[Title/Abstract] OR Stable Diabetes Mellitus[Title/Abstract] OR Diabetes Mellitus, Type II[Title/Abstract] OR NIDDM[Title/Abstract] OR Diabetes Mellitus, Noninsulin Dependent[Title/Abstract] OR Diabetes Mellitus, Maturity-Onset[Title/Abstract] OR Diabetes Mellitus, Maturity Onset[Title/Abstract] OR Maturity-Onset Diabetes Mellitus[Title/Abstract] OR Maturity Onset Diabetes Mellitus[Title/Abstract] OR MODY[Title/Abstract] OR Diabetes Mellitus, SlowOnset[Title/Abstract] OR Diabetes Mellitus, Slow Onset[Title/Abstract] OR SlowOnset Diabetes Mellitus[Title/Abstract] OR Type 2 Diabetes Mellitus[Title/Abstract] OR Noninsulin-Dependent Diabetes Mellitus[Title/Abstract] OR Noninsulin Dependent Diabetes Mellitus[Title/Abstract] OR Maturity-Onset Diabetes[Title/Abstract] OR Diabetes, Maturity-Onset[Title/Abstract] OR Maturity Onset Diabetes[Title/Abstract] OR Type 2 Diabetes[Title/Abstract] OR Diabetes, Type 2[Title/Abstract] OR Diabetes Mellitus, Adult-Onset[Title/Abstract] OR Adult-Onset Diabetes Mellitus[Title/Abstract] OR Diabetes Mellitus, Adult Onset.[Title/Abstract]))) AND (("MiRNAs"[Mesh]) OR (MiRNAs[Title/Abstract] OR MicroRNA[Title/Abstract] OR miRNAs[Title/Abstract] OR Micro RNA[Title/Abstract] OR RNA,

Micro[Title/Abstract] OR miRNA[Title/Abstract] OR Primary MicroRNA[Title/Abstract] OR MicroRNA, Primary[Title/Abstract] OR Primary miRNA[Title/Abstract] OR miRNA, Primary[Title/Abstract] OR primiRNA[Title/Abstract] OR pri miRNA[Title/Abstract] OR RNA, Small Temporal[Title/Abstract] OR Temporal RNA, Small[Title/Abstract] OR stRNA[Title/Abstract] OR Small Temporal RNA[Title/Abstract] OR premiRNA[Title/Abstract] OR pre miRNA[Title/Abstract]))

3. EMBASE

('adult'/mj OR 'adult':ti,ab OR 'adults':ti,ab OR 'grown-ups':ti,ab OR 'grownup':ti,ab OR 'grownups':ti,ab OR 'aged'/mj OR 'aged':ti,ab OR 'aged patient':ti,ab OR 'aged people':ti,ab OR 'aged person':ti,ab OR 'aged subject':ti,ab OR 'elderly':ti,ab OR 'elderly patient':ti,ab OR 'elderly people':ti,ab OR 'elderly person':ti,ab OR 'elderly subject':ti,ab OR 'senior citizen':ti,ab OR 'senium':ti,ab) AND ('non insulin dependent diabetes mellitus'/mj OR 'niddm (non insulin dependent diabetes mellitus)':ti,ab OR 't2dm':ti,ab OR 'adult onset diabetes':ti,ab OR 'adult onset diabetes mellitus':ti,ab OR 'diabetes mellitus type 2':ti,ab OR 'diabetes mellitus type ii':ti,ab OR 'diabetes mellitus, maturity onset':ti,ab OR 'diabetes mellitus, non insulin dependent':ti,ab OR 'diabetes mellitus, non-insulin-dependent':ti,ab OR 'diabetes mellitus, type 2':ti,ab OR 'diabetes mellitus, type ii':ti,ab OR 'diabetes type 2':ti,ab OR 'diabetes type ii':ti,ab OR 'diabetes, adult onset':ti,ab OR 'dm 2':ti,ab OR 'insulin independent diabetes':ti,ab OR 'insulin independent diabetes mellitus':ti,ab OR 'ketosis resistant diabetes mellitus':ti,ab OR 'maturity onset diabetes':ti,ab OR 'maturity onset diabetes mellitus':ti,ab OR 'maturity onset diabetes of the young':ti,ab OR 'niddm':ti,ab OR 'non insulin dependent diabetes':ti,ab OR 'non insulin dependent diabetes mellitus':ti,ab OR 'non-insulin-dependent diabetes mellitus':ti,ab OR 'noninsulin dependent diabetes':ti,ab OR 'noninsulin dependent diabetes mellitus':ti,ab OR 'type 2 diabetes':ti,ab OR 'type 2 diabetes mellitus':ti,ab OR 'type ii diabetes':ti,ab OR 'type ii diabetes mellitus':ti,ab) AND ('microrna'/mj OR 'mirna':ti,ab OR 'mirnas':ti,ab OR 'micro rna':ti,ab OR 'microrna':ti,ab OR 'miRNAs':ti,ab) AND ([embase]/lim OR [pubmed-not-medline]/lim)

4. CINAHL

(TX Adult OR Adults OR aged OR elderly) AND (TX Diabetes Mellitus, Type 2 OR Diabetes Mellitus, Noninsulin-Dependent OR Diabetes Mellitus, Ketosis-Resistant OR Diabetes Mellitus, Ketosis Resistant OR Ketosis-Resistant Diabetes Mellitus OR Diabetes Mellitus, Non Insulin Dependent OR Diabetes Mellitus, Non-Insulin-Dependent OR Non-Insulin-Dependent Diabetes Mellitus OR Diabetes Mellitus, Stable OR Stable Diabetes Mellitus OR Diabetes Mellitus, Type II OR NIDDM OR Diabetes Mellitus, Noninsulin Dependent OR Diabetes Mellitus, Maturity-Onset OR Diabetes Mellitus, Maturity Onset OR Maturity-Onset Diabetes Mellitus OR Maturity Onset Diabetes Mellitus OR MODY OR Diabetes Mellitus, Slow-Onset OR Diabetes Mellitus, Slow Onset OR Slow-Onset Diabetes Mellitus OR Type 2 Diabetes Mellitus OR Noninsulin-Dependent Diabetes Mellitus OR Noninsulin Dependent Diabetes Mellitus OR Maturity-Onset Diabetes OR Diabetes, Maturity-Onset OR Maturity Onset Diabetes OR Type 2 Diabetes OR Diabetes, Type 2 OR Diabetes Mellitus, Adult-Onset OR Adult-Onset Diabetes Mellitus OR Diabetes Mellitus, Adult Onset) AND (TX MiRNAs OR MicroRNA OR miRNAs OR Micro RNA OR RNA, Micro OR miRNA OR Primary MicroRNA OR MicroRNA, Primary OR Primary miRNA OR miRNA, Primary

OR pri-miRNA OR pri miRNA OR RNA, Small Temporal OR Temporal RNA, Small OR stRNA OR Small Temporal RNA OR pre-miRNA OR pre miRNA)

5. SCOPUS

TITLE-ABS-KEY (("MiRNAs") OR ("MicroRNA") OR ("miRNAs") OR ("Micro RNA") OR ("RNA, Micro") OR ("miRNA") OR ("Primary MicroRNA") OR ("MicroRNA, Primary") OR ("Primary miRNA") OR ("miRNA, Primary") OR ("pri-miRNA") OR ("pri miRNA") OR ("RNA, Small Temporal") OR ("Temporal RNA, Small") OR ("stRNA") OR ("Small Temporal RNA") OR ("pre-miRNA") OR ("pre miRNA")) TITLE-ABS-KEY (("Diabetes Mellitus, Type 2") OR ("Diabetes Mellitus, Noninsulin-Dependent") OR (" Diabetes Mellitus, Ketosis-Resistant") OR ("Diabetes Mellitus, Ketosis Resistant") OR (" Ketosis-Resistant Diabetes Mellitus") OR ("Diabetes Mellitus, Non Insulin Dependent") OR ("Diabetes Mellitus, Non-Insulin-Dependent") OR (" Non-Insulin-Dependent Diabetes Mellitus") OR (" Diabetes Mellitus, Stable ") OR ("Stable Diabetes Mellitus") OR ("Diabetes Mellitus, Type ii") OR ("Niddm") OR ("Diabetes Mellitus, Noninsulin Dependent") OR ("Diabetes Mellitus, Maturity-Onset") OR ("Diabetes Mellitus, Maturity Onset") OR (" Maturity-Onset Diabetes Mellitus") OR ("Maturity Onset Diabetes Mellitus") OR ("Mody") OR ("Diabetes Mellitus, Slow-Onset") OR (" Diabetes Mellitus, Slow Onset ") OR ("Slow-Onset Diabetes Mellitus") OR ("Type 2 Diabetes Mellitus") OR ("Noninsulin-Dependent Diabetes Mellitus") OR (" Noninsulin Dependent Diabetes Mellitus") OR (" Maturity-Onset Diabetes") OR ("Diabetes, Maturity-Onset") OR ("Maturity Onset Diabetes") OR ("Type 2 Diabetes") OR ("Diabetes, Type 2") OR ("Diabetes Mellitus, Adult-Onset") OR ("Adult-Onset Diabetes Mellitus") OR ("Diabetes Mellitus, Adult Onset")) AND TITLE-ABS-KEY (("Adult") OR ("Adults") OR ("Aged") OR ("Elderly"))

6. WEB OF SCIENCE

(Adult) OR (Adults) OR (Aged) OR (Elderly) AND (Diabetes Mellitus, Type 2) OR (Diabetes Mellitus, Noninsulin-Dependent) OR (Diabetes Mellitus, Ketosis-Resistant) OR (Diabetes Mellitus, Ketosis Resistant) OR (Ketosis-Resistant Diabetes Mellitus) OR (Diabetes Mellitus, Non Insulin Dependent) OR (Diabetes Mellitus, Non-Insulin-Dependent) OR (Non-Insulin-Dependent Diabetes Mellitus) OR (Diabetes Mellitus, Stable) OR (Stable Diabetes Mellitus) OR (Diabetes Mellitus, Type II) OR (NIDDM) OR (Diabetes Mellitus, Noninsulin Dependent) OR (Diabetes Mellitus, Maturity-Onset) OR (Diabetes Mellitus, Maturity Onset) OR (Maturity-Onset Diabetes Mellitus) OR (Maturity Onset Diabetes Mellitus) OR (MODY) OR (Diabetes Mellitus, Slow-Onset) OR (Diabetes Mellitus, Slow Onset) OR (Slow-Onset Diabetes Mellitus) OR (Type 2 Diabetes Mellitus) OR (Noninsulin-Dependent Diabetes Mellitus) OR (Noninsulin Dependent Diabetes Mellitus) OR (Maturity-Onset Diabetes) OR (Diabetes, Maturity-Onset) OR (Maturity Onset Diabetes) OR (Type 2 Diabetes) OR (Diabetes, Type 2) OR (Diabetes Mellitus, Adult-Onset) OR (Adult-Onset Diabetes Mellitus) OR (Diabetes Mellitus, Adult Onset) AND (MiRNAs) OR (MicroRNA) OR (miRNAs) OR (Micro RNA) OR (RNA, Micro) OR (miRNA) OR (Primary MicroRNA) OR (MicroRNA, Primary) OR (Primary miRNA) OR (miRNA, Primary) OR (pri-miRNA) OR (pri miRNA) OR (RNA, Small Temporal) OR (Temporal RNA, Small) OR (stRNA) OR (Small Temporal RNA) OR (pre-miRNA) OR (pre miRNA)

Manual Search

ID	Title
**(Gonzalez-Sanchez *et al.*, 2021)**	miRNAs as biomarkers for diagnosis of type 2 diabetes: A systematic review
**(He *et al.*, 2017)**	A Systematic Study of Dysregulated MicroRNA in Type 2 Diabetes Mellitus
**(Liang *et al.*, 2020)**	Identification of stress-related microRNA biomarkers in type 2 diabetes: a systematic review and meta-analysis
**(Zhu & Leung, 2015)**	Identification of microRNA biomarkers in type 2 diabetes: a meta-analysis of controlled profiling studies

Supplementary material 2: Flowchart of the literature search and the study selection process of Alzheimer's disease systematic review. *According to The PRISMA 2020 statement: an updated guideline for reporting systematic reviews (Page et al., 2021).*

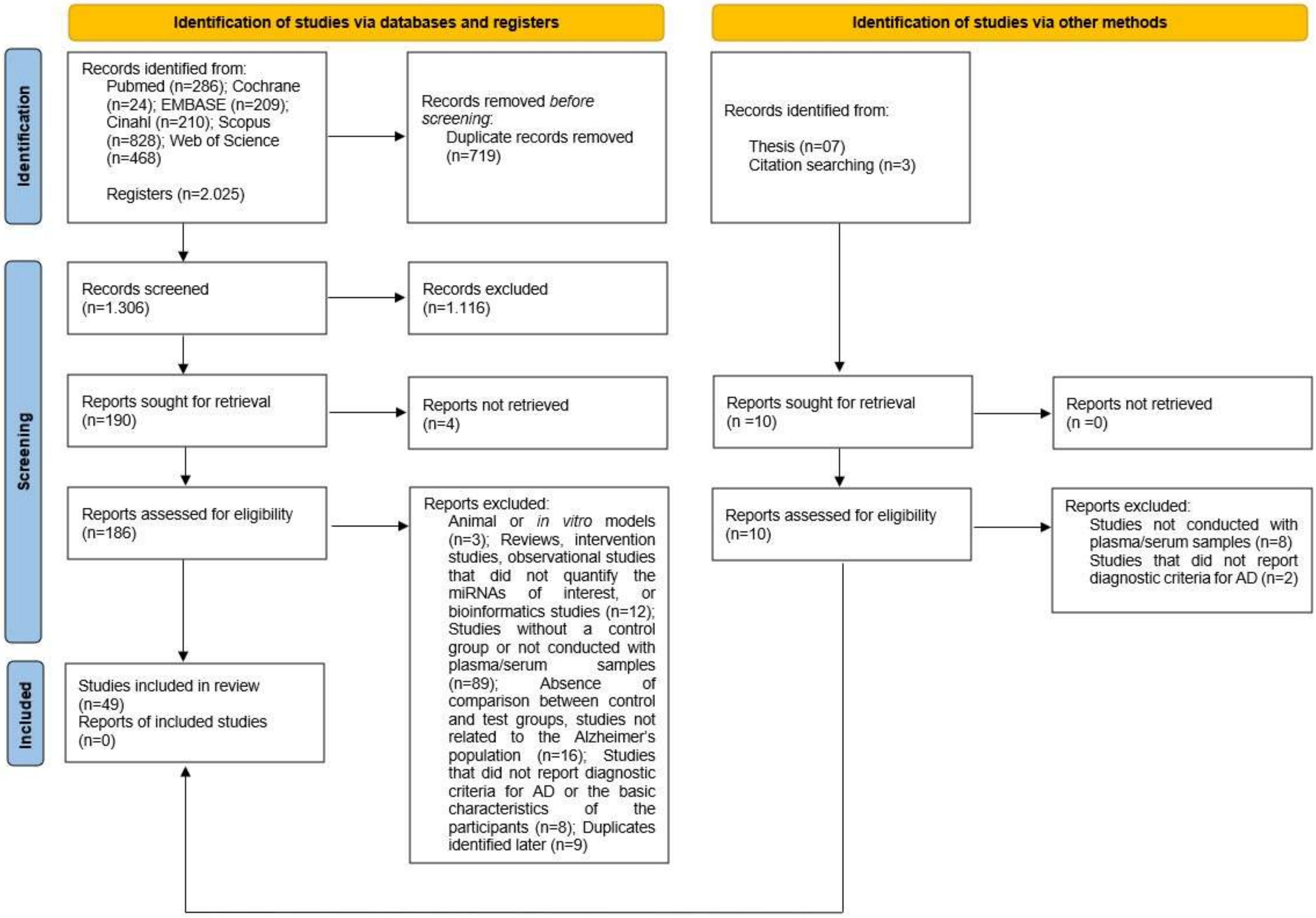

Supplementary material 3: Flowchart of the literature search and the study selection process of type 2 diabetes mellitus systematic review. *According to The PRISMA 2020 statement: an updated guideline for reporting systematic reviews (Page et al., 2021).*

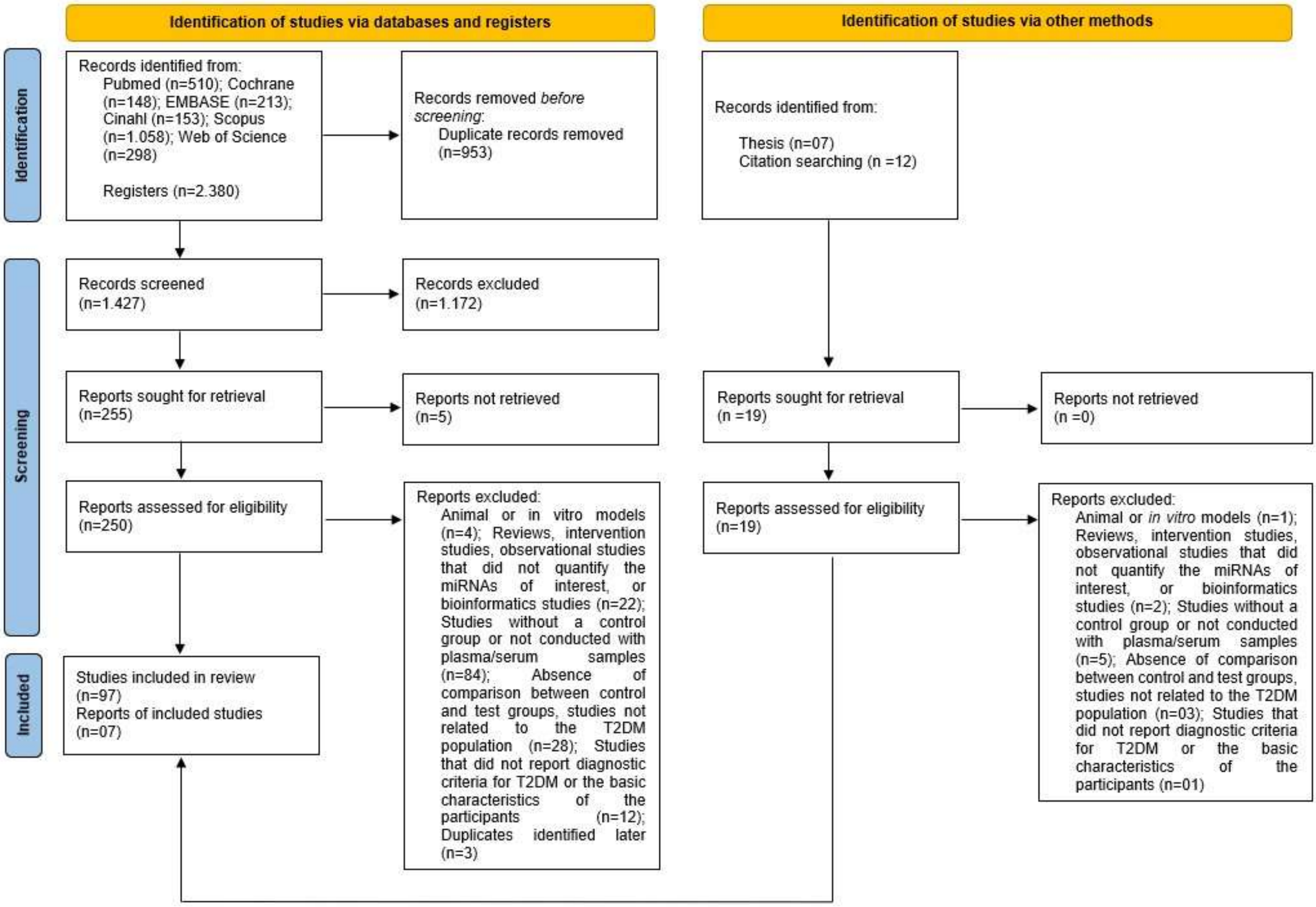

Supplementary material 4: JBI Critical Appraisal Checklist for Case Control Studies and quality rating criteria

	**JBI Critical Appraisal Checklist for Case Control Studies**	**YES**	**NO**
**1**	Were the groups comparable other than the presence of disease in cases or the absence of disease in controls?	+1	0
**2**	Were cases and controls matched appropriately?	+1	0
**3**	Were the same criteria used for identification of cases and controls?	+1	0
**4**	Was exposure measured in a standard, valid and reliable way?	+1	0
**5**	Was exposure measured in the same way for cases and controls?	+1	0
**6**	Were confounding factors identified?	-1	+2
**7**	Were strategies to deal with confounding factors stated?	If not the previous answer: 0<br>If yes to previous answer: +2	If not the previous answer: 0<br>If yes to previous answer: +2
**8**	Were outcomes assessed in a standard, valid and reliable way for cases and controls?	+1	0
**9**	Was the exposure period of interest long enough to be meaningful?	+1	0
**10**	Was appropriate statistical analysis used?	+1	0

Supplementary material 5: Data from articles included in Alzheimer's disease systematic review.

**Author/Year**	**AD Diagnostic criteria**	**Sample size**		**Country of origin**	**Age**		**Sex M=male / F=female**		**Statistically significant difference between**	
		**Control**	**Case**		**Control**	**Case**	**Control**	**Case**	**Age**	**Sex**
(Abolghasemi *et al.*, 2021)	DSM-V	50	50	Iran	71.11 ± 2.61	69.73 ± 2.18	25M / 25F	22M / 28F	No	No
(Barbagallo *et al.*, 2020)	NINCDS-ADRDA	30	30	Italy	67.9 ± 8.2	72.6 ± 8.1	10M / 20F	14M / 16F	Yes	NI
(Bhatnagar *et al.*, 2014)	NINCDS-ADRDA	85	78	Canada	76-90	76-90	NI	NI	No	NI
(Burgos *et al.*, 2014)	NIA-R	78	69	USA	82.1 + 10	81.3 + 7.7	43M / 35F	33M / 36F	No	No
(Cao *et al.*, 2020)	NINCDS–ADRDA	93	108	China	71.10 ± 4.88	71.50 ± 4.76	52M / 41F	59M / 49F	No	No
(Cosin-Tomas *et al.*, 2017)	IWG and NIA-AA	Cohort 1: 21<br>Cohort 2: 15	Cohort 1: 21<br>Cohort 2: 15	Spain	Cohort 1: 69.55 (67.19 – 71.91)<br>Cohort 2: 62.57 (60.48–64.67)	Cohort 1: 71.83 (69.55–74.11)<br>Cohort 2: 62.99 (60.75–65.24)	Cohort 1: 9M / 12F<br>Cohort 2: 7M / 8F	Cohort 1: 9M / 12F<br>Cohort 2: 6M / 9F	No	No
(Denk *et al.*, 2018)	NINCDS–ADRDA	Total: 44 Serum:38	Total: 48 Serum:47	Germany	64 ± 11.3	65 ± 9.3	20M / 24F	30M / 18F	No	No

(Dong *et al.*, 2015)	NINDS-AA	Total:123<br>Serum: 75	Total:127<br>Serum: 79	China	79.5 ± 6.8	79.3 ± 8.9	65M / 58F	72M / 55F	No	No
(Dong *et al.*, 2021)	National Institute of Neurology and the Institute of Alzheimer's Diseases	86	Mild AD: 31<br>Moderate AD: 52<br>Severe AD: 38	China	69.0 ± 5.8	Mild AD: 70.5 ± 6.2<br>Moderate AD: 69.6 ± 7.3<br>Severe AD: 71.7 ± 6.4	45M / 41F	Mild AD: 16M / 15F<br>Moderate AD: 28M / 24F<br>Severe AD: 17M / 21F	No	No
(Nagaraj *et al.*, 2017)	DSM-IV, NINCDS-ADRDA and NIA-AA	Study 1: 6<br><br>Study 2: 9	Study 1: 7<br><br>Study 2: 13	Poland	Study 1: 66 +/-5<br><br>Study 2: 66 +/- 3	Study 1: 73.7 +/-5<br><br>Study 2: 67.5 +/- 8	Study 1: 2M / 4F<br><br>Study 2: 5M / 4F	Study 1: 4M / 3F<br><br>Study 2: 6M / 7F	No	No
(Galimberti *et al.*, 2014)	NINCDS-ADRDA	18	22	Italy	68.82 ± 0.71	72.77 ± 0.65	13M / 5F	8M / 14F	No	No
(Geekiyanage *et al.*, 2012)	NIA	7	7	USA	86.85 (82-91)	86.14 (80-96)	4M / 3F	2M / 5F	NI	NI
(Giuliani *et al.*, 2021)	DMS IV-R and NINCDS–ADRDA	40	116	Italy	75.1 ± 7.5 (65–92)	77.2 ± 5.4 (65–89)	15M / 25F	36M / 80F	No	No

(Guo *et al.*, 2017)	NINCDS–ADRDA	86	Mild AD:31 Moderate AD:52 Severe AD:38	China	67.95 (5.76)	Mild AD:71.43 (6.21) Moderate AD:72.52 (7.45) Severe AD:74.12(5.25)	45M / 41F	Mild AD:16M / 15F Moderate AD:28M / 24F Severe AD:17M / 21F	No	No
(Guven *et al.*, 2021)	NINCDS–ADRDA	12	26	Turkey	73.7 ± 5.3	76.5 ± 4.4	5M / 7F	9M / 17F	No	No
(Hajjri *et al.*, 2020)	NINCDS–ADRDA	15	20	Iran	64.86 ± 4.67 (58-71)	64.45 ± 3.48 (58-73)	Only men	Only men	No	No
(Han *et al.*, 2020)	DSM-IV and NINCDS-ADRDA	30	33	China	65.35 ± 6.93 (55–86)	69.87 ± 7.85 (58–88)	NI	NI	NI	NI
(Hara *et al.*, 2017)	NINCDS–ADRDA	22	36	Japan	73.7 ± 8.4	74.7 ± 7.3	4M / 18F	13M / 23F	No	No
(Heydari *et al.*, 2021)	NINCDS-ADRDA	23	21	Iran	65.21% of 50-65 years 30.43% of 65-80 years 4.34% of 80-95 years	28.57% of 50-65 years 47.61% of 65-80 years 23.80% of 80-95 years	13M / 10F	6M / 15F	NI	NI
(Jia & Liu, 2016)	NINCDS-ADRDA	62	84	China	76.23 ± 19.1	81.36 ± 13.25	41M / 21F	62M / 22F	No	No
(Kiko *et al.*, 2014)	NINCDS-ADRDA	10	10	Japan	73.0 ± 5.2	80.7 ± 5.8	4M / 6F	3M / 7F	NI	NI

(Kim *et al.*, 2021)	NINCDS-ADRDA	31	20	Korea	72.85 ± 5.31	72.54 ± 3.61	18M / 13F	8M / 12F	No	NI
(Kumar *et al.*, 2017)	NINCDS-ADRDA	4	1	USA	65-88	86	4F	1M	NI	Yes
(Li *et al.*, 2019)	NINCDS-ADRDA	65	51	China	70.2+2.8	69.2+3.5	30M / 35F	15M / 36F	No	No
(Liu & Lei, 2021)	NINCDS-ADRDA	60	110	China	72.52 ± 7.57	72.69 ± 6.64	23M / 37F	51M / 59F	No	No
(Maffioletti *et al.*, 2019)	NINCDS-ADRDA	45	49	Italy	66.33 ± 9.61	77.67 ± 5.91	21M / 24F	15M / 34F	Yes	No
(Mancuso *et al.*, 2019)	NINCDS-ADRDA	40	40	Italy	75.00 (72.75–78.00)	79.00 (73.25–80.75)	16M / 24F	18M / 22F	No	No
(Poursaei *et al.*, 2022)	DSM-IV	50	50	Iran	77.32 ± 30.11	75.32 ± 28.6 5	25M / 25F	22M / 28F	No	No
(Prendecki *et al.*, 2019)	NINCDS-ADRDA	74	64	Poland	71.4	76.0	16M / 58F	20M / 44F	NI	NI
(Ragusa *et al.*, 2016)	NINCDS-ADRDA	40	40	Italy	81.9 ± 6.18	81.375 ± 4.68	19M / 21F	17M / 23F	No	No
(Sabry *et al.*, 2021)	DSM-V	20	20	Egypt	62.60 ± 7.98 (50 – 85)	66.70 ± 6.10 (60 – 85)	14M / 6F	8M / 12F	NI	NI
(Tan *et al.*, 2014a)	NINCDS–ADRDA	155	105	China	72.65 ± 4.51	71.64 ± 5.28	75M / 80F	57M / 48F	No	No
(Tan *et al.*, 2014b)	NINCDS–ADRDA	155	158	China	76.86 ± 7.00	77.43 ± 7.40	70M / 85F	78M / 80F	No	No

(Wang *et al.*, 2020)	NINCDS–ADRDA	120	120	China	71.2 ± 10.8	72.5 ± 7.7	54M / 66F	55M / 65F	No	No
(Wang & Zhang, 2020)	NINCDS–ADRDA	62	118	China	72.53 ± 7.60	72.92 ± 6.87	28M / 34F	55M / 63F	No	No
(Wu *et al.*, 2017)	NINCDS-ADRDA	60	65	China	73.0 ± 12.7	72.1 ± 8.5	30M / 20F	32M / 23F	No	No
(Zeng *et al.*, 2017)	NIA-AA	30	Mild AD: 30 Moderate AD: 30	China	62.1 ± 10.6	Mild AD: 63.3 ± 11.5 Moderate AD: 64.5 ± 9.7	13M / 17F	Mild AD: 11M / 19F Moderate AD: 12M / 18F	No	No
(Zhang *et al.*, 2021a)	NINCDS-ADRDA	100	110	China	71.64 ± 4.00	70.89 ± 4.09	46M / 54F	47M / 63F	No	No
(Zhang *et al.*, 2021b)	NINCDS-ADRDA	106	117	China	73.15 ± 9.29	72.57 ± 8.13	61M / 45F	67M / 50F	No	No
(Zhao *et al.*, 2020)	NINCDS-ADRDA	12	19	United Kingdom	78 ± 7 (65–90)	74 ± 9 (57–82)	5M / 7F	7M / 12F	No	No
(Zhu *et al.*, 2015)	NINCDS-ADRDA	42	26	China	71.9 (62-85)	72.3 (60-84)	23M / 19F	12M / 14F	NI	NI
(Zirnheld *et al.*, 2016)	NINCDS-ADRDA	37	Mild AD: 16 Moderate / Severe AD: 20	Canada	75.3 ± 5.4 (67-92)	Mild AD: 78.2 ± 3.5 (73-84) Moderate / Severe AD: 77.7 ± 6.8 (63-88)	6M / 31F	Mild AD: 9M / 7F Moderate / Severe AD: 6M / 14F	NI	NI
(Abed *et al.*, 2023)	DSM-V	40	40	Iraq	75.03 ± 6.59 (62-88)	74.98 ± 5.03 (67-85)	21M / 19F	17M / 23F	No	No

(Abuelezz *et al.*, 2022)	NINCDS-ADRDA	23	23	Egypt	71.26 ± 3.4	73.87 ± 5.7	10M / 13F	7M / 16F	No	No
(Biglari *et al.*, 2022)	NINCDS-ADRDA	50	50	Iran	77.04 ± 6.03	76.65 ± 6.26	20M / 30F	15M / 35F	NI	NI
(Piscopo *et al.*, 2023)	DSM-IV e NINCDS-ADRDA	43	33	Italy	72.74 ± 7.40	69.15 ± 10.46	18M / 25F	14M / 19F	NI	NI
(Wang *et al.*, 2023)	NIA-AA	68	92	China	69.50 ± 6.55	70.12 ± 8.56	37M / 31F	49M / 43F	No	No
(Zhang *et al.*, 2022)	NINCDS-ADRDA	93	98	China	66.19 ± 6.54	66.83 ± 6.39	44M / 49F	57M / 41F	No	No
(Dragović *et al.*, 2022)	NIA-AA	18	12	Montenegro	65.44 ± 8.12	70.92 ± 7.34	11M / 7F	6M / 6F	No	No

DSM-IV: Diagnostic and Statistical Manual of Mental Disorders, 4th Edition / DSM-V: Diagnostic and Statistical Manual of Mental Disorders, 5th Edition / IWG: International Working Group / NIA: National Institute on Aging / NIA-AA: National Institute on Aging - Alzheimer's Association / NIA-R: National Institute on Aging - Revised / NINCDS-ADRA: National Institute of Neurological and Communicative Disorders and Stroke - Alzheimer's Disease and Related Disorders Association / NINDS-AA: National Institute of Neurological Disorders and Stroke - Alzheimer's Association / NI: not informed.

Supplementary material 6: Risk of bias assessment of studies included in the Alzheimer´s disease systematic review.

Study identification	JBI Critical Appraisal Checklist for Case Control Studies										
	**1**	**2**	**3**	**4**	**5**	**6**	**7**	**8**	**9**	**10**	**Score**
(Abolghasemi *et al.*, 2021)	Y	Y	N	Y	Y	N	NA	Y	Y	Y	9
(Barbagallo *et al.*, 2020)	Y	N	N	Y	Y	Y	Y	Y	Y	Y	7
(Bhatnagar *et al.*, 2014)	Y	U	Y	Y	Y	U	U	Y	Y	Y	5
(Burgos *et al.*, 2014)	Y	Y	N	Y	Y	N	NA	Y	Y	Y	9
(Cao *et al.*, 2020)	Y	Y	Y	Y	Y	N	NA	Y	Y	Y	10
(Cosin-Tomas *et al.*, 2017)	Y	Y	Y	Y	Y	N	NA	Y	Y	Y	10
(Denk *et al.*, 2018)	Y	Y	N	Y	Y	N	NA	Y	Y	Y	9
(Dong *et al.*, 2015)	Y	Y	Y	Y	Y	N	NA	Y	Y	Y	10
(Dong *et al.*, 2021)	Y	Y	Y	Y	Y	N	NA	Y	Y	Y	10
(Nagaraj *et al.*, 2017)	Y	Y	N	Y	Y	N	NA	Y	Y	Y	9
(Galimberti *et al.*, 2014)	Y	N	Y	Y	Y	Y	Y	Y	Y	Y	8

(Geekiyanage *et al.*, 2012)	Y	N	Y	Y	Y	Y	Y	Y	Y	Y	8
(Giuliani *et al.*, 2021)	Y	Y	Y	Y	Y	N	NA	Y	Y	Y	10
(Guo *et al.*, 2017)	Y	Y	Y	Y	Y	N	NA	Y	Y	Y	10
(Guven *et al.*, 2021)	Y	Y	Y	Y	Y	N	NA	Y	Y	Y	10
(Hajjri *et al.*, 2020)	U	Y	Y	Y	Y	N	NA	Y	Y	Y	9
(Han *et al.*, 2020)	U	N	N	Y	Y	Y	N	Y	Y	Y	3
(Hara *et al.*, 2017)	Y	Y	Y	Y	Y	N	NA	Y	Y	Y	10
(Heydari *et al.*, 2021)	Y	N	N	Y	Y	Y	N	Y	Y	Y	4
(Jia & Liu, 2016)	Y	Y	N	Y	Y	N	NA	Y	Y	Y	9
(Kiko *et al.*, 2014)	Y	Y	Y	Y	Y	N	NA	Y	Y	Y	10
(Kim *et al.*, 2021)	Y	Y	Y	Y	Y	N	NA	Y	Y	Y	10
(Kumar *et al.*, 2017)	Y	Y	Y	Y	Y	N	NA	Y	Y	Y	10
(Li *et al.*, 2019)	Y	Y	Y	Y	Y	N	NA	Y	Y	Y	10
(Liu & Lei, 2021)	Y	Y	Y	Y	Y	N	NA	Y	Y	Y	10

(Maffioletti *et al.*, 2019)	Y	N	Y	Y	Y	Y	Y	Y	Y	Y	8
(Mancuso *et al.*, 2019)	Y	Y	Y	Y	Y	N	NA	Y	Y	Y	10
(Poursaei *et al.*, 2022)	Y	Y	N	Y	Y	N	NA	Y	Y	Y	9
(Prendecki *et al.*, 2019)	Y	Y	N	Y	Y	N	NA	Y	Y	Y	9
(Ragusa *et al.*, 2016)	Y	Y	N	Y	Y	N	NA	Y	Y	Y	9
(Sabry *et al.*, 2021)	Y	Y	Y	Y	Y	N	NA	Y	Y	Y	10
(Tan *et al.*, 2014a)	Y	Y	Y	Y	Y	N	NA	Y	Y	Y	10
(Tan *et al.*, 2014b)	Y	Y	Y	Y	Y	N	NA	Y	Y	Y	10
(Wang *et al.*, 2020)	Y	Y	Y	Y	Y	N	NA	Y	Y	Y	10
(Wang & Zhang, 2020)	Y	Y	Y	Y	Y	N	NA	Y	Y	Y	10
(Wu *et al.*, 2017)	Y	Y	Y	Y	Y	N	NA	Y	Y	Y	10
(Zeng *et al.*, 2017)	Y	Y	Y	Y	Y	N	NA	Y	Y	Y	10
(Zhang *et al.*, 2021a)	Y	Y	Y	Y	Y	N	NA	Y	Y	Y	10
(Zhang *et al.*, 2021b)	Y	Y	Y	Y	Y	N	NA	Y	Y	Y	10

(Zhao *et al.*, 2020)	Y	Y	N	Y	Y	N	NA	Y	Y	Y	9
(Zhu *et al.*, 2015)	Y	Y	N	Y	Y	N	NA	Y	Y	Y	9
(Zirnheld *et al.*, 2016)	Y	Y	Y	Y	Y	N	NA	Y	Y	Y	10
(Abed *et al.*, 2023)	Y	Y	Y	Y	Y	N	NA	Y	Y	Y	10
(Abuelezz *et al.*, 2022)	Y	Y	Y	Y	Y	N	NA	Y	Y	Y	10
(Biglari *et al.*, 2022)	Y	Y	Y	Y	Y	N	NA	Y	Y	Y	10
(Piscopo *et al.*, 2023)	Y	Y	N	Y	Y	N	NA	Y	Y	Y	9
(Wang *et al.*, 2023)	Y	Y	N	Y	Y	N	NA	Y	Y	Y	9
(Zhang *et al.*, 2022)	Y	Y	Y	Y	Y	N	NA	Y	Y	Y	10
(Dragović *et al.*, 2022)	Y	Y	Y	Y	Y	N	NA	Y	Y	Y	10

Supplementary material 7: Risk of bias heatmap based on the JBI Critical Appraisal Checklist for Case Control Studies applied to the studies included in the Alzheimer's disease systematic review.

JBI Critical Appraisal Checklist for Case Control Studies - Alzheimer´s disease systematic review

Y Yes; N No; U Unclear; NA Not applicable

Study ID	D1	D2	D3	D4	D5	D6	D7	D8	D9	D10
(Abolghasemi *et al.*, 2021)	Y	Y	N	Y	Y	N	NA	Y	Y	Y
(Barbagallo *et al.*, 2020)	Y	N	N	Y	Y	Y	Y	Y	Y	Y
(Bhatnagar *et al.*, 2014)	Y	U	Y	Y	Y	U	U	Y	Y	Y
(Burgos *et al.*, 2014)	Y	Y	N	Y	Y	N	NA	Y	Y	Y
(Cao *et al.*, 2020)	Y	Y	Y	Y	Y	N	NA	Y	Y	Y
(Cosin-Tomas *et al.*, 2017)	Y	Y	Y	Y	Y	N	NA	Y	Y	Y
(Denk *et al.*, 2018)	Y	Y	N	Y	Y	N	NA	Y	Y	Y
(Dong *et al.*, 2015)	Y	Y	Y	Y	Y	N	NA	Y	Y	Y
(Dong *et al.*, 2021)	Y	Y	Y	Y	Y	N	NA	Y	Y	Y
(Nagaraj *et al.*, 2017)	Y	Y	N	Y	Y	N	NA	Y	Y	Y
(Galimberti *et al.*, 2014)	Y	N	Y	Y	Y	Y	Y	Y	Y	Y
(Geekiyanage *et al.*, 2012)	Y	N	Y	Y	Y	Y	Y	Y	Y	Y
(Giuliani *et al.* 2021)	Y	Y	Y	Y	Y	N	NA	Y	Y	Y
(Guo *et al.*, 2017)	Y	Y	Y	Y	Y	N	NA	Y	Y	Y
(Guven *et al.*, 2021)	Y	Y	Y	Y	Y	N	NA	Y	Y	Y
(Hajjri *et al.*, 2020)	U	Y	Y	Y	Y	N	NA	Y	Y	Y
(Han *et al.*, 2020)	U	N	N	Y	Y	Y	N	Y	Y	Y
(Hara *et al.*, 2017)	Y	Y	Y	Y	Y	N	NA	Y	Y	Y
(Heydari *et al.*, 2021)	Y	N	N	Y	Y	Y	N	Y	Y	Y
(Jia & Liu, 2016)	Y	Y	N	Y	Y	N	NA	Y	Y	Y
(Kiko *et al.*, 2014)	Y	Y	Y	Y	Y	N	NA	Y	Y	Y
(Kim *et al.* 2021)	Y	Y	Y	Y	Y	N	NA	Y	Y	Y
(Kumar *et al.*, 2017)	Y	Y	Y	Y	Y	N	NA	Y	Y	Y
(Li *et al.*, 2019)	Y	Y	Y	Y	Y	N	NA	Y	Y	Y
(Liu & Lei, 2021)	Y	Y	Y	Y	Y	N	NA	Y	Y	Y

Study ID	D1	D2	D3	D4	D5	D6	D7	D8	D9	D10
(Maffioletti *et al.*, 2019)	Y	N	Y	Y	Y	Y	Y	Y	Y	Y
(Mancuso *et al.*, 2019)	Y	Y	Y	Y	Y	N	NA	Y	Y	Y
(Poursaei *et al.*, 2022)	Y	Y	N	Y	Y	N	NA	Y	Y	Y
(Prendecki *et al.*, 2019)	Y	Y	N	Y	Y	N	NA	Y	Y	Y
(Ragusa *et al.* 2016)	Y	Y	N	Y	Y	N	NA	Y	Y	Y
(Sabry *et al.*, 2021)	Y	Y	Y	Y	Y	N	NA	Y	Y	Y
(Tan *et al.*, 2014a)	Y	Y	Y	Y	Y	N	NA	Y	Y	Y
(Tan *et al.*, 2014b)	Y	Y	Y	Y	Y	N	NA	Y	Y	Y
(Wang *et al.*, 2020)	Y	Y	Y	Y	Y	N	NA	Y	Y	Y
(Wang & Zhang, 2020)	Y	Y	Y	Y	Y	N	NA	Y	Y	Y
(Wu *et al.*, 2017)	Y	Y	Y	Y	Y	N	NA	Y	Y	Y
(Zeng *et al.*, 2017)	Y	Y	Y	Y	Y	N	NA	Y	Y	Y
(Zhang *et al.* 2021a)	Y	Y	Y	Y	Y	N	NA	Y	Y	Y
(Zhang *et al.*, 2021b)	Y	Y	Y	Y	Y	N	NA	Y	Y	Y
(Zhao *et al.*, 2020)	Y	Y	N	Y	Y	N	NA	Y	Y	Y
(Zhu *et al.*, 2015)	Y	Y	N	Y	Y	N	NA	Y	Y	Y
(Zirnheld *et al.*, 2016)	Y	Y	Y	Y	Y	N	NA	Y	Y	Y
(Abed *et al.*, 2023)	Y	Y	Y	Y	Y	N	NA	Y	Y	Y
(Abulezz *et al.*, 2022)	Y	Y	Y	Y	Y	N	NA	Y	Y	Y
(Biglari *et al.*, 2022)	Y	Y	Y	Y	Y	N	NA	Y	Y	Y
(Piscopo *et al.*, 2023)	Y	Y	N	Y	Y	N	NA	Y	Y	Y
(Wang *et al.* 2023)	Y	Y	N	Y	Y	N	NA	Y	Y	Y
(Zhang *et al.*, 2022)	Y	Y	Y	Y	Y	N	NA	Y	Y	Y
(Dragović *et al.*, 2022)	Y	Y	Y	Y	Y	N	NA	Y	Y	Y

Supplementary material 8: miRNAs differentially expressed in Alzheimer's disease patients compared to controls.

Study identification	Sample size		Sample type	miRNA quantification method	miRNAs	Up or down- regulated? AD X control	*Fold change*	p value
	Control	Case						
(Abolghasemi *et al.*, 2021)	50	50	Serum	RT-qPCR	miR-128<br>miR-138<br>miR-107	miR-128: NS<br>miR-138: down<br>miR-107: down	NI	miR-128: NS<br>miR-138: p=0.047<br>miR-107: p=0.004
(Barbagallo *et al.*, 2020)	30	30	Serum	RT-qPCR	miR-22*<br>miR-23a<br>miR-29a<br>miR-125b	miR-22*: up<br>miR-23a: up<br>miR-29a: up<br>miR-125b: up	miR-22*=1.43<br>miR-23a=2.37<br>miR-29a=1.94<br>miR-125b=2.00	miR-22*: p= 0.001<br>miR-23a: p= 0.009<br>miR-29a: p=0.033<br>miR-125b: p=0.004
(Bhatnagar *et al.*, 2014)	85	78	Plasma	RT-qPCR	miR-34a<br>miR-34c	miR-34a: up<br>miR-34c: up	NI	miR-34a: p<0.01<br>miR-34c: p<0.01
(Burgos *et al.*, 2014)	78	69	Serum	NGS	miR-34b-3p<br>miR-219-2-3p<br>miR-22-5p<br>miR-125b-1-3p<br>miR-1307-5p<br>miR-34c-5p<br>miR-34b-5p<br>miR-887<br>miR-182-5p<br>miR-135a-5p<br>miR-184<br>miR-30c-2-3p<br>miR-873-3p<br>miR-125a-3p<br>miR-671-3p<br>miR-21-5p<br>miR-1285-3p<br>miR-375<br>miR-3176<br>miR-127-3p	miR-34b-3p: up<br>miR-219-2-3p: up<br>miR-22-5p: up<br>miR-125b-1-3p: up<br>miR-1307-5p: up<br>miR-34c-5p: up<br>miR-34b-5p: up<br>miR-887: up<br>miR-182-5p: down<br>miR-135a-5p: up<br>miR-184: up<br>miR-30c-2-3p: up<br>miR-873-3p: up<br>miR-125a-3p: up<br>miR-671-3p: up<br>miR-21-5p: down<br>miR-1285-3p: up<br>miR-375: down<br>miR-3176: up<br>miR-127-3p: up	miR-34b-3p: 2.36<br>miR-219-2-3p: 1.93<br>miR-22-5p: 1.38<br>miR-125b-1-3p: 1.32<br>miR-1307-5p: 1.39<br>miR-34c-5p: 1.57<br>miR-34b-5p: 1.71<br>miR-887: 1.34<br>miR-182-5p: -1.01<br>miR-135a-5p: 1.66<br>miR-184: 1.43<br>miR-30c-2-3p: 1.22<br>miR-873-3p: 1.3<br>miR-125a-3p: 1.27<br>miR-671-3p: 1.21<br>miR-21-5p: -0.82<br>miR-1285-3p: 1.03<br>miR-375: -0.94<br>miR-3176: 1.06<br>miR-127-3p: 0.92	miR-34b-3p: p=1.85E-05<br>miR-219-2-3p: p=5.37E-04<br>miR-22-5p: p=5.37E-04<br>miR-125b-1-3p: p=8.03E-04<br>miR-1307-5p: p=1.28E-03<br>miR-34c-5p: p=1.96E-03<br>miR-34b-5p: p=2.57E-03<br>miR-887: p=3.31E-03<br>miR-182-5p: p=3.82E-03<br>miR-135a-5p: p=4.84E-03<br>miR-184: p=4.84E-03<br>miR-30c-2-3p: p=4.84E-03<br>miR-873-3p: p=4.84E-03<br>miR-125a-3p: p=9.65E-03<br>miR-671-3p: p=9.65E-003<br>miR-21-5p: p=2.35E-02<br>miR-1285-3p: p=2.45E-02<br>miR-375: p=3.26E-02<br>miR-3176: p=3.35E-02<br>miR-127-3p: p=3.74E-02
(Cao *et al.*, 2020)	93	108	Serum	RT-qPCR	miR-193a-3p	down	NI	p<0.001

(Cosin-Tomas *et al.*, 2017)	Cohort 1: 21<br><br>Cohort 2: 15	Cohort 1: 21<br><br>Cohort 2: 15	Plasma	RT-qPCR	Cohort 1:<br>let-7d-5p<br>let-7g-5p<br>miR-15b-5p<br>miR-34a-5p<br>miR-34c-5p<br>miR-142-3p<br>miR-146a-5p<br>miR-191a-5p<br>miR-301a-5p<br>miR-545-3p<br><br>Cohort 2:<br>miR-34a-5p<br>miR-545-3p	Cohort 1:<br>let-7d-5p:NS<br>let-7g-5p:NS<br>miR-15b-5p:down<br>miR-34a-5p: down<br>miR-34c-5p:NS<br>miR-142-3p: down<br>miR-146a-5p:NS<br>miR-191a-5p:NS<br>miR-301a-5p:NS<br>miR-545-3p: down<br><br>Cohort 2:<br>miR-34a-5p: NS<br>miR-545-3p: NS	NI	Cohort 1:<br>let-7d-5p: NS<br>let-7g-5p: NS<br>miR-15b-5p: p<0.01<br>miR-34a-5p: p<0.01<br>miR-34c-5p: NS<br>miR-142-3p: p<0.05<br>miR-146a-5p: NS<br>miR-191a-5p: NS<br>miR-301a-5p: NS<br>miR-545-3p: p<0.01<br><br>Cohort 2:<br>miR-34a-5p: NS<br>miR-545-3p: NS
(Denk *et al.*, 2018)	Total: 44<br>Serum:3<br>8	Total: 48<br>Serum:4<br>7	Serum	RT-qPCR	miR-103a-3p<br>miR-142-3p<br>miR-20a-5p<br>miR-29b-3p<br>miR-132-3p<br>miR-143-3p<br>miR-197-3p<br>miR-27a-3p<br>miR-338-3p<br>miR491-5p<br>miR-100-5p<br>miR-335-5p<br>miR-99a-5p<br>let-7b-5p<br>let-7g-5p<br>miR-106a-5p<br>miR-106b-5p<br>miR-18b-5p<br>miR-223-3p<br>miR-26a-5p<br>miR-26b-5p	miR-103a-3p: up<br>miR-142-3p: up<br>miR-20a-5p: up<br>miR-29b-3p: up<br>miR-132-3p: down<br>miR-143-3p: NS<br>miR-197-3p: NS<br>miR-27a-3p: NS<br>miR-338-3p: NS<br>miR491-5p: NS<br>miR-100-5p: NS<br>miR-335-5p: NS<br>miR-99a-5p: NS<br>let-7b-5p: up<br>let-7g-5p: up<br>miR-106a-5p: up<br>miR-106b-5p: up<br>miR-18b-5p: up<br>miR-223-3p: up<br>miR-26a-5p: up<br>miR-26b-5p: up	NI	miR-103a-3p: p<0.001<br>miR-142-3p: p<0.001<br>miR-20a-5p: p<0.001<br>miR-29b-3p: p<0.001<br>miR-132-3p: p<0.001<br>miR-143-3p: NS<br>miR-197-3p: NS<br>miR-27a-3p: NS<br>miR-338-3p: NS<br>miR491-5p: NS<br>miR-100-5p: NS<br>miR-335-5p: NS<br>miR-99a-5p: NS<br>let-7b-5p: p<0.01<br>let-7g-5p: p<0.001<br>miR-106a-5p: p<0.001<br>miR-106b-5p: p<0.001<br>miR-18b-5p: p<0.001<br>miR-223-3p: p<0.001<br>miR-26a-5p: p<0.001<br>miR-26b-5p: p<0.001

					miR-301a-3p<br>miR-30b-5p<br>miR-146a-5p<br>miR-15a-5p<br>miR-22-3p<br>miR-320a<br>miR-320b<br>miR-92a-3p<br>miR-1246	miR-301a-3p: up<br>miR-30b-5p: up<br>miR-146a-5p: down<br>miR-15a-5p: down<br>miR-22-3p: dpwn<br>miR-320a: down<br>miR-320b: down<br>miR-92a-3p:down<br>miR-1246: down		miR-301a-3p: p<0.001<br>miR-30b-5p: p<0.001<br>miR-146a-5p: p<0.001<br>miR-15a-5p: p<0.001<br>miR-22-3p: p<0.001<br>miR-320a: p<0.001<br>miR-320b: p<0.001<br>miR-92a-3p: p<0.001<br>miR-1246: p<0.001
(Dong *et al.*, 2015)	Total:123<br>Serum: 75	Total:127<br>Serum: 79	Serum	RT-qPCR	miR-31<br>miR-93<br>miR-143<br>miR-146a	miR-31: down<br>miR-93: down<br>miR-143: down<br>miR-146a:down	miR-31: 0.466<br>miR-93: 0.491<br>miR-143: 0.436<br>miR-146a: 0.471	miR-31: p<0.001<br>miR-93: p<0.001<br>miR-143: p<0.001<br>miR-146a: p<0.001
(Dong *et al.*, 2021)	86	Mild AD: 31<br>Moderate AD: 52<br>Severe AD: 38	Serum	RT-qPCR	miR-202	down	NI	p<0.001
(Nagaraj *et al.*, 2017)	Study 1: 6<br><br>Study 2: 9	Study 1: 7<br><br>Study 2: 13	Plasma	RT-qPCR	Study 1 e 2:<br>miR-151a-5p<br>miR-30b-5p<br>miR-486-5p<br>miR-33a-5p<br>miR-483-5p<br>miR-18a-5p<br>miR-320a<br>miR-320b<br>miR-320c<br>miR-502-3p<br>miR-103a-3p<br>miR-301a-3p<br>miR-142-3p<br>miR-200a-3p<br>miR-1260a	Study 1:<br>miR-151a-5p: NS<br>miR-30b-5p: down<br>miR-486-5p: NS<br>miR-33a-5p: down<br>miR-483-5p: up<br>miR-18a-5p: down<br>miR-320a: up<br>miR-320b: up<br>miR-320c: up<br>miR-502-3p: up<br>miR-103a-3p: down<br>miR-301a-3p: down<br>miR-142-3p: down<br>miR-200a-3p: up<br>miR-1260a: up<br>Study 2:	Study 1:<br>miR-151a-5p: NS<br>miR-30b-5p: -1.5<br>miR-486-5p: NS<br>miR-33a-5p: -2.03<br>miR-483-5p: 4.723<br>miR-18a-5p: -1.45<br>miR-320a: 1.553<br>miR-320b: 1.723<br>miR-320c: 2.035<br>miR-502-3p: 1.786<br>miR-103a-3p: -1.29<br>miR-301a-3p: -1.76<br>miR-142-3p: -1.97<br>miR-200a-3p: 5.267<br>miR-1260a: 2.009<br>Study 2:	Study 1:<br>miR-151a-5p: NS<br>miR-30b-5p: p<0.01<br>miR-486-5p: NS<br>miR-33a-5p: p<0.05<br>miR-483-5p: p<0.05<br>miR-18a-5p: p<0.05<br>miR-320a: p<0.01<br>miR-320b: p<0.01<br>miR-320c: p<0.01<br>miR-502-3p: p<0.05<br>miR-103a-3p: p<0.05<br>miR-301a-3p: p<0.05<br>miR-142-3p: p<0.01<br>miR-200a-3p: p<0.01<br>miR-1260a: p<0.05<br>Study 2:

						miR-151a-5p: down<br>miR-30b-5p: down<br>miR-486-5p: up<br>miR-33a-5p: NS<br>miR-483-5p: up<br>miR-18a-5p: NS<br>miR-320a: NS<br>miR-320b: NS<br>miR-320c: NS<br>miR-502-3p: up<br>miR-103a-3p: down<br>miR-301a-3p: NS<br>miR-142-3p: NS<br>miR-200a-3p: up<br>miR-1260a: NS	miR-151a-5p: -2.17<br>miR-30b-5p: -1.93<br>miR-486-5p: 5.11<br>miR-33a-5p: NS<br>miR-483-5p: 13.45<br>miR-18a-5p: NS<br>miR-320a: NS<br>miR-320b: NS<br>miR-320c: NS<br>miR-502-3p: 2.701<br>miR-103a-3p: -1.62<br>miR-301a-3p: NS<br>miR-142-3p: NS<br>miR-200a-3p: 3.15<br>miR-1260a: NS	miR-151a-5p: p<0.001<br>miR-30b-5p: p<0.01<br>miR-486-5p: : p<0.001<br>miR-33a-5p: NS<br>miR-483-5p:p<0.0001<br>miR-18a-5p: NS<br>miR-320a: NS<br>miR-320b: NS<br>miR-320c: NS<br>miR-502-3p: p<0.0001<br>miR-103a-3p: p<0.01<br>miR-301a-3p: NS<br>miR-142-3p: NS<br>miR-200a-3p: p<0.01<br>miR-1260a: NS
(Galimberti *et al.*, 2014)	18	22	Serum	RT-qPCR	miR-125b<br>miR-23a<br>miR-26b	miR-125b: down<br>miR-23a: down<br>miR-26b: down	NI	Serum<br>miR-125b: 0.009<br>miR-23a:<0.001<br>miR-26b:<0.01
(Geekiyanage *et al.*, 2012)	7	7	Serum	RT-qPCR	miR-137<br>miR-181c<br>miR-9<br>miR-29a<br>miR-29b<br>miR-126:NS<br>miR-22:NS<br>miR-191:NS	miR-137: down<br>miR-181c: down<br>miR-9: down<br>miR-29a: down<br>miR-29b: down<br>miR-126: NS<br>miR-22: NS<br>miR-191: NS	NI	miR-137: p<0.05<br>miR-181c: p<0.05<br>miR-9: p<0.05<br>miR-29a: p<0.05<br>miR-29b: p<0.05<br>miR-126:NS: p<0.05<br>miR-22:NS: p<0.05<br>miR-191:NS: p<0.05
(Giuliani *et al.*, 2021)	40	116	Plasma	qPCR	miR-17-5p<br>miR-21-5p<br>miR-126-3p<br>miR-146-5p:NS	miR-17-5p: up<br>miR-21-5p: up<br>miR-126-3p: up<br>miR-146-5p: NS	NI	miR-17-5p: 0.003<br>miR-21-5p: 0.0004<br>miR-126-3p: 0.0004<br>miR-146-5p: NS
(Guo *et al.*, 2017)	86	Mild AD:31<br>Moderate AD:52	Serum	RT-qPCR	miR-26a-5p<br>miR-181c-3p<br>miR-126-5p<br>miR-22-3p<br>miR-148b-5p	miR-26a-5p: down<br>miR-181c-3p: down<br>miR-126-5p: down<br>miR-22-3p: down<br>miR-148b-5p: down	NI	miR-26a-5p: <0.001<br>miR-181c-3p: <0.001<br>miR-126-5p: <0.001<br>miR-22-3p: <0.001<br>miR-148b-5p: <0.001

		Severe AD:38			miR-106b-3p<br>miR-144-5p<br>miR-6119-5p<br>miR-1246<br>miR-660-5p	miR-106b-3p: up<br>miR-144-5p: down<br>miR-6119-5p: up<br>miR-1246: up<br>miR-660-5p: up		miR-106b-3p: <0.001<br>miRNA-144-5p: <0.001<br>miR-6119-5p: <0.001<br>miR-1246: <0.001<br>miR-660-5p: <0.001
(Guven *et al.*, 2021)	12	26	Plasma	RT-qPCR	mir-146a<br>mir-144<br>mir-34a	mir-146a: NS<br>mir-144: NS<br>mir-34a: NS	mir-146a: NS<br>mir-144: NS<br>mir-34a: NS	mir-146a: NS<br>mir-144: NS<br>mir-34a: NS
(Hajjri *et al.*, 2020)	15	20	Plasma	RT-qPCR	miR-4422<br>miR-3714	miR-4422: down<br>miR-3714: NS	miR-4422: 0.43<br>miR-3714: NS	miR-4422: 0.018<br>miR-3714: NS
(Han *et al.*, 2020)	30	33	Serum	RT-qPCR	miR-22	down	NI	p<0.001
(Hara *et al.*, 2017)	22	36	Serum	RT-qPCR	miR-501-3p<br>let-7f-5p<br>miR-26b-5p	miR-501-3p: down<br>let-7f-5p: NS<br>miR-26b-5p: NS	NI	miR-501-3p: 0.00002<br>let-7f-5p: NS<br>miR-26b-5p: NS
(Heydari *et al.*, 2021)	23	21	Serum	RT-qPCR	miR-324-3p<br>miR-331-3p	miR-324-3p: NS<br>miR-331-3p: NS	miR-324-3p: NS<br>miR-331-3p: NS	miR-324-3p: NS<br>miR-331-3p: NS
(Jia & Liu, 2016)	62	84	Serum	RT-qPCR	miR-29<br>miR-125b<br>miR-223<br>miR-519	miR-29: down<br>miR-125b: down<br>miR-223: down<br>miR-519: up	NI	miR-29: p<0.01<br>miR-125b:p<0.001<br>miR-223: p<0.001<br>miR-519: p<0.001
(Kiko *et al.*, 2014)	10	10	Plasma	RT-qPCR	miR-9<br>miR-29a<br>miR-29b<br>miR34a<br>miR-125b<br>miR-146a	miR-9: NS<br>miR-29a: NS<br>miR-29b: NS<br>miR-34a: down<br>miR-125b: NS<br>miR-146a: down	NI	miR-9: NS<br>miR-29a: NS<br>miR-29b: NS<br>miR34a: p<0.05<br>miR-125b: NS<br>miR-146a: p<0.05
(Kim *et al.*, 2021)	31	20	Plasma	RT-qPCR	miR-1273g-3p	up	NI	p<0.05
(Kumar *et al.*, 2017)	4	1	Serum	RT-qPCR	miR-455-3p<br>miR-4668-5p<br>miR-4674<br>miR-3613-3p<br>mir-6722	miR-455-3p: up<br>miR-4668-5p: up<br>miR-4674: NS<br>miR-3613-3p:NS<br>mir-6722:NS	miR-455-3p: 0.019 ± 0.020<br>miR-4668-5p: NS<br>miR-4674: NS<br>miR-3613-3p:NS<br>mir-6722:NS	miR-455-3p: p=0.007<br>miR-4668-5p: NS<br>miR-4674: NS<br>miR-3613-3p:NS<br>mir-6722: NS

(Li *et al.*, 2019)	65	51	Plasma	qRT-PCR	miR-101a	down	NI	p<0.01
(Liu & Lei, 2021)	60	110	Serum	qRT-PCR	miR-331-3p	down	NI	p<0.001
(Maffioletti *et al.*, 2019)	45	49	Plasma	qRT-PCR	miR-146a	NS	NS	NS
(Mancuso *et al.*, 2019)	40	40	Serum	qRT-PCR	miR-223-3p	down	NI	p<0.001
(Poursaei *et al.*, 2022)	50	50	Serum	qRT-PCR	let-7d-5p<br>let-7g-5p<br>miR-15b-5p	let-7d-5p: up<br>let-7g-5p: up<br>miR-15b-5p: NS	let-7d-5p: 2.14<br>let-7g-5p: 1.94<br>miR-15b-5p: NS	let-7d-5p: 0.007<br>let-7g-5p: 0.013<br>miR-15b-5p: NS
(Prendecki *et al.*, 2019)	74	64	Plasma	RT-qPCR	miR-107<br>miR-650	miR-107: NS<br>miR-650: NS	miR-107: NS<br>miR-650: NS	miR-107: NS<br>miR-650: NS
(Ragusa *et al.*, 2016)	40	40	Plasma	RT-qPCR	miR-10b<br>miR29a-3p<br>miR-130b-3p	miR-10b: down<br>miR29a-3p: down<br>miR-130b-3p: down	NI	miR-10b: p=0.022<br>miR29a-3p: p=0.041<br>miR-130b-3p: p=0.002
(Sabry *et al.*, 2021)	20	20	Plasma	RT-qPCR	miR-483-5p	up	NI	p<0.001
(Tan *et al.*, 2014a)	155	105	Serum	RT-qPCR	miR-9<br>miR-29a<br>miR-29b<br>miR-101<br>miR-125b<br>miR-181c	miR-9: down<br>miR-29a: NS<br>miR-29b: NS<br>miR-101: NS<br>miR-125b: up<br>miR-181c: up	miR-9: 2.75<br>miR-29a: NS<br>miR-29b: NS<br>miR-101: NS<br>miR-125b: 0.04<br>miR-181c: 0.18	miR-9: 0.002<br>miR-29a: NS<br>miR-29b: NS<br>miR-101: NS<br>miR-125b: 4.86 × 10−7<br>miR-181c: 3.34 × 10−5
(Tan *et al.*, 2014b)	155	158	Serum	RT-qPCR	miR-98-5p<br>miR-885-5p<br>miR-483-3p<br>miR-342-3p<br>miR-191-5p<br>miR-let-7d-5p	miR-98-5p: down<br>miR-885-5p: down<br>miR-483-3p: down<br>miR-342-3p: down<br>miR-191-5p: down<br>miR-let-7d-5p: down	NI	miR-98-5p: 2.67*10^-4<br>miR-885-5p: 2.8*10^-4<br>miR-483-3p: 1.00*10^-4<br>miR-342-3p: 9.19*10^-16<br>miR-191-5p: 1.54*10^-9<br>miR-let-7d-5p: 1.2*10^-6
(Wang *et al.*, 2020)	120	120	Plasma	RT-qPCR	miR-103<br>miR-107	miR-103: down<br>miR-107: down	NI	miR-103: p<0.001<br>miR-107: p<0.001
(Wang & Zhang, 2020)	62	118	Serum	RT-qPCR	miR-433	down	NI	p<0.01
(Wu *et al.*, 2017)	60	65	Serum	RT-qPCR	miR-146a-5p<br>miR-106b-3p	miR-146a-5p:up<br>miR-106b-3p:up	NI	miR-146a-5p: p<0.05<br>miR-106b-3p: p<0.05

					miR-195-5p<br>miR-20b-5p<br>miR-497-5p<br>miR-125b-3p<br>miR-29c-3p<br>miR-93-5p<br>miR-19b-3p<br>miR-424-3p	miR-195-5p:up<br>miR-20b-5p:up<br>miR-497-5p:up<br>miR-125b-3p:down<br>miR-29c-3p:down<br>miR-93-5p:down<br>miR-19b-3p:down<br>miR-424-3p: NS		miR-195-5p: p<0.05<br>miR-20b-5p: p<0.05<br>miR-497-5p: p<0.05<br>miR-125b-3p: p<0.05<br>miR-29c-3p: p<0.05<br>miR-93-5p: p<0.05<br>miR-19b-3p: p<0.05<br>miR-424-3p: NS
(Zeng *et al.*, 2017)	30	Mild AD: 30<br>Moderate AD: 30	Serum	RT-qPCR	miR-222	down	NI	p<0.05
(Zhang *et al.*, 2021a)	100	110	Serum	RT-qPCR	miR-148a-3p	down	NI	p<0.001
(Zhang *et al.*, 2021b)	106	117	Serum	RT-qPCR	miR-128	up	NI	p<0.01
(Zhao *et al.*, 2020)	12	19	Serum	RT-qPCR	miR-206<br>miR-208b-3p<br>miR-640<br>miR-19a-5p<br>miR-518f-5p<br>miR-193a-3p<br>miR-346<br>miR-122-3p<br>miR-145-5p<br>miR-143-5p<br>miR-106a-3p<br>miR-1246	miR-206: down<br>miR-208b-3p: down<br>miR-640: up<br>miR-19a-5p: up<br>miR-518f-5p: NS<br>miR-193a-3p: down<br>miR-346: up<br>miR-122-3p: up<br>miR-145-5p: up<br>miR-143-5p: up<br>miR-106a-3p: NS<br>miR-1246: up	NI	miR-206: p=0.0129<br>miR-208b-3p: p=0.0055<br>miR-640: p=0.0041<br>miR-19a-5p: p=0.0139<br>miR-518f-5p: NS<br>miR-193a-3p: p=0.0488<br>miR-346: p=0.0075<br>miR-122-3p: p=0.0189<br>miR-145-5p: p=0.0318<br>miR-143-5p: p=0.0127<br>miR-106a-3p: NS<br>miR-1246: p=0.0033
(Zhu *et al.*, 2015)	42	26	Serum	RT-qPCR	miR-210	down	NI	p<0.01
(Zirnheld *et al.*, 2016)	37	Mild AD: 16<br>Moderate / Severe AD: 20	Plasma	RT-qPCR	miR-34c<br>miR-181c<br>miR-411	miR-34c: up<br>miR-181c: up<br>miR-411: up	NI	miR-34c:<br>Mild AD vs. Cont: p=0.027<br>Sev/Mod AD vs. Cont: p=0.022<br>miR-181c:<br>Mild AD vs. Cont: p=0.036<br>Sev/Mod AD vs. Cont: p=0.002

								miR-411<br>Mild AD vs. Cont: p<0.001<br>Sev/Mod AD vs. Cont: p<0.001
(Abed *et al.*, 2023)	40	40	Plasma	RT-qPCR	miR-146a	NS	NS	NS
(Abuelezz *et al.*, 2022)	23	23	Serum	RT-qPCR	miR-34a<br>miR-29b<br>miR-181c	miR-34a: down<br>miR-29b: down<br>miR-181c: down	miR-34a: 0.23<br>miR-29b: 0.37<br>miR-181c: 0.42	miR-34a: 0.032<br>miR-29b: 0.033<br>miR-181c: 0.042
(Biglari *et al.*, 2022)	50	50	Plasma	RT-qPCR	miR502-3p<br>miR200a-3p	miR502-3p: up<br>miR200a-3p: up	miR502-3p: 1.9437<br>miR200a-3p: 2.088	miR-502-3p: p=0.0106<br>miR-200a-3p: p=0.000
(Piscopo *et al.*, 2023)	43	33	Plasma	RT-qPCR	miR-92a-3p<br>miR-320a<br>miR-320b	miR-92a-3p: down<br>miR-320a: NS<br>miR-320b: up	NI	miR-92a-3p: p=0.037<br>miR-320a: NS<br>miR-320b: p<0.05
(Wang *et al.*, 2023)	68	92	Serum	RT-qPCR	miR-511-3p	down	NI	p<0.001
(Zhang *et al.*, 2022)	93	98	Serum	RT-qPCR	miR-381-3p	down	NI	p<0.001
(Dragović *et al.*, 2022)	18	12	Plasma	RT-qPCR	miR-146a: NS<br>miR-155: NS	miR-146a: NS<br>miR-155: NS	miR-146a: NS<br>miR-155: NS	miR-146a: NS<br>miR-155: NS

Supplementary material 9: Data from articles included in systematic review of T2DM.

Author/Year	T2DM Diagnostic criteria	Sample size		Country of origin	Age		Sex M=male / F=female		Statistically significant difference between	
		Control	Case		Control	Case	Control	Case	Age	Sex
(de Candia *et al.*, 2017)	ADA	9	9	Italy	57.9 ± 8.9	60.2 ± 8.0	4M / 5F	2M / 7F	No	No
(Baldeon Rojas *et al.*, 2016)	The Expert Committee on the diagnosis and classification of Diabetes Mellitus	44	64	Ecuador	53 (32–87)	61 (37–85)	13M / 31F	24M / 40F	Yes	NI
(Kong *et al.*, 2011)	Laboratory tests	19	18	China	41.00 ± 2.616	47.33 ± 2.617	12M / 7F	9M / 9F	No	No
(Yang *et al.*, 2014)	WHO	20	24	China	46.65 ± 16.181	51.13 ± 9.214	8M / 12F	16M / 8F	No	NI
(Yang *et al.*, 2017)	ADA	5	10	China	56.4 ± 3.7	58.2 ± 7.7	2M / 3F	4M / 6F	NI	NI
(Pescador *et al.*, 2013)	WHO / IDF	20	13	Spain	42.9 ± 12.13	69.40 ± 7.12	10M / 10F	7M / 6F	Yes	No
(Alipoor *et al.*, 2018)	Laboratory tests	30	30	Iran	50.5 (45.75–61)	57 (48–61)	9M / 21F	11M / 19F	No	No
(Li *et al.*, 2016)	WHO	20	30	China	59.78 ± 11.23	60.79 ± 11.11	Only women	Only women	No	No

(Rezk *et al.*, 2016)	ADA	100	100	Egypt	45.7 ± 8.4	48.2 ± 8.2	46M / 54F	49M / 51F	No	No
(Sucharita *et al.*, 2018)	ADA	30	30	India	42.1 ± 7.8	50.5 ± 6.3	21M / 9F	21M / 9F	Yes	No
(Liu *et al.*, 2014)	WHO	138	160	China	46.7 ± 7.2	50.2 ± 6.7	67M / 71F	78M / 82F	No	No
(Ali Beg *et al.*, 2020)	Laboratory tests	100	100	India	50% < 50years and 50% > 50 years	60% < 50 years and 40% > 50 years	57M / 43F	55M / 45F	NI	NI
(Wang *et al.*, 2018)	WHO	20	36	China	55.6 ± 4.5	52.5 ± 7.0	10M / 10F	26M / 10F	NI	NI
(Wu *et al.*, 2015)	Laboratory tests	25	25	China	45.5 ± 10.2	46.7 ± 8.6	NI	NI	No	NI
(Ortega *et al.*, 2014)	ADA	35	30	Spain	48.1 ± 10.1	54 ± 10	Only men	Only men	Yes	No
(Amr *et al.*, 2018)	ADA	20	54	Egypt	58.1 ± 1.1	56.5 ± 7.7	11M / 9F	29M / 25F	No	No
(Motawi *et al.*, 2018)	ADA	25	25	Egypt	45.8 ± 5.82	48.76 ± 7.75	16M / 9F	23M / 2F	No	NI
(Zhang *et al.*, 2013)	Laboratory tests	30	30	China	61 ± 9	63 ± 8.56	16M / 14F	16M / 14F	No	No
(Abdelaty *et al.*, 2020)	ADA	25	20	Egypt	46.48 ± 3.93	48.75 ± 2.94	12M / 13F	11M / 9F	No	No
(Zou *et al.*, 2017)	WHO	127	65	China	47.25 ± 9.75	49.28 ± 8.54	66M / 61F	36M / 29F	No	No

(Seyhan *et al.*, 2016)	Laboratory tests	27	31	USA	25.3 ± 2.2	52.9 ± 2.0	15M / 12F	15M / 16F	Yes	No
(Lu *et al.*, 2021)	WHO	50	50	China	55 ± 7.8	57 ± 8.2	27M / 23F	24M / 26F	No	No
(Wang *et al.*, 2019b)	Laboratory tests	68	69	China	62.98 ± 7.42	64.29 ± 3.77	45M / 23F	48M / 21F	No	No
(Olivieri *et al.*, 2015)	ADA	107	76	Italy	64.25 ± 7.56	65.56 ± 6.96	49M / 58F	36M / 40F	No	No
(Zhou *et al.*, 2020)	WHO	44	49	China	50.8 ± 17.1	55.4 ± 12.0	NI	NI	No	NI
(Seleem *et al.*, 2019)	Laboratory tests	50	50	Egypt	62.22 ± 0.69	62.06 ± 1.26	NI	NI	No	NI
(Motawae *et al.*, 2015)	Laboratory tests	50	50	Egypt	62.22 ± 0.69	62.06 ± 1.26	36M / 14F	32M / 18F	No	No
(Zhang *et al.*, 2017)	Standards of Medical Care in Diabetes - 2015	79	93	China	Men: 63.5±7.4 Women: 62.1±8.2	Men: 66.7±6.8 Women: 65.3±7.5	53M / 26F	61M / 32F	No	No
(Wan *et al.*, 2017)	WHO	74	76	China	48.8 (15.2)	48.5 (14.5)	41M / 33F	50M / 26F	No	No
(Wang *et al.*, 2016)	WHO	92	92	China	50.2 (14.2)	47.7 (13.9)	56M / 36F	58M / 34F	No	No
(Rong *et al.*, 2013)	WHO	90	90	China	48.00 (41.75-55.00)	48.50 (42.00-56.00)	47M / 43F	47M / 43F	No	No
(Higuchi *et al.*, 2015)	Laboratory tests	49	155	Japan	46.0 ± 9.67	62.3 ± 13.2	25M / 24F	96M / 59F	Yes	NI

(Fejes *et al.*, 2017)	Laboratory tests	23	28	Hungary	53 (34–60)	53 (50–59)	12M / 11F	15M / 13F	No	No
(Wang *et al.*, 2019a)	Laboratory tests	40	40	China	55.12 ± 4.99	56.72 ± 6.07	21M / 19F	22M / 18F	No	No
(Wan, 2020)	Laboratory tests	100	102	China	>55 years: 58 (58.00) ≤55 years: 42 (42.00)	>55 years: 56 (54.90) ≤55 years: 46 (45.10)	52M / 48F	55M / 47F	NI	No
(Li *et al.*, 2020a)	WHO	32	32	China	44.7 ± 7.1	45.1 ± 6.9	17M / 15F	18M / 14F	No	No
(Ma *et al.*, 2016)	WHO	127	157	China	51.33 ± 9.16	51.4 ± 8.57	NI	NI	No	NI
(Akhbari *et al.*, 2018)	ADA	22	21	Iran	47.2 ± 7.2	61.5 ± 6.5	9M / 13F	11M / 10F	NI	NI
(Sun *et al.*, 2014)	WHO	100	100	China	48.55 ± 12.41	51.33 ± 11.75	44M / 56F	54M / 46F	No	No
(Yan *et al.*, 2016)	ADA	50	50	China	45.52 ± 6.215	46.22 ± 6.897	22M / 28F	27M / 23F	No	NI
(Shaker *et al.*, 2019)	ADA	81	30	Egypt	50.7 ± 7.7	51.0 ± 6.1	54M / 27F	19M / 11F	No	No
(Baldeon *et al.*, 2014)	The Expert Committee on the diagnosis and classification of Diabetes Mellitus	40	56	Ecuador	54 (32–87)	62 (38–85)	12M / 28F	22M / 34F	Yes	NI

(Parsa *et al.*, 2020)	Laboratory tests	40	50	Iran	52.82 ± 6.94	54.92 ± 7.05	25M / 15F	24M / 16F	No	No
(Li *et al.*, 2020b)	Laboratory tests	60	60	China	54.7 ± 10.1	55.1 ± 11.9	36M / 24F	34M / 26F	No	No
(Nie *et al.*, 2020)	American Association of Clinical Endocrinologists and American College of Endocrinology on the comprehensive type 2 diabetes management	80	80	China	64.95 ± 1.19	67.81 ± 1.11	45M / 35F	43M / 37F	No	No
(Khan *et al.*, 2020)	Laboratory tests	5	5	India	34 ± 6.26	42 ± 7.16	Only men	Only men	No	No
(Elemam *et al.*, 2021)	Laboratory tests	50	50	United Arab Emirates	44.16 ± 12.19	44.36 ± 10.20	18M / 32F	18M / 32F	No	No
(Prabu *et al.*, 2015)	WHO	49	49	India	44.3 ± 6.9	44.4 ± 8.1	26M / 23F	25M / 24F	No	No
(Garcia-Jacobo *et al.*, 2019)	ADA	35	54	Mexico	46.1 ± 0.9	49.9 ± 10.2	24M / 11F	28M / 26F	No	NI
(Mensa *et al.*, 2019)	Laboratory tests	188	144	Italy	65.6 (15.5)	66.8 (7.7)	92M / 96F	66F / 78F	No	No
(Monfared *et al.*, 2020)	WHO	40	40	Iran	33.59 ± 7.58	53.69 ± 5.69	NI	NI	No	NI

(Luo *et al.*, 2020)	WHO	50	48	China	45.62 ± 8.58	52.6 ± 9.13	26M / 24F	26M / 22F	No	No
(Luo *et al.*, 2019)	WHO	32	47	China	58.6 ± 8.1	60.46 ± 11.14	17M / 15F	23M / 24F	No	NI
(Sadeghzadeh *et al.*, 2020)	Laboratory tests	30	30	Iran	51.44 ± 6.04	52.42 ± 8.77	21M / 9F	20M / 10F	No	No
(Zeinali *et al.*, 2021)	ADA	30	30	Iran	55.37 ± 8.47	53.03 ± 9.66	15M / 15F	15M / 15F	No	No
(Shao *et al.*, 2017)	ADA	195	186	China	54.12 ± 9.45	54.87 ± 11.65	99M / 96F	95M / 91F	No	No
(Lv *et al.*, 2015)	ADA	131	137	China	51 ± 11	50 ± 10	67M / 64F	72M / 65F	No	No
(Samanian *et al.*, 2019)	ADA	20	20	Iran	37.35 + 8.97	44.80 + 1.20	Only women	Only women	No	No
(Alfaifi *et al.*, 2020)	Laboratory tests	200	200	India	47.63 ± 6.76	46.32 ± 7.18	140M / 60F	136M / 64F	No	No
(Prabu *et al.*, 2020)	WHO	40	40	India	48 ± 10.0	54 ± 6.0	21M / 19F	21M / 19F	No	No
(Olivieri *et al.*, 2014)	ADA	92	193	Italy	65.8 ± 11.6	65.9 ± 7.8	47M / 45F	109M / 84F	No	No
(La Sala *et al.*, 2019)	ADA	44	27	Italy	59.3 (9.82)	61.69 (7.59)	15M / 29F	10M / 17F	No	No
(Al-Hayali *et al.*, 2019)	ADA	45	45	Turkey	60.23 ± 6.27	61.50 ± 5.08	22M / 23F	22M / 23F	No	No

(Huang *et al.*, 2018)	WHO	24	24	China	34.71 ± 7.62	34.33 ± 14.18	8M / 16F	8M / 16F	No	No
(Liang *et al.*, 2018)	WHO	94	112	China	52.84 ± 8.85	54.75 ± 7.53	53M / 41F	69M / 43F	No	No
(Yang *et al.*, 2016)	WHO	30	58	China	48.2 ± 8.9	49.8 ± 9.1	NI	NI	No	NI
(Dias, 2016)	WHO	4	4	South Africa	46.3 ± 5.7	46.8 ± 6.6	Only women	Only women	No	No
(ESPINOZA, 2018)	Laboratory tests	16	17	Peru	45.19 ± 10.81	49.88 ± 6.67	8M / 8F	9M / 8F	No	No
(Abdel-Tawab *et al.*, 2023)	ADA	50	50	Egypt	50.2 ± 6.4	50.2 ± 5.1	17M / 33F	15M / 35F	No	NI
(Abdou *et al.*, 2022)	ADA	20	20	Egypt	56.60 ± 7.7	48.95 ± 7.9	11M / 9F	10M / 10F	NI	NI
(Aghaei Zarch *et al.*, 2024)	ADA	50	50	Iran	52.4 ± 6.8	55.2 ± 4.8	27M / 23F	24M / 26F	No	No
(Aljaibeji *et al.*, 2022)	Laboratory tests	25	29	United Arab Emirates	42.8 ± 12.7	55.6 ± 9.0	9M / 16F	13M / 16F	Yes	NI
(Banerjee *et al.*, 2022)	ADA	30	30	India	40.6 ± 5.95	38.9 ± 5.8	17M / 13F	19M / 11F	No	No
(Del Cuore *et al.*, 2023)	ADA	20	40	Italy	67.05 ± 13.82	66.95 ± 11.92	12M / 8F	27M / 13F	No	No
(Dzung *et al.*, 2023)	ADA	93	105	Vietnam	55 (34-70)	55 (39-70)	38M / 55F	52M / 53F	No	No

(Ezzat *et al.*, 2023)	WHO	128	129	Egypt	59.1 ± 6.7	58.7 ± 7.8	67M / 61F	66M / 63F	No	No
(Ghoreishi *et al.*, 2022)	WHO	20	20	Iran	≤45 years: 39.33±2.12<br>46–60 years: 50.5±4.59<br>>61 years: 63.8±3.03	≤45 years: 40.6±3.78<br>46–60 years: 51.88±4.40<br>>61 years: 66.16±2.63	13M / 7F	7M / 13F	No	No
(Greco *et al.*, 2023)	ADA	10	10	Italy	53.50 (47–58)	58 (57–65)	4M / 6F	4M / 6F	No	No
(Guo *et al.*, 2023)	Laboratory tests	17	19	China	49.6 ± 12.5	54.9 ± 14.2	8M / 9F	9M / 10F	No	No
(Hu *et al.*, 2022)	Laboratory tests	113	113	China	61.1 (7.1)	61.1 (7.1)	53M / 60F	53M / 60F	No	No
(Kong *et al.*, 2022)	WHO	342	326	China	54.57 ± 17.93	61.99 ± 14.90	160M / 182F	155M / 171F	Yes	No
(Lin *et al.*, 2021)	ADA	30	36	China	54.31 ± 4.81	55.05 ± 6.66	14M / 16F	20M / 16F	No	No
(Liu *et al.*, 2022a)	Laboratory tests	50	76	China	57.9 ± 10.3	57.3 ± 11.1	24M / 26F	32M / 44F	No	No
(Liu *et al.*, 2022b)	ADA	180	180	China	53.78 ± 8.22	54.04 ± 7.30	98M / 82F	114M / 66F	No	No
(Meng *et al.*, 2023)	ADA	30	36	China	63.83 ± 12.82	60.17 ± 12.43	14M / 16F	19M / 17F	No	No

(Mokhtari Ardekani *et al.*, 2023)	ADA	145	150	Iran	58.8 ± 9.4	60.5 ± 9.5	79M / 66F	82M / 68F	No	No
(Monfared *et al.*, 2022)	WHO	40	40	Iran	44 ± 2	47 ± 2	19M / 21F	15M / 25F	No	No
(Nemecz *et al.*, 2023)	ADA	15	17	Romania	43 ± 8	64.1 ±13	2M / 3F	5M / 2F	NI	NI
(Pan *et al.*, 2022)	WHO	36	12	China	40.00 – 13.54	56.00 – 12.10	18M / 18F	6M / 6F	Yes	No
(Ruan *et al.*, 2023)	ADA	52	85	China	53.38 ± 8.59	52.75 ± 9.66	33M / 19F	54M / 31F	No	No
(Saleh *et al.*, 2022)	ADA	80	80	Egypt	57.5 ± 8.6	57.3 ± 9.1	48M / 32F	52M / 28F	No	No
(Ghaneh *et al.*, 2023)	ADA	17	16	Iran	51.35 ± 6.04	51.50 ± 5.40	8M / 9F	7M / 9F	NI	NI
(Shahouzehi *et al.*, 2021)	ADA	15	15	Iran	46.93 ± 12.29	47.27 ± 11.83	6M / 9F	6M / 9F	No	No
(Simoniene *et al.*, 2022)	Laboratory tests	54	53	Lithuania	62 (48–80)	65 (44–83)	25M / 29F	24M / 29F	No	No
(Su *et al.*, 2022)	Laboratory tests	101	101	China	51 (42-55)	51 (41-56)	66M / 35F	66M / 35F	No	No
(Sun *et al.*, 2023)	WHO	9	10	China	42.78 ± 13.43	52.40 ± 11.03	4M / 5F	4M / 6F	Yes	No
(Sun *et al.*, 2022)	WHO	30	50	China	53.57 ± 8.31	55.86 ± 10.48	16M / 14F	29M / 21F	No	No

(Tonyan *et al.*, 2023)	WHO	22	44	Russia	42.90 ± 16.52	73.15 ± 7.72	11M / 11F	14M / 30F	Yes	NI
(Tursinawati *et al.*, 2022)	Laboratory tests	17	30	Indonesia	45.35 ± 1.59	57.60 ± 1.39	5M / 12F	6M / 24F	Yes	No
(Wu *et al.*, 2021a)	Laboratory tests	60	60	China	54.7 ± 10.1	55.1 ± 11.9	36M / 24F	34M / 26F	No	No
(Wu *et al.*, 2021b)	ADA	21	10	China	28.16	45.2	5M / 16F	7M / 3F	Yes	NI
(Yazdanpanah *et al.*, 2022)	ADA	29	24	Iran	50.42 ± 6.14	54.42 ± 7.76	19M / 10F	15M / 9F	No	No
(Yun *et al.*, 2022)	ADA/WHO	42	50	China	47.40 ± 16.18	48.12 ± 16.51	20M / 22F	30M / 20F	No	No
(Zhao *et al.*, 2023)	Laboratory tests	60	64	China	52.1 ± 8.9	53.3 ± 8.7	33M / 27F	34M / 30F	No	No

Abbreviations: ADA: American Diabetes Association / IDF: International Diabetes Federation / NI: not informed / WHO: World Health Organization.

Supplementary material 10: Risk of bias assessment of studies included in the type 2 diabetes mellitus systematic review.

**Study identification**	**JBI Critical Appraisal Checklist for Case Control Studies**										
	**1**	**2**	**3**	**4**	**5**	**6**	**7**	**8**	**9**	**10**	**Score**
(de Candia *et al.*, 2017)	Y	Y	Y	Y	Y	N	NA	Y	Y	Y	10
(Baldeon Rojas *et al.*, 2016)	Y	N	Y	Y	Y	Y	Y	Y	Y	Y	8
(Kong *et al.*, 2011)	Y	Y	Y	Y	Y	N	NA	Y	Y	Y	10
(Yang *et al.*, 2014)	Y	U	Y	Y	Y	N	NA	Y	Y	Y	9
(Yang *et al.*, 2017)	Y	U	Y	Y	Y	N	NA	Y	Y	Y	9
(Pescador *et al.*, 2013)	Y	N	Y	Y	Y	Y	N	Y	Y	Y	5
(Alipoor *et al.*, 2018)	Y	Y	U	Y	Y	N	N	Y	Y	Y	9
(Li *et al.*, 2016)	Y	N	Y	Y	Y	Y	Y	Y	Y	Y	8
(Rezk *et al.*, 2016)	Y	Y	Y	Y	Y	N	NA	Y	Y	Y	10
(Sucharita *et al.*, 2018)	Y	N	Y	Y	Y	Y	Y	Y	Y	Y	8
(Liu *et al.*, 2014)	Y	Y	Y	Y	Y	N	NA	Y	Y	Y	10

(Ali Beg *et al.*, 2020)	Y	U	Y	Y	Y	N	NA	Y	Y	Y	9
(Wang *et al.*, 2018)	Y	U	Y	Y	Y	N	NA	Y	Y	Y	9
(Wu *et al.*, 2015)	Y	U	Y	Y	Y	U	U	Y	Y	Y	5
(Ortega *et al.*, 2014)	Y	N	Y	Y	Y	Y	Y	Y	Y	Y	8
(Amr *et al.*, 2018)	Y	N	Y	Y	Y	Y	Y	Y	Y	Y	8
(Motawi *et al.*, 2018)	Y	U	Y	Y	Y	Y	Y	Y	Y	Y	5
(Zhang *et al.*, 2013)	Y	U	Y	Y	Y	N	NA	Y	Y	Y	9
(Abdelaty *et al.*, 2020)	Y	U	Y	Y	Y	N	NA	Y	Y	Y	9
(Zou *et al.*, 2017)	Y	Y	Y	Y	Y	N	NA	Y	Y	Y	10
(Seyhan *et al.*, 2016)	Y	N	Y	Y	Y	Y	Y	Y	Y	Y	8
(Lu *et al.*, 2021)	Y	N	Y	Y	Y	Y	N	Y	Y	Y	5
(Wang *et al.*, 2019b)	Y	Y	U	Y	Y	N	NA	Y	Y	Y	9
(Olivieri *et al.*, 2015)	Y	Y	Y	Y	Y	N	NA	Y	Y	Y	10
(Zhou *et al.*, 2020)	Y	N	Y	Y	Y	Y	N	Y	Y	Y	5

(Seleem *et al.*, 2019)	Y	N	Y	Y	Y	Y	Y	Y	Y	Y	8
(Motawae *et al.*, 2015)	Y	U	U	Y	Y	N	NA	Y	Y	Y	8
(Zhang *et al.*, 2017)	Y	Y	Y	Y	Y	N	NA	Y	Y	Y	10
(Wan *et al.*, 2017)	Y	N	Y	Y	Y	Y	Y	Y	Y	Y	8
(Wang *et al.*, 2016)	Y	N	Y	Y	Y	Y	Y	Y	Y	Y	8
(Rong *et al.*, 2013)	Y	N	Y	Y	Y	Y	Y	Y	Y	Y	8
(Higuchi *et al.*, 2015)	Y	N	U	Y	Y	Y	Y	Y	Y	Y	7
(Fejes *et al.*, 2017)	Y	N	U	Y	Y	Y	Y	Y	Y	Y	7
(Wang *et al.*, 2019a)	Y	Y	U	Y	Y	N	NA	Y	Y	Y	9
(Wan, 2020)	Y	Y	Y	Y	Y	N	NA	Y	Y	Y	10
(Li *et al.*, 2020a)	Y	Y	Y	Y	Y	N	NA	Y	Y	Y	10
(Ma *et al.*, 2016)	Y	N	Y	Y	Y	Y	Y	Y	Y	Y	8
(Akhbari *et al.*, 2018)	Y	N	Y	Y	Y	Y	Y	Y	Y	Y	8
(Sun *et al.*, 2014)	Y	N	Y	Y	Y	Y	Y	Y	Y	Y	8

(Yan *et al.*, 2016)	Y	Y	Y	Y	Y	N	NA	Y	Y	Y	10
(Shaker *et al.*, 2019)	Y	Y	Y	Y	Y	N	NA	Y	Y	Y	10
(Baldeon *et al.*, 2014)	Y	N	Y	Y	Y	Y	Y	Y	Y	Y	8
(Parsa *et al.*, 2020)	Y	Y	Y	Y	Y	N	NA	Y	Y	Y	10
(Li *et al.*, 2020b)	Y	U	Y	Y	Y	N	NA	Y	Y	Y	9
(Nie *et al.*, 2020)	Y	Y	Y	Y	Y	N	NA	Y	Y	Y	10
(Khan *et al.*, 2020)	Y	Y	Y	Y	Y	N	NA	Y	Y	Y	10
(Elemam *et al.*, 2021)	Y	Y	U	Y	Y	N	NA	Y	Y	Y	9
(Prabu *et al.*, 2015)	Y	Y	Y	Y	Y	N	NA	Y	Y	Y	10
(Garcia-Jacobo *et al.*, 2019)	Y	N	Y	Y	Y	Y	Y	Y	Y	Y	8
(Mensa *et al.*, 2019)	Y	N	Y	Y	Y	Y	Y	Y	Y	Y	8
(Monfared *et al.*, 2020)	Y	N	Y	Y	Y	Y	Y	Y	Y	Y	8
(Luo *et al.*, 2020)	Y	Y	Y	Y	Y	N	NA	Y	Y	Y	10
(Luo *et al.*, 2019)	Y	Y	Y	Y	Y	N	NA	Y	Y	Y	10

(Sadeghzadeh *et al.*, 2020)	Y	Y	Y	Y	Y	N	NA	Y	Y	Y	10
(Zeinali *et al.*, 2021)	Y	Y	Y	Y	Y	N	NA	Y	Y	Y	10
(Shao *et al.*, 2017)	Y	Y	Y	Y	Y	N	NA	Y	Y	Y	10
(Lv *et al.*, 2015)	Y	Y	Y	Y	Y	N	NA	Y	Y	Y	10
(Samanian *et al.*, 2019)	Y	Y	Y	Y	Y	N	NA	Y	Y	Y	10
(Alfaifi *et al.*, 2020)	Y	N	Y	Y	Y	Y	N	Y	Y	Y	5
(Prabu *et al.*, 2020)	Y	Y	Y	Y	Y	N	NA	Y	Y	Y	10
(Olivieri *et al.*, 2014)	Y	N	Y	Y	Y	Y	N	Y	Y	Y	5
(La Sala *et al.*, 2019)	Y	N	Y	Y	Y	Y	Y	Y	Y	Y	8
(Al-Hayali *et al.*, 2019)	Y	N	Y	Y	Y	Y	Y	Y	Y	Y	8
(Huang *et al.*, 2018)	Y	Y	Y	Y	Y	N	NA	Y	Y	Y	10
(Liang *et al.*, 2018)	Y	N	Y	Y	Y	Y	Y	Y	Y	Y	8
(Yang *et al.*, 2016)	Y	N	Y	Y	Y	Y	N	Y	Y	Y	5
(Dias, 2016)	Y	Y	Y	Y	Y	N	NA	Y	Y	Y	10

(ESPINOZA, 2018)	Y	N	Y	Y	Y	Y	N	Y	Y	Y	5
(Abdel-Tawab *et al.*, 2023)	Y	N	Y	Y	Y	Y	Y	Y	Y	Y	8
(Abdou *et al.*, 2022)	Y	N	Y	Y	Y	Y	N	Y	Y	Y	5
(Aghaei Zarch *et al.*, 2024)	Y	U	Y	Y	Y	N	NA	Y	Y	Y	9
(Aljaibeji *et al.*, 2022)	Y	N	Y	Y	Y	Y	Y	Y	Y	Y	8
(Banerjee *et al.*, 2022)	Y	N	Y	Y	Y	Y	N	Y	Y	Y	5
(Del Cuore *et al.*, 2023)	Y	Y	Y	Y	Y	N	NA	Y	Y	Y	10
(Dzung *et al.*, 2023)	Y	Y	Y	Y	Y	N	NA	Y	Y	Y	10
(Ezzat *et al.*, 2023)	Y	N	Y	Y	Y	Y	N	Y	Y	Y	5
(Ghoreishi *et al.*, 2022)	Y	Y	Y	Y	Y	N	NA	Y	Y	Y	10
(Greco *et al.*, 2023)	Y	Y	Y	Y	Y	N	NA	Y	Y	Y	10
(Guo *et al.*, 2023)	Y	Y	Y	Y	Y	N	NA	Y	Y	Y	10
(Hu *et al.*, 2022)	Y	N	Y	Y	Y	Y	Y	Y	Y	Y	8
(Kong *et al.*, 2022)	Y	N	Y	Y	Y	Y	N	Y	Y	Y	5

(Lin *et al.*, 2021)	Y	Y	Y	Y	Y	N	NA	Y	Y	Y	10
(Liu *et al.*, 2022a)	Y	U	Y	Y	Y	N	NA	Y	Y	Y	9
(Liu *et al.*, 2022b)	Y	Y	Y	Y	Y	N	NA	Y	Y	Y	10
(Meng *et al.*, 2023)	Y	Y	Y	Y	Y	N	NA	Y	Y	Y	10
(Mokhtari Ardekani *et al.*, 2023)	U	Y	Y	Y	Y	N	NA	Y	Y	Y	9
(Monfared *et al.*, 2022)	Y	N	Y	Y	Y	Y	N	Y	Y	Y	5
(Nemecz *et al.*, 2023)	Y	N	Y	Y	Y	Y	N	Y	Y	Y	5
(Pan *et al.*, 2022)	Y	N	Y	Y	Y	Y	N	Y	Y	Y	5
(Ruan *et al.*, 2023)	Y	Y	Y	Y	Y	N	NA	Y	Y	Y	10
(Saleh *et al.*, 2022)	Y	Y	Y	Y	Y	N	NA	Y	Y	Y	10
(Ghaneh *et al.*, 2023)	Y	Y	Y	Y	Y	N	NA	Y	Y	Y	10
(Shahouzehi *et al.*, 2021)	Y	Y	Y	Y	Y	N	NA	Y	Y	Y	10
(Simoniene *et al.*, 2022)	Y	N	U	Y	Y	Y	Y	Y	Y	Y	7
(Su *et al.*, 2022)	Y	U	Y	Y	Y	N	NA	Y	Y	Y	9

(Sun *et al.*, 2023)	Y	N	Y	Y	Y	Y	N	Y	Y	Y	5
(Sun *et al.*, 2022)	Y	Y	Y	Y	Y	N	NA	Y	Y	Y	10
(Tonyan *et al.*, 2023)	Y	N	Y	Y	Y	Y	Y	Y	Y	Y	8
(Tursinawati *et al.*, 2022)	Y	N	Y	Y	Y	Y	Y	Y	Y	Y	8
(Wu *et al.*, 2021a)	Y	U	Y	Y	Y	N	NA	Y	Y	Y	9
(Wu *et al.*, 2021b)	Y	N	Y	Y	Y	Y	N	Y	Y	Y	5
(Yazdanpanah *et al.*, 2022)	Y	U	Y	Y	Y	N	NA	Y	Y	Y	9
(Yun *et al.*, 2022)	Y	Y	Y	Y	Y	N	NA	Y	Y	Y	10
(Zhao *et al.*, 2023)	Y	U	Y	Y	Y	N	NA	Y	Y	Y	9

Supplementary material 11: Risk of bias heatmap based on the JBI Critical Appraisal Checklist for Case Control Studies applied to the studies included in the type 2 diabetes mellitus systematic review.

JBI Critical Appraisal Checklist for Case Control Studies - Type 2 diabetes mellitus systematic review

Y Yes; N No; U Unclear; NA Not applicable

Study ID	D1	D2	D3	D4	D5	D6	D7	D8	D9	D10
(de Candia *et al.*, 2017)	Y	Y	Y	Y	Y	N	NA	Y	Y	Y
(Baldeon Rojas *et al.*, 2016)	Y	N	Y	Y	Y	Y	Y	Y	Y	Y
(Kong *et al.*, 2011)	Y	Y	Y	Y	Y	N	NA	Y	Y	Y
(Yang *et al.*, 2014)	Y	U	Y	Y	Y	N	NA	Y	Y	Y
(Yang *et al.*, 2017)	Y	U	Y	Y	Y	N	NA	Y	Y	Y
(Pescador *et al.*, 2013)	Y	N	Y	Y	Y	Y	N	Y	Y	Y
(Alipoor *et al.*, 2018)	Y	Y	U	Y	Y	N	NA	Y	Y	Y
(Li *et al.*, 2016)	Y	N	Y	Y	Y	Y	Y	Y	Y	Y
(Rezk *et al.*, 2016)	Y	Y	Y	Y	Y	N	NA	Y	Y	Y
(Sucharita *et al.*, 2018)	Y	N	Y	Y	Y	Y	Y	Y	Y	Y
(Liu *et al.*, 2014)	Y	Y	Y	Y	Y	N	NA	Y	Y	Y
(Ali Beg *et al.*, 2020)	Y	U	Y	Y	Y	N	NA	Y	Y	Y
(Wang *et al.*, 2018)	Y	U	Y	Y	Y	N	NA	Y	Y	Y
(Wu *et al.* 2015)	Y	U	Y	Y	Y	U	U	Y	Y	Y
(Ortega *et al.*, 2014)	Y	N	Y	Y	Y	Y	Y	Y	Y	Y
(Amr *et al.*, 2018)	Y	N	Y	Y	Y	Y	Y	Y	Y	Y
(Motawi *et al.*, 2018)	Y	U	Y	Y	Y	N	NA	Y	Y	Y
(Zhang *et al.*, 2013)	Y	U	Y	Y	Y	N	NA	Y	Y	Y
(Abdelaty *et al.*, 2020)	Y	U	Y	Y	Y	N	NA	Y	Y	Y
(Zou *et al.*, 2017)	Y	Y	Y	Y	Y	N	NA	Y	Y	Y
(Seyhan *et al.*, 2016)	Y	N	Y	Y	Y	Y	Y	Y	Y	Y
(Lu *et al.*, 2021)	Y	N	Y	Y	Y	Y	N	Y	Y	Y
(Wang *et al.* 2019b)	Y	Y	U	Y	Y	N	NA	Y	Y	Y
(Olivieri *et al.*, 2015)	Y	Y	Y	Y	Y	N	NA	Y	Y	Y
(Zhou *et al.*, 2020)	Y	N	Y	Y	Y	Y	N	Y	Y	Y
(Seleem *et al.*, 2019)	Y	N	Y	Y	Y	Y	Y	Y	Y	Y

Study ID	D1	D2	D3	D4	D5	D6	D7	D8	D9	D10
(Motawae *et al.*, 2015)	Y	U	U	Y	Y	N	NA	Y	Y	Y
(Zhang *et al.*, 2017)	Y	Y	Y	Y	Y	N	NA	Y	Y	Y
(Wan *et al.*, 2017)	Y	N	Y	Y	Y	Y	Y	Y	Y	Y
(Wang *et al.*, 2016)	Y	N	Y	Y	Y	Y	Y	Y	Y	Y
(Rong *et al.*, 2013)	Y	N	Y	Y	Y	Y	Y	Y	Y	Y
(Higuchi *et al.*, 2015)	Y	N	U	Y	Y	Y	Y	Y	Y	Y
(Fejes *et al.*, 2017)	Y	N	U	Y	Y	Y	Y	Y	Y	Y
(Wang *et al.*, 2019a)	Y	Y	U	Y	Y	N	NA	Y	Y	Y
(Wan, 2020)	Y	Y	Y	Y	Y	N	NA	Y	Y	Y
(Li *et al.*, 2020a)	Y	Y	Y	Y	Y	N	NA	Y	Y	Y
(Ma *et al.*, 2016)	Y	N	Y	Y	Y	Y	Y	Y	Y	Y
(Akhbari *et al.*, 2018)	Y	N	Y	Y	Y	Y	Y	Y	Y	Y
(Sun *et al.*, 2014)	Y	N	Y	Y	Y	Y	Y	Y	Y	Y
(Yan *et al.* 2016)	Y	Y	Y	Y	Y	N	NA	Y	Y	Y
(Shaker *et al.*, 2019)	Y	Y	Y	Y	Y	N	NA	Y	Y	Y
(Baldeon *et al.*, 2014)	Y	N	Y	Y	Y	Y	Y	Y	Y	Y
(Parsa *et al.*, 2020)	Y	Y	Y	Y	Y	N	NA	Y	Y	Y
(Li *et al.*, 2020b)	Y	U	Y	Y	Y	N	NA	Y	Y	Y
(Nie *et al.*, 2020)	Y	Y	Y	Y	Y	N	NA	Y	Y	Y
(Khan *et al.*, 2020)	Y	Y	Y	Y	Y	N	NA	Y	Y	Y
(Elemam, 2021)	Y	Y	U	Y	Y	N	NA	Y	Y	Y
(Prabu *et al.*, 2015)	Y	Y	Y	Y	Y	N	NA	Y	Y	Y
(Garcia-Jacobo *et al.* 2019)	Y	N	Y	Y	Y	Y	Y	Y	Y	Y
(Mensa *et al.*, 2019)	Y	N	Y	Y	Y	Y	Y	Y	Y	Y
(Monfared *et al.*, 2020)	Y	N	Y	Y	Y	Y	Y	Y	Y	Y
(Luo *et al.*, 2020)	Y	Y	Y	Y	Y	N	NA	Y	Y	Y

JBI Critical Appraisal Checklist for Case Control Studies - Type 2 diabetes mellitus systematic review

Y Yes; N No; U Unclear; NA Not applicable

Study ID	D1	D2	D3	D4	D5	D6	D7	D8	D9	D10
(Luo *et al.*, 2019)	Y	Y	Y	Y	Y	N	NA	Y	Y	Y
(Sadeghzadeh *et al.*, 2020)	Y	Y	Y	Y	Y	N	NA	Y	Y	Y
(Zeinali *et al.*, 2021)	Y	Y	Y	Y	Y	N	NA	Y	Y	Y
(Shao *et al.*, 2017)	Y	Y	Y	Y	Y	N	NA	Y	Y	Y
(Lv *et al.*, 2015)	Y	Y	Y	Y	Y	N	NA	Y	Y	Y
(Samanian *et al.*, 2019)	Y	Y	Y	Y	Y	N	NA	Y	Y	Y
(Alfaifi *et al.*, 2020)	Y	N	Y	Y	Y	Y	N	Y	Y	Y
(Prabu *et al.*, 2020)	Y	Y	Y	Y	Y	N	NA	Y	Y	Y
(Olivieri *et al.*, 2014)	Y	N	Y	Y	Y	Y	N	Y	Y	Y
(La Sala *et al.*, 2019)	Y	N	Y	Y	Y	Y	Y	Y	Y	Y
(Al-Hayali *et al.*, 2019)	Y	N	Y	Y	Y	Y	Y	Y	Y	Y
(Huang *et al.*, 2018)	Y	Y	Y	Y	Y	N	NA	Y	Y	Y
(Liang *et al.*, 2018)	Y	N	Y	Y	Y	Y	Y	Y	Y	Y
(Yang *et al.*, 2016)	Y	N	Y	Y	Y	Y	N	Y	Y	Y
(Dias, 2016)	Y	Y	Y	Y	Y	N	NA	Y	Y	Y
(Espinoza, 2018)	Y	N	Y	Y	Y	Y	N	Y	Y	Y
(Abdel-Tawab *et al.*, 2023)	Y	N	Y	Y	Y	Y	Y	Y	Y	Y
(Abdou *et al.*, 2022)	Y	N	Y	Y	Y	Y	N	Y	Y	Y
(Aghaei Zarch *et al.*, 2024)	Y	U	Y	Y	Y	N	NA	Y	Y	Y
(Aljaibeji *et al.*, 2022)	Y	N	Y	Y	Y	Y	Y	Y	Y	Y
(Banerjee *et al.*, 2022)	Y	N	Y	Y	Y	Y	N	Y	Y	Y
(Del Cuore *et al.*, 2023)	Y	Y	Y	Y	Y	N	NA	Y	Y	Y
(Dzung *et al.*, 2023)	Y	Y	Y	Y	Y	N	NA	Y	Y	Y
(Ezzat *et al.* 2023)	Y	N	Y	Y	Y	Y	N	Y	Y	Y
(Ghoreishi *et al.*, 2022)	Y	Y	Y	Y	Y	N	NA	Y	Y	Y
(Greco *et al.*, 2023)	Y	Y	Y	Y	Y	N	NA	Y	Y	Y

Study ID	D1	D2	D3	D4	D5	D6	D7	D8	D9	D10
(Guo *et al.*, 2023)	Y	Y	Y	Y	Y	N	NA	Y	Y	Y
(Hu *et al.*, 2022)	Y	N	Y	Y	Y	Y	Y	Y	Y	Y
(Kong *et al.*, 2022)	Y	N	Y	Y	Y	Y	N	Y	Y	Y
(Lin *et al.*, 2021)	Y	Y	Y	Y	Y	N	NA	Y	Y	Y
(Liu *et al.*, 2022a)	Y	U	Y	Y	Y	N	NA	Y	Y	Y
(Liu *et al.*, 2022b)	Y	Y	Y	Y	Y	N	NA	Y	Y	Y
(Meng *et al.*, 2023)	Y	Y	Y	Y	Y	N	NA	Y	Y	Y
(Mokhtari Ardekani *et al.*, 2023)	U	Y	Y	Y	Y	N	NA	Y	Y	Y
(Monfared *et al.*, 2022)	Y	N	Y	Y	Y	Y	N	Y	Y	Y
(Nemecz *et al.*, 2023)	Y	N	Y	Y	Y	Y	N	Y	Y	Y
(Pan *et al.*, 2022)	Y	N	Y	Y	Y	Y	N	Y	Y	Y
(Ruan *et al.*, 2023)	Y	Y	Y	Y	Y	N	NA	Y	Y	Y
(Saleh *et al.*, 2022)	Y	Y	Y	Y	Y	N	NA	Y	Y	Y
(Ghaneh *et al.*, 2023)	Y	Y	Y	Y	Y	N	NA	Y	Y	Y
(Shahouzehi *et al.* 2021)	Y	Y	Y	Y	Y	N	NA	Y	Y	Y
(Simoniene *et al.*, 2022)	Y	N	U	Y	Y	Y	Y	Y	Y	Y
(Su *et al.*, 2022)	Y	U	Y	Y	Y	N	NA	Y	Y	Y
(Sun *et al.*, 2023)	Y	N	Y	Y	Y	Y	N	Y	Y	Y
(Sun *et al.*, 2022)	Y	Y	Y	Y	Y	N	NA	Y	Y	Y
(Tonyan *et al.*, 2023)	Y	N	Y	Y	Y	Y	Y	Y	Y	Y
(Tursinawati *et al.*, 2022)	Y	N	Y	Y	Y	Y	Y	Y	Y	Y
(Wu *et al.*, , 2021a)	Y	U	Y	Y	Y	N	NA	Y	Y	Y
(Wu *et al.*, 2021b)	Y	N	Y	Y	Y	Y	N	Y	Y	Y
(Yazdanpanah *et al.* 2022)	Y	U	Y	Y	Y	N	NA	Y	Y	Y
(Yun *et al.*, 2022)	Y	Y	Y	Y	Y	N	NA	Y	Y	Y
(Zhao *et al.*, 2023)	Y	U	Y	Y	Y	N	NA	Y	Y	Y

Supplementary material 12: miRNAs differentially expressed in type 2 diabetes mellitus patients compared to controls.

Study identification	Sample size		Sample type	miRNA quantification method	miRNAs	Up or down-regulated? T2DM X control	*Fold change*	p value
	Control	Case						
(de Candia *et al.*, 2017)	9	9	Plasma	RT-qPCR	miR-148a-3p<br>miR-222-3p<br>miR-342-3p<br>let-7d-3p<br>miR-18a-5p<br>miR-18b-5p<br>miR-23a-3p<br>miR-24-3p<br>miR-27a-3p<br>miR-28-3p<br>miR-30d-5p<br>miR-122-5p<br>miR-99a-5p<br>miR-143-3p<br>miR-320b<br>miR-320c	miR-148a-3p: up<br>miR-222-3p: down<br>miR-342-3p: down<br>let-7d-3p: NS<br>miR-18a-5p: NS<br>miR-18b-5p: NS<br>miR-23a-3p: NS<br>miR-24-3p: NS<br>miR-27a-3p: NS<br>miR-28-3p: NS<br>miR-30d-5p: NS<br>miR-122-5p: NS<br>miR-99a-5p: NS<br>miR-143-3p: NS<br>miR-320b: NS<br>miR-320c: NS	NI	miR-148a-3p: p<0.05<br>miR-222-3p: p< 0.05<br>miR-342-3p: p<0.01<br>let-7d-3p: NS<br>miR-18a-5p: NS<br>miR-18b-5p: NS<br>miR-23a-3p: NS<br>miR-24-3p: NS<br>miR-27a-3p: NS<br>miR-28-3p: NS<br>miR-30d-5p: NS<br>miR-122-5p: NS<br>miR-99a-5p: NS<br>miR-143-3p: NS<br>miR-320b: NS<br>miR-320c: NS
(Baldeon Rojas *et al.*, 2016)	44	64	Serum	RT-qPCR	miR-122<br>miR-138<br>miR-146a<br>miR-155<br>miR-34c-5p<br>miR-410<br>miR-574-3p<br>miR-576-3p	miR-122: NS<br>miR-138: NS<br>miR-146a: down<br>miR-155: NS<br>miR-34c-5p: NS<br>miR-410: NS<br>miR-574-3p: down<br>miR-576-3p: NS	miR-122:0.86<br>miR-138: 0.87<br>miR-146a: 0.71<br>miR-155: 0.95<br>miR-34c-5p: 1.00<br>miR-410: 0.92<br>miR-574-3p: 0.69<br>miR-576-3p: 1.43	miR-122: NS<br>miR-138: NS<br>miR-146a: p=0.02<br>miR-155: NS<br>miR-34c-5p: NS<br>miR-410: NS<br>miR-574-3p: p=0.03<br>miR-576-3p: NS
(Kong *et al.*, 2011)	19	18	Serum	RT-qPCR	miR-9<br>miR-29a<br>miR-30d<br>miR-34a<br>miR-124a<br>miR-146a<br>miR-375	miR-9: up<br>miR-29a: up<br>miR-30d: up<br>miR-34a: up<br>miR-124a: up<br>miR-146a: up<br>miR-375: up	NI	miR-9: p=0.0410<br>miR-29a: p=0.0030<br>miR-30d: p=0.0180<br>miR-34a: p=0.0000<br>miR-124a: p=0.0230<br>miR-146a: p=0.0080<br>miR-375: p=0.0040

(Yang *et al.*, 2014)	20	24	Serum	RT-qPCR	miR-23a<br>let-7i<br>miR-486<br>miR-96<br>miR-186<br>miR-191<br>miR 192<br>miR-146a	miR-23a: down<br>let-7i: down<br>miR-486: down<br>miR-96: down<br>miR-186: down<br>miR-191: down<br>miR 192: down<br>miR-146a: down	NI	miR-23a: p<0.001<br>let-7i: p<0.01<br>miR-486: p<0.05<br>miR-96: p<0.05<br>miR-186: p<0.05<br>miR-191: p<0.05<br>miR 192: p<0.05<br>miR-146a: p<0.05
(Yang *et al.*, 2017)	5	10	Serum	RT-qPCR	miR-409-3p<br>miR-665<br>miR-766-3p<br>miR-455-5p<br>miR-454-3p<br>miR-144-3p<br>miR-96-5p	miR-409-3p: down<br>miR-665: down<br>miR-766-3p: down<br>miR-455-5p: up<br>miR-454-3p: up<br>miR-144-3p: up<br>miR-96-5p: up	NI	miR-409-3p: p<0.05<br>miR-665: p<0.05<br>miR-766-3p: p<0.05<br>miR-455-5p: p<0.05<br>miR-454-3p: p<0.05<br>miR-144-3p: p<0.05<br>miR-96-5p: p<0.05
(Pescador *et al.*, 2013)	20	13	Serum	RT-qPCR	miR-138<br>miR-376a<br>miR-15b<br>miR-503	miR-138: NS<br>miR-376a: NS<br>miR-15b: NS<br>miR-503: down	NI	miR-138: NS<br>miR-376a: NS<br>miR-15b: NS<br>miR-503: p<0.0001
(Alipoor *et al.*, 2018)	30	30	Plasma	RT-qPCR	miR-146a	down	NI	p<0.05
(Li *et al.*, 2016)	20	30	Serum	RT-qPCR	miR-221<br>miR-222	miR-221: up<br>miR-222: up	NI	miR-221: p<0.05<br>miR-222: p<0.05
(Rezk *et al.*, 2016)	100	100	Serum	RT-qPCR	miR-126	down	NI	p<0.001
(Sucharita *et al.*, 2018)	30	30	Plasma	RT-qPCR	miR-9<br>miR-30d<br>miR-1<br>miR-133a<br>miR-29a<br>miR-143	miR-9: NS<br>miR-30d: up<br>miR-1: NS<br>miR-133a: NS<br>miR-29a: NS<br>miR-143: NS	NI	miR9: NS<br>miR30d: p<0.01<br>miR1: NS<br>miR133a: NS<br>miR29a: NS<br>miR143: NS
(Liu *et al.*, 2014)	138	160	Serum	RT-qPCR	miR-126	down	NI	p<0.001
(Ali Beg *et al.*, 2020)	100	100	Serum	RT-qPCR	miR-330	up	7.72	NI

(Wang *et al.*, 2018)	20	36	Serum	RT-qPCR	miR-199a-3p	down	NI	p<0.05
(Wu *et al.*, 2015)	25	25	Serum	RT-qPCR	miR-152<br>miR-17<br>miR-593<br>miR-138	miR-152: NS<br>miR-17: NS<br>miR-593: down<br>miR-138: NS	NI	miR-152: NS<br>miR-17: NS<br>miR-593: p<0.001<br>miR-138: NS
(Ortega *et al.*, 2014)	35	30	Plasma	RT-qPCR	miR-140-5p<br>miR-142-3p<br>miR-222<br>miR-125b<br>miR-126a<br>miR-130b<br>miR-192<br>miR-195<br>miR-423-5p<br>miR-532-5p	miR-140-5p: up<br>miR-142-3p: up<br>miR-222: up<br>miR-125b: down<br>miR-126a: down<br>miR-130b: down<br>miR-192: down<br>miR-195: down<br>miR-423-5p: down<br>miR-532-5p: down	NI	miR-140-5p: P<0.0001<br>miR-142-3p: P<0.0001<br>miR-222: P<0.0001<br>miR-125b: P<0.0001<br>miR-126a: P=0.02<br>miR-130b: P=0.002<br>miR-192: P<0.0001<br>miR-195: P=0.001<br>miR-423-5p: P<0.0001<br>miR-532-5p: P=0.005
(Amr *et al.*, 2018)	20	54	Plasma	RT-qPCR	miR-126<br>miR-210	miR-126: down<br>miR-210: up	miR-126: 2.8<br>miR-210: 5.3	miR-126: p<0.001<br>miR-210: p<0.001
(Motawi *et al.*, 2018)	25	25	Serum	RT-qPCR	miR-130b	down	NI	p<0.05
(Zhang *et al.*, 2013)	30	30	Plasma	RT-qPCR	miR-15a<br>miR-223<br>miR-126	miR-15a: NS<br>miR-223: NS<br>miR-126: down	NI	miR-15a: NS<br>miR-223: NS<br>miR-126: p<0.05
(Abdelaty *et al.*, 2020)	25	20	Plasma	RT-qPCR	miR-192	up	NI	p<0.001
(Zou *et al.*, 2017)	127	65	Plasma	RT-qPCR	miR-93	up	NI	p<0.05
(Seyhan *et al.*, 2016)	27	31	Plasma	RT-qPCR	miR-126<br>miR-146a<br>miR-148a<br>miR-21<br>miR-24<br>miR-29a<br>miR-30d<br>miR-34a	miR-126: NS<br>miR-146a: NS<br>miR-148a: up<br>miR-21: up<br>miR-24: NS<br>miR-29a: NS<br>miR-30d: up<br>miR-34a: up	miR-126: NS<br>miR-146a: NS<br>miR-148a: 1.96<br>miR-21: 1.67<br>miR-24: NS<br>miR-29a: NS<br>miR-30d: 2.66<br>miR-34a: 3.30	miR-126: NS<br>miR-146a: NS<br>miR-148a: 0.0481<br>miR-21: 0.0301<br>miR-24: NS<br>miR-29a: NS<br>miR-30d: 0.0034<br>miR-34a: 0.0184

					miR-375<br>miR-376a	miR-375: NS<br>miR-376a: NS	miR-375: NS<br>miR-376a: NS	miR-375: NS<br>miR-376a: NS
(Lu *et al.*, 2021)	50	50	Serum	RT-qPCR	miR-720	up	NI	p<0.01
(Wang *et al.*, 2019b)	68	69	Serum	RT-qPCR	miR-92a	up	NI	p<0.05
(Olivieri *et al.*, 2015)	107	76	Plasma	RT-qPCR	miR-126-3p<br>miR-21-5p	miR-126-3p: down<br>miR-21-5p: down	NI	miR-126-3p: p<0.05<br>miR-21-5p: p<0.05
(Zhou *et al.*, 2020)	44	49	Serum	RT-qPCR	miR-18a	down	NI	p<0.05
(Seleem *et al.*, 2019)	50	50	Serum	RT-qPCR	miR-342<br>miR-450	miR-342: up<br>miR-450: down	NI	miR-342: p<0.05<br>miR-450: p<0.05
(Motawae *et al.*, 2015)	50	50	Serum	RT-qPCR	miR-9<br>miR-370	miR-9: up<br>miR-370: up	NI	miR-9: up: p=0.000<br>miR-370: p=0.000
(Zhang *et al.*, 2017)	79	93	Plasma	RT-qPCR	miR-126	down	NI	0.001
(Wan *et al.*, 2017)	74	76	Serum	RT-qPCR	miR-7	up	NI	p<0.001
(Wang *et al.*, 2016)	92	92	Serum	RT-qPCR	miR-571<br>miR-661<br>miR-770-5p<br>miR-892b<br>miR-1303	miR-571: up<br>miR-661: up<br>miR-770-5p: up<br>miR-892b: up<br>miR-1303: up	NI	miR-571: p<0.01<br>miR-661: p<0.001<br>miR-770-5p: p<0.01<br>miR-892b: p<0.01<br>miR-1303: p<0.05
(Rong *et al.*, 2013)	90	90	Plasma	RT-qPCR	miR-146a	up	NI	p<0.05
(Higuchi *et al.*, 2015)	49	155	Serum	RT-qPCR	miR-101<br>miR-375<br>miR-802	miR-101: up<br>miR-375: up<br>miR-802: up	NI	miR-101: p<0.01<br>miR-375: p<0.01<br>miR-802: p<0.05
(Fejes *et al.*, 2017)	23	28	Plasma	RT-qPCR	miR-223<br>miR-26b<br>miR-140<br>miR-126	miR-223: down<br>miR-26b: down<br>miR-140: down<br>miR-126: down	NI	miR-223: 0.048<br>miR-26b: p=0.006<br>miR-140: p<0.001<br>miR-126p<0.001
(Wang *et al.*, 2019a)	40	40	Plasma	RT-qPCR	miR-155-5p<br>miR-150-5p<br>miR-30e<br>miR-3196	miR-155-5p: NS<br>miR-150-5p: down<br>miR-30e: down<br>miR-3196: down	NI	miR-155-5p: NS<br>miR-150-5p: p=0.001<br>miR-30e: p=0.002<br>miR-3196: p=0.014

					miR-320<br>let-7a-5p	miR-320: down<br>let-7a-5p: NS		miR-320: p=0.002<br>let-7a-5p: NS
(Wan, 2020)	100	102	Serum	RT-qPCR	miR-409-5p<br>miR-216a	miR-409-5p: up<br>miR-216a: down	NI	miR-409-5p: p<0.05<br>miR-216a: p<0.05
(Li *et al.*, 2020a)	32	32	Plasma	RT-qPCR	miR-20	up	NI	p<0.001
(Ma *et al.*, 2016)	127	157	Serum	RT-qPCR	miR-192	NS	NS	NS
(Akhbari *et al.*, 2018)	22	21	Serum	RT-qPCR	miR-93	down	NI	p=0.02
(Sun *et al.*, 2014)	100	100	Plasma	RT-qPCR	miR-375	up	NI	p=0.0313
(Yan *et al.*, 2016)	50	50	Plasma	RT-qPCR	miR-1249<br>miR-320b<br>miR-572<br>miR-6069	miR-1249: down<br>miR-320b: down<br>miR-572: up<br>miR-6069: NS	NI	miR-1249: P<0.0001<br>miR-320b: P<0.0001<br>miR-572: P<0.0001<br>miR-6069: NS
(Shaker *et al.*, 2019)	81	30	Serum	RT-qPCR	miR-20b<br>miR-17-3p	miR-20b: down<br>miR-17-3p: NS	miR-20b: 0.17<br>miR-17-3p: NS	miR-20b: p<0.0001<br>miR-17-3p: NS
(Baldeon *et al.*, 2014)	40	56	Serum	RT-qPCR	miR-146a<br>miR-155	miR-146a: down<br>miR-155: NS	NI	miR-146a: 0.04<br>miR-155: NS
(Parsa *et al.*, 2020)	40	50	Plasma	RT-qPCR	miR-124a	down	NI	p=0.028
(Li *et al.*, 2020b)	60	60	Plasma	RT-qPCR	miR-24	down	NI	p<0.05
(Nie *et al.*, 2020)	80	80	Plasma	RT-qPCR	miR-126<br>miR-28- 3p	miR-126: up<br>miR-28- 3p: up	NI	miR-126: p<0.05<br>miR-28- 3p: p<0.05
(Khan *et al.*, 2020)	5	5	Serum	RT-qPCR	miR-98-5p<br>miR-423-3p<br>miR-6131<br>miR-1246<br>miR-99b-5p<br>miR-1304-5p<br>miR-302d-3p	miR-98-5p: down<br>miR-423-3p: down<br>miR-6131: down<br>miR-1246: down<br>miR-99b-5p: up<br>miR-1304-5p: up<br>miR-302d-3p: up	NI	miR-98-5p: p<0.001<br>miR-423-3p: p<0.001<br>miR-6131: p<0.001<br>miR-1246: p<0.001<br>miR-99b-5p: p<0.01<br>miR-1304-5p: p<0.001<br>miR-302d-3p: p<0.01
(Elemam *et al.*, 2021)	50	50	Serum	RT-qPCR	miR-421<br>miR-3909	miR-421: down<br>miR-3909: NS	NI	miR-421: p<0.05<br>miR-3909: NS

					miR-212-5p<br>miR-4677-3p	miR-212-5p: NS<br>miR-4677-3p: NS		miR-212-5p: NS<br>miR-4677-3p: NS
(Prabu *et al.*, 2015)	49	49	Serum	RT-qPCR	miR-128<br>miR-99b-5p<br>miR-130b-3p<br>miR-142-3p<br>miR-374a-5p<br>miR-423-5p<br>miR-484<br>miR-629-5p<br>let-7d-3p	miR-128: NS<br>miR-99b-5p: NS<br>miR-130b-3p: up<br>miR-142-3p: NS<br>miR-374a-5p: up<br>miR-423-5p: NS<br>miR-484: NS<br>miR-629-5p: NS<br>let-7d-3p: NS	NI	miR-128: NS<br>miR-99b-5p: NS<br>miR-130b-3p: p<0.05<br>miR-142-3p: NS<br>miR-374a-5p: p<0.05<br>miR-423-5p: NS<br>miR-484: NS<br>miR-629-5p: NS<br>let-7d-3p: NS
(Garcia-Jacobo *et al.*, 2019)	35	54	Serum	RT-qPCR	miR-146a<br>miR-34a<br>miR-375	miR-146a: NS<br>miR-34a: NS<br>miR-375: NS	NI	miR-146a: NS<br>miR-34a: NS<br>miR-375: NS
(Mensa *et al.*, 2019)	188	144	Plasma	RT-qPCR	miR-146a	NS	NS	NS
(Monfared *et al.*, 2020)	40	40	Plasma	RT-qPCR	miR-135a	up	NI	p<0.05
(Luo *et al.*, 2020)	50	48	Plasma	RT-qPCR	miR-103a<br>miR-103b	miR-103a: up<br>miR-103b: down	NI	miR-103a: p<0.01<br>miR-103b: p<0.01
(Luo *et al.*, 2019)	32	47	Plasma	RT-qPCR	miR-30c	down	NI	p<0.001
(Sadeghzadeh *et al.*, 2020)	30	30	Plasma	RT-qPCR	miR-15a<br>miR-222	miR-15a: down<br>miR-222: up	NI	miR-15a: p<0.001<br>miR-222: p<0.001
(Zeinali *et al.*, 2021)	30	30	Plasma	RT-qPCR	miR-122<br>miR-126-3p<br>miR-146a	miR-122: up<br>miR-126-3p: down<br>miR-146a: down	NI	miR-122: p<0.001<br>miR-126-3p: p<0.001<br>miR-146a: p<0.001
(Shao *et al.*, 2017)	195	186	Serum	RT-qPCR	miR-217	up	NI	p<0.01
(Lv *et al.*, 2015)	131	137	Serum	RT-qPCR	miR-130b	down	NI	p<0.001
(Samanian *et al.*, 2019)	20	20	Plasma	RT-qPCR	miR-126<br>miR-15a-3p	miR-126: down<br>miR-15a-3p: down	NI	miR-126: p<0.001<br>miR-15a-3p: p<0.001
(Alfaifi *et al.*, 2020)	200	200	Serum	RT-qPCR	miR-29a<br>miR-29b	miR-29a: up<br>miR-29b: up	miR-29a: 5.62<br>miR-29b: 5.58	NI

(Prabu *et al.*, 2020)	40	40	Serum	RT-qPCR	miR-128	up	NI	p<0.05
(Olivieri *et al.*, 2014)	92	193	Plasma	RT-qPCR	miR-126	down	NI	p<0.01
(La Sala *et al.*, 2019)	44	27	Plasma	RT-qPCR	miR-21	up	NI	p=0.0173
(Al-Hayali *et al.*, 2019)	45	45	Serum	RT-qPCR	miR-1<br>miR-21	miR-1: down<br>miR-21: NS	miR-1: 0.54<br>miR-21: NS	miR-1: p<0.001<br>miR-21: NS
(Huang *et al.*, 2018)	24	24	Serum	RT-qPCR	miR-122	NS	NS	NS
(Liang *et al.*, 2018)	94	112	Plasma	RT-qPCR	let-7b<br>miR-142<br>miR-144<br>miR-29a	let-7b: up<br>miR-142: down<br>miR-144: up<br>miR-29a: up	NI	let-7b: p<0.01<br>miR-142: p<0.01<br>miR-144: p<0.01<br>miR-29a: p<0.01
(Yang *et al.*, 2016)	30	58	Plasma	RT-qPCR	miR-144<br>miR-223	miR-144: up<br>miR-223: down	NI	miR-144: p<0.05<br>miR-223: p<0.05
(Dias, 2016)	4	4	Serum	RT-qPCR	miR-27b<br>miR-143<br>miR-21<br>miR-98<br>miR-379<br>MYNO59<br>MYNO95<br>MYNO66<br>MYNO8<br>MYNO22	miR-27b: NS<br>miR-143: NS<br>miR-21: NS<br>miR-98: NS<br>miR-379: NS<br>MYNO59: down<br>MYNO95: down<br>MYNO66: down<br>MYNO8: NS<br>MYNO22: NS	NI	miR-27b: NS<br>miR-143: NS<br>miR-21: NS<br>miR-98: NS<br>miR-379: NS<br>MYNO59: p<0.05<br>MYNO95: p<0.05<br>MYNO66: p<0.01<br>MYNO8: NS<br>MYNO22: NS
(ESPINOZA, 2018)	16	17	Serum	RT-qPCR	miR-126-3p<br>miR-375	miR-126-3p: NS<br>miR-375: NS	NI	miR-126-3p: NS<br>miR-375: :NS
(Abdel-Tawab *et al.*, 2023)	50	50	Serum	RT-qPCR	mir-221	up	NI	p<0.001
(Abdou *et al.*, 2022)	20	20	Serum	RT-qPCR	miR-152-3p	up	NI	p = 0.029
(Aghaei Zarch *et al.*, 2024)	50	50	Serum	RT-qPCR	miR-9<br>miR-214	miR-9: down<br>miR-214: down	NI	miR-9: p<0.0001<br>miR-214: p<0.0001
(Aljaibeji *et al.*, 2022)	25	29	Serum	RT-qPCR	let7b-5p	up	NI	p<0.05

(Banerjee *et al.*, 2022)	30	30	Plasma	RT-qPCR	miR-146a	up	>4	p<0.05
(Del Cuore *et al.*, 2023)	20	40	Serum	RT-qPCR	miR-217-5p<br>miR126-3p<br>miR-503-5p	miR-217-5p: up<br>miR126-3p: NS<br>miR-503-5p: up	miR-217-5p: 2.41<br>miR126-3p: 0.61<br>miR-503-5p: 2.24	miR-217-5p: 0<br>miR126-3p: NS<br>miR-503-5p: 0.029
(Dzung *et al.*, 2023)	93	105	Plasma	RT-qPCR	miR-29a<br>miR-146a<br>miR-147b	miR-29a: up<br>miR-146a: NS<br>miR-147b: up	NI	miR-29a: p<0.0001<br>miR-146a: NS<br>miR-147b: p<0.0001
(Ezzat *et al.*, 2023)	128	129	Plasma	RT-qPCR	miR-375	up	NI	p<0.001
(Ghoreishi *et al.*, 2022)	20	20	Plasma	RT-qPCR	miR-148b-3p<br>miR-27a-3p	miR-148b-3p: up<br>miR-27a-3p: down	NI	miR-148b-3p: p<0.0001<br>miR-27a-3p: p<0.01
(Greco *et al.*, 2023)	10	10	Plasma	RT-qPCR	miR-4301<br>let-7b-5p<br>miR-1287-5p<br>let-7a-5p<br>miR-1913<br>miR-523-5p<br>miR-26b-5p<br>miR-195-5p<br>miR-1587<br>miR-3135b<br>miR-1260a<br>miR-1260b<br>miR-20b-5p<br>miR-1281<br>miR-3131<br>miR-4687-5p<br>miR-3200-5p<br>miR-100-5p<br>let-7c-5p<br>miR-365b-3p<br>let-7f-5p<br>miR-671-3p<br>miR-625-3p<br>miR-4651	miR-4301: up<br>let-7b-5p: up<br>miR-1287-5p: up<br>let-7a-5p: up<br>miR-1913: up<br>miR-523-5p: up<br>miR-26b-5p: up<br>miR-195-5p: up<br>miR-1587: up<br>miR-3135b: up<br>miR-1260a: up<br>miR-1260b: up<br>miR-20b-5p: up<br>miR-1281: up<br>miR-3131: up<br>miR-4687-5p: up<br>miR-3200-5p: up<br>miR-100-5p: up<br>let-7c-5p: up<br>miR-365b-3p: up<br>let-7f-5p: up<br>miR-671-3p: up<br>miR-625-3p: up<br>miR-4651: up	miR-4301: 142.74<br>let-7b-5p: 90.34<br>miR-1287-5p: 50.12<br>let-7a-5p: 48.41<br>miR-1913: 42.73<br>miR-523-5p: 34.47<br>miR-26b-5p: 31.06<br>miR-195-5p: 25.76<br>miR-1587: 21.07<br>miR-3135b: 18.99<br>miR-1260a: 17.23<br>miR-1260b: 16.53<br>miR-20b-5p: 15.97<br>miR-1281: 14.9<br>miR-3131: 14.49<br>miR-4687-5p: 13.24<br>miR-3200-5p: 13.06<br>miR-100-5p: 12.88<br>let-7c-5p: 11.94<br>miR-365b-3p: 11.77	NI

					miR-4505<br>miR-144-3p<br>miR-1237-3p<br>miR-378g<br>miR-1183<br>miR-596<br>miR-1910-5p<br>miR-3141<br>miR-185-5p<br>miR-3610<br>miR-877-3p<br>miR-1207-5p<br>miR-3911<br>miR-99a-5p<br>miR-3183<br>let-7e-5p<br>miR-223-3p<br>miR-373-5p<br>miR-4274<br>miR-370-3p<br>miR-101-3p<br>miR-181c-5p<br>miR-4689<br>miR-142-5p<br>miR-1301-3p<br>miR-106b-5p<br>miR-3907<br>miR-1290<br>miR-877-5p<br>miR-1909-5p<br>miR-4732-5p<br>miR-320e<br>miR-138-1-3p<br>let-7d-5p<br>miR-10a-5p<br>miR-1203	miR-4505: up<br>miR-144-3p: up<br>miR-1237-3p: up<br>miR-378g: up<br>miR-1183: up<br>miR-596: up<br>miR-1910-5p: up<br>miR-3141: up<br>miR-185-5p: up<br>miR-3610: up<br>miR-877-3p: up<br>miR-1207-5p: up<br>miR-3911: up<br>miR-99a-5p: up<br>miR-3183: up<br>let-7e-5p: up<br>miR-223-3p: up<br>miR-373-5p: up<br>miR-4274: up<br>miR-370-3p: up<br>miR-101-3p: up<br>miR-181c-5p: up<br>miR-4689: up<br>miR-142-5p: up<br>miR-1301-3p: up<br>miR-106b-5p: up<br>miR-3907: up<br>miR-1290: up<br>miR-877-5p: up<br>miR-1909-5p: up<br>miR-4732-5p: up<br>miR-320e: up<br>miR-138-1-3p: up<br>let-7d-5p: up<br>miR-10a-5p: up<br>miR-1203: up	let-7f-5p: 11.37<br>miR-671-3p: 11.29<br>miR-625-3p: 11.29<br>miR-4651: 10.76<br>miR-4505: 9.9<br>miR-144-3p: 9.76<br>miR-1237-3p: 9.56<br>miR-378g: 9.36<br>miR-1183: 9.11<br>miR-596: 8.92<br>miR-1910-5p: 8.86<br>miR-3141: 8.44<br>miR-185-5p: 8.15<br>miR-3610: 7.93<br>miR-877-3p: 7.71<br>miR-1207-5p: 7.61<br>miR-3911: 7.55<br>miR-99a-5p: 7.55<br>miR-3183: 7.4<br>let-7e-5p: 7.35<br>miR-223-3p: 7.2<br>miR-373-5p: 6.31<br>miR-4274: 6.18<br>miR-370-3p: 6.05<br>miR-101-3p: 5.93<br>miR-181c-5p: 5.69<br>miR-4689: 5.65<br>miR-142-5p: 5.57<br>miR-1301-3p: 5.53<br>miR-106b-5p: 5.23<br>miR-3907: 4.92<br>miR-1290: 4.81<br>miR-877-5p: 4.71<br>miR-1909-5p: 4.68<br>miR-4732-5p: 4.68<br>miR-320e: 4.52	

					miR-3646<br>miR-34c-3p<br>miR-181c-3p<br>miR-1539<br>miR-3185<br>miR-4688<br>miR-3191-3p<br>miR-4291<br>miR-363-3p<br>miR-219a-1-3p<br>miR-628-3p<br>miR-1225-3p<br>miR-2467-3p<br>miR-375<br>let-7i-5p<br>miR-324-3p<br>miR-27b-3p<br>miR-4516<br>miR-92b-3p<br>miR-675-3p<br>miR-490-3p<br>miR-146b-5p<br>miR-4267<br>miR-425-3p<br>miR-942-5p<br>miR-374c-5p<br>miR-3176<br>miR-550a-5p<br>miR-2276-3p<br>miR-425-5p<br>miR-190a-5p<br>miR-10b-5p<br>miR-139-3p<br>miR-188-5p<br>miR-451a<br>miR-1247-5p	miR-3646: up<br>miR-34c-3p: up<br>miR-181c-3p: up<br>miR-1539: up<br>miR-3185: up<br>miR-4688: up<br>miR-3191-3p: up<br>miR-4291: up<br>miR-363-3p: up<br>miR-219a-1-3p: up<br>miR-628-3p: up<br>miR-1225-3p: up<br>miR-2467-3p: up<br>miR-375: up<br>let-7i-5p: up<br>miR-324-3p: up<br>miR-27b-3p: up<br>miR-4516: up<br>miR-92b-3p: up<br>miR-675-3p: up<br>miR-490-3p: up<br>miR-146b-5p: up<br>miR-4267: up<br>miR-425-3p: up<br>miR-942-5p: up<br>miR-374c-5p: up<br>miR-3176: up<br>miR-550a-5p: up<br>miR-2276-3p: up<br>miR-425-5p: up<br>miR-190a-5p: up<br>miR-10b-5p: up<br>miR-139-3p: up<br>miR-188-5p: up<br>miR-451a: up<br>miR-1247-5p: up	miR-138-1-3p: 4.49<br>let-7d-5p: 4.46<br>miR-10a-5p: 4.25<br>miR-1203: 4.25<br>miR-3646: 4.19<br>miR-34c-3p: 4.05<br>miR-181c-3p: 4.02<br>miR-1539: 3.96<br>miR-3185: 3.96<br>miR-4688: 3.96<br>miR-3191-3p: 3.91<br>miR-4291: 3.88<br>miR-363-3p: 3.88<br>miR-219a-1-3p: 3.83<br>miR-628-3p: 3.8<br>miR-1225-3p: 3.62<br>miR-2467-3p: 3.55<br>miR-375: 3.5<br>let-7i-5p: 3.43<br>miR-324-3p: 3.36<br>miR-27b-3p: 3.36<br>miR-4516: 3.33<br>miR-92b-3p: 3.2<br>miR-675-3p: 3.2<br>miR-490-3p: 3.15<br>miR-146b-5p: 3.09<br>miR-4267: 2.98<br>miR-425-3p: 2.96<br>miR-942-5p: 2.92<br>miR-374c-5p: 2.86<br>miR-3176: 2.8<br>miR-550a-5p: 2.78<br>miR-2276-3p: 2.75<br>miR-425-5p: 2.75<br>miR-190a-5p: 2.67	

					miR-664a-3p<br>miR-361-3p<br>miR-2110<br>miR-605-5p<br>miR-4302<br>miR-1180-3p<br>miR-548o-5p<br>miR-193b-3p<br>miR-331-3p<br>miR-7-1-3p<br>miR-769-5p<br>miR-4538<br>miR-140-5p<br>miR-19a-3p<br>miR-378b<br>miR-4454<br>miR-328-3p<br>miR-598-3p<br>miR-152-3p<br>miR-502-3p<br>miR-382-5p<br>miR-409-3p<br>miR-181d-5p<br>miR-28-5p<br>miR-1307-3p<br>miR-154-5p<br>miR-424-3p<br>miR-132-3p<br>miR-181a-5p<br>miR-30a-3p<br>miR-4323<br>miR-3651<br>miR-210-3p<br>miR-627-5p<br>miR-339-5p<br>miR-34a-5p	miR-664a-3p: up<br>miR-361-3p: up<br>miR-2110: up<br>miR-605-5p: up<br>miR-4302: up<br>miR-1180-3p: up<br>miR-548o-5p: up<br>miR-193b-3p: up<br>miR-331-3p: up<br>miR-7-1-3p: up<br>miR-769-5p: up<br>miR-4538: up<br>miR-140-5p: up<br>miR-19a-3p: up<br>miR-378b: up<br>miR-4454: up<br>miR-328-3p: down<br>miR-598-3p: down<br>miR-152-3p: down<br>miR-502-3p: down<br>miR-382-5p: down<br>miR-409-3p: down<br>miR-181d-5p: down<br>miR-28-5p: down<br>miR-1307-3p: down<br>miR-154-5p: down<br>miR-424-3p: down<br>miR-132-3p: down<br>miR-181a-5p: down<br>miR-30a-3p: down<br>miR-4323: down<br>miR-3651: down<br>miR-210-3p: down<br>miR-627-5p: down<br>miR-339-5p: down<br>miR-34a-5p: down	miR-10b-5p: 2.6<br>miR-139-3p: 2.58<br>miR-188-5p: 2.46<br>miR-451a: 2.41<br>miR-1247-5p: 2.39<br>miR-664a-3p: 2.37<br>miR-361-3p: 2.32<br>miR-2110: 2.31<br>miR-605-5p: 2.29<br>miR-4302: 2.29<br>miR-1180-3p: 2.28<br>miR-548o-5p: 2.28<br>miR-193b-3p: 2.28<br>miR-331-3p: 2.25<br>miR-7-1-3p: 2.25<br>miR-769-5p: 2.2<br>miR-4538: 2.18<br>miR-140-5p: 2.17<br>miR-19a-3p: 2.17<br>miR-378b: 2.04<br>miR-4454: 2.04<br>miR-328-3p: -30.54<br>miR-598-3p: -13.96<br>miR-152-3p: -12.84<br>miR-502-3p: -11.74<br>miR-382-5p: -11.49<br>miR-409-3p: -11.49<br>miR-181d-5p: -8.07<br>miR-28-5p: -7.91<br>miR-1307-3p: -7.58<br>miR-154-5p: -7.03<br>miR-424-3p: -6.98<br>miR-132-3p: -6.79<br>miR-181a-5p: -6.65<br>miR-30a-3p: -6.56<br>miR-4323: -6.25	

					miR-199a-5p	miR-199a-5p: down	miR-3651: -6.03	
					miR-4258	miR-4258: down	miR-210-3p: -5.91	
					miR-378a-3p	miR-378a-3p: down	miR-627-5p: -5.87	
					miR-340-5p	miR-340-5p: down	miR-339-5p: -5.47	
					miR-93-3p	miR-93-3p: down	miR-34a-5p: -5.4	
					miR-130a-3p	miR-130a-3p: down	miR-199a-5p: -4.87	
					miR-15a-5p	miR-15a-5p: down	miR-4258: -4.57	
					miR-151a-3p	miR-151a-3p: down	miR-378a-3p: -4.45	
					miR-421	miR-421: down	miR-340-5p: -4.12	
					miR-199b-3p	miR-199b-3p: down	miR-93-3p: -3.87	
					miR-1976	miR-1976: down	miR-130a-3p: -3.69	
					miR-194-5p	miR-194-5p: down	miR-15a-5p: -3.35	
					miR-324-5p	miR-324-5p: down	miR-151a-3p: -3.3	
					miR-93-5p	miR-93-5p: down	miR-421: -3.26	
					miR-125a-5p	miR-125a-5p: down	miR-199b-3p: -3.19	
					miR-191-5p	miR-191-5p: down	miR-1976: -3.1	
					miR-224-5p	miR-224-5p: down	miR-194-5p: -3.06	
					miR-151a-3p	miR-151a-3p: down	miR-324-5p: -2.95	
					miR-24-3p	miR-24-3p: down	miR-93-5p: -2.93	
					miR-192-5p	miR-192-5p: down	miR-125a-5p: -2.81	
					miR-19b-3p	miR-19b-3p: down	miR-191-5p: -2.78	
					miR-16-5p	miR-16-5p: down	miR-224-5p: -2.76	
					miR-146a-5p	miR-146a-5p: down	miR-151a-3p: -2.76	
					miR-186-5p	miR-186-5p: down	miR-24-3p: -2.76	
					miR-433-3p	miR-433-3p: down	miR-192-5p: -2.74	
					miR-126-3p	miR-126-3p: down	miR-19b-3p: -2.68	
					miR-505-3p	miR-505-3p: down	miR-16-5p: -2.66	
					miR-21-5p	miR-21-5p: down	miR-146a-5p: -2.64	
					miR-151b	miR-151b: down	miR-186-5p: -2.61	
					miR-25-3p	miR-25-3p: down	miR-433-3p: -2.59	
					miR-342-3p	miR-342-3p: down	miR-126-3p: -2.55	
					miR-15b-5p	miR-15b-5p: down	miR-505-3p: -2.52	
					miR-7-5p	miR-7-5p: down	miR-21-5p: -2.37	
							miR-151b: -2.35	
							miR-25-3p: -2.16	
							miR-342-3p: -2.02	

							miR-15b-5p: -2.02<br>miR-7-5p: -2	
(Guo *et al.*, 2023)	17	19	Plasma	RT-qPCR	miR-223	down	NI	p<0.031
(Hu *et al.*, 2022)	113	113	Plasma	RT-qPCR	miR-193b-3p	up	2.01	p= 0.006
(Kong *et al.*, 2022)	342	326	Serum	RT-qPCR	miR-143	up	NI	p<0.001
(Lin *et al.*, 2021)	30	36	Serum	RT-qPCR	miR-638	down	NI	p<0.001
(Liu *et al.*, 2022a)	50	76	Plasma	RT-qPCR	miR-203	down	NI	p<0.01
(Liu *et al.*, 2022b)	180	180	Serum	RT-qPCR	miR-29a	up	NI	p<0.0001
(Meng *et al.*, 2023)	30	36	Serum	RT-qPCR	miR-23a-3p	down	NI	P=0.0305
(Mokhtari Ardekani *et al.*, 2023)	145	150	Serum	RT-qPCR	miR-122	up	NI	P<0.001
(Monfared *et al.*, 2022)	40	40	Serum	RT-qPCR	miR-182<br>miR-320a<br>miR-375<br>miR-503	miR-182: up<br>miR-320a: down<br>miR-375: up<br>miR-503: up	NI	miR-182: p<0.05<br>miR-320a: p<0.001<br>miR-375: p<0.05<br>miR-503: p<0.05
(Nemecz *et al.*, 2023)	15	17	Plasma	RT-qPCR	miR-21-5p<br>miR-34a-5p<br>miR-132-3p<br>miR-143-3p<br>miR-223-3p<br>miR-122-5p<br>miR-155-5p<br>miR 200b-3p<br>miR-218-5p<br>miR-212-5p<br>miR-126-3p	miR-21-5p: NS<br>miR-34a-5p: NS<br>miR-132-3p: NS<br>miR-143-3p: NS<br>miR-223-3p: NS<br>miR-122-5p: up<br>miR-155-5p: up<br>miR 200b-3p: NS<br>miR-218-5p: up<br>miR-212-5p: up<br>miR-126-3p: NS	NI	miR-21-5p: NS<br>miR-34a-5p: NS<br>miR-132-3p: NS<br>miR-143-3p: NS<br>miR-223-3p: NS<br>miR-122-5p: p<0.05<br>miR-155-5p: p<0.05<br>miR 200b-3p: NS<br>miR-218-5p: p<0.05<br>miR-212-5p: p<0.05<br>miR-126-3p: NS
(Pan *et al.*, 2022)	36	12	Serum	RT-qPCR	miR-4431	up	NI	p<0.05

(Ruan *et al.*, 2023)	52	85	Serum	RT-qPCR	miR-199-3p	down	NI	P<0.0001
(Saleh *et al.*, 2022)	80	80	Serum	RT-qPCR	miR-93<br>miR-152	miR-93: down<br>miR-152: up	NI	miR-93: p<0.001<br>miR-152: p<0.001
(Ghaneh *et al.*, 2023)	17	16	Plasma	RT-qPCR	miR-30d-5p<br>miR-126-3p	miR-30d-5p: NS<br>miR-126-3p: NS	miR-30d-5p: NS<br>miR-126-3p: NS	miR-30d-5p: NS<br>miR-126-3p: NS
(Shahouzehi *et al.*, 2021)	15	15	Serum	RT-qPCR	miR-33	NS	NS	NS
(Simoniene *et al.*, 2022)	54	53	Serum	RT-qPCR	miR-107	up	NI	p=0.016
(Su *et al.*, 2022)	101	101	Serum	RT-qPCR	miR 29a-5p<br>miR-3690<br>miR-607<br>miR-409-3p	miR 29a-5p: up<br>miR-3690: up<br>miR-607: up<br>miR-409-3p: NS	NI	miR 29a-5p: p<0.001<br>miR-3690: p<0.001<br>miR-607: p<0.001<br>miR-409-3p: NS
(Sun *et al.*, 2023)	9	10	Serum	RT-qPCR	miR-21	NS	NS	NS
(Sun *et al.*, 2022)	30	50	Serum	RT-qPCR	miR-146a	down	NI	p<0.001
(Tonyan *et al.*, 2023)	22	44	Plasma	RT-qPCR	miR-496<br>miR-5588-5p<br>miR-125b-2-3p<br>miR-1284	miR-496: down<br>miR-5588-5p: NS<br>miR-125b-2-3p: NS<br>miR-1284: NS	NI	miR-496: p=0.036<br>miR-5588-5p: NS<br>miR-125b-2-3p: NS<br>miR-1284: NS
(Tursinawati *et al.*, 2022)	17	30	Plasma	RT-qPCR	miR-29b	up	14.12	p=0.000
(Wu *et al.*, 2021a)	60	60	Plasma	RT-qPCR	miR-34c	up	NI	p<0.01
(Wu *et al.*, 2021b)	21	10	Serum	RT-qPCR	miR-375	up	NI	p<0.001
(Yazdanpanah *et al.*, 2022)	29	24	Plasma	RT-qPCR	miR-21<br>miR-126	miR-21: up<br>miR-126: NS	NI	miR-21: p<0.0001<br>miR-126: NS
(Yun *et al.*, 2022)	42	50	Serum	RT-qPCR	miR-590-3p	down	NI	p<0.001
(Zhao *et al.*, 2023)	60	64	Plasma	RT-qPCR	miR-204-3p	down	NI	p<0.05

Supplementary material 13: Pathways targeted by down and upregulated miRNAs.

- **Pathways targeted by downregulated miRNAs:**

Pathway	GeneRatio	p.adjust	geneID
WP_PI3KAKT_SIGNALING_PATHWAY	52/313	1.14E-25	CCNE1/MYB/CCND2/CDKN1A/MYC/MET/CDK4/CCNE2/BCL2/CDK6/CCND1/BRCA1/AKT3/PTEN/VEGFA/ITGB8/MAP2K1/BCL2L11/IFNB1/CHUK/TP53/FGF7/PDGFRA/IGF1R/TLR2/PRKAA1/PPP2R5E/HSP90B1/IL6/NRAS/STK11/PHLPP2/ITGB3/CDK2/PDGFRB/BDNF/MAPK1/KDR/PHLPP1/FOXO3/CSF1R/KIT/IL6R/INSR/EGFR/RAF1/IGF1/IKBKG/CCND3/PIK3CG/AKT1/PIK3R3
REACTOME_SIGNALING_BY_INTERLEUKINS	58/313	9.95E-24	MEF2C/CDKN1A/MYC/BCL2/CCND1/VAMP2/CASP3/VEGFA/SOCS1/MAP2K1/IL1A/CHUK/CCL5/TP53/CRKL/NANOG/SOX2/LIF/STX1A/IL13/FOXO1/CCL22/IFNG/HSP90B1/CRK/IL6/BIRC5/MAPK1/CBL/STAT5A/CXCL8/FOXO3/CSF1R/CCL3/CXCL2/IL6R/IRAK4/CXCL10/IKBKG/TAB2/TAB3/STAT3/CCL2/PDCD4/AKT1/SOCS3/STAT1/FOS/MMP9/NLRC5/TGFB1/RPS6KA3/AIP/ZEB1/PIK3R3/IL10RA/MMP3/HMGB1
WP_EGFR_TYROSINE_KINASE_INHIBITOR_RESISTANCE	28/313	4.63E-23	MYC/MET/BCL2/CCND1/AKT3/PTEN/VEGFA/MAP2K1/BCL2L11/PDGFRA/IGF1R/AXL/IL6/NRAS/PDGFRB/SRC/MAPK1/KDR/FOXO3/IL6R/EGFR/RAF1/BAX/IGF1/STAT3/ERBB2/AKT1/PIK3R3
REACTOME_INTERLEUKIN_4_AND_INTERLEUKIN_13_SIGNALING	30/313	8.58E-22	CDKN1A/MYC/BCL2/CCND1/VEGFA/SOCS1/IL1A/TP53/NANOG/SOX2/LIF/IL13/FOXO1/CCL22/HSP90B1/IL6/BIRC5/CXCL8/FOXO3/IL6R/STAT3/CCL2/AKT1/SOCS3/STAT1/FOS/MMP9/TGFB1/ZEB1/MMP3

WP_DNA_DAMAGE_RESPONSE_ONLY_ATM_DEPENDENT	29/313	8.72E-21	WNT3A/CCND2/CDKN1A/MYC/BCL2/CCND1/WNT1/AKT3/PTEN/BCL2L11/TP53/FOSL1/PPP2R5E/LEF1/NRAS/SMAD4/ATM/MAPK1/FOXO3/TCF7/INSR/BAX/CCND3/ERBB2/PIK3CG/AKT1/TGFB1/PIK3R3/HMGB1
PID_SMAD2_3NUCLEAR_PATHWAY	24/313	2.04E-18	MEF2C/CDKN1A/MYC/CDK4/HNF4A/ESR1/KAT2B/IFNB1/SP3/FOXO1/HDAC1/SMAD4/CDK2/AR/SMAD7/CEBPB/TGIF2/E2F5/FOXO3/SP1/AKT1/FOS/SMAD2/CDKN2B
WP_DNA_DAMAGE_RESPONSE	22/313	9.10E-18	CCNE1/CDC25A/CCND2/CDKN1A/E2F1/MYC/CDK4/CCNE2/CDK6/CCND1/BRCA1/CASP3/TP53/RB1/CCNB1/CHEK1/CDK2/ATM/BAX/CCND3/AKT1/RAD51
WP_SPINAL_CORD_INJURY	26/313	7.28E-17	E2F1/MYC/CDK4/CCND1/RHOB/CASP3/IL1A/TP53/RB1/IFNG/IL6/CDK2/EGR1/BDNF/MAPK1/E2F5/CXCL8/FOXO3/CXCL2/RHOC/CXCL10/EGFR/CCL2/FOS/MMP9/TGFB1
WP_FOCAL_ADHESION_PI3KAKTMTORSIGNALING_PATHWAY	38/313	5.27E-16	CDKN1A/MET/AKT3/PTEN/VEGFA/ITGB8/MAP2K1/IFNB1/FGF7/PDGFRA/IGF1R/SLC2A4/FOXO1/PRKAA1/PPP2R5E/HSP90B1/NRAS/STK11/PHLPP2/ITGB3/PDGFRB/MAPK1/KDR/SREBF1/PHLPP1/FOXO3/CSF1R/KIT/IL6R/INSR/EGFR/RAF1/IGF1/IKBKG/PIK3CG/AKT1/SLC2A1/FOXA1
WP_TGFBETA_SIGNALING_PATHWAY	26/313	1.32E-15	MEF2C/CDKN1A/MYC/MET/CCND1/MAP2K1/TGFBR2/TP53/HDAC1/SMAD4/ITGB3/SRC/DAB2/SMAD7/YAP1/SMURF1/MAPK1/E2F5/SP1/RAF1/AKT1/FOS/TGFB1/SMAD2/CDKN2B/ZEB1
WP_ERBB_SIGNALING_PATHWAY	22/313	4.49E-15	CDKN1A/MYC/CCND1/AKT3/MAP2K1/BCL2L11/TP53/CRKL/HBEGF/FOXO1/CRK/NRAS/SRC/MAPK1/CBL/STAT5A/EGFR/RAF1/PAK1/ERBB2/AKT1/PIK3R3
WP_NEUROINFLAMMATION_AND_GLUTAMATERGIC_SIGNALING	26/313	5.46E-15	GRM7/BCL2/TGFBR2/IL1A/LIF/IL13/IFNG/IL6/SMAD4/SMAD7/LRRC8A/BDNF/MAPK1/IL6R/INSR/IGF1/STAT3/AKT1/SOCS3/STAT1/FOS/TGFB1/SMAD2/IL10RA/PPP1CC/SLC2A1

KEGG_FOCAL_ADHESION	30/313	9.15E-15	CCND2/MET/BCL2/CCND1/AKT3/PTEN/VEGFA/ITGB8/MAP2K1/CRKL/PDGFRA/IGF1R/CRK/TLN2/ITGB3/PDGFRB/SRC/MAPK1/KDR/MYL9/EGFR/RAF1/IGF1/PAK1/CCND3/ERBB2/PIK3CG/AKT1/PIK3R3/PPP1CC
PID_AP1_PATHWAY	19/313	3.81E-14	MYB/MYC/CCND1/DMTF1/PTEN/ESR1/BCL2L11/TP53/FOSL1/IFNG/IL6/EGR1/EDN1/CXCL8/SP1/CCL2/FOS/MMP9/TGFB1
WP_FOCAL_ADHESION	29/313	5.92E-14	CCND2/MET/BCL2/CCND1/AKT3/PTEN/VEGFA/ITGB8/MAP2K1/CRKL/PDGFRA/IGF1R/CRK/TLN2/ITGB3/PDGFRB/SRC/MAPK1/KDR/MYL9/EGFR/RAF1/IGF1/PAK1/CCND3/ERBB2/AKT1/PIK3R3/PPP1CC
REACTOME_INTRACELLULAR_SIGNALING_BY_SECOND_MESSENGERS	35/313	1.67E-13	BMI1/CDKN1A/MET/AKT3/PTEN/ESR1/CHUK/EZH2/TP53/FGF7/PDGFRA/HBEGF/FOXO1/PPP2R5E/KLB/MTA2/HDAC1/PHLPP2/SNAI1/PDGFRB/SRC/EGR1/RICTOR/MAPK1/PHLPP1/FOXO3/KIT/IRAK4/AGO2/INSR/EGFR/ERBB2/AKT1/SALL4/PIK3R3
WP_AGERAGE_PATHWAY	18/313	1.84E-13	CASP3/MAP2K1/CHUK/FOXO1/SRC/INHBB/MAPK1/STAT5A/SP1/IRAK4/INSR/EGFR/RAF1/STAT3/AKT1/STAT1/MMP9/SMAD2
WP_MIRNA_REGULATION_OF_DNA_DAMAGE_RESPONSE	21/313	1.92E-13	CCNE1/CDC25A/CCND2/CDKN1A/E2F1/MYC/CDK4/CCNE2/CDK6/CCND1/BRCA1/CASP3/TP53/RB1/CCNB1/CHEK1/CDK2/ATM/BAX/CCND3/RAD51
WP_IL2_SIGNALING_PATHWAY	15/313	3.53E-13	CCND2/MYC/BCL2/MAP2K1/CRKL/MAPK1/CBL/STAT5A/FOXO3/RAF1/STAT3/AKT1/SOCS3/STAT1/FOS
REACTOME_SIGNALING_BY_TGF_BETA_RECEPTOR_COMPLEX	20/313	7.33E-13	MYC/ITGB8/TGFBR2/HDAC1/SMAD4/ITGB3/PARP1/SMAD7/SMURF1/MAPK1/TGIF2/E2F5/CBL/SP1/STAT1/TGFB1/SMAD2/CDKN2B/CDK9/PPP1CC
WP_ADIPOGENESIS	23/313	8.34E-13	MEF2C/CDKN1A/E2F1/WNT1/SOCS1/LIF/RB1/SLC2A4/FOXO1/HMGA1/IL6/NAMPT/CEBPB/HNF1A/STAT5A/SREBF1/SP1/IGF1/PPARA/STAT3/SOCS3/STAT1/TGFB1

REACTOME_SIGNALING_BY_TGFB_FAMILY_MEMBERS	22/313	1.66E-12	MYC/ITGB8/TGFBR2/BMPR2/HDAC1/SMAD4/ITGB3/PARP1/SMAD7/INHBB/SMURF1/MAPK1/TGIF2/E2F5/CBL/SP1/STAT1/TGFB1/SMAD2/CDKN2B/CDK9/PPP1CC
KEGG_ERBB_SIGNALING_PATHWAY	19/313	2.23E-12	CDKN1A/MYC/AKT3/MAP2K1/CRKL/HBEGF/CRK/NRAS/SRC/MAPK1/CBL/STAT5A/EGFR/RAF1/PAK1/ERBB2/PIK3CG/AKT1/PIK3R3
WP_LEPTIN_SIGNALING_PATHWAY	18/313	2.23E-12	CCND1/PTEN/ESR1/MAP2K1/CHUK/FOXO1/PRKAA1/SRC/MAPK1/SP1/RAF1/BAX/IKBKG/STAT3/ERBB2/AKT1/SOCS3/STAT1
WP_MAPK_SIGNALING_PATHWAY	30/313	2.41E-12	MEF2C/STMN1/MYC/AKT3/CASP3/MAP2K1/TGFBR2/IL1A/CHUK/TP53/FGF7/CRKL/RASA1/CRK/NRAS/PDGFRB/BDNF/MAPK1/HSPA1B/EGFR/RAF1/IKBKG/TAB2/CACNB3/PAK1/RPS6KA4/AKT1/FOS/TGFB1/RPS6KA3
REACTOME_NEGATIVE_REGULATION_OF_THE_PI3K_AKT_NETWORK	21/313	3.08E-12	MET/AKT3/PTEN/ESR1/FGF7/PDGFRA/HBEGF/PPP2R5E/KLB/PHLPP2/PDGFRB/SRC/MAPK1/PHLPP1/KIT/IRAK4/INSR/EGFR/ERBB2/AKT1/PIK3R3
KEGG_MAPK_SIGNALING_PATHWAY	31/313	3.08E-12	MEF2C/STMN1/MYC/AKT3/CASP3/MAP2K1/TGFBR2/IL1A/CHUK/TP53/FGF7/CRKL/PDGFRA/RASA1/CRK/NRAS/PDGFRB/BDNF/MAPK1/HSPA1B/EGFR/RAF1/IKBKG/TAB2/CACNB3/PAK1/RPS6KA4/AKT1/FOS/TGFB1/RPS6KA3
KEGG_JAK_STAT_SIGNALING_PATHWAY	24/313	3.53E-12	CCND2/MYC/CCND1/AKT3/SOCS1/IFNB1/SOCS4/LIF/IL13/IFNG/SPRED1/IL6/CBL/STAT5A/IL6R/STAT3/CCND3/PIK3CG/AKT1/SOCS3/STAT1/SPRY1/PIK3R3/IL10RA
WP_TOLLLIKE_RECEPTOR_SIGNALING_PATHWAY	20/313	4.81E-12	AKT3/MAP2K1/IFNB1/CHUK/CCL5/TLR2/IL6/MAPK1/CXCL8/CCL3/IRAK4/CXCL10/IKBKG/TAB2/TAB3/PIK3CG/AKT1/STAT1/FOS/PIK3R3
WP_IL18_SIGNALING_PATHWAY	31/313	5.32E-12	GRM7/STMN1/BCL2/PTEN/CASP3/VEGFA/CHUK/CCL5/TP53/FBXW7/ULBP2/IL13/PRKAA1/IFNG/CCNA2/IL6/PARP1/MAPK1/CEBPB/CXCL8/SP1/CCL3/CXCL2/TNFAIP3/BAX/CCL2/KCNH2/SOCS3/FOS/MMP9/MMP3

WP_INSULIN_SIGNALING	24/313	6.75E-12	VAMP2/PTEN/SOCS1/MAP2K1/IGF1R/SLC2A4/FOXO1/PRKAA1/CRK/EGR1/MAPK1/CBL/FOXO3/INSR/RAF1/RPS6KA4/PIK3CG/AKT1/SOCS3/FLOT2/FOS/RPS6KA3/PIK3R3/SLC2A1
WP_EGFEGFR_SIGNALING_PATHWAY	24/313	8.83E-12	MEF2C/STMN1/E2F1/PTEN/MAP2K1/CRKL/FOXO1/RASA1/CRK/SRC/RICTOR/MAPK1/CBL/STAT5A/SP1/EGFR/RAF1/STAT3/PAK1/ERBB2/AKT1/STAT1/FOS/RPS6KA3
KEGG_INSULIN_SIGNALING_PATHWAY	22/313	1.60E-11	AKT3/SOCS1/MAP2K1/SOCS4/CRKL/SLC2A4/FOXO1/PRKAA1/CRK/HK2/NRAS/MAPK1/CBL/SREBF1/INSR/RAF1/PIK3CG/AKT1/SOCS3/FLOT2/PIK3R3/PPP1CC
BIOCARTA_IL2RB_PATHWAY	13/313	2.01E-11	E2F1/MYC/BCL2/SOCS1/CRKL/MAPK1/CBL/STAT5A/RAF1/PIK3CG/AKT1/SOCS3/FOS
PID_IL2_1PATHWAY	15/313	2.35E-11	MYC/BCL2/SOCS1/MAP2K1/IFNG/RASA1/NRAS/CDK2/MAPK1/STAT5A/RAF1/STAT3/SOCS3/STAT1/FOS
WP_WNT_SIGNALING_PATHWAY_AND_PLURIPOTENCY	19/313	3.10E-11	WNT3A/CCND2/MYC/CCND1/WNT1/AXIN2/CD44/TP53/NANOG/SOX2/FOSL1/PPP2R5E/LEF1/POU5F1/TCF7/CCND3/CTBP2/PRKD1/CTNND1
KEGG_TOLL_LIKE_RECEPTOR_SIGNALING_PATHWAY	19/313	3.69E-11	AKT3/MAP2K1/IFNB1/CHUK/CCL5/TLR2/IL6/MAPK1/CXCL8/CCL3/IRAK4/CXCL10/IKBKG/TAB2/PIK3CG/AKT1/STAT1/FOS/PIK3R3
WP_ANGIOPOIETINLIKE_PROTEIN_8_REGULATORY_PATHWAY	21/313	5.81E-11	MAP2K1/SLC2A4/FOXO1/PRKAA1/CRK/SLC16A2/RICTOR/MAPK1/CBL/SREBF1/SREBF2/FOXO3/INSR/RAF1/RPS6KA4/PIK3CG/AKT1/FLOT2/RPS6KA3/PIK3R3/SLC2A1
WP_IL5_SIGNALING_PATHWAY	13/313	5.81E-11	MYC/BCL2/MAP2K1/SPRED1/MAPK1/STAT5A/FOXO3/RAF1/STAT3/PIK3CG/AKT1/STAT1/FOS
KEGG_CYTOKINE_CYTOKINE_RECEPTOR_INTERACTION	29/313	6.91E-11	MET/VEGFA/TGFBR2/IFNB1/IL1A/BMPR2/CCL5/BMP7/PDGFRA/LIF/IL13/CCL22/IFNG/IL6/PDGFRB/INHBB/KDR/CXCL8/CSF1R/KIT/CCL3/CXCL2/IL6R/CXCL10/EGFR/CCL2/TGFB1/GDF5/IL10RA
BIOCARTA_MET_PATHWAY	12/313	8.28E-11	MET/PTEN/MAP2K1/CRKL/RASA1/CRK/MAPK1/RAF1/STAT3/PAK1/PIK3CG/FOS

WP_PROSTAGLANDIN_SIGNALING	12/313	8.28E-11	VEGFA/IL1A/IFNG/IL6/CXCL8/CCL3/CXCL10/CCL2/PIK3CG/NLRP3/MMP9/TGFB1
WP_VEGFAVEGFR2_SIGNALING	37/313	1.04E-10	JAG1/MEF2C/BCL2/CCND1/VEGFA/MAP2K1/HBEGF/FOXO1/PRKAA1/CRK/LDHA/HDAC1/ITGB3/BIRC5/SRC/TAL1/EGR1/RICTOR/MAPK1/KDR/CBL/CYBB/CXCL8/FOXO3/RHOC/NUMB/RAF1/STAT3/PAK1/CCL2/AKT1/STAT1/PRKD1/CTNND1/NR4A2/RCAN1/HMGB1
WP_HEPATOCYTE_GROWTH_FACTOR_RECEPTOR_SIGNALING	12/313	1.21E-10	MET/PTEN/MAP2K1/CRKL/RASA1/CRK/SRC/MAPK1/RAF1/STAT3/PAK1/FOS
KEGG_TGF_BETA_SIGNALING_PATHWAY	17/313	1.69E-10	MYC/TGFBR2/BMPR2/BMP7/IFNG/SMAD4/SMAD7/INHBB/SMURF1/MAPK1/ID4/E2F5/SP1/TGFB1/SMAD2/CDKN2B/GDF5
REACTOME_SIGNALING_BY_NUCLEAR_RECEPTORS	30/313	1.97E-10	MYB/MYC/BCL2/CCND1/AKT3/YY1/MYLIP/ESR1/KAT2B/IGF1R/HBEGF/HDAC1/NRAS/SRC/KDM4A/CARM1/MAPK1/SREBF1/FOXO3/SP1/AGO2/ABCA1/EGFR/AKT1/FOS/MMP9/CDK9/PIK3R3/MMP3/FOXA1
REACTOME_SIGNALING_BY_PTK6	14/313	2.25E-10	CCNE1/CDK4/CCND1/HBEGF/RASA1/CRK/NRAS/CDK2/CBL/EGFR/STAT3/ERBB2/AKT1/SOCS3
WP_EXTRACELLULAR_VESICLE MEDIATED_SIGNALING_IN_RECIPIENT_CELLS	11/313	5.03E-10	WNT3A/MET/TGFBR2/NRAS/SMAD4/EGFR/RAF1/ERBB2/AKT1/TGFB1/SMAD2
WP_IL3_SIGNALING_PATHWAY	13/313	8.19E-10	BCL2/MAP2K1/CRKL/SRC/MAPK1/CBL/STAT5A/CXCL8/RAF1/STAT3/AKT1/FOS/TGFB1
WP_ATM_SIGNALING_PATHWAY	12/313	9.14E-10	CCNE1/CDC25A/CDKN1A/BRCA1/MDM4/TP53/CCNB1/CHEK1/CDK2/ATM/IKBKG/RAD51
KEGG_CHEMOKINE_SIGNALING_PATHWAY	23/313	1.17E-09	AKT3/MAP2K1/CHUK/CCL5/CRKL/CCL22/CRK/NRAS/MAPK1/CXCL8/FOXO3/CCL3/CXCL2/CXCL10/RAF1/IKBKG/STAT3/PAK1/CCL2/PIK3CG/AKT1/STAT1/PIK3R3

KEGG_NEUROTROPHIN_SIGNALING_PATHWAY	19/313	1.27E-09	BCL2/AKT3/MAP2K1/TP53/CRKL/CRK/NRAS/BDNF/MAPK1/ARHGDIB/FOXO3/IRAK4/RAF1/BAX/RPS6KA4/PIK3CG/AKT1/RPS6KA3/PIK3R3
REACTOME_TRANSCRIPTIONAL_ACTIVITY_OF_SMAD2_SMAD3_SMAD4_HETEROTRIMER	13/313	1.36E-09	MYC/HDAC1/SMAD4/PARP1/SMAD7/MAPK1/TGIF2/E2F5/SP1/STAT1/SMAD2/CDKN2B/CDK9
PID_TCPTP_PATHWAY	12/313	1.66E-09	MET/VEGFA/PDGFRB/SRC/KDR/STAT5A/CSF1R/INSR/EGFR/STAT3/STAT1/PIK3R3
REACTOME_MAPK_FAMILY_SIGNALING_CASCADES	30/313	1.85E-09	MYC/MET/MAP2K1/FGF7/PDGFRA/HBEGF/ARTN/FOXO1/PPP2R5E/KLB/RASA1/SPRED1/IL6/NRAS/ITGB3/PDGFRB/SRC/MAPK1/FOXO3/KIT/RET/IL6R/AGO2/GFRA3/EGFR/RAF1/PAK1/CCND3/ERBB2/PPP1CC
PID_MYC_REPRESS_PATHWAY	14/313	1.85E-09	CDKN1A/MYC/BCL2/CCND1/BRCA1/CCL5/HDAC1/SMAD4/PDGFRB/FOXO3/SP1/ERBB2/SMAD2/CDKN2B
WP_INTERLEUKIN11_SIGNALING_PATHWAY	12/313	2.95E-09	BCL2/MAP2K1/CHUK/BIRC5/SRC/MAPK1/RAF1/STAT3/AKT1/SOCS3/STAT1/TGFB1
REACTOME_FOXO_MEDIATED_TRANSCRIPTION	14/313	3.48E-09	SIRT1/CDKN1A/AKT3/KAT2B/BCL2L11/FOXO1/HDAC1/STK11/SMAD4/KLF4/SREBF1/FOXO3/AKT1/SMAD2
REACTOME_SMAD2_SMAD3_SMAD4_HETEROTRIMER_REGULATES_TRANSCRIPTION	11/313	4.19E-09	MYC/HDAC1/SMAD4/SMAD7/MAPK1/TGIF2/E2F5/SP1/SMAD2/CDKN2B/CDK9
REACTOME_INTERLEUKIN_10_SIGNALING	12/313	4.95E-09	IL1A/CCL5/LIF/CCL22/IL6/CXCL8/CCL3/CXCL2/CXCL10/STAT3/CCL2/IL10RA
PID_SHP2_PATHWAY	13/313	5.61E-09	VEGFA/MAP2K1/IGF1R/IFNG/IL6/NRAS/PDGFRB/BDNF/KDR/IL6R/EGFR/RAF1/STAT1
REACTOME_DOWNSTREAM_SIGNAL_TRANSDUCTION	10/313	6.80E-09	CRKL/PDGFRA/RASA1/CRK/NRAS/PDGFRB/SRC/STAT5A/STAT3/STAT1

PID_CMYB_PATHWAY	15/313	9.76E-09	MYB/CDKN1A/MYC/BCL2/CDK6/CCND1/WNT1/CCNB1/LEF1/NRAS/CEBPB/SP1/CSF1R/KIT/TAB2
WP_BRAINDERIVED_NEUROTROPHIC_FACTOR_BDNF_SIGNALING_PATHWAY	19/313	1.14E-08	MEF2C/CASP3/MAP2K1/BCL2L11/CHUK/PRKAA1/SRC/EGR1/BDNF/MAPK1/STAT5A/FOXO3/RAF1/IKBKG/STAT3/AKT1/STAT1/FOS/RPS6KA3
KEGG_ADHERENS_JUNCTION	14/313	1.29E-08	MET/TGFBR2/IGF1R/LEF1/SMAD4/SNAI1/SRC/MAPK1/TCF7/INSR/EGFR/ERBB2/SMAD2/CTNND1
WP_NOTCH_SIGNALING_PATHWAY	13/313	1.29E-08	JAG1/CDKN1A/MYC/CCND1/DLL1/NOTCH1/NOTCH2/FBXW7/HDAC1/SRC/NUMB/STAT3/AKT1
PID_IFNG_PATHWAY	11/313	1.33E-08	SOCS1/MAP2K1/CRKL/IFNG/SMAD7/MAPK1/CEBPB/CBL/STAT3/AKT1/STAT1
WP_PDGF_PATHWAY	11/313	1.33E-08	MAP2K1/CHUK/RASA1/PDGFRB/SRC/MAPK1/RAF1/STAT3/PAK1/STAT1/FOS
KEGG_NOD_LIKE_RECEPTOR_SIGNALING_PATHWAY	13/313	1.57E-08	CHUK/CCL5/HSP90B1/IL6/MAPK1/CXCL8/CXCL2/TNFAIP3/IKBKG/TAB2/TAB3/CCL2/NLRP3
WP_CHEMOKINE_SIGNALING_PATHWAY	20/313	1.78E-08	AKT3/MAP2K1/CHUK/CCL5/CRKL/CCL22/CRK/NRAS/MAPK1/FOXO3/CCL3/CXCL10/RAF1/IKBKG/STAT3/PAK1/PIK3CG/AKT1/STAT1/PIK3R3
WP_RAS_SIGNALING	21/313	2.07E-08	MET/AKT3/MAP2K1/CHUK/ZAP70/PDGFRA/IGF1R/RASA1/NRAS/PDGFRB/MAPK1/KDR/CSF1R/KIT/INSR/EGFR/RAF1/IKBKG/PAK1/AKT1/PIK3R3
WP_TCELL_RECEPTOR_SIGNALING_PATHWAY	15/313	2.43E-08	MAP2K1/IL1A/CHUK/ZAP70/CRKL/CRK/IL6/MAPK1/CBL/RAF1/IKBKG/PAK1/AKT1/FOS/TGFB1
PID_ERBB1_DOWNSTREAM_PATHWAY	16/313	2.71E-08	MEF2C/MAP2K1/NRAS/SRC/EGR1/RICTOR/MAPK1/EGFR/RAF1/STAT3/RPS6KA4/AKT1/STAT1/FOS/RPS6KA3/PIK3R3

WP_RELATIONSHIP_BETWEEN_INFLAMMATION_COX2_AND_EGFR	9/313	2.89E-08	AKT3/ESR1/CYP19A1/NRAS/SRC/MAPK1/EGFR/PIK3CG/AKT1
REACTOME_SIGNALING_BY_SCF_KIT	11/313	2.95E-08	SOCS1/NRAS/CHEK1/SRC/CBL/STAT5A/KIT/STAT3/STAT1/MMP9/PIK3R3
BIOCARTA_CHEMICAL_PATHWAY	8/313	3.06E-08	BCL2/CASP3/TP53/PARP1/ATM/BAX/AKT1/STAT1
WP_IL4_SIGNALING_PATHWAY	12/313	3.13E-08	SOCS1/CHUK/BIRC5/MAPK1/CEBPB/CBL/STAT5A/STAT3/AKT1/SOCS3/STAT1/FOS
WP_INTERFERON_TYPE_I_SIGNALING_PATHWAYS	12/313	3.13E-08	SOCS1/EIF4A1/ZAP70/CRKL/CRK/CBL/STAT5A/STAT3/RPS6KA4/PDCD4/SOCS3/STAT1
WP_TGFBETA_RECEPTOR_SIGNALING	12/313	3.88E-08	WNT1/TGFBR2/LIF/IFNG/LEF1/SMAD4/SMAD7/STAT3/STAT1/FOS/TGFB1/SMAD2
KEGG_T_CELL_RECEPTOR_SIGNALING_PATHWAY	16/313	3.92E-08	CDK4/AKT3/MAP2K1/CHUK/ZAP70/IFNG/NRAS/MAPK1/CBL/RAF1/IKBKG/PAK1/PIK3CG/AKT1/FOS/PIK3R3
KEGG_ADIPOCYTOKINE_SIGNALING_PATHWAY	13/313	3.92E-08	AKT3/CHUK/SLC2A4/PRKAA1/ACSL4/ACSL1/STK11/IKBKG/PPARA/STAT3/AKT1/SOCS3/SLC2A1
WP_AMPACTIVATED_PROTEIN_KINASE_SIGNALING	13/313	3.92E-08	CDKN1A/HNF4A/TP53/SLC2A4/PRKAA1/CCNA2/CCNB1/STK11/SREBF1/INSR/PIK3CG/AKT1/PIK3R3
PID_PI3KCI_AKT_PATHWAY	10/313	4.56E-08	CDKN1A/AKT3/CHUK/SLC2A4/FOXO1/SRC/RICTOR/FOXO3/RAF1/AKT1
WP_NUCLEAR_RECEPTORS_METAPATHWAY	27/313	5.07E-08	MYC/CDK4/CCND1/ESR1/KAT2B/TGFBR2/SLC2A4/HBEGF/FOXO1/IFNG/SRC/EGR1/LRRC8A/POU5F1/SREBF1/SP1/ABCB1/TNFAIP3/EGFR/BAX/PPARA/STAT3/CCL2/SPRY1/TGFB1/AIP/SLC2A1

REACTOME_SENESCENCE_ASSOCIATED_SECRETORY_PHENOTYPE_SASP	16/313	6.43E-08	CDKN1A/CDK4/CDK6/IL1A/CCNA2/CDKN2C/IL6/CDK2/MAPK1/CEBPB/CXCL8/CDC27/STAT3/FOS/CDKN2B/RPS6KA3
WP_OVERVIEW_OF_PROINFLAMMATORY_AND_PROFIBROTIC_MEDIATORS	17/313	6.66E-08	VEGFA/IFNB1/IL1A/CCL5/LIF/IL13/CCL22/IFNG/IL6/CXCL8/CCL3/CXCL2/CXCL10/CCL2/MMP9/TGFB1/MMP3
WP_HIPPO_SIGNALING_REGULATION_PATHWAYS	15/313	7.21E-08	MET/PDGFRA/IGF1R/PRKAA1/LATS2/LEF1/PDGFRB/KDR/CSF1R/KIT/TCF7/INSR/EGFR/SMAD2/YY1AP1
PID_IL6_7_PATHWAY	11/313	7.38E-08	MYC/FOXO1/HSP90B1/IL6/CEBPB/IL6R/STAT3/AKT1/SOCS3/STAT1/FOS
PID_FRA_PATHWAY	10/313	7.87E-08	CCND1/DMTF1/LIF/FOSL1/CCNA2/IL6/CXCL8/SP1/CCL2/MMP9
BIOCARTA_PDGF_PATHWAY	9/313	8.12E-08	MAP2K1/PDGFRA/RASA1/STAT5A/RAF1/STAT3/PIK3CG/STAT1/FOS
PID_NOTCH_PATHWAY	12/313	8.28E-08	JAG1/CDKN1A/MYC/CCND1/DLL1/NOTCH1/YY1/NOTCH2/FBXW7/HDAC1/CBL/NUMB
PID_HES_HEY_PATHWAY	11/313	9.09E-08	MYB/E2F1/NOTCH1/YY1/RB1/HDAC1/PARP1/AR/KDR/STAT3/RCAN1
PID_CXCR4_PATHWAY	15/313	9.20E-08	RHOB/PTEN/FOXO1/CRK/SRC/RICTOR/STAT5A/RHOC/STAT3/PAK1/PIK3CG/AKT1/STAT1/MMP9/PIK3R3
WP_VITAMIN_D_RECEPTOR_PATHWAY	20/313	1.09E-07	CCNE1/CDKN1A/MYC/CCND1/SLC2A4/SATB1/FOXO1/CDKN2C/CDK2/KLF4/MXD1/LRRC8A/PRDM1/ID4/HNF1A/ABCB1/TNFAIP3/TGFB1/CDKN2B/SALL4
WP_GALANIN_RECEPTOR_PATHWAY	8/313	1.14E-07	CDKN1A/VAMP2/VEGFA/BCL2L11/SLC2A4/IL6/YAP1/FOS
WP_NONALCOHOLIC_FATTY_LIVER_DISEASE	18/313	1.89E-07	AKT3/CASP3/BCL2L11/IL1A/PRKAA1/IL6/SMAD7/SREBF1/CXCL8/IL6R/INSR/BAX/PPARA/CCL2/AKT1/SOCS3/TGFB1/PIK3R3

REACTOME_TRANSCRIPTIONAL_REGULATION_OF_PLURIPOTENT_STEM_CELLS	9/313	2.09E-07	FOXP1/NANOG/SOX2/SMAD4/KLF4/POU5F1/STAT3/SMAD2/SALL4
KEGG_MTOR_SIGNALING_PATHWAY	11/313	2.11E-07	AKT3/VEGFA/PRKAA1/STK11/RICTOR/MAPK1/IGF1/PIK3CG/AKT1/RPS6KA3/PIK3R3
PID_PTP1B_PATHWAY	11/313	2.11E-07	CRK/ITGB3/PDGFRB/SRC/STAT5A/CSF1R/INSR/EGFR/STAT3/AKT1/SOCS3
WP_NETRINUNC5B_SIGNALING_PATHWAY	11/313	2.11E-07	CASP3/MAP2K1/IL1A/TP53/SRC/YAP1/MAPK1/KDR/RAF1/CCL2/AKT1
PID_MET_PATHWAY	13/313	2.71E-07	MET/MAP2K1/CRKL/CRK/SNAI1/SRC/EGR1/MAPK1/CBL/NUMB/RAF1/PAK1/AKT1
WP_TNFRELATED_WEAK_INDUCER_OF_APOPTOSIS_TWEAK_SIGNALING_PATHWAY	10/313	2.72E-07	CASP3/CHUK/CCL5/HDAC1/IL6/MAPK1/RAF1/CCL2/AKT1/MMP9
WP_IL6_SIGNALING_PATHWAY	10/313	3.44E-07	MAP2K1/HDAC1/IL6/PRDM1/MAPK1/IL6R/STAT3/AKT1/SOCS3/STAT1
BIOCARTA_TPO_PATHWAY	8/313	3.63E-07	MAP2K1/RASA1/STAT5A/RAF1/STAT3/PIK3CG/STAT1/FOS
PID_ERBB2_ERBB3_PATHWAY	10/313	4.28E-07	MAP2K1/NRAS/SRC/MAPK1/RAF1/STAT3/ERBB2/AKT1/FOS/PIK3R3
WP_B_CELL_RECEPTOR_SIGNALING_PATHWAY	14/313	4.32E-07	MEF2C/MYC/E2F3/MAP2K1/CHUK/CRKL/FOXO1/CRK/MAPK1/CBL/RAF1/IKBKG/PIK3CG/AKT1
BIOCARTA_SPRY_PATHWAY	7/313	4.58E-07	MAP2K1/RASA1/MAPK1/CBL/EGFR/RAF1/SPRY1
BIOCARTA_TEL_PATHWAY	7/313	4.58E-07	MYC/BCL2/TP53/IGF1R/RB1/EGFR/AKT1
WP_LEPTININSULIN_SIGNALING_OVERLAP	7/313	4.58E-07	SOCS1/INSR/STAT3/PIK3CG/AKT1/SOCS3/PIK3R3

PID_MTOR_4PATHWAY	12/313	4.58E-07	CCNE1/YY1/MAP2K1/EIF4A1/NRAS/CDK2/RICTOR/MAPK1/SREBF1/RAF1/PDCD4/AKT1
WP_TH17_CELL_DIFFERENTIATION_PATHWAY	12/313	4.58E-07	ZAP70/IFNG/IL6/MAPK1/STAT5A/IL6R/STAT3/SOCS3/STAT1/FOS/TGFB1/SMAD2
BIOCARTA_BAD_PATHWAY	8/313	4.97E-07	BCL2/IGF1R/MAPK1/KIT/BAX/IGF1/PIK3CG/AKT1
WP_IL7_SIGNALING_PATHWAY	8/313	4.97E-07	MYC/CCND1/MAP2K1/MAPK1/STAT5A/STAT3/AKT1/STAT1
REACTOME_INTERLEUKIN_6_SIGNALING	6/313	5.20E-07	IL6/CBL/IL6R/STAT3/SOCS3/STAT1
WP_NEUROGENESIS_REGULATION_IN_THE_OLFACTORY_EPITHELIUM	11/313	5.29E-07	JAG1/MEF2C/MYC/DLL1/NOTCH1/SOX2/BDNF/RET/PAX6/NUMB/STAT3
REACTOME_TOLL_LIKE_RECEPTOR_TLR1_TLR2_CASCADE	15/313	5.46E-07	MEF2C/SOCS1/MAP2K1/CHUK/TP53/TLR2/MAPK1/IRAK4/IKBKG/TAB2/TAB3/FOS/NLRC5/RPS6KA3/HMGB1
KEGG_REGULATION_OF_AUTOPHAGY	9/313	5.97E-07	PRKAA1/IFNG/ATG4A/ATG4B/ATG4C/ATG4D/ATG5/ATG7/BECN1
PID_ERBB1_RECEPTOR_PROXIMAL_PATHWAY	9/313	5.97E-07	RASA1/NRAS/SRC/MAPK1/EGFR/STAT3/PAK1/STAT1/PIK3R3
REACTOME_TGF_BETA_RECEPTOR_SIGNALING_ACTIVATES_SMADS	10/313	6.29E-07	ITGB8/TGFBR2/SMAD4/ITGB3/SMAD7/SMURF1/CBL/TGFB1/SMAD2/PPP1CC
WP_NEURAL_CREST_DIFFERENTIATION	14/313	6.79E-07	WNT3A/MYB/MYC/RHOB/WNT1/AXIN2/DLL1/NOTCH1/NOTCH2/BMP7/HDAC1/SNAI1/SOX5/CTBP2
PID_HDAC_CLASSIII_PATHWAY	8/313	6.79E-07	SIRT1/CDKN1A/KAT2B/TP53/FOXO1/FOXO3/SIRT7/BAX

WP_SREBF_AND_MIR33_IN_CHOLESTEROL_AND_LIPID_HOMEOSTASIS	7/313	6.97E-07	SIRT1/PRKAA1/SREBF1/SREBF2/ABCA1/PPARA/SIRT6
PID_GMCSF_PATHWAY	9/313	7.57E-07	MAP2K1/NRAS/MAPK1/STAT5A/RAF1/STAT3/CCL2/STAT1/FOS
WP_THYMIC_STROMAL_LYMPHOPOIETIN_TSLP_SIGNALING_PATHWAY	10/313	7.60E-07	MYC/MAP2K1/IL6/SRC/MAPK1/STAT5A/CXCL8/STAT3/AKT1/STAT1
KEGG_APOPTOSIS	13/313	7.77E-07	BCL2/AKT3/CASP3/IL1A/CHUK/TP53/ATM/IRAK4/BAX/IKBKG/PIK3CG/AKT1/PIK3R3
BIOCARTA_EGF_PATHWAY	8/313	9.11E-07	MAP2K1/RASA1/STAT5A/EGFR/RAF1/STAT3/STAT1/FOS
BIOCARTA_ERK_PATHWAY	8/313	9.11E-07	MYC/MAP2K1/PDGFRA/IGF1R/MAPK1/EGFR/RAF1/STAT3
WP_NEOVASCULARISATION_PROCESSES	9/313	9.49E-07	JAG1/NOTCH1/MAPK1/KDR/KIT/AKT1/MMP9/TGFB1/SMAD2
WP_LTF_DANGER_SIGNAL_RESPONSE_PATHWAY	7/313	1.03E-06	IFNB1/IL1A/TLR2/IL6/MAPK1/CXCL8/IRAK4
REACTOME_SIGNALING_BY_VEGF	14/313	1.18E-06	AKT3/VEGFA/AXL/RASA1/CRK/NRAS/ITGB3/SRC/RICTOR/KDR/CYBB/PAK1/AKT1/CTNND1
WP_DYRK1A	11/313	1.19E-06	SIRT1/CDK4/CDK6/CCND1/DLL1/NOTCH1/CASP3/TP53/FOXO1/LATS2/E2F5
WP_CORTICOTROPINRELEASING_HORMONE_SIGNALING_PATHWAY	13/313	1.42E-06	BCL2/CASP3/MAP2K1/FOSL1/PARP1/MAPK1/CXCL8/SP1/RAF1/AKT1/FOS/TGFB1/NR4A2
WP_TNFALPHA_SIGNALING_PATHWAY	13/313	1.42E-06	CASP3/CHUK/IL6/NRAS/MAPK1/TNFAIP3/RAF1/BAX/IKBKG/TAB2/TAB3/CCL2/AKT1

REACTOME_SIGNALING_BY_KIT_IN_DISEASE	7/313	1.48E-06	NRAS/SRC/STAT5A/KIT/STAT3/STAT1/PIK3R3
WP_PDGFRBETA_PATHWAY	8/313	1.57E-06	MAP2K1/RASA1/PDGFRB/STAT5A/RAF1/STAT3/STAT1/FOS
SIG_INSULIN_RECEPTOR_PATHWAY_IN_CARDIAC_MYOCYTES	10/313	1.57E-06	AKT3/PTEN/SLC2A4/FOXO1/MAPK1/CBL/RAF1/AKT1/FLOT2/RPS6KA3
WP_REGULATORY_CIRCUITS_OF_THE_STAT3_SIGNALING_PATHWAY	12/313	1.58E-06	STMN1/PDGFRA/PDGFRB/SRC/RICTOR/MAPK1/IL6R/EGFR/STAT3/SOCS3/IL10RA/AGTR1
BIOCARTA_IL10_PATHWAY	6/313	1.58E-06	IL1A/IL6/STAT5A/STAT3/STAT1/IL10RA
WP_NEURAL_CREST_CELL_MIGRATION_DURING_DEVELOPMENT	9/313	1.80E-06	AKT3/BDNF/STAT3/PAK1/PIK3CG/AKT1/FOS/MMP9/PIK3R3
PID_LYSOPHOSPHOLIPID_PATHWAY	11/313	1.85E-06	CASP3/HBEGF/CRK/IL6/SRC/CXCL8/EGFR/AKT1/FOS/MMP9/PRKD1
PID_IL2_STAT5_PATHWAY	8/313	2.03E-06	CCND2/MYC/BCL2/CDK6/CCNA2/STAT5A/SP1/CCND3
WP_PI3KAKTMTOR_SIGNALING_PATHWAY_AND_THERAPEUTIC_OPPORTUNITIES	8/313	2.03E-06	PTEN/FOXO1/NRAS/RICTOR/FOXO3/PIK3CG/AKT1/PIK3R3
PID_BETA_CATENIN_NUC_PATHWAY	12/313	2.06E-06	CCND2/MYC/CCND1/AXIN2/LEF1/HDAC1/KLF4/AR/CXCL8/TCF7/MMP9/SALL4
BIOCARTA_IGF1_PATHWAY	7/313	2.06E-06	MAP2K1/IGF1R/RASA1/RAF1/IGF1/PIK3CG/FOS
BIOCARTA_IL6_PATHWAY	7/313	2.06E-06	MAP2K1/IL6/CEBPB/IL6R/RAF1/STAT3/FOS
BIOCARTA_INSULIN_PATHWAY	7/313	2.06E-06	MAP2K1/SLC2A4/RASA1/INSR/RAF1/PIK3CG/FOS

REACTOME_REGULATION_OF_GENE_EXPRESSION_IN_BETA_CELLS	7/313	2.06E-06	AKT3/HNF4A/FOXO1/HNF1A/PAX6/AKT1/HNF4G
PID_HDAC_CLASSI_PATHWAY	11/313	2.10E-06	SIRT1/YY1/KAT2B/MTA2/HDAC1/MXD1/SMAD7/SMURF1/SIRT7/STAT3/SIRT6
PID_HIF1_TFPATHWAY	11/313	2.10E-06	HNF4A/VEGFA/LDHA/HK2/SMAD4/EDN1/SP1/ABCB1/AKT1/FOS/SLC2A1
WP_CCL18_SIGNALING_PATHWAY	10/313	2.16E-06	MYC/PTEN/CD44/LDHA/SNAI1/HOTAIR/STAT1/MMP9/MTDH/SLC2A1
BIOCARTA_MAPK_PATHWAY	12/313	2.28E-06	MYC/MAP2K1/CHUK/MAPK1/SP1/RAF1/PAK1/RPS6KA4/STAT1/FOS/TGFB1/RPS6KA3
REACTOME_REPRESSION_OF_WNT_TARGET_GENES	6/313	2.55E-06	MYC/AXIN2/LEF1/HDAC1/TCF7/CTBP2
REACTOME_DOWNREGULATION_OF_SMAD2_3_SMAD4_TRANSCRIPTIONAL_ACTIVITY	8/313	2.55E-06	HDAC1/SMAD4/PARP1/SMAD7/MAPK1/TGIF2/STAT1/SMAD2
REACTOME_REGULATION_OF_BETA_CELL_DEVELOPMENT	9/313	2.65E-06	NOTCH1/AKT3/HNF4A/KAT2B/FOXO1/HNF1A/PAX6/AKT1/HNF4G
PID_P75_NTR_PATHWAY	11/313	2.77E-06	E2F1/RHOB/CASP3/BCL2L11/CHUK/TP53/BDNF/RHOC/IKBKG/AKT1/MMP3
NABA_SECRETED_FACTORS	25/313	2.95E-06	WNT3A/WNT1/VEGFA/IFNB1/IL1A/CCL5/BMP7/FGF7/LIF/IL13/HBEGF/ARTN/CCL22/IFNG/IL6/INHBB/BDNF/CXCL8/CCL3/CXCL2/CXCL10/IGF1/CCL2/TGFB1/GDF5
PID_FGF_PATHWAY	10/313	2.95E-06	MET/KLB/SRC/MAPK1/CBL/AKT1/STAT1/FOS/MMP9/CTNND1
REACTOME_INTRINSIC_PATHWAY_FOR_APOPTOSIS	10/313	2.95E-06	E2F1/BCL2/AKT3/CASP3/BCL2L11/TP53/MAPK1/BAX/STAT3/AKT1

WP_DNA_IRDOUBLE_STRAND_BREAKS_AND_CELLULAR_RESPONSE_VIA_ATM	10/313	2.95E-06	E2F1/BRCA1/CASP3/TP53/CHEK1/PARP1/YAP1/ATM/BAX/RAD51
WP_IL1_SIGNALING_PATHWAY	10/313	2.95E-06	MAP2K1/IL1A/CHUK/MAPK1/IRAK4/IKBKG/TAB2/TAB3/CCL2/AKT1
WP_RANKLRANK_SIGNALING_PATHWAY	10/313	2.95E-06	MAP2K1/CHUK/SRC/MAPK1/CBL/IKBKG/TAB2/AKT1/STAT1/FOS
PID_CXCR3_PATHWAY	9/313	3.13E-06	MAP2K1/NRAS/SRC/RICTOR/MAPK1/CXCL10/RAF1/AKT1/PIK3R3
WP_FIBRIN_COMPLEMENT_RECEPTOR_3_SIGNALING_PATHWAY	9/313	3.13E-06	IFNB1/CHUK/IL6/SRC/IRAK4/CXCL10/IKBKG/CCL2/AKT1
WP_REGUCALCIN_IN_PROXIMAL_TUBULE_EPITHELIAL_KIDNEY_CELLS	8/313	3.16E-06	CASP3/SMAD4/MAPK1/RAF1/BAX/AKT1/TGFB1/SMAD2
REACTOME_SIGNALING_BY_NTRKS	15/313	3.19E-06	MEF2C/MAP2K1/CRKL/FOSL1/CRK/NRAS/SRC/EGR1/BDNF/MAPK1/ID4/BAX/STAT3/FOS/RPS6KA3
WP_NANOPARTICLE_TRIGGERED_AUTOPHAGIC_CELL_DEATH	7/313	3.79E-06	BCL2/ATG16L1/INSR/ATG4A/ATG5/ATG7/BECN1
REACTOME_TFAP2_AP_2_FAMILY_REGULATES_TRANSCRIPTION_OF_GROWTH_FACTORS_AND_THEIR_RECEPTORS	6/313	3.89E-06	YY1/VEGFA/ESR1/KIT/EGFR/ERBB2
WP_RESISTIN_AS_A_REGULATOR_OF_INFLAMMATION	8/313	4.01E-06	AKT3/CHUK/IL6/MAPK1/CXCL8/IKBKG/PIK3CG/AKT1
REACTOME_SIGNALING_BY_PDGF	10/313	4.76E-06	CRKL/PDGFRA/RASA1/CRK/NRAS/PDGFRB/SRC/STAT5A/STAT3/STAT1
BIOCARTA_CTCF_PATHWAY	7/313	5.12E-06	MYC/PTEN/TGFBR2/TP53/SMAD4/PIK3CG/TGFB1

REACTOME_INTERLEUKIN_6_FAMILY_SIGNALING	7/313	5.12E-06	LIF/IL6/CBL/IL6R/STAT3/SOCS3/STAT1
WP_ANGIOGENESIS	7/313	5.12E-06	VEGFA/PDGFRA/SRC/MAPK1/KDR/AKT1/MMP9
WP_APOPTOSIS	12/313	5.19E-06	MYC/BCL2/CASP3/BCL2L11/CHUK/TP53/IGF1R/BIRC5/BAX/IGF1/IKBKG/AKT1
PID_PS1_PATHWAY	9/313	5.49E-06	MYC/CCND1/WNT1/DLL1/NOTCH1/HDAC1/MAPK1/HNF1A/FOS
WP_TRANSLATION_INHIBITORS_IN_CHRONICALLY_ACTIVATED_PDGFRA_CELLS	9/313	5.49E-06	AKT3/MAP2K1/EIF4A1/MAPK1/RPS6KA4/PDCD4/PIK3CG/AKT1/PIK3R3
WP_H19_ACTION_RBE2F1_SIGNALING_AND_CDKBETACATENIN_ACTIVITY	6/313	5.90E-06	JAG1/E2F1/CDK4/CCND1/SOX4/RB1
REACTOME_TOLL_LIKE_RECEPTOR_9_TLR9_CASCADE	13/313	6.19E-06	MEF2C/MAP2K1/CHUK/TP53/MAPK1/IRAK4/IKBKG/TAB2/TAB3/FOS/NLRC5/RPS6KA3/HMGB1
KEGG_TYPE_II_DIABETES_MELLITUS	9/313	6.56E-06	SOCS1/SOCS4/SLC2A4/HK2/MAPK1/INSR/PIK3CG/SOCS3/PIK3R3
KEGG_B_CELL_RECEPTOR_SIGNALING_PATHWAY	11/313	6.93E-06	AKT3/MAP2K1/CHUK/NRAS/MAPK1/RAF1/IKBKG/PIK3CG/AKT1/FOS/PIK3R3
PID_AR_PATHWAY	10/313	7.40E-06	CDK6/CCND1/BRCA1/LATS2/AR/CTDSP2/CARM1/CCND3/AKT1/RPS6KA3
REACTOME_REGULATION_OF_PTEN_GENE_TRANSCRIPTION	10/313	7.40E-06	BMI1/PTEN/EZH2/TP53/MTA2/HDAC1/SNAI1/EGR1/MAPK1/SALL4
PID_AJDISS_2PATHWAY	9/313	7.79E-06	MET/CASP3/IGF1R/SRC/BDNF/RET/EGFR/CTNND1/MMP3
REACTOME_OXIDATIVE_STRESS_INDUCED_SENESCENCE	14/313	7.79E-06	BMI1/E2F1/CDK4/CDK6/E2F2/E2F3/MDM4/IFNB1/EZH2/TP53/CDKN2C/MAPK1/FOS/CDKN2B

KEGG_VEGF_SIGNALING_PATHWAY	11/313	7.79E-06	AKT3/VEGFA/MAP2K1/NRAS/SRC/MAPK1/KDR/RAF1/PIK3CG/AKT1/PIK3R3
REACTOME_REGULATION_OF_RUNX1_EXPRESSION_AND_ACTIVITY	6/313	8.56E-06	CCND2/CDK6/CCND1/SRC/AGO2/CCND3
WP_IL9_SIGNALING_PATHWAY	6/313	8.56E-06	MAP2K1/MAPK1/STAT5A/STAT3/STAT1/CDK9
WP_CYTOKINES_AND_INFLAMMATORY_RESPONSE	7/313	8.72E-06	IFNB1/IL1A/IL13/IFNG/IL6/CXCL2/TGFB1
WP_THYROID_HORMONES_PRODUCTION_AND_PERIPHERAL_DOWNSTREAM_SIGNALING_EFFECTS	12/313	8.84E-06	NOTCH1/AKT3/MAP2K1/TP53/FOXO1/KLB/ITGB3/SRC/SLC16A2/MAPK1/RAF1/SIRT6
PID_ANGIOPOIETIN_RECEPTOR_PATHWAY	9/313	8.97E-06	CDKN1A/FOXO1/RASA1/CRK/MAPK1/STAT5A/PAK1/AKT1/AGTR1
PID_FOXO_PATHWAY	9/313	8.97E-06	SIRT1/KAT2B/BCL2L11/CHUK/FOXO1/CCNB1/CDK2/FOXO3/AKT1
WP_INTERLEUKIN1_IL1_STRUCTURAL_PATHWAY	9/313	8.97E-06	MYC/MAP2K1/IL1A/CHUK/MAPK1/IRAK4/TAB2/TAB3/FOS
REACTOME_TOLL_LIKE_RECEPTOR_CASCADES	16/313	9.04E-06	MEF2C/SOCS1/MAP2K1/CHUK/TP53/TLR2/HSP90B1/MAPK1/IRAK4/IKBKG/TAB2/TAB3/FOS/NLRC5/RPS6KA3/HMGB1
BIOCARTA_ALK_PATHWAY	8/313	9.28E-06	MEF2C/WNT1/TGFBR2/BMPR2/BMP7/SMAD4/HNF1A/TGFB1
REACTOME_DEUBIQUITINATION	22/313	1.08E-05	CDC25A/MYC/AXIN2/BRCA1/PTEN/YY1/ESR1/KAT2B/MDM4/TGFBR2/TP53/CCNA2/SMAD4/AR/SMAD7/CFTR/TNFAIP3/IKBKG/BECN1/NLRP3/TGFB1/SMAD2
PID_ERA_GENOMIC_PATHWAY	10/313	1.10E-05	MYC/CCND1/AXIN2/BRCA1/ESR1/CHUK/HDAC1/SMAD4/CEBPB/STAT5A

PID_IL4_2PATHWAY	10/313	1.10E-05	MYB/SOCS1/HMGA1/ITGB3/CEBPB/CBL/STAT5A/SP1/AKT1/SOCS3
WP_FGFR3_SIGNALING_IN_CHONDROCYTE_PROLIFERATION_AND_TERMINAL_DIFFERENTIATION	7/313	1.11E-05	CDKN1A/MAP2K1/SNAI1/MAPK1/RAF1/ATG5/STAT1
REACTOME_TRANSCRIPTIONAL_REGULATION_BY_THE_AP_2_TFAP2_FAMILY_OF_TRANSCRIPTION_FACTORS	8/313	1.13E-05	CDKN1A/MYC/YY1/VEGFA/ESR1/KIT/EGFR/ERBB2
BIOCARTA_NFAT_PATHWAY	9/313	1.23E-05	MEF2C/MAP2K1/HBEGF/MAPK1/EDN1/RAF1/IGF1/PIK3CG/AKT1
KEGG_WNT_SIGNALING_PATHWAY	15/313	1.27E-05	WNT3A/CCND2/MYC/CCND1/WNT1/AXIN2/TP53/FOSL1/PPP2R5E/LEF1/SMAD4/TCF7/CCND3/CTBP2/SMAD2
WP_DNA_IRDAMAGE_AND_CELLULAR_RESPONSE_VIA_ATR	11/313	1.38E-05	E2F1/BRCA1/TP53/CHEK1/PARP1/CDK2/ATM/PPM1D/SP1/IKBKG/RAD51
WP_ANGIOTENSIN_II_RECEPTOR_TYPE_1_PATHWAY	7/313	1.41E-05	TGFBR2/SMAD4/MAPK1/SP1/RAF1/TGFB1/AGTR1
WP_NANOPARTICLEMEDIATED_ACTIVATION_OF_RECEPTOR_SIGNALING	7/313	1.41E-05	AKT3/MAP2K1/NRAS/SRC/MAPK1/EGFR/RAF1
WP_SPHINGOLIPID_METABOLISM_IN_SENESCENCE	7/313	1.41E-05	CDKN1A/E2F1/CDK4/RB1/CERS2/CDK2/MAPK1
WP_TLR4_SIGNALING_AND_TOLERANCE	7/313	1.41E-05	IFNB1/CHUK/IL6/CXCL8/IRAK4/IKBKG/TAB2
WP_NAD_METABOLISM_SIRTUINS_AND_AGING	5/313	1.42E-05	SIRT1/FOXO1/PARP1/NAMPT/FOXO3

BIOCARTA_CYTOKINE_PATHWAY	6/313	1.65E-05	IFNB1/IL1A/IL13/IFNG/IL6/CXCL8
BIOCARTA_TGFB_PATHWAY	6/313	1.65E-05	MAP2K1/TGFBR2/SMAD4/SMAD7/TGFB1/SMAD2
WP_OVERVIEW_OF_NANOPARTICLE_EFFECTS	6/313	1.65E-05	BCL2/AKT3/IL6/CXCL8/BAX/CCND3
WP_COPPER_HOMEOSTASIS	9/313	1.66E-05	CCND1/PTEN/CASP3/TP53/FOXO1/FOXO3/SP1/AKT1/SLC31A1
BIOCARTA_INFLAM_PATHWAY	7/313	1.78E-05	IFNB1/IL1A/IL13/IFNG/IL6/CXCL8/TGFB1
WP_MIRNAS_INVOLVED_IN_DNA_DAMAGE_RESPONSE	10/313	1.81E-05	CCNE1/CDC25A/CDKN1A/E2F1/MYC/CDK6/CCND1/TP53/ATM/CCND3
REACTOME_TRANSCRIPTIONAL_REGULATION_OF_WHITE_ADIPOCYTE_DIFFERENTIATION	11/313	1.90E-05	CDK4/WNT1/SLC2A4/KLF4/CARM1/CEBPB/SREBF1/SREBF2/PPARA/CCND3/TGFB1
PID_TGFBR_PATHWAY	9/313	1.92E-05	TGFBR2/SMAD4/DAB2/SMAD7/YAP1/SMURF1/TAB2/TGFB1/SMAD2
PID_VEGFR1_2_PATHWAY	10/313	2.06E-05	VEGFA/MAP2K1/PRKAA1/ITGB3/SRC/MAPK1/KDR/CBL/RAF1/AKT1
BIOCARTA_ATM_PATHWAY	6/313	2.25E-05	CDKN1A/BRCA1/TP53/CHEK1/ATM/RAD51
BIOCARTA_CBL_PATHWAY	5/313	2.29E-05	MET/PDGFRA/CBL/CSF1R/EGFR
BIOCARTA_HES_PATHWAY	5/313	2.29E-05	WNT1/DLL1/NOTCH1/NOTCH2/HNF1A
WP_IL10_ANTIINFLAMMATORY_SIGNALING_PATHWAY	5/313	2.29E-05	IL1A/IL6/STAT3/STAT1/IL10RA
PID_BMP_PATHWAY	8/313	2.32E-05	BMPR2/BMP7/SMAD4/SMAD7/CTDSP2/SMURF1/MAPK1/TAB2
PID_UPA_UPAR_PATHWAY	8/313	2.32E-05	CRK/ITGB3/PDGFRB/SRC/EGFR/MMP9/TGFB1/MMP3

PID_AR_NONGENOMIC_PATHWAY	7/313	2.79E-05	MAP2K1/AR/SRC/MAPK1/RAF1/AKT1/FOS
REACTOME_SIGNAL_TRANSDUCTION_BY_L1	6/313	3.01E-05	MAP2K1/ITGB3/MAPK1/L1CAM/EGFR/PAK1
WP_HEMATOPOIETIC_STEM_CELL_GENE_REGULATION_BY_GABP_ALPHABETA_COMPLEX	6/313	3.01E-05	BCL2/PTEN/DNMT1/SMAD4/ATM/FOXO3
PID_HNF3A_PATHWAY	8/313	3.30E-05	BRCA1/ESR1/NFIA/AR/CEBPB/SP1/FOS/FOXA1
REACTOME_SIGNALING_BY_ERBB4	9/313	3.41E-05	STMN1/ESR1/HBEGF/NRAS/SRC/YAP1/STAT5A/EGFR/TAB2
REACTOME_NUCLEAR_SIGNALING_BY_ERBB4	7/313	3.44E-05	STMN1/ESR1/HBEGF/SRC/YAP1/STAT5A/TAB2
REACTOME_POU5F1_OCT4_SOX2_NANOG_ACTIVATE_GENES_RELATED_TO_PROLIFERATION	5/313	3.54E-05	NANOG/SOX2/POU5F1/STAT3/SALL4
REACTOME_TRANSCRIPTIONAL_REGULATION_OF_GRANULOPOIESIS	11/313	3.57E-05	MYB/CDKN1A/E2F1/MYC/CDK4/LEF1/CDK2/TAL1/CEBPB/IL6R/STAT3
REACTOME_MYD88_INDEPENDENT_TLR4_CASCADE	12/313	3.74E-05	MEF2C/MAP2K1/CHUK/TP53/MAPK1/IKBKG/TAB2/TAB3/FOS/NLRC5/RPS6KA3/HMGB1
PID_AVB3_INTEGRIN_PATHWAY	10/313	3.74E-05	VEGFA/TGFBR2/IGF1R/ITGB3/SRC/MAPK1/KDR/CBL/CSF1R/AKT1
PID_NFAT_TFPATHWAY	8/313	3.84E-05	E2F1/CDK4/CASP3/FOSL1/IFNG/EGR1/CXCL8/FOS
WP_NOTCH_SIGNALING	8/313	3.84E-05	JAG1/DLL1/NOTCH1/KAT2B/NOTCH2/HDAC1/NUMB/CTBP2
BIOCARTA_AKT_PATHWAY	6/313	3.94E-05	CHUK/FOXO1/FOXO3/IKBKG/PIK3CG/AKT1

PID_SYNDECAN_2_PATHWAY	7/313	4.17E-05	CASP3/RASA1/SRC/MAPK1/CXCL8/BAX/TGFB1
WP_NONGENOMIC_ACTIONS_OF_125_DIHYDROXYVITAMIN_D3	10/313	4.17E-05	SP3/TLR2/IFNG/IL6/NRAS/MAPK1/CXCL8/SP1/CCL2/STAT1
PID_FCER1_PATHWAY	9/313	4.40E-05	MAP2K1/CHUK/RASA1/MAPK1/CBL/RAF1/IKBKG/AKT1/FOS
BIOCARTA_KERATINOCYTE_PATHWAY	8/313	4.47E-05	BCL2/MAP2K1/CHUK/MAPK1/SP1/EGFR/RAF1/FOS
WP_MICROTUBULE_CYTOSKELETON_REGULATION	8/313	4.47E-05	STMN1/WNT3A/PTEN/SPRED1/SRC/STAT3/PAK1/AKT1
KEGG_REGULATION_OF_ACTIN_CYTOSKELETON	17/313	4.78E-05	ITGB8/MAP2K1/FGF7/CRKL/PDGFRA/CRK/NRAS/ITGB3/PDGFRB/MAPK1/MYL9/EGFR/RAF1/PAK1/PIK3CG/PIK3R3/PPP1CC
PID_TRKR_PATHWAY	9/313	4.99E-05	CCND1/MAP2K1/CRKL/RASA1/CRK/NRAS/BDNF/MAPK1/STAT3
PID_MAPK_TRK_PATHWAY	7/313	5.05E-05	MEF2C/MAP2K1/NRAS/EGR1/MAPK1/RAF1/FOS
PID_TCR_RAS_PATHWAY	5/313	5.15E-05	MAP2K1/NRAS/MAPK1/RAF1/FOS
REACTOME_FORMATION_OF_AXIAL_MESODERM	5/313	5.15E-05	LEF1/YAP1/TCF7/SMAD2/FOXA1
KEGG_NOTCH_SIGNALING_PATHWAY	8/313	5.18E-05	JAG1/DLL1/NOTCH1/KAT2B/NOTCH2/HDAC1/NUMB/CTBP2
REACTOME_TRANSCRIPTIONAL_REGULATION_BY_RUNX1	18/313	5.73E-05	LMO2/BMI1/MYB/CCND2/CDK6/CCND1/ESR1/KAT2B/SOCS4/IFNG/HDAC1/SRC/TAL1/YAP1/AGO2/MYL9/CCND3/SOCS3
WP_ENERGY_METABOLISM	8/313	6.05E-05	MEF2C/SIRT1/UCP2/FOXO1/PRKAA1/HDAC1/FOXO3/PPARA
PID_BCR_5PATHWAY	9/313	6.39E-05	PTEN/MAP2K1/CHUK/RASA1/MAPK1/RAF1/IKBKG/AKT1/FOS
REACTOME_TRANSCRIPTIONAL_REGULATION_BY_MECP2	9/313	6.39E-05	MEF2C/MET/DLL1/NOTCH1/PTEN/SOX2/HDAC1/BDNF/AGO2

BIOCARTA_WNT_PATHWAY	6/313	6.47E-05	MYC/CCND1/WNT1/HDAC1/SMAD4/HNF1A
REACTOME_GROWTH_HORMONE_RECEPTOR_SIGNALING	6/313	6.47E-05	SOCS1/MAPK1/STAT5A/STAT3/SOCS3/STAT1
WP_IL1_AND_MEGAKARYOCYTES_IN_OBESITY	6/313	6.47E-05	TLR2/HBEGF/IFNG/CCL2/NLRP3/MMP9
REACTOME_DNA_DAMAGE_TELOMERE_STRESS_INDUCED_SENESCENCE	10/313	7.10E-05	CCNE1/CDKN1A/CCNE2/HMGA2/TP53/RB1/HMGA1/CCNA2/CDK2/ATM
PID_PI3K_PLC_TRK_PATHWAY	7/313	7.26E-05	CCND1/NRAS/SRC/EGR1/STAT5A/FOXO3/AKT1
WP_FACTORS_AND_PATHWAYS_AFFECTING_INSULINLIKE_GROWTH_FACTOR_IGF1AKT_SIGNALING	7/313	7.26E-05	PTEN/IGF1R/RICTOR/IGF1/PIK3CG/AKT1/SMAD2
BIOCARTA_BCELLSURVIVAL_PATHWAY	5/313	7.26E-05	CASP3/BIRC5/PIK3CG/AKT1/FOS
BIOCARTA_ERYTH_PATHWAY	5/313	7.26E-05	IL1A/IL6/CCL3/IGF1/TGFB1
BIOCARTA_LONGEVITY_PATHWAY	5/313	7.26E-05	IGF1R/FOXO3/IGF1/PIK3CG/AKT1
WP_LET7_INHIBITION_OF_ES_CELL_REPROGRAMMING	5/313	7.26E-05	MYC/SOX2/KLF4/EGR1/POU5F1
REACTOME_SIGNALING_BY_ERBB2	8/313	7.98E-05	AKT3/CUL5/HBEGF/NRAS/SRC/EGFR/ERBB2/AKT1
REACTOME_INACTIVATION_OF_CSF3_G_CSF_SIGNALING	6/313	8.14E-05	SOCS1/CUL5/STAT5A/STAT3/SOCS3/STAT1

REACTOME_SIGNALING_BY_NOTCH	18/313	8.53E-05	JAG1/E2F1/MYC/CCND1/E2F3/DLL1/NOTCH1/KAT2B/NOTCH2/TP53/FBXW7/HDAC1/AGO2/NUMB/EGFR/AKT1/STAT1/SIRT6
PID_IL23_PATHWAY	7/313	8.53E-05	IFNG/IL6/STAT5A/STAT3/CCL2/SOCS3/STAT1
WP_TYPE_II_INTERFERON_SIGNALING	7/313	8.53E-05	SOCS1/IFNB1/IFNG/CYBB/CXCL10/SOCS3/STAT1
REACTOME_PTEN_REGULATION	13/313	9.36E-05	BMI1/AKT3/PTEN/EZH2/TP53/MTA2/HDAC1/SNAI1/EGR1/MAPK1/AGO2/AKT1/SALL4

❖ **Pathways targeted by upregulated miRNAs:**

Pathway	GeneRatio	p.adjust	geneID
WP_EGFR_TYROSINE_KINASE_INHIBITOR_RESISTANCE	30/218	3.87E-30	CCND1/STAT3/MET/NRAS/BCL2/VEGFA/EGFR/MYC/BCL2L11/PTEN/HRAS/RAF1/JAK1/PDGFRA/PDGFRB/IGF1R/RPS6KB1/FGF2/BAX/AKT2/IL6R/MAP2K1/EIF4E/IGF1/MTOR/PIK3R1/AXL/HGF/AKT3/KDR
WP_PI3KAKT_SIGNALING_PATHWAY	46/218	4.49E-27	CDK6/CCND1/CDKN1A/MET/CDK4/CCNE2/RELN/CCND2/NRAS/BCL2/VEGFA/EGFR/MYC/BCL2L11/IFNB1/EPHA2/PTEN/RBL2/HRAS/RAF1/CCND3/CCNE1/CHUK/JAK1/VEGFC/CSF1/PDGFRA/PDGFRB/IGF1R/MYB/RPS6KB1/FGF2/AKT2/BRCA1/TLR4/INSR/IL6R/MAP2K1/COL1A2/EIF4E/IGF1/MTOR/PIK3R1/HGF/AKT3/KDR
WP_FOCAL_ADHESION_PI3KAKTMTORSIGNALING_PATHWAY	38/218	5.06E-21	HIF1A/CDKN1A/MET/RELN/NRAS/VEGFA/EGFR/IFNB1/EPHA2/PTEN/HRAS/RAF1/SLC2A3/CAB39/JAK1/VEGFC/IRS2/CSF1/PDGFRA/PDGFRB/IGF1R/COL3A1/SREBF1/RPS6KB1/FGF2/AKT2/INSR/IL6R/MAP2K1/COL1A2/EIF4E/IGF1/SLC2A4/MTOR/PIK3R1/HGF/AKT3/KDR
WP_DNA_DAMAGE_RESPONSE_ONLY_ATM_DEPENDENT	24/218	1.08E-18	CCND1/CDKN1A/CCND2/NRAS/BCL2/MYC/BCL2L11/PTEN/RBL2/HRAS/CCND3/CDC42/APC/CTNNB1/LEF1/MAPK9/WNT7A/PDK1/BAX/AKT2/INSR/FOSL1/PIK3R1/AKT3
WP_TGFBETA_SIGNALING_PATHWAY	25/218	5.62E-18	CCND1/ITCH/APP/CDKN1A/MET/MYC/TGFBR1/RBL1/RBL2/RAF1/RUNX2/CDC42/E2F5/SMAD7/MAPK9/TAB1/PDK1/BTRC/MAPK14/MAP2K1/SMAD2/COL1A2/DAB2/PIK3R1/YAP1
KEGG_FOCAL_ADHESION	28/218	1.10E-16	CCND1/MET/RELN/CCND2/BCL2/VEGFA/EGFR/PTEN/HRAS/RAF1/CCND3/CDC42/VEGFC/PDGFRA/PDGFRB/CTNNB1/IGF1R/COL3A1/MAPK9/AKT2/MAP2K1/COL1A2/FYN/IGF1/PIK3R1/HGF/AKT3/KDR
WP_FOCAL_ADHESION	27/218	8.29E-16	CCND1/MET/RELN/CCND2/BCL2/VEGFA/EGFR/PTEN/HRAS/RAF1/CCND3/CDC42/VEGFC/PDGFRA/PDGFRB/CTNNB1/IGF1R/MAPK9/AKT2/MAP2K1/COL1A2/FYN/IGF1/PIK3R1/HGF/AKT3/KDR
WP_ALZHEIMERS_DISEASE	30/218	1.46E-15	APP/NRAS/PTGS2/HRAS/RAF1/APC/CHUK/MAPT/CASP7/IRS2/CSF1/CTNNB1/AXIN2/MAPK9/WNT7A/FADD/ATG14/CASP8/AKT2/INSR/ITPR1/MAP2K1/RB1CC1/ULK2/MTOR/PIK3R1/FZD6/SNCA/CYBB/AKT3
WP_DNA_DAMAGE_RESPONSE	17/218	2.42E-14	CDK6/CCND1/CDKN1A/CDK4/CCNE2/CDC25A/E2F1/CCND2/MYC/RB1/CCND3/CCNE1/CASP8/CHEK1/BAX/BRCA1/TNFRSF10B

REACTOME_DISEASES_OF_SIGNAL_TRANSDUCTION_BY_GROWTH_FACTOR_RECEPTORS_AND_SECOND_MESSENGERS	35/218	1.00E-13	STAT3/CDKN1A/MET/NRAS/ESR1/KAT2B/EGFR/MYC/BCL2L11/TGFBR1/FBXW7/PTEN/HRAS/RAF1/NOTCH1/APC/CHUK/IRS2/PDGFRA/PDGFRB/CTNNB1/FGF2/AKT2/MAP2K1/SMAD2/FYN/CDK8/MTOR/RICTOR/PIK3R1/HGF/JAG1/FZD6/AKT3/KDR
WP_LEPTIN_SIGNALING_PATHWAY	17/218	1.28E-13	CCND1/STAT3/ESR1/PTEN/HRAS/RAF1/CDC42/CHUK/JAK1/RPS6KB1/BAX/MAPK14/MAP2K1/EIF4E/FYN/MTOR/PIK3R1
WP_ALZHEIMERS_DISEASE_AND_MIRNA_EFFECTS	30/218	4.17E-13	APP/NRAS/PTGS2/HRAS/RAF1/APC/CHUK/MAPT/CASP7/IRS2/CSF1/CTNNB1/AXIN2/MAPK9/WNT7A/FADD/ATG14/CASP8/AKT2/INSR/ITPR1/MAP2K1/RB1CC1/ULK2/MTOR/PIK3R1/FZD6/SNCA/CYBB/AKT3
REACTOME_INTERLEUKIN_4_AND_INTERLEUKIN_13_SIGNALING	19/218	5.12E-13	CCND1/STAT3/HIF1A/CDKN1A/BCL2/VEGFA/MYC/PTGS2/SOX2/NANOG/JAK1/TWIST1/BIRC5/FGF2/MMP2/IL6R/COL1A2/PIK3R1/HGF
REACTOME_INTRACELLULAR_SIGNALING_BY_SECOND_MESSENGERS	29/218	5.24E-13	CDKN1A/MET/PPARG/ESR1/EGFR/NR2E1/BMI1/PTEN/CHUK/AGO1/IRS2/SNAI1/SUZ12/PDGFRA/PDGFRB/SNAI2/EZH2/KPNA2/FGF2/AKT2/INSR/ITPR1/CBX4/FYN/MTOR/RICTOR/PIK3R1/HGF/AKT3
WP_BRAINDERIVED_NEUROTROPHIC_FACTOR_BDNF_SIGNALING_PATHWAY	21/218	5.24E-13	NTRK3/STAT3/IGF2BP1/BCL2L11/HRAS/RAF1/CDC42/APC/CHUK/MAPT/IRS2/CTNNB1/MAPK9/RPS6KB1/MAPK14/MAP2K1/EIF4E/FYN/MARCKS/MTOR/PIK3R1
WP_TROP2_REGULATORY_SIGNALING	14/218	7.66E-13	CCND1/CDK4/BCL2/EGFR/RB1/PTEN/CCNE1/NOTCH1/CTNNB1/IGF1R/CHEK1/BAX/IGF1/PIK3R1
REACTOME_SIGNALING_BY_VEGF	18/218	2.40E-12	NRAS/VEGFA/HRAS/CDC42/VEGFC/CTNNB1/AKT2/MAPK14/ITPR1/FYN/NCKAP1/MTOR/RICTOR/PIK3R1/AXL/CYBB/AKT3/KDR
WP_RAC1PAK1P38MMP2_PATHWAY	15/218	5.15E-12	STAT3/NRAS/EGFR/MYC/HRAS/CHUK/CASP7/CTNNB1/MAPK9/BIRC5/BAX/MMP2/MAPK14/PIK3R1/YAP1
WP_MIRNA_REGULATION_OF_DNA_DAMAGE_RESPONSE	17/218	7.74E-12	CDK6/CCND1/CDKN1A/CDK4/CCNE2/CDC25A/E2F1/CCND2/MYC/RB1/CCND3/CCNE1/CASP8/CHEK1/BAX/BRCA1/TNFRSF10B

REACTOME_SIGNALING_BY_NUCLEAR_RECEPTORS	27/218	8.80E-12	CCND1/RDH10/MYLIP/NRAS/BCL2/ESR1/KAT2B/EGFR/MYC/HRAS/ABCA1/ARL4C/AGO1/FASN/YY1/IGF1R/SREBF1/ABCG1/RXRA/RARG/MYB/KPNA2/PDK1/AKT2/MMP2/PIK3R1/AKT3
WP_EXTRACELLULAR_VESICLE MEDIATED_SIGNALING_IN_RECIPIENT_CELLS	11/218	1.72E-11	MET/NRAS/EGFR/TGFBR1/HRAS/RAF1/APC/CTNNB1/SMAD2/MTOR/HGF
REACTOME_SIGNALING_BY_INTERLEUKINS	33/218	2.97E-11	CCND1/APP/STAT3/HIF1A/CDKN1A/BCL2/VEGFA/MYC/PTGS2/CCL4/SOX2/NANOG/CDC42/CHUK/TAB3/JAK1/IRS2/CSF1/MAPK9/TWIST1/BIRC5/TAB1/CASP8/FGF2/BTRC/MMP2/MAPK14/IL6R/MAP2K1/COL1A2/FYN/PIK3R1/HGF
KEGG_MTOR_SIGNALING_PATHWAY	13/218	3.25E-11	HIF1A/VEGFA/CAB39/VEGFC/RPS6KB1/AKT2/ULK2/EIF4E/IGF1/MTOR/RICTOR/PIK3R1/AKT3
PID_TCPTP_PATHWAY	12/218	4.09E-11	STAT3/MET/VEGFA/EGFR/JAK1/CSF1/PDGFRB/KPNA2/INSR/PIK3R1/HGF/KDR
WP_IL2_SIGNALING_PATHWAY	12/218	4.09E-11	STAT3/CCND2/BCL2/MYC/HRAS/RAF1/MAPT/JAK1/RPS6KB1/MAP2K1/FYN/PIK3R1
WP_AGERAGE_PATHWAY	14/218	4.77E-11	STAT3/HIF1A/EGFR/RAF1/CDC42/CHUK/MAPK9/CASP8/MMP2/INSR/MAPK14/MAP2K1/SMAD2/LGALS3
PID_IL2_1PATHWAY	13/218	6.59E-11	STAT3/NRAS/BCL2/MYC/HRAS/RAF1/JAK1/IRS2/MAPK9/MAPK14/MAP2K1/FYN/PIK3R1
WP_TGFBETA_RECEPTOR_SIGNALING	13/218	6.59E-11	STAT3/BAMBI/TGFBR1/HRAS/RUNX2/JAK1/SMAD7/CTNNB1/LEF1/MAPK9/RUNX3/SMAD1/SMAD2
WP_INTERLEUKIN11_SIGNALING_PATHWAY	12/218	7.03E-11	STAT3/BCL2/HRAS/RAF1/RUNX2/CHUK/JAK1/BIRC5/RPS6KB1/MAP2K1/FYN/PIK3R1
PID_SMAD2_3NUCLEAR_PATHWAY	15/218	7.08E-11	CDKN1A/CDK4/ESR1/KAT2B/MYC/IFNB1/RBL1/RUNX2/HNF4A/E2F5/SMAD7/RUNX3/SMAD2/COL1A2/TGIF2
WP_ADIPOGENESIS	18/218	7.38E-11	STAT3/HIF1A/CDKN1A/HMGA1/E2F1/PPARG/RB1/RBL1/RBL2/GATA4/IRS2/CTNNB1/SREBF1/RXRA/TWIST1/NAMPT/IGF1/SLC2A4
WP_ANGIOPOIETINLIKE_PROTEIN_8_REGULATORY_PATHWAY	18/218	8.33E-11	HRAS/RAF1/IRS2/FASN/MAPK9/SREBF1/SREBF2/RXRA/RPS6KB1/AKT2/INSR/MAPK14/MAP2K1/EIF4E/SLC2A4/MTOR/RICTOR/PIK3R1
PID_SHP2_PATHWAY	13/218	1.01E-10	NTRK3/NRAS/VEGFA/EGFR/HRAS/RAF1/JAK1/PDGFRB/IGF1R/IL6R/MAP2K1/PIK3R1/KDR

KEGG_ADHERENS_JUNCTION	14/218	1.79E-10	MET/EGFR/TGFBR1/CDC42/SNAI1/CTNNB1/LEF1/IGF1R/SNAI2/INSR/NECTIN4/SMAD2/FYN/TJP1
WP_NEOVASCULARISATION_PROCESSES	11/218	1.80E-10	HIF1A/TGFBR1/NOTCH4/EPHB4/NOTCH1/MAPK9/EPHB2/SMAD1/SMAD2/JAG1/KDR
WP_VEGFAVEGFR2_SIGNALING	30/218	2.72E-10	CCND1/ITCH/STAT3/BCL2/VEGFA/PTGS2/EPHA2/HRAS/RAF1/NOTCH4/CDC42/CTNNB1/MAPK9/BIRC5/FADD/RPS6KB1/MMP2/PABPC1/MAPK14/EPHB2/MAP2K1/RHOC/EIF4E/FYN/MTOR/RICTOR/PIK3R1/JAG1/CYBB/KDR
WP_ERBB_SIGNALING_PATHWAY	15/218	3.45E-10	CCND1/CDKN1A/NRAS/EGFR/MYC/BCL2L11/HRAS/RAF1/MAPK9/RPS6KB1/AKT2/MAP2K1/MTOR/PIK3R1/AKT3
PID_MTOR_4PATHWAY	13/218	1.12E-09	NRAS/HRAS/RAF1/CCNE1/YY1/SREBF1/RPS6KB1/MAP2K1/RB1CC1/ULK2/EIF4E/MTOR/RICTOR
PID_CMYB_PATHWAY	14/218	1.13E-09	CDK6/CCND1/CDKN1A/NRAS/BCL2/MYC/PTGS2/HRAS/ADORA2B/LEF1/TAB1/MYB/CBX4/COL1A2
WP_WNT_SIGNALING_PATHWAY_AND_PLURIPOTENCY	15/218	1.27E-09	CCND1/CCND2/MYC/SOX2/NANOG/CCND3/APC/CTNNB1/LEF1/AXIN2/MAPK9/WNT7A/FOSL1/NKD1/FZD6
REACTOME_APOPTOSIS	19/218	1.47E-09	STAT3/E2F1/BCL2/BCL2L11/APC/MAPT/CASP7/BMF/CTNNB1/FADD/CASP8/BAX/AKT2/TLR4/TNFRSF10B/SPTAN1/TJP1/TNFRSF10A/AKT3
WP_APOPTOSIS	14/218	2.11E-09	BCL2/MYC/BCL2L11/CHUK/BCL2L2/CASP7/IGF1R/BIRC5/FADD/CASP8/BAX/TNFRSF10B/IGF1/PIK3R1
WP_KIT_RECEPTOR_SIGNALING_PATHWAY	12/218	2.27E-09	STAT3/BCL2/HRAS/RAF1/MAPT/SNAI1/SNAI2/RPS6KB1/MAPK14/MAP2K1/FYN/PIK3R1
WP_RAS_SIGNALING	19/218	2.29E-09	MET/NRAS/EGFR/EPHA2/ZAP70/HRAS/RAF1/CDC42/CHUK/PDGFRA/PDGFRB/IGF1R/MAPK9/AKT2/INSR/MAP2K1/PIK3R1/AKT3/KDR
PID_HES_HEY_PATHWAY	11/218	3.34E-09	STAT3/HIF1A/E2F1/RB1/RUNX2/NOTCH1/GATA4/YY1/TWIST1/MYB/KDR
WP_NOTCH_SIGNALING_PATHWAY	12/218	3.34E-09	CCND1/ITCH/STAT3/HIF1A/CDKN1A/MYC/FBXW7/NOTCH4/NOTCH1/MAPT/PIK3R1/JAG1
REACTOME_PROGRAMMED_CELL_DEATH	20/218	3.37E-09	ITCH/STAT3/E2F1/BCL2/BCL2L11/APC/MAPT/CASP7/BMF/CTNNB1/FADD/CASP8/BAX/AKT2/TLR4/TNFRSF10B/SPTAN1/TJP1/TNFRSF10A/AKT3

WP_DYRK1A	12/218	3.97E-09	CDK6/CCND1/APP/CDK4/RBL1/RBL2/NOTCH1/MAPT/SIRT1/E2F5/FGF2/MTOR
PID_MET_PATHWAY	13/218	5.77E-09	MET/HRAS/RAF1/CDC42/APC/SNAI1/CTNNB1/AKT2/MAP2K1/EIF4E/MTOR/PIK3R1/HGF
WP_IL18_SIGNALING_PATHWAY	22/218	8.73E-09	BCL2/CCNA2/VEGFA/PTGS2/CCL4/FBXW7/PTEN/RUNX2/ULBP2/CHUK/CTNNB1/COL3A1/MAPK9/FADD/RPS6KB1/CASP8/BAX/MMP2/COL1A2/GRM7/PIK3R1/TNFSF11
REACTOME_MAPK_FAMILY_SIGNALING_CASCADES	24/218	9.27E-09	MET/IGF2BP1/NRAS/EGFR/MYC/HRAS/RAF1/CCND3/CDC42/JAK1/AGO1/IRS2/PDGFRA/PDGFRB/ARL2/FGF2/MMP2/RET/IL6R/MAP2K1/FYN/SPTAN1/PIK3R1/HGF
WP_AMPACTIVATED_PROTEIN_KINASE_SIGNALING	12/218	9.61E-09	CDKN1A/CCNA2/HNF4A/CAB39/FASN/SREBF1/RPS6KB1/AKT2/INSR/SLC2A4/MTOR/PIK3R1
KEGG_INSULIN_SIGNALING_PATHWAY	16/218	9.85E-09	NRAS/HRAS/RAF1/IRS2/FASN/MAPK9/SREBF1/RPS6KB1/AKT2/INSR/MAP2K1/EIF4E/SLC2A4/MTOR/PIK3R1/AKT3
WP_TNFRELATED_WEAK_INDUCER_OF_APOPTOSIS_TWEAK_SIGNALING_PATHWAY	10/218	1.34E-08	RAF1/CHUK/CASP7/CTNNB1/MAPK9/FADD/CASP8/AKT2/MAPK14/TRAF5
WP_RANKLRANK_SIGNALING_PATHWAY	11/218	1.40E-08	CDC42/CHUK/MAPK9/TAB1/AKT2/MAPK14/MAP2K1/TRAF5/MTOR/PIK3R1/TNFSF11
WP_EGFEGFR_SIGNALING_PATHWAY	17/218	1.47E-08	ITCH/STAT3/E2F1/EGFR/PTEN/HRAS/RAF1/CDC42/JAK1/MAPK9/TWIST1/RPS6KB1/MAPK14/MAP2K1/MTOR/RICTOR/PIK3R1
WP_TOLLLIKE_RECEPTOR_SIGNALING_PATHWAY	14/218	1.50E-08	IFNB1/CCL4/CHUK/TAB3/MAPK9/TAB1/FADD/CASP8/AKT2/TLR4/MAPK14/MAP2K1/PIK3R1/AKT3
KEGG_ERBB_SIGNALING_PATHWAY	13/218	1.77E-08	CDKN1A/NRAS/EGFR/MYC/HRAS/RAF1/MAPK9/RPS6KB1/AKT2/MAP2K1/MTOR/PIK3R1/AKT3
KEGG_T_CELL_RECEPTOR_SIGNALING_PATHWAY	14/218	2.71E-08	CDK4/NRAS/ZAP70/HRAS/RAF1/CDC42/CHUK/MAPK9/AKT2/MAPK14/MAP2K1/FYN/PIK3R1/AKT3

WP_TRANSLATION_INHIBITORS_IN_CHRONICALLY_ACTIVATED_PDGFRA_CELLS	10/218	3.21E-08	MAPK9/RPS6KB1/PDK1/AKT2/MAPK14/MAP2K1/EIF4E/MTOR/PIK3R1/AKT3
WP_H19_ACTION_RBE2F1_SIGNALING_AND_CDKBETACATENIN_ACTIVITY	7/218	3.44E-08	CCND1/CDK4/E2F1/RB1/CTNNB1/CDK8/JAG1
KEGG_VEGF_SIGNALING_PATHWAY	12/218	3.84E-08	NRAS/VEGFA/PTGS2/HRAS/RAF1/CDC42/AKT2/MAPK14/MAP2K1/PIK3R1/AKT3/KDR
REACTOME_NEGATIVE_REGULATION_OF_THE_PI3K_AKT_NETWORK	14/218	4.74E-08	MET/ESR1/EGFR/PTEN/IRS2/PDGFRA/PDGFRB/FGF2/AKT2/INSR/FYN/PIK3R1/HGF/AKT3
WP_FACTORS_AND_PATHWAYS_AFFECTING_INSULINLIKE_GROWTH_FACTOR_IGF1AKT_SIGNALING	9/218	5.23E-08	PTEN/IGF1R/RPS6KB1/PDK1/SMAD2/EIF4E/IGF1/MTOR/RICTOR
WP_NONALCOHOLIC_FATTY_LIVER_DISEASE	16/218	5.27E-08	ITCH/BCL2L11/CDC42/CASP7/IRS2/SMAD7/MAPK9/SREBF1/RXRA/CASP8/BAX/AKT2/INSR/IL6R/PIK3R1/AKT3
WP_HIPPO_SIGNALING_REGULATION_PATHWAYS	13/218	7.24E-08	MET/EGFR/EPHA2/CDC42/PDGFRA/PDGFRB/CTNNB1/LEF1/IGF1R/INSR/SMAD2/MTOR/KDR
WP_INSULIN_SIGNALING	16/218	8.20E-08	PTEN/HRAS/RAF1/IRS2/IGF1R/MAPK9/RPS6KB1/AKT2/INSR/MAPK14/MAP2K1/EIF4E/SLC2A4/PFKL/MTOR/PIK3R1
BIOCARTA_ERK_PATHWAY	8/218	8.55E-08	STAT3/EGFR/MYC/HRAS/RAF1/PDGFRA/IGF1R/MAP2K1
WP_WNT_SIGNALING_PATHWAY	10/218	8.67E-08	CDK6/CCND1/PPARG/MYC/APC/CTNNB1/LEF1/AXIN2/MAPK9/MTOR
KEGG_TOLL_LIKE_RECEPTOR_SIGNALING_PATHWAY	13/218	1.12E-07	IFNB1/CCL4/CHUK/MAPK9/TAB1/FADD/CASP8/AKT2/TLR4/MAPK14/MAP2K1/PIK3R1/AKT3

WP_NANOPARTICLEMEDIATED_ACTIVATION_OF_RECEPTOR_SIGNALING	8/218	1.12E-07	NRAS/EGFR/HRAS/RAF1/MAPK9/MAPK14/MAP2K1/AKT3
BIOCARTA_IGF1MTOR_PATHWAY	7/218	1.28E-07	PTEN/IGF1R/RPS6KB1/EIF4E/IGF1/MTOR/PIK3R1
PID_EPHB_FWD_PATHWAY	9/218	1.28E-07	NRAS/HRAS/EFNB2/EPHB4/CDC42/EPHB2/EPHB1/MAP2K1/PIK3R1
WP_IL5_SIGNALING_PATHWAY	9/218	1.28E-07	STAT3/BCL2/MYC/RAF1/MAPT/JAK1/RPS6KB1/MAP2K1/PIK3R1
WP_PDGF_PATHWAY	9/218	1.28E-07	STAT3/HRAS/RAF1/CDC42/CHUK/JAK1/PDGFRB/MAP2K1/PIK3R1
PID_TGFBR_PATHWAY	10/218	1.43E-07	ITCH/BAMBI/TGFBR1/SMAD7/CTNNB1/TAB1/RPS6KB1/SMAD2/DAB2/YAP1
WP_INTERFERON_TYPE_I_SIGNALING_PATHWAYS	10/218	1.43E-07	STAT3/ZAP70/JAK1/IRS2/RPS6KB1/MAPK14/EIF4E/FYN/MTOR/PIK3R1
PID_ERBB1_DOWNSTREAM_PATHWAY	13/218	1.53E-07	STAT3/NRAS/EGFR/HRAS/RAF1/CDC42/MAPK9/MAP2K1/SMAD1/NCKAP1/MTOR/RICTOR/PIK3R1
REACTOME_INTRINSIC_PATHWAY_FOR_APOPTOSIS	10/218	1.70E-07	STAT3/E2F1/BCL2/BCL2L11/CASP7/BMF/CASP8/BAX/AKT2/AKT3
KEGG_NEUROTROPHIN_SIGNALING_PATHWAY	14/218	1.70E-07	NTRK3/NRAS/BCL2/HRAS/RAF1/CDC42/IRS2/MAPK9/BAX/AKT2/MAPK14/MAP2K1/PIK3R1/AKT3
PID_IL2_STAT5_PATHWAY	8/218	1.92E-07	CDK6/CCND2/BCL2/CCNA2/MYC/CCND3/JAK1/PIK3R1
WP_PI3KAKTMTOR_SIGNALING_PATHWAY_AND_THERAPEUTIC_OPPORTUNITIES	8/218	1.92E-07	NRAS/PTEN/HRAS/PDK1/RB1CC1/MTOR/RICTOR/PIK3R1
WNT_SIGNALING	12/218	2.04E-07	CCND1/CCND2/MYC/CCND3/APC/CTNNB1/LEF1/WNT7A/BTRC/FOSL1/NKD1/FZD6
KEGG_WNT_SIGNALING_PATHWAY	15/218	2.35E-07	CCND1/CCND2/MYC/CCND3/APC/CTNNB1/LEF1/AXIN2/MAPK9/WNT7A/BTRC/FOSL1/SMAD2/NKD1/FZD6

WP_NEUROGENESIS_REGULATION_IN_THE_OLFACTORY_EPITHELIUM	10/218	2.36E-07	NTRK3/APP/STAT3/RELN/MYC/SOX2/NOTCH1/MAPT/RET/JAG1
REACTOME_TRANSCRIPTIONAL_REGULATION_OF_PLURIPOTENT_STEM_CELLS	8/218	2.49E-07	STAT3/LIN28A/SOX2/NANOG/EOMES/KLF4/FGF2/SMAD2
WP_MESODERMAL_COMMITMENT_PATHWAY	15/218	2.76E-07	CCND1/HMGA2/SOX2/NANOG/HNF4A/EOMES/KLF4/SETD2/SNAI1/LEF1/AXIN2/RARG/SMAD1/SMAD2/YAP1
WP_TNFALPHA_SIGNALING_PATHWAY	12/218	2.89E-07	NRAS/HRAS/RAF1/CHUK/TAB3/CASP7/MAPK9/TAB1/FADD/CASP8/BTRC/BAX
PID_ERBB2_ERBB3_PATHWAY	9/218	2.89E-07	STAT3/NRAS/HRAS/RAF1/CDC42/MAPK9/MAP2K1/MTOR/PIK3R1
BIOCARTA_MET_PATHWAY	8/218	4.07E-07	STAT3/MET/PTEN/HRAS/RAF1/MAP2K1/PIK3R1/HGF
REACTOME_REGULATION_OF_PTEN_GENE_TRANSCRIPTION	10/218	4.40E-07	PPARG/NR2E1/BMI1/PTEN/SNAI1/SUZ12/SNAI2/EZH2/CBX4/MTOR
WP_SPINAL_CORD_INJURY	13/218	5.10E-07	CCND1/CDK4/E2F1/EGFR/MYC/PTGS2/RB1/EFNB2/CDC42/E2F5/TLR4/RHOC/LGALS3
PID_IL2_PI3K_PATHWAY	8/218	5.13E-07	E2F1/BCL2/MYC/JAK1/MYB/RPS6KB1/MTOR/PIK3R1
PID_BETA_CATENIN_NUC_PATHWAY	11/218	5.95E-07	CCND1/IGF2BP1/CCND2/MYC/APC/KLF4/CTNNB1/LEF1/AXIN2/SNAI2/MMP2
PID_PI3KCI_AKT_PATHWAY	8/218	6.45E-07	CDKN1A/RAF1/CHUK/AKT2/SLC2A4/MTOR/RICTOR/AKT3
BIOCARTA_CTCF_PATHWAY	7/218	6.85E-07	MYC/TGFBR1/PTEN/RPS6KB1/SMAD1/MTOR/PIK3R1
BIOCARTA_EIF4_PATHWAY	7/218	6.85E-07	PTEN/RPS6KB1/PABPC1/MAPK14/EIF4E/MTOR/PIK3R1
WP_ANGIOGENESIS	7/218	6.85E-07	HIF1A/VEGFA/PDGFRA/FGF2/MAPK14/SMAD1/KDR

WP_NEURAL_CREST_DIFFERENTIATION	12/218	7.54E-07	MYC/NOTCH4/NOTCH1/SNAI1/CTNNB1/AXIN2/SNAI2/TWIST1/MYB/FGF2/ISL1/SMAD1
REACTOME_SMAD2_SMAD3_SMAD4_HETEROTRIMER_REGULATES_TRANSCRIPTION	8/218	7.88E-07	MYC/RBL1/E2F5/SMAD7/SMAD2/COL1A2/CDK8/TGIF2
REACTOME_FOXO_MEDIATED_TRANSCRIPTION	10/218	8.90E-07	CDKN1A/KAT2B/BCL2L11/RBL2/SIRT1/KLF4/SREBF1/AKT2/SMAD2/AKT3
WP_IL7_SIGNALING_PATHWAY	7/218	9.05E-07	CCND1/STAT3/MYC/JAK1/MAP2K1/FYN/PIK3R1
WP_RELATIONSHIP_BETWEEN_INFLAMMATION_COX2_AND_EGFR	7/218	9.05E-07	NRAS/ESR1/EGFR/PTGS2/HRAS/AKT2/AKT3
BIOCARTA_IL2RB_PATHWAY	8/218	9.71E-07	E2F1/BCL2/MYC/HRAS/RAF1/JAK1/RPS6KB1/PIK3R1
BIOCARTA_NFAT_PATHWAY	9/218	9.78E-07	HRAS/RAF1/GATA4/RPS6KB1/FGF2/MAPK14/MAP2K1/IGF1/PIK3R1
REACTOME_TRANSCRIPTIONAL_ACTIVITY_OF_SMAD2_SMAD3_SMAD4_HETEROTRIMER	9/218	9.78E-07	MYC/RBL1/E2F5/SMAD7/USP9X/SMAD2/COL1A2/CDK8/TGIF2
SIG_INSULIN_RECEPTOR_PATHWAY_IN_CARDIAC_MYOCYTES	9/218	9.78E-07	PTEN/RAF1/CDC42/IRS2/RPS6KB1/AKT2/SLC2A4/PIK3R1/AKT3
REACTOME_OXIDATIVE_STRESS_INDUCED_SENESCENCE	13/218	1.11E-06	E2F3/CDK6/CDK4/E2F1/IFNB1/BMI1/AGO1/SUZ12/MAPK9/EZH2/MAPK14/CBX4/E2F2
PID_RXR_VDR_PATHWAY	7/218	1.16E-06	BCL2/PPARG/ABCA1/SREBF1/RXRA/RARG/RPS6KB1
KEGG_APOPTOSIS	11/218	1.28E-06	BCL2/CHUK/CASP7/FADD/CASP8/BAX/AKT2/TNFRSF10B/PIK3R1/TNFRSF10A/AKT3
WP_TH17_CELL_DIFFERENTIATION_PATHWAY	10/218	1.28E-06	STAT3/HIF1A/TGFBR1/ZAP70/JAK1/RXRA/MAPK14/IL6R/SMAD2/MTOR

PID_AP1_PATHWAY	10/218	1.46E-06	CCND1/HIF1A/ESR1/MYC/BCL2L11/PTEN/CTNNB1/MYB/FOSL1/COL1A2
PID_CDC42_PATHWAY	10/218	1.46E-06	HRAS/RAF1/CDC42/APC/CTNNB1/MAPK9/RPS6KB1/MAPK14/MTOR/PIK3R1
BIOCARTA_EGF_PATHWAY	7/218	1.50E-06	STAT3/EGFR/HRAS/RAF1/JAK1/MAP2K1/PIK3R1
SIG_IL4RECEPTOR_IN_B_LYPHOCYTES	7/218	1.50E-06	BCL2/RAF1/JAK1/IRS2/AKT2/PIK3R1/AKT3
WP_NEURAL_CREST_CELL_MIGRATION_DURING_DEVELOPMENT	8/218	1.71E-06	STAT3/EPHB4/TWIST1/AKT2/MMP2/EPHB2/EPHB1/AKT3
WP_TCELL_RECEPTOR_SIGNALING_PATHWAY	11/218	1.76E-06	ZAP70/HRAS/RAF1/CDC42/CHUK/MAPK9/MAPK14/ITPR1/MAP2K1/FYN/PIK3R1
BIOCARTA_PDGF_PATHWAY	7/218	1.93E-06	STAT3/HRAS/RAF1/JAK1/PDGFRA/MAP2K1/PIK3R1
PID_ECADHERIN_STABILIZATION_PATHWAY	8/218	2.05E-06	MET/EGFR/EPHA2/CTNNB1/IGF1R/GIT1/NCKAP1/HGF
WP_SREBF_AND_MIR33_IN_CHOLESTEROL_AND_LIPID_HOMEOSTASIS	6/218	2.16E-06	ABCA1/SIRT1/FASN/SREBF1/SREBF2/MTOR
WP_APOPTOSIS_MODULATION_AND_SIGNALING	11/218	2.16E-06	BCL2/BCL2L11/BCL2L2/CASP7/BMF/BIRC5/FADD/CASP8/BAX/TNFRSF10B/TNFRSF10A
PID_AVB3_INTEGRIN_PATHWAY	10/218	2.38E-06	VEGFA/CSF1/IGF1R/COL3A1/RPS6KB1/CASP8/FGF2/COL1A2/PIK3R1/KDR
REACTOME_SIGNALING_BY_TGF_BETA_RECEPTOR_COMPLEX	11/218	2.39E-06	BAMBI/MYC/TGFBR1/RBL1/E2F5/SMAD7/USP9X/SMAD2/COL1A2/CDK8/TGIF2
WP_THYROID_HORMONES_PRODUCTION_AND_PERIPHERAL_DOWNSTREAM_SIGNALING_EFFECTS	11/218	2.39E-06	PPARG/HRAS/RAF1/NOTCH1/CTNNB1/RXRA/RPS6KB1/MAPK14/MAP2K1/MTOR/AKT3

WP_PDGFRBETA_PATHWAY	7/218	2.43E-06	STAT3/HRAS/RAF1/JAK1/PDGFRB/MAP2K1/PIK3R1
WP_WNT_SIGNALING	12/218	2.48E-06	CCND1/CCND2/MYC/CCND3/APC/CTNNB1/LEF1/MAPK9/WNT7A/FOSL1/NKD1/FZD6
KEGG_MAPK_SIGNALING_PATHWAY	18/218	2.74E-06	NRAS/EGFR/MYC/TGFBR1/HRAS/RAF1/CDC42/CHUK/MAPT/PDGFRA/PDGFRB/MAPK9/TAB1/FGF2/AKT2/MAPK14/MAP2K1/AKT3
PID_CXCR3_PATHWAY	8/218	2.90E-06	NRAS/HRAS/RAF1/MAPK14/MAP2K1/MTOR/RICTOR/PIK3R1
BIOCARTA_TGFB_PATHWAY	6/218	2.95E-06	TGFBR1/APC/SMAD7/TAB1/MAP2K1/SMAD2
REACTOME_EPHRIN_SIGNALING	6/218	2.95E-06	EFNB2/EPHB4/EPHB2/EPHB1/GIT1/FYN
REACTOME_TRANSCRIPTION_OF_E2F_TARGETS_UNDER_NEGATIVE_CONTROL_BY_DREAM_COMPLEX	6/218	2.95E-06	CDC25A/E2F1/MYC/RBL1/RBL2/E2F5
REACTOME_PTEN_REGULATION	13/218	3.08E-06	PPARG/NR2E1/BMI1/PTEN/AGO1/SNAI1/SUZ12/SNAI2/EZH2/AKT2/CBX4/MTOR/AKT3
PID_NOTCH_PATHWAY	9/218	3.10E-06	CCND1/ITCH/CDKN1A/MYC/FBXW7/NOTCH4/NOTCH1/YY1/JAG1
WP_NEUROINFLAMMATION_AND_GLUTAMATERGIC_SIGNALING	13/218	3.30E-06	STAT3/BCL2/TGFBR1/SLC2A3/JAK1/SMAD7/FGF2/INSR/IL6R/TRAF5/SMAD2/IGF1/GRM7
WP_B_CELL_RECEPTOR_SIGNALING_PATHWAY	11/218	3.48E-06	E2F3/MYC/HRAS/RAF1/CDC42/CHUK/MAPK9/MAPK14/MAP2K1/FYN/PIK3R1
WP_REGULATORY_CIRCUITS_OF_THE_STAT3_SIGNALING_PATHWAY	10/218	3.64E-06	STAT3/EGFR/JAK1/PDGFRA/PDGFRB/MAPK9/MAPK14/IL6R/MTOR/RICTOR
REACTOME_SIGNALING_BY_PDGFR_IN_DISEASE	6/218	3.99E-06	STAT3/NRAS/HRAS/PDGFRA/PIK3R1/KDR

WP_SEROTONIN_RECEPTOR_2_AND_ELKSRFGATA4_SIGNALING	6/218	3.99E-06	NRAS/HRAS/RAF1/GATA4/ITPR1/MAP2K1
KEGG_FC_EPSILON_RI_SIGNALING_PATHWAY	10/218	4.05E-06	NRAS/HRAS/RAF1/MAPK9/AKT2/MAPK14/MAP2K1/FYN/PIK3R1/AKT3
WP_MAPK_SIGNALING_PATHWAY	17/218	4.09E-06	NRAS/EGFR/MYC/TGFBR1/HRAS/RAF1/CDC42/CHUK/MAPT/PDGFRB/MAPK9/TAB1/FGF2/AKT2/MAPK14/MAP2K1/AKT3
WP_REGUCALCIN_IN_PROXIMAL_TUBULE_EPITHELIAL_KIDNEY_CELLS	7/218	4.62E-06	TGFBR1/RAF1/CASP8/BAX/SMAD2/MTOR/TNFSF11
REACTOME_SIGNALING_BY_TGFB_FAMILY_MEMBERS	12/218	4.77E-06	BAMBI/MYC/TGFBR1/RBL1/E2F5/SMAD7/USP9X/SMAD1/SMAD2/COL1A2/CDK8/TGIF2
PID_MYC_REPRESS_PATHWAY	9/218	5.23E-06	CCND1/CDKN1A/BCL2/MYC/RBL1/PDGFRB/BRCA1/SMAD2/COL1A2
BIOCARTA_CERAMIDE_PATHWAY	6/218	5.29E-06	BCL2/RAF1/FADD/CASP8/BAX/MAP2K1
BIOCARTA_IGF1_PATHWAY	6/218	5.29E-06	HRAS/RAF1/IGF1R/MAP2K1/IGF1/PIK3R1
BIOCARTA_IL6_PATHWAY	6/218	5.29E-06	STAT3/HRAS/RAF1/JAK1/IL6R/MAP2K1
BIOCARTA_INSULIN_PATHWAY	6/218	5.29E-06	HRAS/RAF1/INSR/MAP2K1/SLC2A4/PIK3R1
WP_GALANIN_RECEPTOR_PATHWAY	6/218	5.29E-06	CDKN1A/PPARG/VEGFA/BCL2L11/SLC2A4/YAP1
REACTOME_NR1H2_AND_NR1H3_MEDIATED_SIGNALING	8/218	5.36E-06	MYLIP/ABCA1/ARL4C/AGO1/FASN/SREBF1/ABCG1/RXRA
WP_THYMIC_STROMAL_LYMPHOPOIETIN_TSLP_SIGNALING_PATHWAY	8/218	5.36E-06	STAT3/MYC/JAK1/MAPK9/MAPK14/MAP2K1/FYN/MTOR

REACTOME_SIGNALING_BY_PDGFRA_TRANSMEMBRANE_JUXTAMEMBRANE_AND_KINASE_DOMAIN_MUTANTS	5/218	5.38E-06	STAT3/NRAS/HRAS/PDGFRA/PIK3R1
REACTOME_CD28_CO_STIMULATION	7/218	5.54E-06	CDC42/AKT2/FYN/MTOR/RICTOR/PIK3R1/AKT3
PID_IL4_2PATHWAY	9/218	5.77E-06	HMGA1/JAK1/IRS2/MYB/RPS6KB1/MAPK14/COL1A2/MTOR/PIK3R1
WP_NUCLEAR_RECEPTORS_METAPATHWAY	19/218	5.91E-06	CCND1/STAT3/CDK4/ESR1/KAT2B/EGFR/MYC/PTGS2/EPHA2/SLC2A3/IRS2/FASN/SNAI2/SREBF1/RXRA/BAX/CYP2C9/SLC2A4/HGF
PID_AJDISS_2PATHWAY	8/218	6.20E-06	MET/EGFR/HRAS/CDC42/CTNNB1/IGF1R/RET/FYN
WP_PLURIPOTENT_STEM_CELL_DIFFERENTIATION_PATHWAY	8/218	6.20E-06	VEGFA/NOTCH1/CSF1/FGF2/IL6R/IGF1/HGF/TNFSF11
WP_HEPATOCYTE_GROWTH_FACTOR_RECEPTOR_SIGNALING	7/218	6.74E-06	STAT3/MET/PTEN/HRAS/RAF1/MAP2K1/HGF
BIOCARTA_RAS_PATHWAY	6/218	6.85E-06	HRAS/RAF1/CDC42/CHUK/MAP2K1/PIK3R1
REACTOME_CD28_DEPENDENT_PI3K_AKT_SIGNALING	6/218	6.85E-06	AKT2/FYN/MTOR/RICTOR/PIK3R1/AKT3
WP_TRANSCRIPTION_FACTOR_REGULATION_IN_ADIPOGENESIS	6/218	6.85E-06	PPARG/IRS2/RXRA/TWIST1/INSR/SLC2A4
WP_IL3_SIGNALING_PATHWAY	8/218	7.11E-06	STAT3/BCL2/HRAS/RAF1/JAK1/MAP2K1/FYN/PIK3R1
WP_INTERLEUKIN1_IL1_STRUCTURAL_PATHWAY	8/218	7.11E-06	MYC/CHUK/TAB3/MAPK9/TAB1/MAPK14/MAP2K1/EIF4E
PID_PDGFRB_PATHWAY	12/218	7.99E-06	STAT3/NRAS/MYC/PTEN/HRAS/RAF1/PDGFRB/MAPK9/MAP2K1/FYN/NCKAP1/PIK3R1

KEGG_ADIPOCYTOKINE_SIGNALING_PATHWAY	9/218	8.11E-06	STAT3/CHUK/IRS2/MAPK9/RXRA/AKT2/SLC2A4/MTOR/AKT3
REACTOME_MYD88_INDEPENDENT_TLR4_CASCADE	11/218	8.92E-06	APP/CHUK/TAB3/MAPK9/TAB1/FADD/CASP8/BTRC/TLR4/MAPK14/MAP2K1
PID_VEGFR1_2_PATHWAY	9/218	1.03E-05	VEGFA/RAF1/CDC42/CTNNB1/MAPK14/MAP2K1/FYN/PIK3R1/KDR
BIOCARTA_WNT_PATHWAY	6/218	1.14E-05	CCND1/MYC/APC/CTNNB1/TAB1/BTRC
BIOCARTA_PRC2_PATHWAY	5/218	1.20E-05	BMI1/SUZ12/YY1/EZH2/CBX4
WP_CCL18_SIGNALING_PATHWAY	8/218	1.26E-05	HIF1A/MYC/PTEN/VEGFC/SNAI1/TWIST1/MMP2/COL1A2
REACTOME_APOPTOTIC_CLEAVAGE_OF_CELLULAR_PROTEINS	7/218	1.37E-05	APC/MAPT/CASP7/CTNNB1/CASP8/SPTAN1/TJP1
REACTOME_FLT3_SIGNALING	7/218	1.37E-05	NRAS/BCL2L11/HRAS/AKT2/FYN/PIK3R1/AKT3
WP_NUCLEAR_RECEPTORS	7/218	1.37E-05	PPARG/ESR1/NR2E1/HNF4A/RXRA/RARG/NR2C2
REACTOME_SIGNALING_BY_PTK6	8/218	1.42E-05	CCND1/STAT3/HIF1A/CDK4/NRAS/EGFR/HRAS/CCNE1
REACTOME_SIGNALING_BY_TYPE_1_INSULIN_LIKE_GROWTH_FACTOR_1_RECEPTOR_IGF1R	8/218	1.42E-05	NRAS/HRAS/IRS2/IGF1R/FGF2/AKT2/IGF1/PIK3R1
WP_IL4_SIGNALING_PATHWAY	8/218	1.42E-05	STAT3/CHUK/JAK1/IRS2/BIRC5/RPS6KB1/MAPK14/PIK3R1
REACTOME_EPH_EPHRIN_SIGNALING	10/218	1.42E-05	EPHA2/HRAS/EFNB2/EPHB4/CDC42/MMP2/EPHB2/EPHB1/GIT1/FYN
REACTOME_NUCLEOTIDE_BINDING_DOMAIN_LEUCINE_RICH_REPEAT_CONTAINING_RECEPT	8/218	1.85E-05	ITCH/APP/BCL2/CHUK/TAB3/TAB1/CASP8/MAPK14

OR_NLR_SIGNALING_PATHWAYS			
PID_GLYPICAN_1PATHWAY	6/218	2.26E-05	APP/VEGFA/TGFBR1/FGF2/SMAD2/FYN
REACTOME_SIGNALING_BY_ALK	6/218	2.26E-05	STAT3/HIF1A/MYC/PRDM1/MYCN/PIK3R1
WP_FGFR3_SIGNALING_IN_CHONDROCYTE_PROLIFERATION_AND_TERMINAL_DIFFERENTIATION	6/218	2.26E-05	CDKN1A/RBL1/RAF1/SNAI1/MAPK14/MAP2K1
REACTOME_CASPASE_ACTIVATION_VIA_DEATH_RECEPTORS_IN_THE_PRESENCE_OF_LIGAND	5/218	2.39E-05	FADD/CASP8/TLR4/TNFRSF10B/TNFRSF10A
REACTOME_TRANSCRIPTION_OF_E2F_TARGETS_UNDER_NEGATIVE_CONTROL_BY_P107_RBL1_AND_P130_RBL2_IN_COMPLEX_WITH_HDAC1	5/218	2.39E-05	E2F1/CCNA2/RBL1/RBL2/E2F5
WP_ID_SIGNALING_PATHWAY	5/218	2.39E-05	RB1/RBL1/RBL2/CCNE1/SREBF1
REACTOME_EPHB_MEDIATED_FORWARD_SIGNALING	7/218	2.62E-05	HRAS/EFNB2/EPHB4/CDC42/EPHB2/EPHB1/FYN
WP_MARKERS_OF_KIDNEY_CELL_LINEAGE	8/218	2.68E-05	HNF4A/NOTCH1/PDGFRB/CTNNB1/AXIN2/SMAD1/JAG1/KDR
PID_TRAIL_PATHWAY	6/218	2.77E-05	CHUK/FADD/CASP8/TNFRSF10B/PIK3R1/TNFRSF10A
WP_IL6_SIGNALING_PATHWAY	7/218	3.05E-05	STAT3/PRDM1/JAK1/RPS6KB1/IL6R/MAP2K1/PIK3R1
BIOCARTA_ARF_PATHWAY	5/218	3.22E-05	E2F1/MYC/RB1/TWIST1/PIK3R1

BIOCARTA_TEL_PATHWAY	5/218	3.22E-05	BCL2/EGFR/MYC/RB1/IGF1R
REACTOME_REGULATION_OF_RUNX1_EXPRESSION_AND_ACTIVITY	5/218	3.22E-05	CDK6/CCND1/CCND2/CCND3/AGO1
REACTOME_SIGNALING_BY_NTRK3_TRKC	5/218	3.22E-05	NTRK3/NRAS/HRAS/BAX/PIK3R1
SA_TRKA_RECEPTOR	5/218	3.22E-05	CDKN1A/HRAS/AKT2/MAP2K1/AKT3
BIOCARTA_BIOPEPTIDES_PATHWAY	6/218	3.30E-05	STAT3/HRAS/RAF1/MAPK14/MAP2K1/FYN
BIOCARTA_DEATH_PATHWAY	6/218	3.30E-05	BCL2/CHUK/CASP7/FADD/CASP8/SPTAN1
PID_IGF1_PATHWAY	6/218	3.30E-05	HRAS/RAF1/IRS2/IGF1R/RPS6KB1/PIK3R1
REACTOME_DOWNSTREAM_SIGNAL_TRANSDUCTION	6/218	3.30E-05	STAT3/NRAS/HRAS/PDGFRA/PDGFRB/PIK3R1
WP_REGULATION_OF_ACTIN_CYTOSKELETON	12/218	3.30E-05	NRAS/EGFR/RAF1/CDC42/APC/PDGFRA/PDGFRB/FGF2/MAP2K1/GIT1/NCKAP1/PIK3R1
PID_TRKR_PATHWAY	8/218	3.30E-05	CCND1/NTRK3/STAT3/NRAS/HRAS/CDC42/MAP2K1/PIK3R1
PID_CERAMIDE_PATHWAY	7/218	3.42E-05	BCL2/MYC/RAF1/FADD/CASP8/BAX/MAP2K1
BIOCARTA_MAPK_PATHWAY	9/218	3.45E-05	MYC/TGFBR1/HRAS/RAF1/CHUK/MAPK9/RPS6KB1/MAPK14/MAP2K1
KEGG_CYTOKINE_CYTOKINE_RECEPTOR_INTERACTION	16/218	3.69E-05	MET/VEGFA/EGFR/IFNB1/TGFBR1/CCL4/VEGFC/CSF1/PDGFRA/PDGFRB/TNFRSF10B/IL6R/HGF/TNFRSF10A/TNFSF11/KDR
PID_EPHRINB_REV_PATHWAY	6/218	3.98E-05	EFNB2/EPHB4/EPHB2/EPHB1/FYN/PIK3R1
REACTOME_ACTIVATION_OF_BH3_ONLY_PROTEINS	6/218	3.98E-05	E2F1/BCL2/BCL2L11/BMF/AKT2/AKT3

REACTOME_DEUBIQUITINATION	17/218	4.15E-05	HIF1A/CDC25A/CCNA2/ESR1/KAT2B/MYC/TGFBR1/PTEN/APC/SMAD7/YY1/AXIN2/TAB1/USP9X/BRCA1/SMAD1/SMAD2
REACTOME_TRANSCRIPTIONAL_REGULATION_BY_RUNX1	15/218	4.46E-05	CDK6/CCND1/ITCH/CCND2/ESR1/KAT2B/BMI1/RUNX2/CCND3/AGO1/MYB/CBX4/LGALS3/YAP1/TJP1
BIOCARTA_KERATINOCYTE_PATHWAY	7/218	4.51E-05	BCL2/EGFR/HRAS/RAF1/CHUK/MAPK14/MAP2K1
PID_A6B1_A6B4_INTEGRIN_PATHWAY	7/218	4.51E-05	MET/EGFR/HRAS/CASP7/RXRA/RPS6KB1/PIK3R1
PID_PS1_PATHWAY	7/218	4.51E-05	CCND1/MYC/NOTCH1/APC/CTNNB1/TAB1/NKD1
REACTOME_TOLL_LIKE_RECEPTOR_9_TLR9_CASCADE	10/218	4.51E-05	APP/CHUK/TAB3/MAPK9/TAB1/CASP8/BTRC/TLR4/MAPK14/MAP2K1
REACTOME_TRANSCRIPTIONAL_REGULATION_OF_WHITE_ADIPOCYTE_DIFFERENTIATION	9/218	4.52E-05	CDK4/PPARG/CCND3/KLF4/SREBF1/SREBF2/RXRA/SLC2A4/CDK8
PID_IL6_7_PATHWAY	7/218	5.16E-05	STAT3/MYC/JAK1/MAPK14/IL6R/PIK3R1/TNFSF11
WP_HEART_DEVELOPMENT	7/218	5.16E-05	VEGFA/NOTCH1/GATA4/VEGFC/CTNNB1/ISL1/SMAD1
WP_VITAMIN_D_RECEPTOR_PATHWAY	13/218	5.30E-05	CCND1/HIF1A/CDKN1A/CDC34/MYC/PRDM1/CCNE1/EPHB4/KLF4/RXRA/CYP2C9/SLC2A4/TNFSF11
KEGG_TGF_BETA_SIGNALING_PATHWAY	9/218	5.42E-05	MYC/TGFBR1/RBL1/RBL2/E2F5/SMAD7/RPS6KB1/SMAD1/SMAD2
BIOCARTA_ECM_PATHWAY	5/218	5.42E-05	HRAS/RAF1/MAP2K1/FYN/PIK3R1
REACTOME_VEGFR2_MEDIATED_CELL_PROLIFERATION	5/218	5.42E-05	NRAS/VEGFA/HRAS/ITPR1/KDR

WP_OVERVIEW_OF_NANOPARTICLE_EFFECTS	5/218	5.42E-05	BCL2/PTGS2/CCND3/BAX/AKT3
WP_PHYSICOCHEMICAL_FEATURES_AND_TOXICITYASSOCIATED_PATHWAYS	8/218	5.61E-05	CDKN1A/EGFR/MYC/RAF1/APC/CTNNB1/MAP2K1/FZD6
PID_WNT_NONCANONICAL_PATHWAY	6/218	5.62E-05	PPARG/CDC42/MAPK9/TAB1/CTHRC1/FZD6
REACTOME_SIGNALING_BY_NTRKS	11/218	5.89E-05	NTRK3/STAT3/NRAS/HRAS/IRS2/BAX/MAPK14/FOSL1/MAP2K1/FYN/PIK3R1
PID_ANGIOPOIETIN_RECEPTOR_PATHWAY	7/218	6.64E-05	CDKN1A/RPS6KB1/FGF2/MMP2/MAPK14/FYN/PIK3R1
PID_FOXO_PATHWAY	7/218	6.64E-05	KAT2B/BCL2L11/RBL2/CHUK/SIRT1/CTNNB1/MAPK9
WP_MIRNAS_INVOLVED_IN_DNA_DAMAGE_RESPONSE	8/218	6.86E-05	CDK6/CCND1/CDKN1A/CDC25A/E2F1/MYC/CCND3/CCNE1
BIOCARTA_GSK3_PATHWAY	5/218	6.87E-05	CCND1/APC/CTNNB1/LEF1/PIK3R1
REACTOME_SIGNALING_BY_KIT_IN_DISEASE	5/218	6.87E-05	STAT3/NRAS/HRAS/FYN/PIK3R1
WP_NANOMATERIAL_INDUCED_APOPTOSIS	5/218	6.87E-05	BCL2/CASP7/FADD/CASP8/BAX
KEGG_NATURAL_KILLER_CELL_MEDIATED_CYTOTOXICITY	11/218	7.06E-05	NRAS/IFNB1/ZAP70/HRAS/RAF1/ULBP2/TNFRSF10B/MAP2K1/FYN/PIK3R1/TNFRSF10A
REACTOME_SIGNALING_BY_ERBB2	7/218	7.42E-05	NRAS/EGFR/HRAS/AKT2/FYN/PIK3R1/AKT3
KEGG_GAP_JUNCTION	9/218	7.43E-05	NRAS/EGFR/HRAS/RAF1/PDGFRA/PDGFRB/ITPR1/MAP2K1/TJP1

REACTOME_TRANSCRIPTIONAL_REGULATION_OF_GRANULOPOIESIS	9/218	7.43E-05	STAT3/CDKN1A/CDK4/E2F1/MYC/LEF1/RXRA/MYB/IL6R
REACTOME_TRANSCRIPTIONAL_REGULATION_BY_E2F6	6/218	7.77E-05	E2F1/BMI1/SUZ12/EZH2/CHEK1/BRCA1
PID_CASPASE_PATHWAY	7/218	8.37E-05	APP/BCL2/CASP7/SREBF1/CASP8/BAX/SPTAN1
REACTOME_EPH_EPHRIN_MEDIATED_REPULSION_OF_CELLS	7/218	8.37E-05	EPHA2/EFNB2/EPHB4/MMP2/EPHB2/EPHB1/FYN
REACTOME_C_TYPE_LECTIN_RECEPTORS_CLRS	11/218	8.50E-05	CDC34/NRAS/HRAS/RAF1/CHUK/TAB3/TAB1/CASP8/BTRC/ITPR1/FYN
REACTOME_TOLL_LIKE_RECEPTOR_TLR1_TLR2_CASCADE	10/218	8.51E-05	APP/CHUK/TAB3/MAPK9/TAB1/CASP8/BTRC/TLR4/MAPK14/MAP2K1
PID_ECADHERIN_KERATINOCYTE_PATHWAY	5/218	8.65E-05	EGFR/CTNNB1/AKT2/FYN/PIK3R1
WP_STEROL_REGULATORY_ELEMENTBINDING_PROTEINS_SREBP_SIGNALING	8/218	9.10E-05	PPARG/SIRT1/FASN/YY1/SREBF1/SREBF2/CDK8/MTOR
PID_PTP1B_PATHWAY	7/218	9.38E-05	STAT3/EGFR/CSF1/PDGFRB/INSR/FYN/PIK3R1
REACTOME_APOPTOTIC_EXECUTION_PHASE	7/218	9.38E-05	APC/MAPT/CASP7/CTNNB1/CASP8/SPTAN1/TJP1
BIOCARTA_EGFR_SMRTE_PATHWAY	4/218	9.77E-05	EGFR/RXRA/MAPK14/MAP2K1
REACTOME_MET_ACTIVATES_RAS_SIGNALING	4/218	9.77E-05	MET/NRAS/HRAS/HGF

REACTOME_REGULATION_BY_C_FLIP	4/218	9.77E-05	FADD/CASP8/TNFRSF10B/TNFRSF10A
WP_NAD_METABOLISM_SIRTUINS_AND_AGING	4/218	9.77E-05	HIF1A/PPARG/SIRT1/NAMPT

Supplementary material 14: Frequency of gene combinations in pathways targeted by downregulated miRNAs.

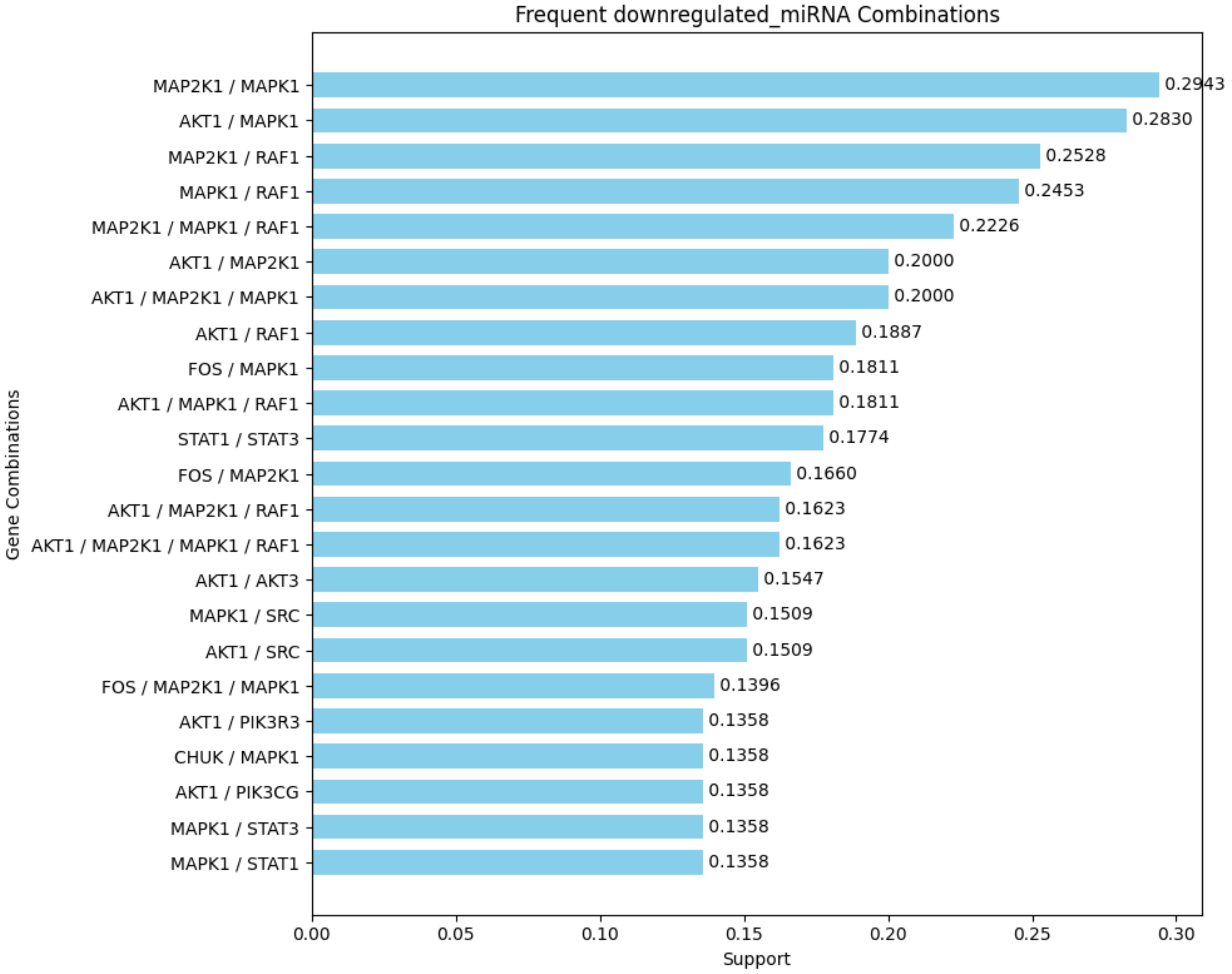

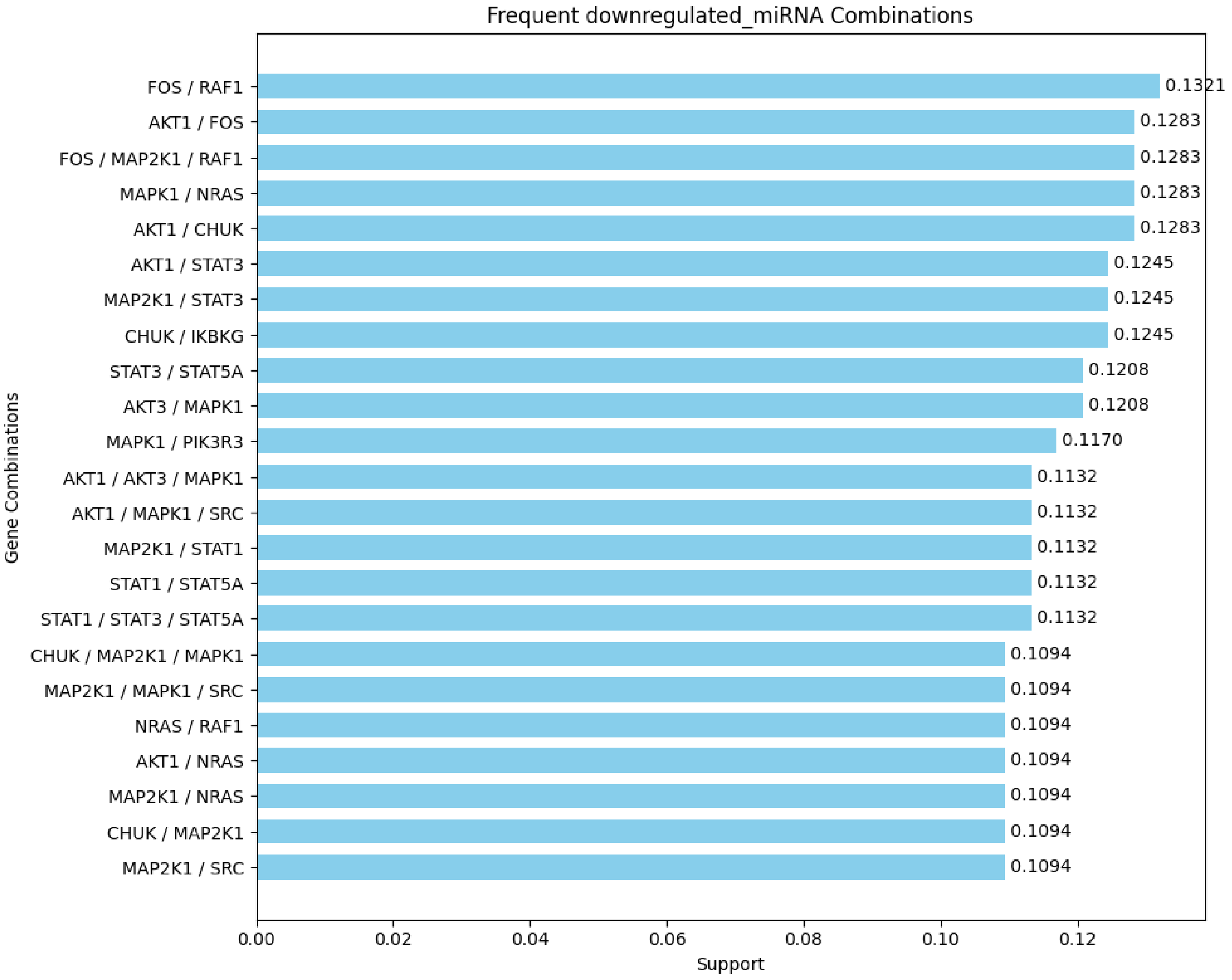
Frequent downregulated_miRNA Combinations
FOS / RAF1
AKT1 / FOS
FOS / MAP2K1 / RAF1
MAPK1 / NRAS
AKT1 / CHUK
AKT1 / STAT3
MAP2K1 / STAT3
CHUK / IKBKG
STAT3 / STAT5A
AKT3 / MAPK1
MAPK1 / PIK3R3
AKT1 / AKT3 / MAPK1
AKT1 / MAPK1 / SRC
MAP2K1 / STAT1
STAT1 / STAT5A
STAT1 / STAT3 / STAT5A
CHUK / MAP2K1 / MAPK1
MAP2K1 / MAPK1 / SRC
NRAS / RAF1
AKT1 / NRAS
MAP2K1 / NRAS
CHUK / MAP2K1
MAP2K1 / SRC
0.1321
0.1283
0.1283
0.1283
0.1283
0.1245
0.1245
0.1245
0.1208
0.1208
0.1170
0.1132
0.1132
0.1132
0.1132
0.1132
0.1094
0.1094
0.1094
0.1094
0.1094
0.1094
0.1094
Gene Combinations
0.00
0.02
0.04
0.06
0.08
0.10
0.12
Support

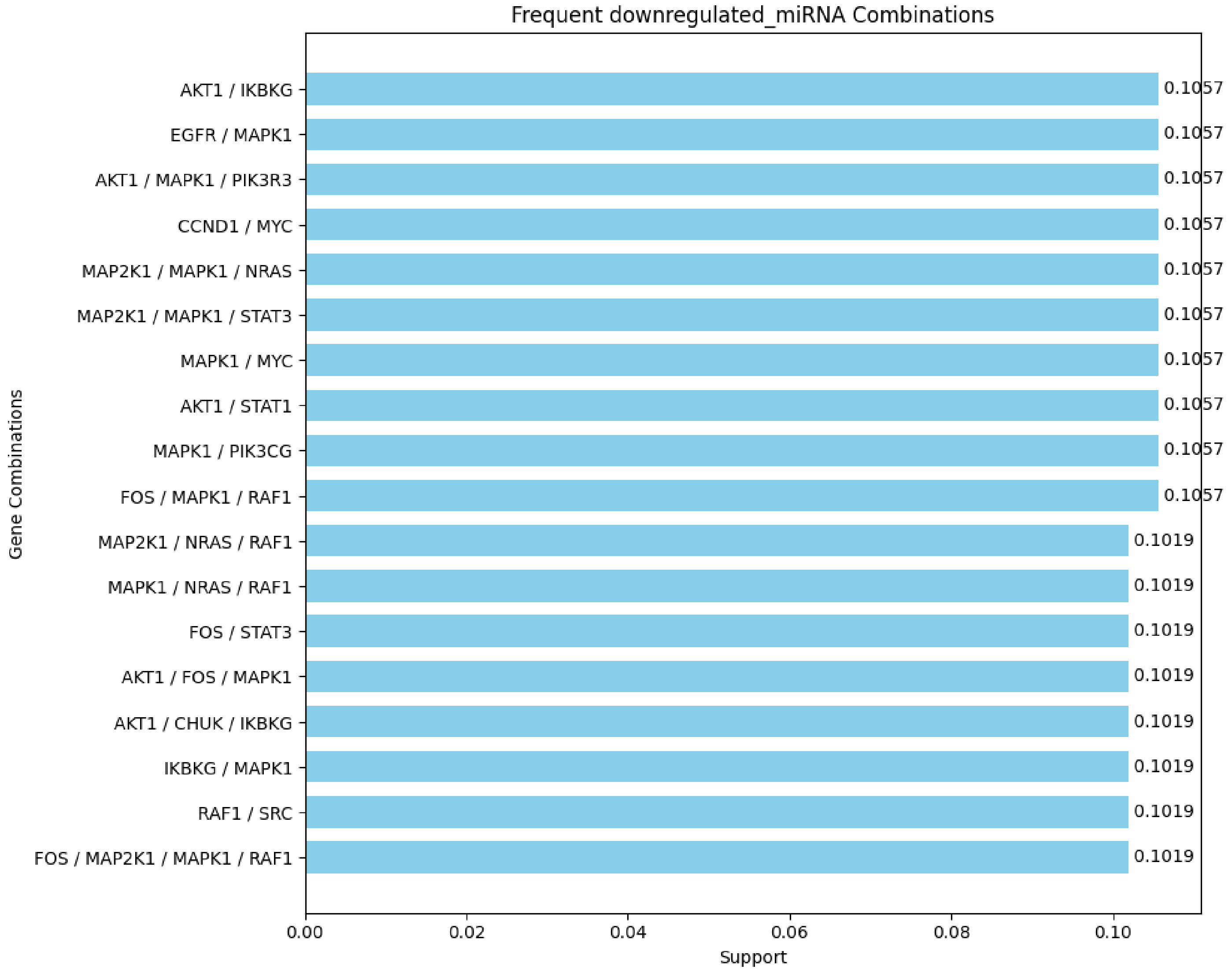
Frequent downregulated_miRNA Combinations
Gene Combinations
AKT1 / IKBKG
EGFR / MAPK1
AKT1 / MAPK1 / PIK3R3
CCND1 / MYC
MAP2K1 / MAPK1 / NRAS
MAP2K1 / MAPK1 / STAT3
MAPK1 / MYC
AKT1 / STAT1
MAPK1 / PIK3CG
FOS / MAPK1 / RAF1
MAP2K1 / NRAS / RAF1
MAPK1 / NRAS / RAF1
FOS / STAT3
AKT1 / FOS / MAPK1
AKT1 / CHUK / IKBKG
IKBKG / MAPK1
RAF1 / SRC
FOS / MAP2K1 / MAPK1 / RAF1
0.1057
0.1057
0.1057
0.1057
0.1057
0.1057
0.1057
0.1057
0.1057
0.1057
0.1019
0.1019
0.1019
0.1019
0.1019
0.1019
0.1019
0.1019
0.00
0.02
0.04
0.06
0.08
0.10
Support

Supplementary material 15: Frequency of gene combinations in pathways targeted by upregulated miRNAs.

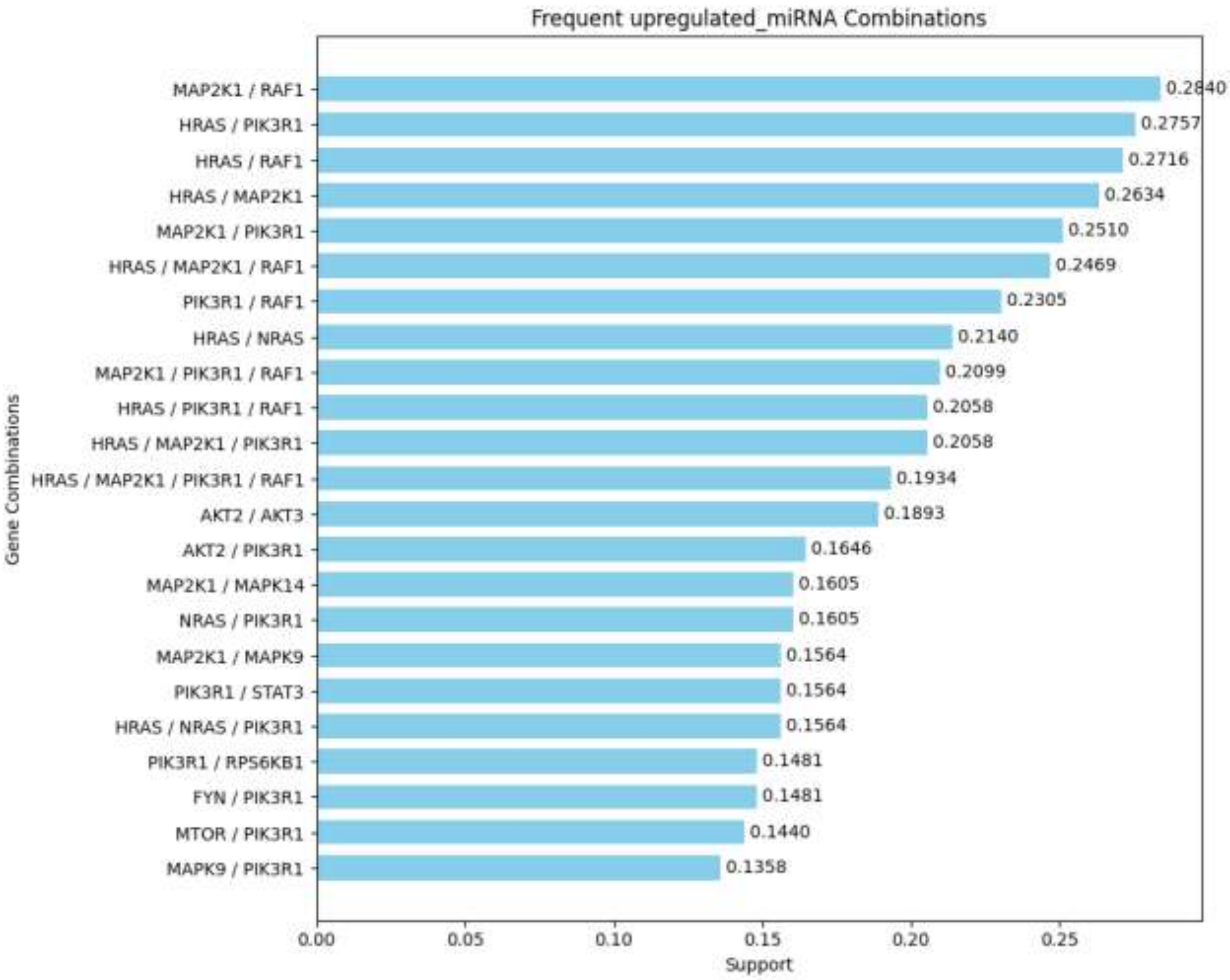

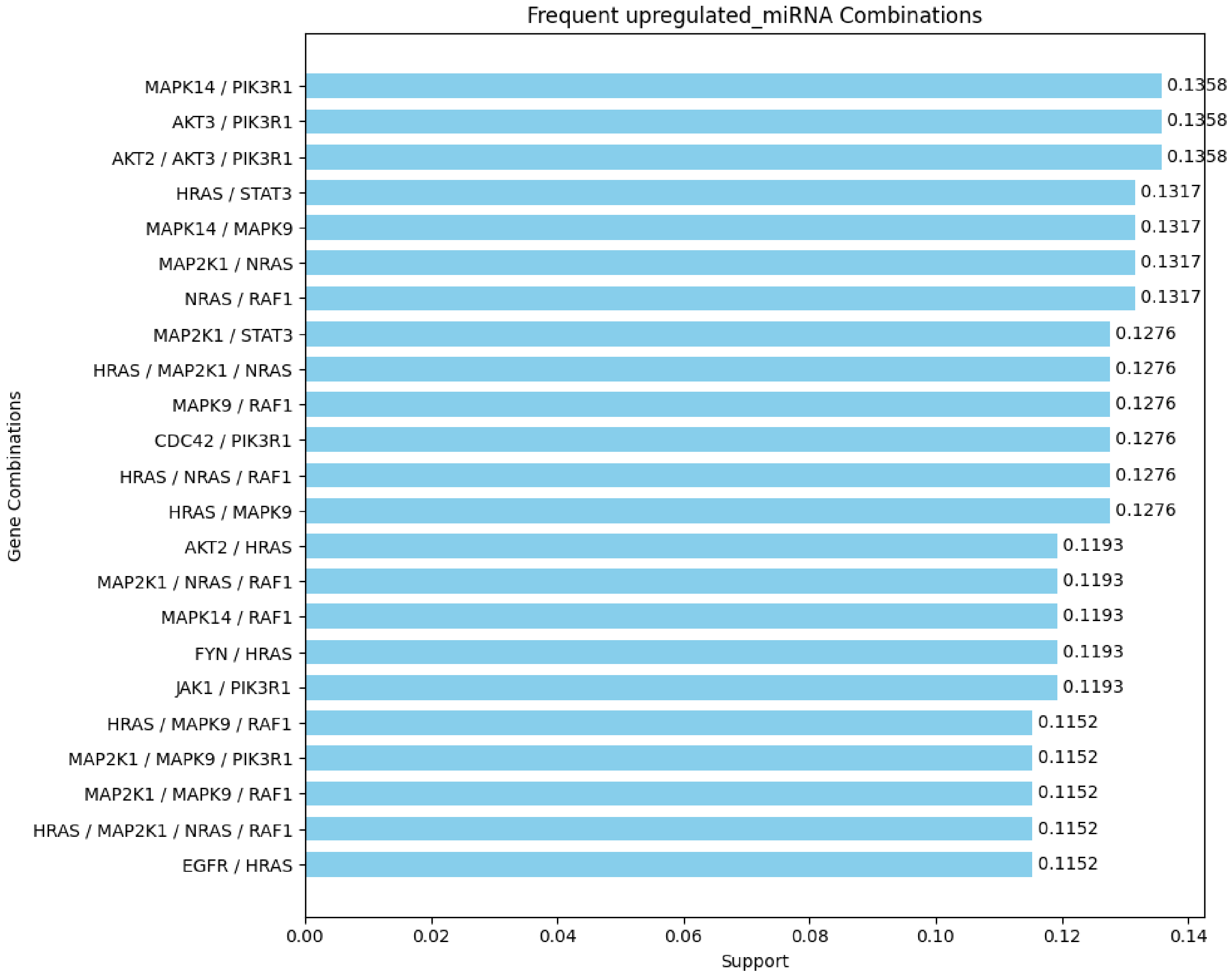
Frequent upregulated_miRNA Combinations
Gene Combinations
MAPK14 / PIK3R1
AKT3 / PIK3R1
AKT2 / AKT3 / PIK3R1
HRAS / STAT3
MAPK14 / MAPK9
MAP2K1 / NRAS
NRAS / RAF1
MAP2K1 / STAT3
HRAS / MAP2K1 / NRAS
MAPK9 / RAF1
CDC42 / PIK3R1
HRAS / NRAS / RAF1
HRAS / MAPK9
AKT2 / HRAS
MAP2K1 / NRAS / RAF1
MAPK14 / RAF1
FYN / HRAS
JAK1 / PIK3R1
HRAS / MAPK9 / RAF1
MAP2K1 / MAPK9 / PIK3R1
MAP2K1 / MAPK9 / RAF1
HRAS / MAP2K1 / NRAS / RAF1
EGFR / HRAS
0.1358
0.1358
0.1358
0.1317
0.1317
0.1317
0.1317
0.1276
0.1276
0.1276
0.1276
0.1276
0.1276
0.1193
0.1193
0.1193
0.1193
0.1193
0.1152
0.1152
0.1152
0.1152
0.1152
0.00
0.02
0.04
0.06
0.08
0.10
0.12
0.14
Support

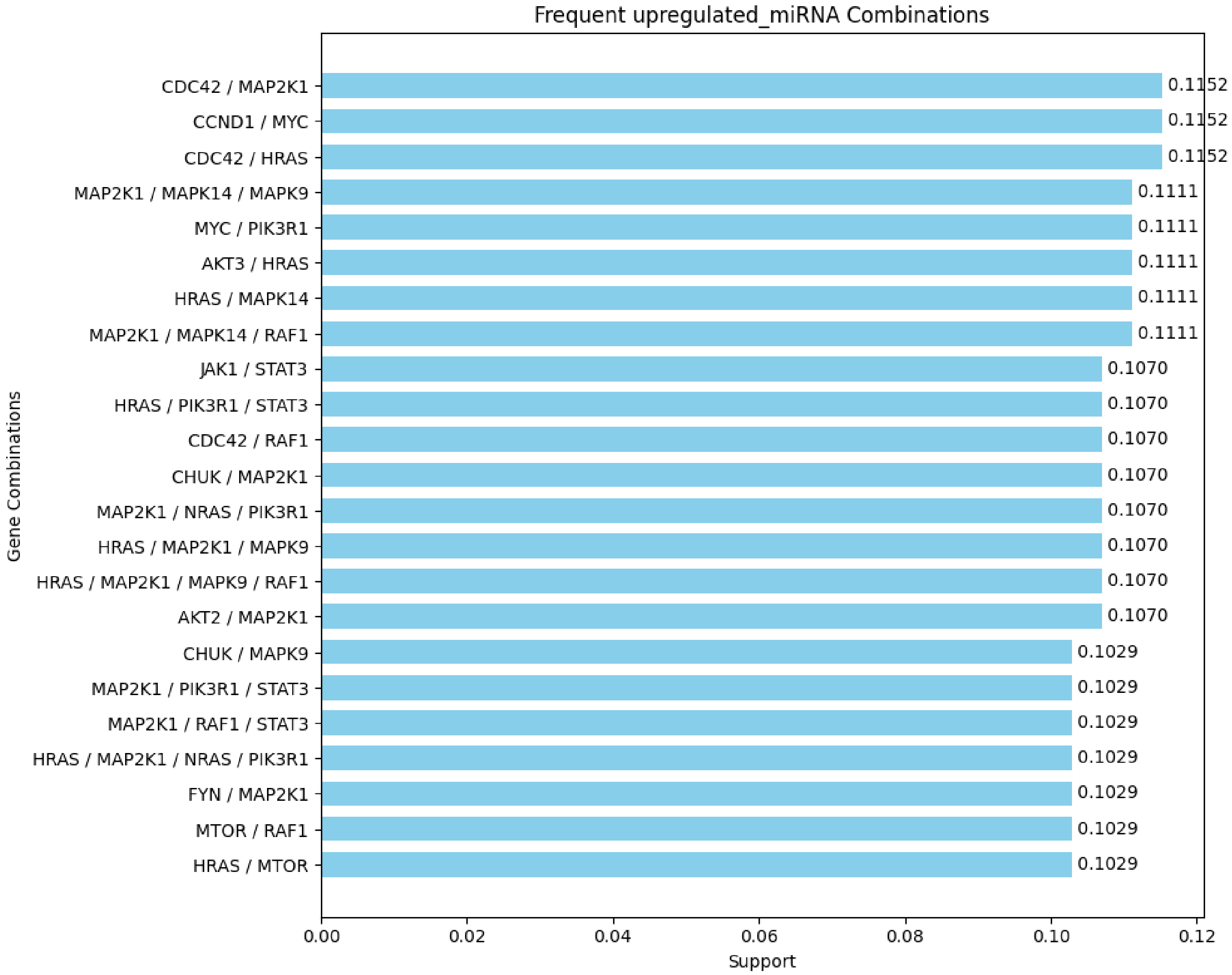
Frequent upregulated_miRNA Combinations
Gene Combinations
Support
CDC42 / MAP2K1 0.1152
CCND1 / MYC 0.1152
CDC42 / HRAS 0.1152
MAP2K1 / MAPK14 / MAPK9 0.1111
MYC / PIK3R1 0.1111
AKT3 / HRAS 0.1111
HRAS / MAPK14 0.1111
MAP2K1 / MAPK14 / RAF1 0.1111
JAK1 / STAT3 0.1070
HRAS / PIK3R1 / STAT3 0.1070
CDC42 / RAF1 0.1070
CHUK / MAP2K1 0.1070
MAP2K1 / NRAS / PIK3R1 0.1070
HRAS / MAP2K1 / MAPK9 0.1070
HRAS / MAP2K1 / MAPK9 / RAF1 0.1070
AKT2 / MAP2K1 0.1070
CHUK / MAPK9 0.1029
MAP2K1 / PIK3R1 / STAT3 0.1029
MAP2K1 / RAF1 / STAT3 0.1029
HRAS / MAP2K1 / NRAS / PIK3R1 0.1029
FYN / MAP2K1 0.1029
MTOR / RAF1 0.1029
HRAS / MTOR 0.1029
0.00 0.02 0.04 0.06 0.08 0.10 0.12

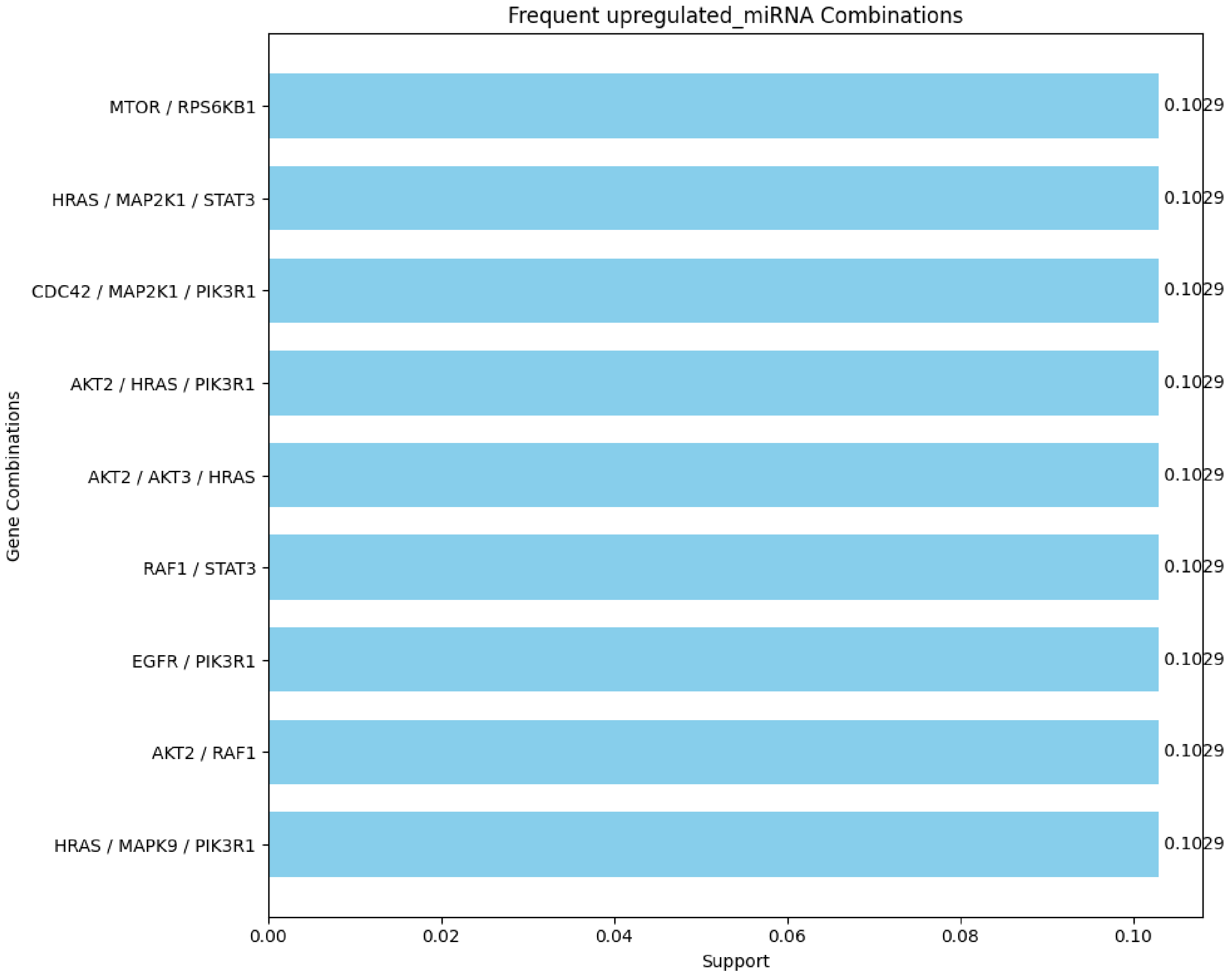

Frequent upregulated_miRNA Combinations
Gene Combinations
MTOR / RPS6KB1
HRAS / MAP2K1 / STAT3
CDC42 / MAP2K1 / PIK3R1
AKT2 / HRAS / PIK3R1
AKT2 / AKT3 / HRAS
RAF1 / STAT3
EGFR / PIK3R1
AKT2 / RAF1
HRAS / MAPK9 / PIK3R1
0.1029
0.1029
0.1029
0.1029
0.1029
0.1029
0.1029
0.1029
0.1029
0.00
0.02
0.04
0.06
0.08
0.10
Support

Supplementary material 16: Ranking of pathways targeted by down and upregulated miRNAs.

- **Ranking of pathways targeted by downregulated miRNAs:**

Pathway	Genes	Rank	Score
PID_TCR_RAS_PATHWAY	FOS/MAP2K1/MAPK1/NRAS/RAF1	1	0.3041825095
PID_AR_NONGENOMIC_PATHWAY	AKT1/AR/FOS/MAP2K1/MAPK1/RAF1/SRC	2	0.2922324823
WP_IL7_SIGNALING_PATHWAY	AKT1/CCND1/MAP2K1/MAPK1/MYC/STAT1/STAT3/STAT5A	3	0.2880228137
PID_ERBB2_ERBB3_PATHWAY	AKT1/ERBB2/FOS/MAP2K1/MAPK1/NRAS/PIK3R3/RAF1/SRC/STAT3	4	0.2703422053
WP_THYMIC_STROMAL_LYMPHOPOIETIN_TSLP_SIGNALING_PATHWAY	AKT1/CXCL8/IL6/MAP2K1/MAPK1/MYC/SRC/STAT1/STAT3/STAT5A	5	0.2680608365
WP_NANOPARTICLEMEDIATED_ACTIVATION_OF_RECEPTOR_SIGNALING	AKT3/EGFR/MAP2K1/MAPK1/NRAS/RAF1/SRC	6	0.2634437806
PID_FCER1_PATHWAY	AKT1/CBL/CHUK/FOS/IKBKG/MAP2K1/MAPK1/RAF1/RASA1	7	0.2594000845
PID_BCR_5PATHWAY	AKT1/CHUK/FOS/IKBKG/MAP2K1/MAPK1/PTEN/RAF1/RASA1	8	0.2564427545
PID_GMCSF_PATHWAY	CCL2/FOS/MAP2K1/MAPK1/NRAS/RAF1/STAT1/STAT3/STAT5A	9	0.2539079003
KEGG_B_CELL_RECEPTOR_SIGNALING_PATHWAY	AKT1/AKT3/CHUK/FOS/IKBKG/MAP2K1/MAPK1/NRAS/PIK3CG/PIK3R3/RAF1	10	0.2523332181
WP_RANKLRANK_SIGNALING_PATHWAY	AKT1/CBL/CHUK/FOS/IKBKG/MAP2K1/MAPK1/SRC/STAT1/TAB2	11	0.2498098859
WP_IL9_SIGNALING_PATHWAY	CDK9/MAP2K1/MAPK1/STAT1/STAT3/STAT5A	12	0.2496831432
PID_CXCR3_PATHWAY	AKT1/CXCL10/MAP2K1/MAPK1/NRAS/PIK3R3/RAF1/RICTOR/SRC	13	0.2450359104

BIOCARTA_KERATINOCYTE_PATHWAY	BCL2/CHUK/EGFR/FOS/MAP2K1/MAPK1/RAF1/SP1	14	0.2414448669
WP_INTERLEUKIN11_SIGNALING_PATHWAY	AKT1/BCL2/BIRC5/CHUK/MAP2K1/MAPK1/RAF1/SOCS3/SRC/STAT1/STAT3/TGFB1	15	0.2408111534
BIOCARTA_ERK_PATHWAY	EGFR/IGF1R/MAP2K1/MAPK1/MYC/PDGFRA/RAF1/STAT3	16	0.2404942966
WP_IL5_SIGNALING_PATHWAY	AKT1/BCL2/FOS/FOXO3/MAP2K1/MAPK1/MYC/PIK3CG/RAF1/SPRED1/STAT1/STAT3/STAT5A	17	0.2398362094
WP_IL3_SIGNALING_PATHWAY	AKT1/BCL2/CBL/CRKL/CXCL8/FOS/MAP2K1/MAPK1/RAF1/SRC/STAT3/STAT5A/TGFB1	18	0.2380813103
PID_MAPK_TRK_PATHWAY	EGR1/FOS/MAP2K1/MAPK1/MEF2C/NRAS/RAF1	19	0.2379141771
KEGG_VEGF_SIGNALING_PATHWAY	AKT1/AKT3/KDR/MAP2K1/MAPK1/NRAS/PIK3CG/PIK3R3/RAF1/SRC/VEGFA	20	0.2367784307
WP_RESISTIN_AS_A_REGULATOR_OF_INFLAMMATION	AKT1/AKT3/CHUK/CXCL8/IKBKG/IL6/MAPK1/PIK3CG	21	0.2343155894
WP_PDGF_PATHWAY	CHUK/FOS/MAP2K1/MAPK1/PAK1/PDGFRB/RAF1/RASA1/SRC/STAT1/STAT3	22	0.2319391635
BIOCARTA_EGF_PATHWAY	EGFR/FOS/MAP2K1/RAF1/RASA1/STAT1/STAT3/STAT5A	23	0.2267110266
WP_IL6_SIGNALING_PATHWAY	AKT1/HDAC1/IL6/IL6R/MAP2K1/MAPK1/PRDM1/SOCS3/STAT1/STAT3	24	0.2266159696
BIOCARTA_TPO_PATHWAY	FOS/MAP2K1/PIK3CG/RAF1/RASA1/STAT1/STAT3/STAT5A	25	0.2257604563
REACTOME_GROWTH_HORMONE_RECEPTOR_SIGNALING	MAPK1/SOCS1/SOCS3/STAT1/STAT3/STAT5A	26	0.2243346008
WP_IL2_SIGNALING_PATHWAY	AKT1/BCL2/CBL/CCND2/CRKL/FOS/FOXO3/MAP2K1/MAPK1/MYC/RAF1/SOCS3/STAT1/STAT3/STAT5A	27	0.2230671736
WP_REGUCALCIN_IN_PROXIMAL_TUBULE_EPITHELIAL_KIDNEY_CELLS	AKT1/BAX/CASP3/MAPK1/RAF1/SMAD2/SMAD4/TGFB1	28	0.2210076046
PID_VEGFR1_2_PATHWAY	AKT1/CBL/ITGB3/KDR/MAP2K1/MAPK1/PRKAA1/RAF1/SRC/VEGFA	29	0.2209125475

BIOCARTA_SPRY_PATHWAY	CBL/EGFR/MAP2K1/MAPK1/RAF1/RASA1/SPRY1	30	0.216186855
WP_PDGFRBETA_PATHWAY	FOS/MAP2K1/PDGFRB/RAF1/RASA1/STAT1/STAT3/STAT5A	31	0.2153041825
PID_ERBB1_RECEPTOR_PROXIMAL_PATHWAY	EGFR/MAPK1/NRAS/PAK1/PIK3R3/RASA1/SRC/STAT1/STAT3	32	0.2146176595
WP_FGFR3_SIGNALING_IN_CHONDROCYTE_PROLIFERATION_AND_TERMINAL_DIFFERENTIATION	ATG5/CDKN1A/MAP2K1/MAPK1/RAF1/SNAI1/STAT1	33	0.2134709397
BIOCARTA_NFAT_PATHWAY	AKT1/EDN1/HBEGF/IGF1/MAP2K1/MAPK1/MEF2C/PIK3CG/RAF1	34	0.2133502324
WP_NETRINUNC5B_SIGNALING_PATHWAY	AKT1/CASP3/CCL2/IL1A/KDR/MAP2K1/MAPK1/RAF1/SRC/TP53/YAP1	35	0.2125820947
WP_RELATIONSHIP_BETWEEN_INFLAMMATION_COX2_AND_EGFR	AKT1/AKT3/CYP19A1/EGFR/ESR1/MAPK1/NRAS/PIK3CG/SRC	36	0.2116603295
WP_ANGIOGENESIS	AKT1/KDR/MAPK1/MMP9/PDGFRA/SRC/VEGFA	37	0.2102118414
PID_ERBB1_DOWNSTREAM_PATHWAY	AKT1/EGFR/EGR1/FOS/MAP2K1/MAPK1/MEF2C/NRAS/PIK3R3/RAF1/RICTOR/RPS6KA3/RPS6KA4/SRC/STAT1/STAT3	38	0.2093631179
PID_IFNG_PATHWAY	AKT1/CBL/CEBPB/CRKL/IFNG/MAP2K1/MAPK1/SMAD7/SOCS1/STAT1/STAT3	39	0.2091254753
BIOCARTA_PDGF_PATHWAY	FOS/MAP2K1/PDGFRA/PIK3CG/RAF1/RASA1/STAT1/STAT3/STAT5A	40	0.2087029996
BIOCARTA_IL6_PATHWAY	CEBPB/FOS/IL6/IL6R/MAP2K1/RAF1/STAT3	41	0.2080391092
BIOCARTA_MAPK_PATHWAY	CHUK/FOS/MAP2K1/MAPK1/MYC/PAK1/RAF1/RPS6KA3/RPS6KA4/SP1/STAT1/TGFB1	42	0.2056400507
WP_HEPATOCYTE_GROWTH_FACTOR_RECEPTOR_SIGNALING	CRK/CRKL/FOS/MAP2K1/MAPK1/MET/PAK1/PTEN/RAF1/RASA1/SRC/STAT3	43	0.2056400507
BIOCARTA_IL2RB_PATHWAY	AKT1/BCL2/CBL/CRKL/E2F1/FOS/MAPK1/MYC/PIK3CG/RAF1/SOCS1/SOCS3/STAT5A	44	0.2029833285
WP_IL4_SIGNALING_PATHWAY	AKT1/BIRC5/CBL/CEBPB/CHUK/FOS/MAPK1/SOCS1/SOCS3/STAT1/STAT3/STAT5A	45	0.2027883397

WP_B_CELL_RECEPTOR_SIGNALING_PATHWAY	AKT1/CBL/CHUK/CRK/CRKL/E2F3/FOXO1/IKBKG/MAP2K1/MAPK1/MEF2C/MYC/PIK3CG/RAF1	46	0.2023356871
WP_TCELL_RECEPTOR_SIGNALING_PATHWAY	AKT1/CBL/CHUK/CRK/CRKL/FOS/IKBKG/IL1A/IL6/MAP2K1/MAPK1/PAK1/RAF1/TGFB1/ZAP70	47	0.2015209125
WP_TNFRELATED_WEAK_INDUCER_OF_APOPTOSIS_TWEAK_SIGNALING_PATHWAY	AKT1/CASP3/CCL2/CCL5/CHUK/HDAC1/IL6/MAPK1/MMP9/RAF1	48	0.2011406844
WP_TRANSLATION_INHIBITORS_IN_CHRONICALLY_ACTIVATED_PDGFRA_CELLS	AKT1/AKT3/EIF4A1/MAP2K1/MAPK1/PDCD4/PIK3CG/PIK3R3/RPS6KA4	49	0.2006759611
BIOCARTA_MET_PATHWAY	CRK/CRKL/FOS/MAP2K1/MAPK1/MET/PAK1/PIK3CG/PTEN/RAF1/RASA1/STAT3	50	0.2005703422
PID_IL2_1PATHWAY	BCL2/CDK2/FOS/IFNG/MAP2K1/MAPK1/MYC/NRAS/RAF1/RASA1/SOCS1/SOCS3/STAT1/STAT3/STAT5A	51	0.2002534854
KEGG_T_CELL_RECEPTOR_SIGNALING_PATHWAY	AKT1/AKT3/CBL/CDK4/CHUK/FOS/IFNG/IKBKG/MAP2K1/MAPK1/NRAS/PAK1/PIK3CG/PIK3R3/RAF1/ZAP70	52	0.200095057
BIOCARTA_BCELLSURVIVAL_PATHWAY	AKT1/BIRC5/CASP3/FOS/PIK3CG	53	0.2
PID_TRKR_PATHWAY	BDNF/CCND1/CRK/CRKL/MAP2K1/MAPK1/NRAS/RASA1/STAT3	54	0.199408534
PID_FGF_PATHWAY	AKT1/CBL/CTNND1/FOS/KLB/MAPK1/MET/MMP9/SRC/STAT1	55	0.1984790875
BIOCARTA_AKT_PATHWAY	AKT1/CHUK/FOXO1/FOXO3/IKBKG/PIK3CG	56	0.1977186312
REACTOME_INTRINSIC_PATHWAY_FOR_APOPTOSIS	AKT1/AKT3/BAX/BCL2/BCL2L11/CASP3/E2F1/MAPK1/STAT3/TP53	57	0.1958174905
KEGG_ERBB_SIGNALING_PATHWAY	AKT1/AKT3/CBL/CDKN1A/CRK/CRKL/EGFR/ERBB2/HBEGF/MAP2K1/MAPK1/MYC/NRAS/PAK1/PIK3CG/PIK3R3/RAF1/SRC/STAT5A	58	0.1957174305
WP_LEPTIN_SIGNALING_PATHWAY	AKT1/BAX/CCND1/CHUK/ERBB2/ESR1/FOXO1/IKBKG/MAP2K1/MAPK1/PRKAA1/PTEN/RAF1/SOCS3/SP1/SRC/STAT1/STAT3	59	0.1951837769

REACTOME_SIGNAL_TRANSDUCTION_BY_L1	EGFR/ITGB3/L1CAM/MAP2K1/MAPK1/PAK1	60	0.1939163498
WP_AGERAGE_PATHWAY	AKT1/CASP3/CHUK/EGFR/FOXO1/INHBB/INSR/IRAK4/MAP2K1/MAPK1/MMP9/RAF1/SMAD2/SP1/SRC/STAT1/STAT3/STAT5A	61	0.1934938741
PID_PI3K_PLC_TRK_PATHWAY	AKT1/CCND1/EGR1/FOXO3/NRAS/SRC/STAT5A	62	0.1928299837
WP_IL1_SIGNALING_PATHWAY	AKT1/CCL2/CHUK/IKBKG/IL1A/IRAK4/MAP2K1/MAPK1/TAB2/TAB3	63	0.1927756654
WP_INTERLEUKIN1_IL1_STRUCTURAL_PATHWAY	CHUK/FOS/IL1A/IRAK4/MAP2K1/MAPK1/MYC/TAB2/TAB3	64	0.1926489227
WP_CORTICOTROPINRELEASING_HORMONE_SIGNALING_PATHWAY	AKT1/BCL2/CASP3/CXCL8/FOS/FOSL1/MAP2K1/MAPK1/NR4A2/PARP1/RAF1/SP1/TGFB1	65	0.1915764844
WP_BRAINDERIVED_NEUROTROPHIC_FACTOR_BDNF_SIGNALING_PATHWAY	AKT1/BCL2L11/BDNF/CASP3/CHUK/EGR1/FOS/FOXO3/IKBKG/MAP2K1/MAPK1/MEF2C/PRKAA1/RAF1/RPS6KA3/SRC/STAT1/STAT3/STAT5A	66	0.1911146688
WP_NEURAL_CREST_CELL_MIGRATION_DURING_DEVELOPMENT	AKT1/AKT3/BDNF/FOS/MMP9/PAK1/PIK3CG/PIK3R3/STAT3	67	0.1909590199
WP_LEPTININSULIN_SIGNALING_OVERLAP	AKT1/INSR/PIK3CG/PIK3R3/SOCS1/SOCS3/STAT3	68	0.1901140684
SIG_INSULIN_RECEPTOR_PATHWAY_IN_CARDIAC_MYOCYTES	AKT1/AKT3/CBL/FLOT2/FOXO1/MAPK1/PTEN/RAF1/RPS6KA3/SLC2A4	69	0.1893536122
PID_MET_PATHWAY	AKT1/CBL/CRK/CRKL/EGR1/MAP2K1/MAPK1/MET/NUMB/PAK1/RAF1/SNAI1/SRC	70	0.1866042703
BIOCARTA_BAD_PATHWAY	AKT1/BAX/BCL2/IGF1/IGF1R/KIT/MAPK1/PIK3CG	71	0.1853612167
REACTOME_SIGNALING_BY_KIT_IN_DISEASE	KIT/NRAS/PIK3R3/SRC/STAT1/STAT3/STAT5A	72	0.1846822379
PID_ANGIOPOIETIN_RECEPTOR_PATHWAY	AGTR1/AKT1/CDKN1A/CRK/FOXO1/MAPK1/PAK1/RASA1/STAT5A	73	0.1846218842
WP_ERBB_SIGNALING_PATHWAY	AKT1/AKT3/BCL2L11/CBL/CCND1/CDKN1A/CRK/CRKL/EGFR/ERBB2/FOXO1/HBEGF/MAP2K1/MAPK1/MYC/NRAS/PAK1/PIK3R3/RAF1/SRC/STAT5A/TP53	74	0.1845834774

PID_IL6_7_PATHWAY	AKT1/CEBPB/FOS/FOXO1/HSP90B1/IL6/IL6R/MYC/SOCS3/STAT1/STAT3	75	0.1845834774
WP_CHEMOKINE_SIGNALING_PATHWAY	AKT1/AKT3/CCL22/CCL3/CCL5/CHUK/CRK/CRKL/CXCL10/FOXO3/IKBKG/MAP2K1/MAPK1/NRAS/PAK1/PIK3CG/PIK3R3/RAF1/STAT1/STAT3	76	0.1830798479
BIOCARTA_INSULIN_PATHWAY	FOS/INSR/MAP2K1/PIK3CG/RAF1/RASA1/SLC2A4	77	0.1819663227
PID_SYNDECAN_2_PATHWAY	BAX/CASP3/CXCL8/MAPK1/RASA1/SRC/TGFB1	78	0.1819663227
BIOCARTA_IGF1_PATHWAY	FOS/IGF1/IGF1R/MAP2K1/PIK3CG/RAF1/RASA1	79	0.1819663227
WP_TH17_CELL_DIFFERENTIATION_PATHWAY	FOS/IFNG/IL6/IL6R/MAPK1/SMAD2/SOCS3/STAT1/STAT3/STAT5A/TGFB1/ZAP70	80	0.1809252218
PID_PI3KCI_AKT_PATHWAY	AKT1/AKT3/CDKN1A/CHUK/FOXO1/FOXO3/RAF1/RICTOR/SLC2A4/SRC	81	0.1794676806
BIOCARTA_TEL_PATHWAY	AKT1/BCL2/EGFR/IGF1R/MYC/RB1/TP53	82	0.1787072243
WP_ANGIOTENSIN_II_RECEPTOR_TYPE_1_PATHWAY	AGTR1/MAPK1/RAF1/SMAD4/SP1/TGFB1/TGFBR2	83	0.1765344921
WP_TNFALPHA_SIGNALING_PATHWAY	AKT1/BAX/CASP3/CCL2/CHUK/IKBKG/IL6/MAPK1/NRAS/RAF1/TAB2/TAB3/TNFAIP3	84	0.1763673589
BIOCARTA_LONGEVITY_PATHWAY	AKT1/FOXO3/IGF1/IGF1R/PIK3CG	85	0.174904943
WP_EGFEGFR_SIGNALING_PATHWAY	AKT1/CBL/CRK/CRKL/E2F1/EGFR/ERBB2/FOS/FOXO1/MAP2K1/MAPK1/MEF2C/PAK1/PTEN/RAF1/RASA1/RICTOR/RPS6KA3/SP1/SRC/STAT1/STAT3/STAT5A/STMN1	86	0.1731622307
PID_AVB3_INTEGRIN_PATHWAY	AKT1/CBL/CSF1R/IGF1R/ITGB3/KDR/MAPK1/SRC/TGFBR2/VEGFA	87	0.1718631179
REACTOME_INTERLEUKIN_6_SIGNALING	CBL/IL6/IL6R/SOCS3/STAT1/STAT3	88	0.1717363752
WP_PI3KAKTMTOR_SIGNALING_PATHWAY_AND_THERAPEUTIC_OPPORTUNITIES	AKT1/FOXO1/FOXO3/NRAS/PIK3CG/PIK3R3/PTEN/RICTOR	89	0.1711026616
KEGG_TOLL_LIKE_RECEPTOR_SIGNALING_PATHWAY	AKT1/AKT3/CCL3/CCL5/CHUK/CXCL10/CXCL8/FOS/IFNB1/IKBKG/IL6/IRAK4/MAP2K1/MAPK1/PIK3CG/PIK3R3/STAT1/TAB2/TLR2	90	0.1703021813

KEGG_CHEMOKINE_SIGNALING_PATHWAY	AKT1/AKT3/CCL2/CCL22/CCL3/CCL5/CHUK/CRK/CRKL/CXCL10/CXCL2/CXCL8/FOXO3/IKBKG/MAP2K1/MAPK1/NRAS/PAK1/PIK3CG/PIK3R3/RAF1/STAT1/STAT3	91	0.1702760787
PID_IL23_PATHWAY	CCL2/IFNG/IL6/SOCS3/STAT1/STAT3/STAT5A	92	0.1683867463
REACTOME_SIGNALING_BY_ERBB2	AKT1/AKT3/CUL5/EGFR/ERBB2/HBEGF/NRAS/SRC	93	0.1668250951
KEGG_NEUROTROPHIN_SIGNALING_PATHWAY	AKT1/AKT3/ARHGDIB/BAX/BCL2/BDNF/CRK/CRKL/FOXO3/IRAK4/MAP2K1/MAPK1/NRAS/PIK3CG/PIK3R3/RAF1/RPS6KA3/RPS6KA4/TP53	94	0.1660996598
PID_PTP1B_PATHWAY	AKT1/CRK/CSF1R/EGFR/INSR/ITGB3/PDGFRB/SOCS3/SRC/STAT3/STAT5A	95	0.1648807466
WP_NEOVASCULARISATION_PROCESSES	AKT1/JAG1/KDR/KIT/MAPK1/MMP9/NOTCH1/SMAD2/TGFB1	96	0.1643430503
WP_IL10_ANTIINFLAMMATORY_SIGNALING_PATHWAY	IL10RA/IL1A/IL6/STAT1/STAT3	97	0.1642585551
WP_TOLLLIKE_RECEPTOR_SIGNALING_PATHWAY	AKT1/AKT3/CCL3/CCL5/CHUK/CXCL10/CXCL8/FOS/IFNB1/IKBKG/IL6/IRAK4/MAP2K1/MAPK1/PIK3CG/PIK3R3/STAT1/TAB2/TAB3/TLR2	98	0.1636882129
WP_THYROID_HORMONES_PRODUCTION_AND_PERIPHERAL_DOWNSTREAM_SIGNALING_EFFECTS	AKT3/FOXO1/ITGB3/KLB/MAP2K1/MAPK1/NOTCH1/RAF1/SIRT6/SLC16A2/SRC/TP53	99	0.1634980989
PID_MTOR_4PATHWAY	AKT1/CCNE1/CDK2/EIF4A1/MAP2K1/MAPK1/NRAS/PDCD4/RAF1/RICTOR/SREBF1/YY1	100	0.1631812421
PID_LYSOPHOSPHOLIPID_PATHWAY	AKT1/CASP3/CRK/CXCL8/EGFR/FOS/HBEGF/IL6/MMP9/PRKD1/SRC	101	0.1631524369
BIOCARTA_IL10_PATHWAY	IL10RA/IL1A/IL6/STAT1/STAT3/STAT5A	102	0.1628643853
KEGG_MTOR_SIGNALING_PATHWAY	AKT1/AKT3/IGF1/MAPK1/PIK3CG/PIK3R3/PRKAA1/RICTOR/RPS6KA3/STK11/VEGFA	103	0.162461113
WP_FIBRIN_COMPLEMENT_RECEPTOR_3_SIGNALING_PATHWAY	AKT1/CCL2/CHUK/CXCL10/IFNB1/IKBKG/IL6/IRAK4/SRC	104	0.1605407689

WP_EGFR_TYROSINE_KINASE_INHIBITOR_RESISTANCE	AKT1/AKT3/AXL/BAX/BCL2/BCL2L11/CCND1/EGFR/ERBB2/FOXO3/IGF1/IGF1R/IL6/IL6R/KDR/MAP2K1/MAPK1/MET/MYC/NRAS/PDGFRA/PDGFRB/PIK3R3/PTEN/RAF1/SRC/STAT3/VEGFA	105	0.159967409
WP_RAS_SIGNALING	AKT1/AKT3/CHUK/CSF1R/EGFR/IGF1R/IKBKG/INSR/KDR/KIT/MAP2K1/MAPK1/MET/NRAS/PAK1/PDGFRA/PDGFRB/PIK3R3/RAF1/RASA1/ZAP70	106	0.1578852073
REACTOME_SIGNALING_BY_NTRKS	BAX/BDNF/CRK/CRKL/EGR1/FOS/FOSL1/ID4/MAP2K1/MAPK1/MEF2C/NRAS/RPS6KA3/SRC/STAT3	107	0.1576679341
PID_PS1_PATHWAY	CCND1/DLL1/FOS/HDAC1/HNF1A/MAPK1/MYC/NOTCH1/WNT1	108	0.1563160118
WP_EXTRACELLULAR_VESICLEMEDIATED_SIGNALING_IN_RECIPIENT_CELLS	AKT1/EGFR/ERBB2/MET/NRAS/RAF1/SMAD2/SMAD4/TGFB1/TGFBR2/WNT3A	109	0.1562391981
BIOCARTA_CHEMICAL_PATHWAY	AKT1/ATM/BAX/BCL2/CASP3/PARP1/STAT1/TP53	110	0.155418251
REACTOME_MYD88_INDEPENDENT_TLR4_CASCADE	CHUK/FOS/HMGB1/IKBKG/MAP2K1/MAPK1/MEF2C/NLRC5/RPS6KA3/TAB2/TAB3/TP53	111	0.1552598226
REACTOME_DOWNSTREAM_SIGNAL_TRANSDUCTION	CRK/CRKL/NRAS/PDGFRA/PDGFRB/RASA1/SRC/STAT1/STAT3/STAT5A	112	0.1551330798
REACTOME_SIGNALING_BY_PDGF	CRK/CRKL/NRAS/PDGFRA/PDGFRB/RASA1/SRC/STAT1/STAT3/STAT5A	112	0.1551330798
WP_MICROTUBULE_CYTOSKELETON_REGULATION	AKT1/PAK1/PTEN/SPRED1/SRC/STAT3/STMN1/WNT3A	113	0.1544676806
WP_NONGENOMIC_ACTIONS_OF_125_DIHYDROXYVITAMIN_D3	CCL2/CXCL8/IFNG/IL6/MAPK1/NRAS/SP1/SP3/STAT1/TLR2	114	0.1539923954
PID_CXCR4_PATHWAY	AKT1/CRK/FOXO1/MMP9/PAK1/PIK3CG/PIK3R3/PTEN/RHOB/RHOC/RICTOR/SRC/STAT1/STAT3/STAT5A	115	0.1538656527
REACTOME_INTERLEUKIN_6_FAMILY_SIGNALING	CBL/IL6/IL6R/LIF/SOCS3/STAT1/STAT3	116	0.1537208039
WP_COPPER_HOMEOSTASIS	AKT1/CASP3/CCND1/FOXO1/FOXO3/PTEN/SLC31A1/SP1/TP53	117	0.1512463033

PID_SHP2_PATHWAY	BDNF/EGFR/IFNG/IGF1R/IL6/IL6R/KDR/MAP2K1/NRAS/PDGFRB/RAF1/STAT1/VEGFA	118	0.1506288388
KEGG_APOPTOSIS	AKT1/AKT3/ATM/BAX/BCL2/CASP3/CHUK/IKBKG/IL1A/IRAK4/PIK3CG/PIK3R3/TP53	119	0.1488739397
WP_TGFBETA_SIGNALING_PATHWAY	AKT1/CCND1/CDKN1A/CDKN2B/DAB2/E2F5/FOS/HDAC1/ITGB3/MAP2K1/MAPK1/MEF2C/MET/MYC/RAF1/SMAD2/SMAD4/SMAD7/SMURF1/SP1/SRC/TGFB1/TGFBR2/TP53/YAP1/ZEB1	120	0.1482889734
REACTOME_TOLL_LIKE_RECEPTOR_9_TLR9_CASCADE	CHUK/FOS/HMGB1/IKBKG/IRAK4/MAP2K1/MAPK1/MEF2C/NLRC5/RPS6KA3/TAB2/TAB3/TP53	121	0.1479964902
BIOCARTA_CTCF_PATHWAY	MYC/PIK3CG/PTEN/SMAD4/TGFB1/TGFBR2/TP53	122	0.1477457903
REACTOME_DOWNREGULATION_OF_SMAD2_3_SMAD4_TRANSCRIPTIONAL_ACTIVITY	HDAC1/MAPK1/PARP1/SMAD2/SMAD4/SMAD7/STAT1/TGIF2	123	0.147338403
WP_LTF_DANGER_SIGNAL_RESPONSE_PATHWAY	CXCL8/IFNB1/IL1A/IL6/IRAK4/MAPK1/TLR2	124	0.1472026073
REACTOME_SIGNALING_BY_SCF_KIT	CBL/CHEK1/KIT/MMP9/NRAS/PIK3R3/SOCS1/SRC/STAT1/STAT3/STAT5A	125	0.1469063256
KEGG_INSULIN_SIGNALING_PATHWAY	AKT1/AKT3/CBL/CRK/CRKL/FLOT2/FOXO1/HK2/INSR/MAP2K1/MAPK1/NRAS/PIK3CG/PIK3R3/PPP1CC/PRKAA1/RAF1/SLC2A4/SOCS1/SOCS3/SOCS4/SREBF1	126	0.1465606637
REACTOME_INACTIVATION_OF_CSF3_G_CSF_SIGNALING	CUL5/SOCS1/SOCS3/STAT1/STAT3/STAT5A	127	0.1463878327
WP_MAPK_SIGNALING_PATHWAY	AKT1/AKT3/BDNF/CACNB3/CASP3/CHUK/CRK/CRKL/EGFR/FGF7/FOS/HSPA1B/IKBKG/IL1A/MAP2K1/MAPK1/MEF2C/MYC/NRAS/PAK1/PDGFRB/RAF1/RASA1/RPS6KA3/RPS6KA4/STMN1/TAB2/TGFB1/TGFBR2/TP53	128	0.1458808619
WP_FACTORS_AND_PATHWAYS_AFFECTING_INSULINLIKE_GROWTH_FACTOR_IGF1AKT_SIGNALING	AKT1/IGF1/IGF1R/PIK3CG/PTEN/RICTOR/SMAD2	129	0.1450298751

WP_INSULIN_SIGNALING	AKT1/CBL/CRK/EGR1/FLOT2/FOS/FOXO1/FOXO3/IGF1R/INSR/MAP2K1/MAPK1/PIK3CG/PIK3R3/PRKAA1/PTEN/RAF1/RPS6KA3/RPS6KA4/SLC2A1/SLC2A4/SOCS1/SOCS3/VAMP2	130	0.1438529785
KEGG_REGULATION_OF_ACTIN_CYTOSKELETON	CRK/CRKL/EGFR/FGF7/ITGB3/ITGB8/MAP2K1/MAPK1/MYL9/NRAS/PAK1/PDGFRA/PDGFRB/PIK3CG/PIK3R3/PPP1CC/RAF1	131	0.1438157012
KEGG_MAPK_SIGNALING_PATHWAY	AKT1/AKT3/BDNF/CACNB3/CASP3/CHUK/CRK/CRKL/EGFR/FGF7/FOS/HSPA1B/IKBKG/IL1A/MAP2K1/MAPK1/MEF2C/MYC/NRAS/PAK1/PDGFRA/PDGFRB/RAF1/RASA1/RPS6KA3/RPS6KA4/STMN1/TAB2/TGFB1/TGFBR2/TP53	132	0.1435054581
REACTOME_SIGNALING_BY_PTK6	AKT1/CBL/CCND1/CCNE1/CDK2/CDK4/CRK/EGFR/ERBB2/HBEGF/NRAS/RASA1/SOCS3/STAT3	133	0.1428571429
BIOCARTA_TGFB_PATHWAY	MAP2K1/SMAD2/SMAD4/SMAD7/TGFB1/TGFBR2	134	0.1425855513
PID_TCPTP_PATHWAY	CSF1R/EGFR/INSR/KDR/MET/PDGFRB/PIK3R3/SRC/STAT1/STAT3/STAT5A/VEGFA	135	0.141634981
WP_APOPTOSIS	AKT1/BAX/BCL2/BCL2L11/BIRC5/CASP3/CHUK/IGF1/IGF1R/IKBKG/MYC/TP53	136	0.1397338403
WP_REGULATORY_CIRCUITS_OF_THE_STAT3_SIGNALING_PATHWAY	AGTR1/EGFR/IL10RA/IL6R/MAPK1/PDGFRA/PDGFRB/RICTOR/SOCS3/SRC/STAT3/STMN1	137	0.1371989861
WP_NOTCH_SIGNALING_PATHWAY	AKT1/CCND1/CDKN1A/DLL1/FBXW7/HDAC1/JAG1/MYC/NOTCH1/NOTCH2/NUMB/SRC/STAT3	138	0.1360046797
WP_ANGIOPOIETINLIKE_PROTEIN_8_REGULATORY_PATHWAY	AKT1/CBL/CRK/FLOT2/FOXO1/FOXO3/INSR/MAP2K1/MAPK1/PIK3CG/PIK3R3/PRKAA1/RAF1/RICTOR/RPS6KA3/RPS6KA4/SLC16A2/SLC2A1/SLC2A4/SREBF1/SREBF2	139	0.1359768242
REACTOME_TOLL_LIKE_RECEPTOR_TLR1_TLR2_CASCADE	CHUK/FOS/HMGB1/IKBKG/IRAK4/MAP2K1/MAPK1/MEF2C/NLRC5/RPS6KA3/SOCS1/TAB2/TAB3/TLR2/TP53	140	0.1353612167

KEGG_FOCAL_ADHESION	AKT1/AKT3/BCL2/CCND1/CCND2/CCND3/CRK/CRKL/EGFR/ERBB2/IGF1/IGF1R/ITGB3/ITGB8/KDR/MAP2K1/MAPK1/MET/MYL9/PAK1/PDGFRA/PDGFRB/PIK3CG/PIK3R3/PPP1CC/PTEN/RAF1/SRC/TLN2/VEGFA	141	0.1351077313
WP_FOCAL_ADHESION	AKT1/AKT3/BCL2/CCND1/CCND2/CCND3/CRK/CRKL/EGFR/ERBB2/IGF1/IGF1R/ITGB3/ITGB8/KDR/MAP2K1/MAPK1/MET/MYL9/PAK1/PDGFRA/PDGFRB/PIK3R3/PPP1CC/PTEN/RAF1/SRC/TLN2/VEGFA	142	0.1338665268
KEGG_TYPE_II_DIABETES_MELLITUS	HK2/INSR/MAPK1/PIK3CG/PIK3R3/SLC2A4/SOCS1/SOCS3/SOCS4	143	0.130122518
REACTOME_PTEN_REGULATION	AGO2/AKT1/AKT3/BMI1/EGR1/EZH2/HDAC1/MAPK1/MTA2/PTEN/SALL4/SNAI1/TP53	144	0.1292775665
REACTOME_TOLL_LIKE_RECEPTOR_CASCADES	CHUK/FOS/HMGB1/HSP90B1/IKBKG/IRAK4/MAP2K1/MAPK1/MEF2C/NLRC5/RPS6KA3/SOCS1/TAB2/TAB3/TLR2/TP53	145	0.1285646388
WP_TGFBETA_RECEPTOR_SIGNALING	FOS/IFNG/LEF1/LIF/SMAD2/SMAD4/SMAD7/STAT1/STAT3/TGFB1/TGFBR2/WNT1	146	0.1280101394
WP_NEUROINFLAMMATION_AND_GLUTAMATERGIC_SIGNALING	AKT1/BCL2/BDNF/FOS/GRM7/IFNG/IGF1/IL10RA/IL13/IL1A/IL6/IL6R/INSR/LIF/LRRC8A/MAPK1/PPP1CC/SLC2A1/SMAD2/SMAD4/SMAD7/SOCS3/STAT1/STAT3/TGFB1/TGFBR2	147	0.1275226674
KEGG_JAK_STAT_SIGNALING_PATHWAY	AKT1/AKT3/CBL/CCND1/CCND2/CCND3/IFNB1/IFNG/IL10RA/IL13/IL6/IL6R/LIF/MYC/PIK3CG/PIK3R3/SOCS1/SOCS3/SOCS4/SPRED1/SPRY1/STAT1/STAT3/STAT5A	148	0.1265842839
REACTOME_TRANSCRIPTIONAL_ACTIVITY_OF_SMAD2_SMAD3_SMAD4_HETEROTRIMER	CDK9/CDKN2B/E2F5/HDAC1/MAPK1/MYC/PARP1/SMAD2/SMAD4/SMAD7/SP1/STAT1/TGIF2	149	0.1243053524
WP_DNA_DAMAGE_RESPONSE_ONLY_ATM_DEPENDENT	AKT1/AKT3/ATM/BAX/BCL2/BCL2L11/CCND1/CCND2/CCND3/CDKN1A/ERBB2/FOSL1/FOXO3/HMGB1/INSR/LEF1/MAPK1/MYC/NRAS/PIK3CG/PIK3R3/PPP2R5E/PTEN/SMAD4/TCF7/TGFB1/TP53/WNT1/WNT3A	150	0.1237708142
WP_OVERVIEW_OF_NANOPARTICLE_EFFECTS	AKT3/BAX/BCL2/CCND3/CXCL8/IL6	151	0.1229404309

REACTOME_SMAD2_SMAD3_SMAD4_HETEROTRIMER_REGULATES_TRANSCRIPTION	CDK9/CDKN2B/E2F5/HDAC1/MAPK1/MYC/SMAD2/SMAD4/SMAD7/SP1/TGIF2	152	0.1216730038
PID_IL4_2PATHWAY	AKT1/CBL/CEBPB/HMGA1/ITGB3/MYB/SOCS1/SOCS3/SP1/STAT5A	153	0.1216730038
REACTOME_INTERLEUKIN_4_AND_INTERLEUKIN_13_SIGNALING	AKT1/BCL2/BIRC5/CCL2/CCL22/CCND1/CDKN1A/CXCL8/FOS/FOXO1/FOXO3/HSP90B1/IL13/IL1A/IL6/IL6R/LIF/MMP3/MMP9/MYC/NANOG/SOCS1/SOCS3/SOX2/STAT1/STAT3/TGFB1/TP53/VEGFA/ZEB1	154	0.1215462611
PID_UPA_UPAR_PATHWAY	CRK/EGFR/ITGB3/MMP3/MMP9/PDGFRB/SRC/TGFB1	155	0.1211977186
PID_FOXO_PATHWAY	AKT1/BCL2L11/CCNB1/CDK2/CHUK/FOXO1/FOXO3/KAT2B/SIRT1	156	0.1204055767
REACTOME_NEGATIVE_REGULATION_OF_THE_PI3K_AKT_NETWORK	AKT1/AKT3/EGFR/ERBB2/ESR1/FGF7/HBEGF/INSR/IRAK4/KIT/KLB/MAPK1/MET/PDGFRA/PDGFRB/PHLPP1/PHLPP2/PIK3R3/PPP2R5E/PTEN/SRC	157	0.1196813326
WP_NONALCOHOLIC_FATTY_LIVER_DISEASE	AKT1/AKT3/BAX/BCL2L11/CASP3/CCL2/CXCL8/IL1A/IL6/IL6R/INSR/PIK3R3/PPARA/PRKAA1/SMAD7/SOCS3/SREBF1/TGFB1	158	0.1193493874
REACTOME_SENESCENCE_ASSOCIATED_SECRETORY_PHENOTYPE_SASP	CCNA2/CDC27/CDK2/CDK4/CDK6/CDKN1A/CDKN2B/CDKN2C/CEBPB/CXCL8/FOS/IL1A/IL6/MAPK1/RPS6KA3/STAT3	159	0.1192965779
REACTOME_SIGNALING_BY_VEGF	AKT1/AKT3/AXL/CRK/CTNND1/CYBB/ITGB3/KDR/NRAS/PAK1/RASA1/RICTOR/SRC/VEGFA	160	0.1192286801
KEGG_ADIPOCYTOKINE_SIGNALING_PATHWAY	ACSL1/ACSL4/AKT1/AKT3/CHUK/IKBKG/PPARA/PRKAA1/SLC2A1/SLC2A4/SOCS3/STAT3/STK11	161	0.1184556888
WP_SPHINGOLIPID_METABOLISM_IN_SENESCENCE	CDK2/CDK4/CDKN1A/CERS2/E2F1/MAPK1/RB1	162	0.1184139055
WP_TLR4_SIGNALING_AND_TOLERANCE	CHUK/CXCL8/IFNB1/IKBKG/IL6/IRAK4/TAB2	163	0.1173275394
WP_SPINAL_CORD_INJURY	BDNF/CASP3/CCL2/CCND1/CDK2/CDK4/CXCL10/CXCL2/CXCL8/E2F1/E2F5/EGFR/EGR1/FOS/FOXO3/IFNG/IL1A/IL6/MAPK1/MMP9/MYC/RB1/RHOB/RHOC/TGFB1/TP53	164	0.1172857561

REACTOME_SIGNALING_BY_NUCLEAR_RECEPTORS	ABCA1/AGO2/AKT1/AKT3/BCL2/CARM1/CCND1/CDK9/EGFR/ESR1/FOS/FOXA1/FOXO3/HBEGF/HDAC1/IGF1R/KAT2B/KDM4A/MAPK1/MMP3/MMP9/MYB/MYC/MYLIP/NRAS/PIK3R3/SP1/SRC/SREBF1/YY1	165	0.1169835234
BIOCARTA_WNT_PATHWAY	CCND1/HDAC1/HNF1A/MYC/SMAD4/WNT1	166	0.1166032953
WP_VEGFAVEGFR2_SIGNALING	AKT1/BCL2/BIRC5/CBL/CCL2/CCND1/CRK/CTNND1/CXCL8/CYBB/EGR1/FOXO1/FOXO3/HBEGF/HDAC1/HMGB1/ITGB3/JAG1/KDR/LDHA/MAP2K1/MAPK1/MEF2C/NR4A2/NUMB/PAK1/PRKAA1/PRKD1/RAF1/RCAN1/RHOC/RICTOR/SRC/STAT1/STAT3/TAL1/VEGFA	167	0.1159181996
PID_P75_NTR_PATHWAY	AKT1/BCL2L11/BDNF/CASP3/CHUK/E2F1/IKBKG/MMP3/RHOB/RHOC/TP53	168	0.1157967508
REACTOME_REGULATION_OF_GENE_EXPRESSION_IN_BETA_CELLS	AKT1/AKT3/FOXO1/HNF1A/HNF4A/HNF4G/PAX6	169	0.1156979902
KEGG_ADHERENS_JUNCTION	CTNND1/EGFR/ERBB2/IGF1R/INSR/LEF1/MAPK1/MET/SMAD2/SMAD4/SNAI1/SRC/TCF7/TGFBR2	170	0.1154263987
PID_ERA_GENOMIC_PATHWAY	AXIN2/BRCA1/CCND1/CEBPB/CHUK/ESR1/HDAC1/MYC/SMAD4/STAT5A	171	0.1136882129
REACTOME_MAPK_FAMILY_SIGNALING_CASCADES	AGO2/ARTN/CCND3/EGFR/ERBB2/FGF7/FOXO1/FOXO3/GFRA3/HBEGF/IL6/IL6R/ITGB3/KIT/KLB/MAP2K1/MAPK1/MET/MYC/NRAS/PAK1/PDGFRA/PDGFRB/PPP1CC/PPP2R5E/RAF1/RASA1/RET/SPRED1/SRC	172	0.1124207858
KEGG_NOD_LIKE_RECEPTOR_SIGNALING_PATHWAY	CCL2/CCL5/CHUK/CXCL2/CXCL8/HSP90B1/IKBKG/IL6/MAPK1/NLRP3/TAB2/TAB3/TNFAIP3	173	0.1120210588
REACTOME_SIGNALING_BY_ERBB4	EGFR/ESR1/HBEGF/NRAS/SRC/STAT5A/STMN1/TAB2/YAP1	174	0.1115335868
BIOCARTA_INFLAM_PATHWAY	CXCL8/IFNB1/IFNG/IL13/IL1A/IL6/TGFB1	175	0.1108093427
REACTOME_FOXO_MEDIATED_TRANSCRIPTION	AKT1/AKT3/BCL2L11/CDKN1A/FOXO1/FOXO3/HDAC1/KAT2B/KLF4/SIRT1/SMAD2/SMAD4/SREBF1/STK11	176	0.1102661597

WP_FOCAL_ADHESION_PI3KAKTMTORSIGNALING_PATHWAY	AKT1/AKT3/CDKN1A/CSF1R/EGFR/FGF7/FOXA1/FOXO1/FOXO3/HSP90B1/IFNB1/IGF1/IGF1R/IKBKG/IL6R/INSR/ITGB3/ITGB8/KDR/KIT/MAP2K1/MAPK1/MET/NRAS/PDGFRA/PDGFRB/PHLPP1/PHLPP2/PIK3CG/PPP2R5E/PRKAA1/PTEN/RAF1/SLC2A1/SLC2A4/SREBF1/STK11/VEGFA	177	0.1101660997
BIOCARTA_ERYTH_PATHWAY	CCL3/IGF1/IL1A/IL6/TGFB1	178	0.1095057034
REACTOME_SIGNALING_BY_INTERLEUKINS	AIP/AKT1/BCL2/BIRC5/CASP3/CBL/CCL2/CCL22/CCL3/CCL5/CCND1/CDKN1A/CHUK/CRK/CRKL/CSF1R/CXCL10/CXCL2/CXCL8/FOS/FOXO1/FOXO3/HMGB1/HSP90B1/IFNG/IKBKG/IL10RA/IL13/IL1A/IL6/IL6R/IRAK4/LIF/MAP2K1/MAPK1/MEF2C/MMP3/MMP9/MYC/NANOG/NLRC5/PDCD4/PIK3R3/RPS6KA3/SOCS1/SOCS3/SOX2/STAT1/STAT3/STAT5A/STX1A/TAB2/TAB3/TGFB1/TP53/VAMP2/VEGFA/ZEB1	179	0.1090861413
WP_INTERFERON_TYPE_I_SIGNALING_PATHWAYS	CBL/CRK/CRKL/EIF4A1/PDCD4/RPS6KA4/SOCS1/SOCS3/STAT1/STAT3/STAT5A/ZAP70	180	0.1089987326
WP_PI3KAKT_SIGNALING_PATHWAY	AKT1/AKT3/BCL2/BCL2L11/BDNF/BRCA1/CCND1/CCND2/CCND3/CCNE1/CCNE2/CDK2/CDK4/CDK6/CDKN1A/CHUK/CSF1R/EGFR/FGF7/FOXO3/HSP90B1/IFNB1/IGF1/IGF1R/IKBKG/IL6/IL6R/INSR/ITGB3/ITGB8/KDR/KIT/MAP2K1/MAPK1/MET/MYB/MYC/NRAS/PDGFRA/PDGFRB/PHLPP1/PHLPP2/PIK3CG/PIK3R3/PPP2R5E/PRKAA1/PTEN/RAF1/STK11/TLR2/TP53/VEGFA	181	0.1088037438
WP_AMPACTIVATED_PROTEIN_KINASE_SIGNALING	AKT1/CCNA2/CCNB1/CDKN1A/HNF4A/INSR/PIK3CG/PIK3R3/PRKAA1/SLC2A4/SREBF1/STK11/TP53	182	0.1085112606
REACTOME_SIGNALING_BY_TGF_BETA_RECEPTOR_COMPLEX	CBL/CDK9/CDKN2B/E2F5/HDAC1/ITGB3/ITGB8/MAPK1/MYC/PARP1/PPP1CC/SMAD2/SMAD4/SMAD7/SMURF1/SP1/STAT1/TGFB1/TGFBR2/TGIF2	183	0.1079847909
PID_AP1_PATHWAY	BCL2L11/CCL2/CCND1/CXCL8/DMTF1/EDN1/EGR1/ESR1/FOS/FOSL1/IFNG/IL6/MMP9/MYB/MYC/PTEN/SP1/TGFB1/TP53	184	0.1074644787
PID_MYC_REPRESS_PATHWAY	BCL2/BRCA1/CCL5/CCND1/CDKN1A/CDKN2B/ERBB2/FOXO3/HDAC1/MYC/PDGFRB/SMAD2/SMAD4/SP1	185	0.1072786529

REACTOME_TRANSCRIPTIONAL_REGULATION_BY_THE_AP_2_TFAP2_FAMILY_OF_TRANSCRIPTION_FACTORS	CDKN1A/EGFR/ERBB2/ESR1/KIT/MYC/VEGFA/YY1	186	0.1059885932
REACTOME_SIGNALING_BY_NOTCH	AGO2/AKT1/CCND1/DLL1/E2F1/E2F3/EGFR/FBXW7/HDAC1/JAG1/KAT2B/MYC/NOTCH1/NOTCH2/NUMB/SIRT6/STAT1/TP53	187	0.1049852134
REACTOME_REGULATION_OF_PTEN_GENE_TRANSCRIPTION	BMI1/EGR1/EZH2/HDAC1/MAPK1/MTA2/PTEN/SALL4/SNAI1/TP53	188	0.1041825095
REACTOME_INTRACELLULAR_SIGNALING_BY_SECOND_MESSENGERS	AGO2/AKT1/AKT3/BMI1/CDKN1A/CHUK/EGFR/EGR1/ERBB2/ESR1/EZH2/FGF7/FOXO1/FOXO3/HBEGF/HDAC1/INSR/IRAK4/KIT/KLB/MAPK1/MET/MTA2/PDGFRA/PDGFRB/PHLPP1/PHLPP2/PIK3R3/PPP2R5E/PTEN/RICTOR/SALL4/SNAI1/SRC/TP53	189	0.10320478
BIOCARTA_CBL_PATHWAY	CBL/CSF1R/EGFR/MET/PDGFRA	190	0.102661597
WP_PROSTAGLANDIN_SIGNALING	CCL2/CCL3/CXCL10/CXCL8/IFNG/IL1A/IL6/MMP9/NLRP3/PIK3CG/TGFB1/VEGFA	191	0.1023447402
BIOCARTA_CYTOKINE_PATHWAY	CXCL8/IFNB1/IFNG/IL13/IL1A/IL6	192	0.1020278834
PID_BMP_PATHWAY	BMP7/BMPR2/CTDSP2/MAPK1/SMAD4/SMAD7/SMURF1/TAB2	193	0.1017110266
PID_NFAT_TFPATHWAY	CASP3/CDK4/CXCL8/E2F1/EGR1/FOS/FOSL1/IFNG	194	0.1012357414
WP_GALANIN_RECEPTOR_PATHWAY	BCL2L11/CDKN1A/FOS/IL6/SLC2A4/VAMP2/VEGFA/YAP1	195	0.1012357414
REACTOME_REGULATION_OF_BETA_CELL_DEVELOPMENT	AKT1/AKT3/FOXO1/HNF1A/HNF4A/HNF4G/KAT2B/NOTCH1/PAX6	196	0.1009716941
PID_HIF1_TFPATHWAY	ABCB1/AKT1/EDN1/FOS/HK2/HNF4A/LDHA/SLC2A1/SMAD4/SP1/VEGFA	197	0.1009332872
PID_IL2_STAT5_PATHWAY	BCL2/CCNA2/CCND2/CCND3/CDK6/MYC/SP1/STAT5A	198	0.1007604563
PID_SMAD2_3NUCLEAR_PATHWAY	AKT1/AR/CDK2/CDK4/CDKN1A/CDKN2B/CEBPB/E2F5/ESR1/FOS/FOXO1/FOXO3/HDAC1/HNF4A/IFNB1/KAT2B/MEF2C/MYC/SMAD2/SMAD4/SMAD7/SP1/SP3/TGIF2	199	0.1001267427

REACTOME_SIGNALING_BY_TGFB_FAMILY_MEMBERS	BMPR2/CBL/CDK9/CDKN2B/E2F5/HDAC1/INHBB/ITGB3/ITGB8/MAPK1/MYC/PARP1/PPP1CC/SMAD2/SMAD4/SMAD7/SMURF1/SP1/STAT1/TGFB1/TGFBR2/TGIF2	200	0.09989630142
WP_CYTOKINES_AND_INFLAMMATORY_RESPONSE	CXCL2/IFNB1/IFNG/IL13/IL1A/IL6/TGFB1	201	0.09885931559
REACTOME_REGULATION_OF_RUNX1_EXPRESSION_AND_ACTIVITY	AGO2/CCND1/CCND2/CCND3/CDK6/SRC	202	0.09759188847
WP_MIRNAS_INVOLVED_IN_DNA_DAMAGE_RESPONSE	ATM/CCND1/CCND3/CCNE1/CDC25A/CDK6/CDKN1A/E2F1/MYC/TP53	203	0.09619771863
KEGG_TGF_BETA_SIGNALING_PATHWAY	BMP7/BMPR2/CDKN2B/E2F5/GDF5/ID4/IFNG/INHBB/MAPK1/MYC/SMAD2/SMAD4/SMAD7/SMURF1/SP1/TGFB1/TGFBR2	204	0.09617535227
WP_TYPE_II_INTERFERON_SIGNALING	CXCL10/CYBB/IFNB1/IFNG/SOCS1/SOCS3/STAT1	205	0.09614340033
REACTOME_TRANSCRIPTIONAL_REGULATION_OF_GRANULOPOIESIS	CDK2/CDK4/CDKN1A/CEBPB/E2F1/IL6R/LEF1/MYB/MYC/STAT3/TAL1	206	0.09402004839
WP_ADIPOGENESIS	CDKN1A/CEBPB/E2F1/FOXO1/HMGA1/HNF1A/IGF1/IL6/LIF/MEF2C/NAMPT/PPARA/RB1/SLC2A4/SOCS1/SOCS3/SP1/SREBF1/STAT1/STAT3/STAT5A/TGFB1/WNT1	207	0.09389981815
WP_IL18_SIGNALING_PATHWAY	BAX/BCL2/CASP3/CCL2/CCL3/CCL5/CCNA2/CEBPB/CHUK/CXCL2/CXCL8/FBXW7/FOS/GRM7/IFNG/IL13/IL6/KCNH2/MAPK1/MMP3/MMP9/PARP1/PRKAA1/PTEN/SOCS3/SP1/STMN1/TNFAIP3/TP53/ULBP2/VEGFA	208	0.09333987489
REACTOME_NUCLEAR_SIGNALING_BY_ERBB4	ESR1/HBEGF/SRC/STAT5A/STMN1/TAB2/YAP1	209	0.09288430201
PID_HDAC_CLASSIII_PATHWAY	BAX/CDKN1A/FOXO1/FOXO3/KAT2B/SIRT1/SIRT7/TP53	210	0.08982889734
REACTOME_OXIDATIVE_STRESS_INDUCED_SENESCENCE	BMI1/CDK4/CDK6/CDKN2B/CDKN2C/E2F1/E2F2/E2F3/EZH2/FOS/IFNB1/MAPK1/MDM4/TP53	211	0.08962520369
PID_CMYB_PATHWAY	BCL2/CCNB1/CCND1/CDK6/CDKN1A/CEBPB/CSF1R/KIT/LEF1/MYB/MYC/NRAS/SP1/TAB2/WNT1	212	0.08948035488

PID_AJDISS_2PATHWAY	BDNF/CASP3/CTNND1/EGFR/IGF1R/MET/MMP3/RET/SRC	213	0.08914237431
REACTOME_INTERLEUKIN_10_SIGNALIN G	CCL2/CCL22/CCL3/CCL5/CXCL10/CXCL2/CXCL8/IL10RA/IL1A/IL6/LIF/STAT3	214	0.08871989861
PID_AR_PATHWAY	AKT1/AR/BRCA1/CARM1/CCND1/CCND3/CDK6/CTDSP2/LATS2/RPS6KA3	215	0.08859315589
WP_NUCLEAR_RECEPTORS_METAPATH WAY	ABCB1/AIP/BAX/CCL2/CCND1/CDK4/EGFR/EGR1/ESR1/FOXO1/HBEGF/IFNG/ KAT2B/LRRC8A/MYC/POU5F1/PPARA/SLC2A1/SLC2A4/SP1/SPRY1/SRC/SREB F1/STAT3/TGFB1/TGFBR2/TNFAIP3	216	0.08787494719
WP_DNA_DAMAGE_RESPONSE	AKT1/ATM/BAX/BRCA1/CASP3/CCNB1/CCND1/CCND2/CCND3/CCNE1/CCNE 2/CDC25A/CDK2/CDK4/CDK6/CDKN1A/CHEK1/E2F1/MYC/RAD51/RB1/TP53	217	0.08779813343
WP_HEMATOPOIETIC_STEM_CELL_GENE _REGULATION_BY_GABP_ALPHABETA_C OMPLEX	ATM/BCL2/DNMT1/FOXO3/PTEN/SMAD4	218	0.08681875792
PID_FRA_PATHWAY	CCL2/CCNA2/CCND1/CXCL8/DMTF1/FOSL1/IL6/LIF/MMP9/SP1	219	0.08631178707
REACTOME_TGF_BETA_RECEPTOR_SIGN ALING_ACTIVATES_SMADS	CBL/ITGB3/ITGB8/PPP1CC/SMAD2/SMAD4/SMAD7/SMURF1/TGFB1/TGFBR2	220	0.08288973384
REACTOME_TFAP2_AP_2_FAMILY_REGU LATES_TRANSCRIPTION_OF_GROWTH_F ACTORS_AND_THEIR_RECEPTORS	EGFR/ERBB2/ESR1/KIT/VEGFA/YY1	221	0.08238276299
PID_NOTCH_PATHWAY	CBL/CCND1/CDKN1A/DLL1/FBXW7/HDAC1/JAG1/MYC/NOTCH1/NOTCH2/N UMB/YY1	222	0.08206590621
WP_OVERVIEW_OF_PROINFLAMMATOR Y_AND_PROFIBROTIC_MEDIATORS	CCL2/CCL22/CCL3/CCL5/CXCL10/CXCL2/CXCL8/IFNB1/IFNG/IL13/IL1A/IL6/L IF/MMP3/MMP9/TGFB1/VEGFA	223	0.07895325431
PID_BETA_CATENIN_NUC_PATHWAY	AR/AXIN2/CCND1/CCND2/CXCL8/HDAC1/KLF4/LEF1/MMP9/MYC/SALL4/TC F7	224	0.07826362484
WP_NEUROGENESIS_REGULATION_IN_T HE_OLFACTORY_EPITHELIUM	BDNF/DLL1/JAG1/MEF2C/MYC/NOTCH1/NUMB/PAX6/RET/SOX2/STAT3	225	0.07742827515

REACTOME_REPRESSION_OF_WNT_TARGET_GENES	AXIN2/CTBP2/HDAC1/LEF1/MYC/TCF7	226	0.0773130545
WP_DYRK1A	CASP3/CCND1/CDK4/CDK6/DLL1/E2F5/FOXO1/LATS2/NOTCH1/SIRT1/TP53	227	0.07673695126
KEGG_WNT_SIGNALING_PATHWAY	AXIN2/CCND1/CCND2/CCND3/CTBP2/FOSL1/LEF1/MYC/PPP2R5E/SMAD2/SMAD4/TCF7/TP53/WNT1/WNT3A	228	0.07655259823
PID_TGFBR_PATHWAY	DAB2/SMAD2/SMAD4/SMAD7/SMURF1/TAB2/TGFB1/TGFBR2/YAP1	229	0.07477820025
WP_CCL18_SIGNALING_PATHWAY	CD44/HOTAIR/LDHA/MMP9/MTDH/MYC/PTEN/SLC2A1/SNAI1/STAT1	230	0.07452471483
REACTOME_POU5F1_OCT4_SOX2_NANOG_ACTIVATE_GENES_RELATED_TO_PROLIFERATION	NANOG/POU5F1/SALL4/SOX2/STAT3	231	0.07452471483
WP_ENERGY_METABOLISM	FOXO1/FOXO3/HDAC1/MEF2C/PPARA/PRKAA1/SIRT1/UCP2	232	0.07366920152
BIOCARTA_ATM_PATHWAY	ATM/BRCA1/CDKN1A/CHEK1/RAD51/TP53	233	0.07287705957
WP_LET7_INHIBITION_OF_ES_CELL_REPROGRAMMING	EGR1/KLF4/MYC/POU5F1/SOX2	234	0.07224334601
KEGG_CYTOKINE_CYTOKINE_RECEPTOR_INTERACTION	BMP7/BMPR2/CCL2/CCL22/CCL3/CCL5/CSF1R/CXCL10/CXCL2/CXCL8/EGFR/GDF5/IFNB1/IFNG/IL10RA/IL13/IL1A/IL6/IL6R/INHBB/KDR/KIT/LIF/MET/PDGFRA/PDGFRB/TGFB1/TGFBR2/VEGFA	235	0.0714566671
PID_HNF3A_PATHWAY	AR/BRCA1/CEBPB/ESR1/FOS/FOXA1/NFIA/SP1	236	0.07129277567
WP_MIRNA_REGULATION_OF_DNA_DAMAGE_RESPONSE	ATM/BAX/BRCA1/CASP3/CCNB1/CCND1/CCND2/CCND3/CCNE1/CCNE2/CDC25A/CDK2/CDK4/CDK6/CDKN1A/CHEK1/E2F1/MYC/RAD51/RB1/TP53	237	0.07097591888
BIOCARTA_ALK_PATHWAY	BMP7/BMPR2/HNF1A/MEF2C/SMAD4/TGFB1/TGFBR2/WNT1	238	0.06986692015
PID_HES_HEY_PATHWAY	AR/E2F1/HDAC1/KDR/MYB/NOTCH1/PARP1/RB1/RCAN1/STAT3/YY1	239	0.06947805047
REACTOME_DEUBIQUITINATION	AR/AXIN2/BECN1/BRCA1/CCNA2/CDC25A/CFTR/ESR1/IKBKG/KAT2B/MDM4/MYC/NLRP3/PTEN/SMAD2/SMAD4/SMAD7/TGFB1/TGFBR2/TNFAIP3/TP53/YY1	240	0.06913238852

REACTOME_TRANSCRIPTIONAL_REGULATION_OF_PLURIPOTENT_STEM_CELLS	FOXP1/KLF4/NANOG/POU5F1/SALL4/SMAD2/SMAD4/SOX2/STAT3	241	0.06844106464
WP_NAD_METABOLISM_SIRTUINS_AND_AGING	FOXO1/FOXO3/NAMPT/PARP1/SIRT1	242	0.06768060837
REACTOME_TRANSCRIPTIONAL_REGULATION_BY_MECP2	AGO2/BDNF/DLL1/HDAC1/MEF2C/MET/NOTCH1/PTEN/SOX2	243	0.06759611322
WP_HIPPO_SIGNALING_REGULATION_PATHWAYS	CSF1R/EGFR/IGF1R/INSR/KDR/KIT/LATS2/LEF1/MET/PDGFRA/PDGFRB/PRKAA1/SMAD2/TCF7/YY1AP1	244	0.06717363752
WP_DNA_IRDAMAGE_AND_CELLULAR_RESPONSE_VIA_ATR	ATM/BRCA1/CDK2/CHEK1/E2F1/IKBKG/PARP1/PPM1D/RAD51/SP1/TP53	245	0.06705841687
WP_IL1_AND_MEGAKARYOCYTES_IN_OBESITY	CCL2/HBEGF/IFNG/MMP9/NLRP3/TLR2	246	0.06527249683
REACTOME_TRANSCRIPTIONAL_REGULATION_BY_RUNX1	AGO2/BMI1/CCND1/CCND2/CCND3/CDK6/ESR1/HDAC1/IFNG/KAT2B/LMO2/MYB/MYL9/SOCS3/SOCS4/SRC/TAL1/YAP1	247	0.06527249683
WP_DNA_IRDOUBLE_STRAND_BREAKS_AND_CELLULAR_RESPONSE_VIA_ATM	ATM/BAX/BRCA1/CASP3/CHEK1/E2F1/PARP1/RAD51/TP53/YAP1	248	0.06425855513
NABA_SECRETED_FACTORS	ARTN/BDNF/BMP7/CCL2/CCL22/CCL3/CCL5/CXCL10/CXCL2/CXCL8/FGF7/GDF5/HBEGF/IFNB1/IFNG/IGF1/IL13/IL1A/IL6/INHBB/LIF/TGFB1/VEGFA/WNT1/WNT3A	249	0.06326996198
WP_ATM_SIGNALING_PATHWAY	ATM/BRCA1/CCNB1/CCNE1/CDC25A/CDK2/CDKN1A/CHEK1/IKBKG/MDM4/RAD51/TP53	250	0.06051964512
WP_H19_ACTION_RBE2F1_SIGNALING_AND_CDKBETACATENIN_ACTIVITY	CCND1/CDK4/E2F1/JAG1/RB1/SOX4	251	0.05956907478
PID_HDAC_CLASSI_PATHWAY	HDAC1/KAT2B/MTA2/MXD1/SIRT1/SIRT6/SIRT7/SMAD7/SMURF1/STAT3/YY1	252	0.05876253025
WP_VITAMIN_D_RECEPTOR_PATHWAY	ABCB1/CCND1/CCNE1/CDK2/CDKN1A/CDKN2B/CDKN2C/FOXO1/HNF1A/ID4/KLF4/LRRC8A/MXD1/MYC/PRDM1/SALL4/SATB1/SLC2A4/TGFB1/TNFAIP3	253	0.05855513308

WP_WNT_SIGNALING_PATHWAY_AND_PLURIPOTENCY	AXIN2/CCND1/CCND2/CCND3/CD44/CTBP2/CTNND1/FOSL1/LEF1/MYC/NANOG/POU5F1/PPP2R5E/PRKD1/SOX2/TCF7/TP53/WNT1/WNT3A	254	0.05483289974
REACTOME_DNA_DAMAGE_TELOMERE_STRESS_INDUCED_SENESCENCE	ATM/CCNA2/CCNE1/CCNE2/CDK2/CDKN1A/HMGA1/HMGA2/RB1/TP53	255	0.05285171103
REACTOME_TRANSCRIPTIONAL_REGULATION_OF_WHITE_ADIPOCYTE_DIFFERENTIATION	CARM1/CCND3/CDK4/CEBPB/KLF4/PPARA/SLC2A4/SREBF1/SREBF2/TGFB1/WNT1	256	0.05150362945
WP_NEURAL_CREST_DIFFERENTIATION	AXIN2/BMP7/CTBP2/DLL1/HDAC1/MYB/MYC/NOTCH1/NOTCH2/RHOB/SNAI1/SOX5/WNT1/WNT3A	257	0.04915806627
REACTOME_FORMATION_OF_AXIAL_MESODERM	FOXA1/LEF1/SMAD2/TCF7/YAP1	258	0.04486692015
KEGG_NOTCH_SIGNALING_PATHWAY	CTBP2/DLL1/HDAC1/JAG1/KAT2B/NOTCH1/NOTCH2/NUMB	259	0.04420152091
WP_NOTCH_SIGNALING	CTBP2/DLL1/HDAC1/JAG1/KAT2B/NOTCH1/NOTCH2/NUMB	259	0.04420152091
BIOCARTA_HES_PATHWAY	DLL1/HNF1A/NOTCH1/NOTCH2/WNT1	260	0.03878326996
WP_NANOPARTICLE_TRIGGERED_AUTOPHAGIC_CELL_DEATH	ATG16L1/ATG4A/ATG5/ATG7/BCL2/BECN1/INSR	261	0.03476371537
WP_SREBF_AND_MIR33_IN_CHOLESTEROL_AND_LIPID_HOMEOSTASIS	ABCA1/PPARA/PRKAA1/SIRT1/SIRT6/SREBF1/SREBF2	262	0.02933188485
KEGG_REGULATION_OF_AUTOPHAGY	ATG4A/ATG4B/ATG4C/ATG4D/ATG5/ATG7/BECN1/IFNG/PRKAA1	263	0.02492606675

- Ranking of pathways targeted by upregulated miRNAs:

**Pathway**	**Genes**	**Rank**	**Score**
BIOCARTA_ECM_PATHWAY	FYN/HRAS/MAP2K1/PIK3R1/RAF1	1	0.3456066946
BIOCARTA_RAS_PATHWAY	CDC42/CHUK/HRAS/MAP2K1/PIK3R1/RAF1	2	0.3117154812
BIOCARTA_EGF_PATHWAY	EGFR/HRAS/JAK1/MAP2K1/PIK3R1/RAF1/STAT3	3	0.3036461447
REACTOME_SIGNALING_BY_KIT_IN_DISEASE	FYN/HRAS/NRAS/PIK3R1/STAT3	4	0.3020920502
WP_PDGFRBETA_PATHWAY	HRAS/JAK1/MAP2K1/PDGFRB/PIK3R1/RAF1/STAT3	5	0.291691572
PID_ERBB2_ERBB3_PATHWAY	CDC42/HRAS/MAP2K1/MAPK9/MTOR/NRAS/PIK3R1/RAF1/STAT3	6	0.2905625291
BIOCARTA_BIOPEPTIDES_PATHWAY	FYN/HRAS/MAP2K1/MAPK14/RAF1/STAT3	7	0.2894002789
BIOCARTA_PDGF_PATHWAY	HRAS/JAK1/MAP2K1/PDGFRA/PIK3R1/RAF1/STAT3	8	0.2893006575
WP_IL3_SIGNALING_PATHWAY	BCL2/FYN/HRAS/JAK1/MAP2K1/PIK3R1/RAF1/STAT3	9	0.2876569038
BIOCARTA_IGF1_PATHWAY	HRAS/IGF1/IGF1R/MAP2K1/PIK3R1/RAF1	10	0.2831241283
REACTOME_SIGNALING_BY_PDGFRA_TRANSMEMBRANE_JUXTAMEMBRANE_AND_KINASE_DOMAIN_MUTANTS	HRAS/NRAS/PDGFRA/PIK3R1/STAT3	11	0.2820083682

PID_CXCR3_PATHWAY	HRAS/MAP2K1/MAPK14/MTOR/NRAS/PIK3R1/RAF1/RICTOR	12	0.2819037657
KEGG_FC_EPSILON_RI_SIGNALING_PATHWAY	AKT2/AKT3/FYN/HRAS/MAP2K1/MAPK14/MAPK9/NRAS/PIK3R1/RAF1	13	0.2815899582
BIOCARTA_INSULIN_PATHWAY	HRAS/INSR/MAP2K1/PIK3R1/RAF1/SLC2A4	14	0.280334728
REACTOME_SIGNALING_BY_ERBB2	AKT2/AKT3/EGFR/FYN/HRAS/NRAS/PIK3R1	15	0.2677824268
WP_NANOPARTICLEMEDIATED_ACTIVATION_OF_RECEPTOR_SIGNALING	AKT3/EGFR/HRAS/MAP2K1/MAPK14/MAPK9/NRAS/RAF1	16	0.2662133891
WP_IL7_SIGNALING_PATHWAY	CCND1/FYN/JAK1/MAP2K1/MYC/PIK3R1/STAT3	17	0.264196055
WP_PDGF_PATHWAY	CDC42/CHUK/HRAS/JAK1/MAP2K1/PDGFRB/PIK3R1/RAF1/STAT3	18	0.2640632264
BIOCARTA_MET_PATHWAY	HGF/HRAS/MAP2K1/MET/PIK3R1/PTEN/RAF1/STAT3	19	0.2609832636
PID_IGF1_PATHWAY	HRAS/IGF1R/IRS2/PIK3R1/RAF1/RPS6KB1	20	0.2608089261
BIOCARTA_KERATINOCYTE_PATHWAY	BCL2/CHUK/EGFR/HRAS/MAP2K1/MAPK14/RAF1	21	0.2606096832
KEGG_ERBB_SIGNALING_PATHWAY	AKT2/AKT3/CDKN1A/EGFR/HRAS/MAP2K1/MAPK9/MTOR/MYC/NRAS/PIK3R1/RAF1/RPS6KB1	22	0.2597360798
PID_IL2_1PATHWAY	BCL2/FYN/HRAS/IRS2/JAK1/MAP2K1/MAPK14/MAPK9/MYC/NRAS/PIK3R1/RAF1/STAT3	23	0.2574831027
BIOCARTA_IL6_PATHWAY	HRAS/IL6R/JAK1/MAP2K1/RAF1/STAT3	24	0.2566248257

SA_TRKA_RECEPTOR	AKT2/AKT3/CDKN1A/HRAS/MAP2K1	25	0.2560669456
PID_TRKR_PATHWAY	CCND1/CDC42/HRAS/MAP2K1/NRAS/NTRK3/PIK3R1/STAT3	26	0.2552301255
BIOCARTA_IL2RB_PATHWAY	BCL2/E2F1/HRAS/JAK1/MYC/PIK3R1/RAF1/RPS6KB1	27	0.2541841004
WP_B_CELL_RECEPTOR_SIGNALING_PATHWAY	CDC42/CHUK/E2F3/FYN/HRAS/MAP2K1/MAPK14/MAPK9/MYC/PIK3R1/RAF1	28	0.2537086345
REACTOME_DOWNSTREAM_SIGNAL_TRANSDUCTION	HRAS/NRAS/PDGFRA/PDGFRB/PIK3R1/STAT3	29	0.2531380753
PID_ECADHERIN_KERATINOCYTE_PATHWAY	AKT2/CTNNB1/EGFR/FYN/PIK3R1	30	0.2510460251
REACTOME_FLT3_SIGNALING	AKT2/AKT3/BCL2L11/FYN/HRAS/NRAS/PIK3R1	31	0.2510460251
REACTOME_SIGNALING_BY_PDGFR_IN_DISEASE	HRAS/KDR/NRAS/PDGFRA/PIK3R1/STAT3	32	0.250348675
WP_IL5_SIGNALING_PATHWAY	BCL2/JAK1/MAP2K1/MAPT/MYC/PIK3R1/RAF1/RPS6KB1/STAT3	33	0.2477917248
PID_CDC42_PATHWAY	APC/CDC42/CTNNB1/HRAS/MAPK14/MAPK9/MTOR/PIK3R1/RAF1/RPS6KB1	34	0.2472803347
BIOCARTA_ERK_PATHWAY	EGFR/HRAS/IGF1R/MAP2K1/MYC/PDGFRA/RAF1/STAT3	35	0.2468619247
BIOCARTA_MAPK_PATHWAY	CHUK/HRAS/MAP2K1/MAPK14/MAPK9/MYC/RAF1/RPS6KB1/TGFBR1	36	0.2468619247

PID_PDGFRB_PATHWAY	FYN/HRAS/MAP2K1/MAPK9/MYC/NCKAP1/NRAS/PDGFRB/PIK3R1/PTEN/RAF1/STAT3	37	0.2451185495
WP_ERBB_SIGNALING_PATHWAY	AKT2/AKT3/BCL2L11/CCND1/CDKN1A/EGFR/HRAS/MAP2K1/MAPK9/MTOR/MYC/NRAS/PIK3R1/RAF1/RPS6KB1	38	0.2412831241
REACTOME_SIGNALING_BY_NTRK3_TRKC	BAX/HRAS/NRAS/NTRK3/PIK3R1	39	0.240167364
WP_IL2_SIGNALING_PATHWAY	BCL2/CCND2/FYN/HRAS/JAK1/MAP2K1/MAPT/MYC/PIK3R1/RAF1/RPS6KB1/STAT3	40	0.2388423989
BIOCARTA_NFAT_PATHWAY	FGF2/GATA4/HRAS/IGF1/MAP2K1/MAPK14/PIK3R1/RAF1/RPS6KB1	41	0.2357043236
SIG_IL4RECEPTOR_IN_B_LYPHOCYTES	AKT2/AKT3/BCL2/IRS2/JAK1/PIK3R1/RAF1	42	0.2355050807
WP_THYMIC_STROMAL_LYMPHOPOIETIN_TSLP_SIGNALING_PATHWAY	FYN/JAK1/MAP2K1/MAPK14/MAPK9/MTOR/MYC/STAT3	43	0.234832636
PID_VEGFR1_2_PATHWAY	CDC42/CTNNB1/FYN/KDR/MAP2K1/MAPK14/PIK3R1/RAF1/VEGFA	44	0.2343096234
KEGG_VEGF_SIGNALING_PATHWAY	AKT2/AKT3/CDC42/HRAS/KDR/MAP2K1/MAPK14/NRAS/PIK3R1/PTGS2/RAF1/VEGFA	45	0.2336122734
WP_TCELL_RECEPTOR_SIGNALING_PATHWAY	CDC42/CHUK/FYN/HRAS/ITPR1/MAP2K1/MAPK14/MAPK9/PIK3R1/RAF1/ZAP70	46	0.2335488779
WP_HEPATOCYTE_GROWTH_FACTOR_RECEPTOR_SIGNALING	HGF/HRAS/MAP2K1/MET/PTEN/RAF1/STAT3	47	0.2319187089

KEGG_T_CELL_RECEPTOR_SIGNALING_PATHWAY	AKT2/AKT3/CDC42/CDK4/CHUK/FYN/HRAS/MAP2K1/MAPK14/MAPK9/NRAS/PIK3R1/RAF1/ZAP70	48	0.2307232516
KEGG_NEUROTROPHIN_SIGNALING_PATHWAY	AKT2/AKT3/BAX/BCL2/CDC42/HRAS/IRS2/MAP2K1/MAPK14/MAPK9/NRAS/NTRK3/PIK3R1/RAF1	49	0.2295277944
REACTOME_CD28_DEPENDENT_PI3K_AKT_SIGNALING	AKT2/AKT3/FYN/MTOR/PIK3R1/RICTOR	50	0.2266387727
PID_ERBB1_DOWNSTREAM_PATHWAY	CDC42/EGFR/HRAS/MAP2K1/MAPK9/MTOR/NCKAP1/NRAS/PIK3R1/RAF1/RICTOR/SMAD1/STAT3	51	0.2265851304
WP_INTERLEUKIN11_SIGNALING_PATHWAY	BCL2/BIRC5/CHUK/FYN/HRAS/JAK1/MAP2K1/PIK3R1/RAF1/RPS6KB1/RUNX2/STAT3	52	0.2255927476
WP_KIT_RECEPTOR_SIGNALING_PATHWAY	BCL2/FYN/HRAS/MAP2K1/MAPK14/MAPT/PIK3R1/RAF1/RPS6KB1/SNAI1/SNAI2/STAT3	53	0.2238493724
WP_SEROTONIN_RECEPTOR_2_AND_ELKSRFGATA4_SIGNALING	GATA4/HRAS/ITPR1/MAP2K1/NRAS/RAF1	54	0.2238493724
REACTOME_CD28_CO_STIMULATION	AKT2/AKT3/CDC42/FYN/MTOR/PIK3R1/RICTOR	55	0.218768679
WP_TRANSLATION_INHIBITORS_IN_CHRONICALLY_ACTIVATED_PDGFRA_CELLS	AKT2/AKT3/EIF4E/MAP2K1/MAPK14/MAPK9/MTOR/PDK1/PIK3R1/RPS6KB1	56	0.2184100418
REACTOME_SIGNALING_BY_NTRKS	BAX/FOSL1/FYN/HRAS/IRS2/MAP2K1/MAPK14/NRAS/NTRK3/PIK3R1/STAT3	57	0.2145302396
WP_IL6_SIGNALING_PATHWAY	IL6R/JAK1/MAP2K1/PIK3R1/PRDM1/RPS6KB1/STAT3	58	0.2115959354

WP_LEPTIN_SIGNALING_PATHWAY	BAX/CCND1/CDC42/CHUK/EIF4E/ESR1/FYN/HRAS/JAK1/MAP2K1/MAPK14/MTOR/PIK3R1/PTEN/RAF1/RPS6KB1/STAT3	59	0.2111740094
BIOCARTA_EGFR_SMRTE_PATHWAY	EGFR/MAP2K1/MAPK14/RXRA	60	0.210251046
PID_MET_PATHWAY	AKT2/APC/CDC42/CTNNB1/EIF4E/HGF/HRAS/MAP2K1/MET/MTOR/PIK3R1/RAF1/SNAI1	61	0.2095268748
PID_A6B1_A6B4_INTEGRIN_PATHWAY	CASP7/EGFR/HRAS/MET/PIK3R1/RPS6KB1/RXRA	62	0.2080095637
PID_IL6_7_PATHWAY	IL6R/JAK1/MAPK14/MYC/PIK3R1/STAT3/TNFSF11	63	0.2080095637
PID_CERAMIDE_PATHWAY	BAX/BCL2/CASP8/FADD/MAP2K1/MYC/RAF1	64	0.207411835
REACTOME_SIGNALING_BY_TYPE_1_INSULIN_LIKE_GROWTH_FACTOR_1_RECEPTOR_IGF1R	AKT2/FGF2/HRAS/IGF1/IGF1R/IRS2/NRAS/PIK3R1	65	0.2071129707
SIG_INSULIN_RECEPTOR_PATHWAY_IN_CARDIAC_MYOCYTES	AKT2/AKT3/CDC42/IRS2/PIK3R1/PTEN/RAF1/RPS6KB1/SLC2A4	66	0.2064156206
WP_THYROID_HORMONES_PRODUCTION_AND_PERIPHERAL_DOWNSTREAM_SIGNALING_EFFECTS	AKT3/CTNNB1/HRAS/MAP2K1/MAPK14/MTOR/NOTCH1/PPARG/RAF1/RPS6KB1/RXRA	67	0.2054012933
WP_EGFEGFR_SIGNALING_PATHWAY	CDC42/E2F1/EGFR/HRAS/ITCH/JAK1/MAP2K1/MAPK14/MAPK9/MTOR/PIK3R1/PTEN/RAF1/RICTOR/RPS6KB1/STAT3/TWIST1	68	0.2050209205

KEGG_INSULIN_SIGNALING_PATHWAY	AKT2/AKT3/EIF4E/FASN/HRAS/INSR/IRS2/MAP2K1/MAPK9/MTOR/NRAS/PIK3R1/RAF1/RPS6KB1/SLC2A4/SREBF1	69	0.2018828452
WP_MAPK_SIGNALING_PATHWAY	AKT2/AKT3/CDC42/CHUK/EGFR/FGF2/HRAS/MAP2K1/MAPK14/MAPK9/MAPT/MYC/NRAS/PDGFRB/RAF1/TAB1/TGFBR1	70	0.1993600788
PID_SHP2_PATHWAY	EGFR/HRAS/IGF1R/IL6R/JAK1/KDR/MAP2K1/NRAS/NTRK3/PDGFRB/PIK3R1/RAF1/VEGFA	71	0.1989056968
BIOCARTA_CTCF_PATHWAY	MTOR/MYC/PIK3R1/PTEN/RPS6KB1/SMAD1/TGFBR1	72	0.1978481769
WP_PHYSICOCHEMICAL_FEATURES_AND_TOXICITYASSOCIATED_PATHWAYS	APC/CDKN1A/CTNNB1/EGFR/FZD6/MAP2K1/MYC/RAF1	73	0.1976987448
WP_RANKLRANK_SIGNALING_PATHWAY	AKT2/CDC42/CHUK/MAP2K1/MAPK14/MAPK9/MTOR/PIK3R1/TAB1/TNFSF11/TRAF5	74	0.1966527197
WP_INSULIN_SIGNALING	AKT2/EIF4E/HRAS/IGF1R/INSR/IRS2/MAP2K1/MAPK14/MAPK9/MTOR/PFKL/PIK3R1/PTEN/RAF1/RPS6KB1/SLC2A4	75	0.1966527197
BIOCARTA_CERAMIDE_PATHWAY	BAX/BCL2/CASP8/FADD/MAP2K1/RAF1	76	0.1966527197
PID_IL2_PI3K_PATHWAY	BCL2/E2F1/JAK1/MTOR/MYB/MYC/PIK3R1/RPS6KB1	77	0.1966527197
REACTOME_MET_ACTIVATES_RAS_SIGNALING	HGF/HRAS/MET/NRAS	78	0.1966527197

WP_IL4_SIGNALING_PATHWAY	BIRC5/CHUK/IRS2/JAK1/MAPK14/PIK3R1/RPS6KB1/STAT3	79	0.1961297071
BIOCARTA_GSK3_PATHWAY	APC/CCND1/CTNNB1/LEF1/PIK3R1	80	0.1941422594
KEGG_MAPK_SIGNALING_PATHWAY	AKT2/AKT3/CDC42/CHUK/EGFR/FGF2/HRAS/MAP2K1/MAPK14/MAPK9/MAPT/MYC/NRAS/PDGFRA/PDGFRB/RAF1/TAB1/TGFBR1	81	0.1933984193
KEGG_GAP_JUNCTION	EGFR/HRAS/ITPR1/MAP2K1/NRAS/PDGFRA/PDGFRB/RAF1/TJP1	82	0.1933984193
PID_EPHB_FWD_PATHWAY	CDC42/EFNB2/EPHB1/EPHB2/EPHB4/HRAS/MAP2K1/NRAS/PIK3R1	83	0.1920037192
WP_RAC1PAK1P38MMP2_PATHWAY	BAX/BIRC5/CASP7/CHUK/CTNNB1/EGFR/HRAS/MAPK14/MAPK9/MMP2/MYC/NRAS/PIK3R1/STAT3/YAP1	84	0.1910739191
KEGG_NATURAL_KILLER_CELL_MEDIATED_CYTOTOXICITY	FYN/HRAS/IFNB1/MAP2K1/NRAS/PIK3R1/RAF1/TNFRSF10A/TNFRSF10B/ULBP2/ZAP70	85	0.1905667554
PID_ANGIOPOIETIN_RECEPTOR_PATHWAY	CDKN1A/FGF2/FYN/MAPK14/MMP2/PIK3R1/RPS6KB1	86	0.1900777047
PID_PTP1B_PATHWAY	CSF1/EGFR/FYN/INSR/PDGFRB/PIK3R1/STAT3	87	0.1900777047
WP_INTERFERON_TYPE_I_SIGNALING_PATHWAYS	EIF4E/FYN/IRS2/JAK1/MAPK14/MTOR/PIK3R1/RPS6KB1/STAT3/ZAP70	88	0.1878661088

WP_PI3KAKTMTOR_SIGNALING_PATHWAY_AND_THERAPEUTIC_OPPORTUNITIES	HRAS/MTOR/NRAS/PDK1/PIK3R1/PTEN/RB1CC1/RICTOR	89	0.1877615063
WP_RELATIONSHIP_BETWEEN_INFLAMMATION_COX2_AND_EGFR	AKT2/AKT3/EGFR/ESR1/HRAS/NRAS/PTGS2	90	0.1876867902
WP_FGFR3_SIGNALING_IN_CHONDROCYTE_PROLIFERATION_AND_TERMINAL_DIFFERENTIATION	CDKN1A/MAP2K1/MAPK14/RAF1/RBL1/SNAI1	91	0.1875871688
WP_RAS_SIGNALING	AKT2/AKT3/CDC42/CHUK/EGFR/EPHA2/HRAS/IGF1R/INSR/KDR/MAP2K1/MAPK9/MET/NRAS/PDGFRA/PDGFRB/PIK3R1/RAF1/ZAP70	92	0.1874036556
BIOCARTA_EIF4_PATHWAY	EIF4E/MAPK14/MTOR/PABPC1/PIK3R1/PTEN/RPS6KB1	93	0.1829049611
WP_EXTRACELLULAR_VESICLEMEDIATED_SIGNALING_IN_RECIPIENT_CELLS	APC/CTNNB1/EGFR/HGF/HRAS/MET/MTOR/NRAS/RAF1/SMAD2/TGFBR1	94	0.1821985546
WP_BRAINDERIVED_NEUROTROPHIC_FACTOR_BDNF_SIGNALING_PATHWAY	APC/BCL2L11/CDC42/CHUK/CTNNB1/EIF4E/FYN/HRAS/IGF2BP1/IRS2/MAP2K1/MAPK14/MAPK9/MAPT/MARCKS/MTOR/NTRK3/PIK3R1/RAF1/RPS6KB1/STAT3	95	0.1807132895
WP_REGULATION_OF_ACTIN_CYTOSKELETON	APC/CDC42/EGFR/FGF2/GIT1/MAP2K1/NCKAP1/NRAS/PDGFRA/PDGFRB/PIK3R1/RAF1	96	0.180264993
REACTOME_SIGNALING_BY_ALK	HIF1A/MYC/MYCN/PIK3R1/PRDM1/STAT3	97	0.179916318
BIOCARTA_ARF_PATHWAY	E2F1/MYC/PIK3R1/RB1/TWIST1	98	0.1790794979

WP_INTERLEUKIN1_IL1_STRUCTURAL_PATHWAY	CHUK/EIF4E/MAP2K1/MAPK14/MAPK9/MYC/TAB1/TAB3	99	0.1788702929
WP_ANGIOPOIETINLIKE_PROTEIN_8_REGULATORY_PATHWAY	AKT2/EIF4E/FASN/HRAS/INSR/IRS2/MAP2K1/MAPK14/MAPK9/MTOR/PIK3R1/RAF1/RICTOR/RPS6KB1/RXRA/SLC2A4/SREBF1/SREBF2	100	0.1771269177
BIOCARTA_IGF1MTOR_PATHWAY	EIF4E/IGF1/IGF1R/MTOR/PIK3R1/PTEN/RPS6KB1	101	0.173341303
KEGG_TOLL_LIKE_RECEPTOR_SIGNALING_PATHWAY	AKT2/AKT3/CASP8/CCL4/CHUK/FADD/IFNB1/MAP2K1/MAPK14/MAPK9/PIK3R1/TAB1/TLR4	102	0.1725136788
REACTOME_SIGNALING_BY_PTK6	CCND1/CCNE1/CDK4/EGFR/HIF1A/HRAS/NRAS/STAT3	103	0.1720711297
PID_PI3KCI_AKT_PATHWAY	AKT2/AKT3/CDKN1A/CHUK/MTOR/RAF1/RICTOR/SLC2A4	104	0.1720711297
WP_AGERAGE_PATHWAY	CASP8/CDC42/CHUK/EGFR/HIF1A/INSR/LGALS3/MAP2K1/MAPK14/MAPK9/MMP2/RAF1/SMAD2/STAT3	105	0.1703526599
KEGG_ADIPOCYTOKINE_SIGNALING_PATHWAY	AKT2/AKT3/CHUK/IRS2/MAPK9/MTOR/RXRA/SLC2A4/STAT3	106	0.1687587169
WP_EGFR_TYROSINE_KINASE_INHIBITOR_RESISTANCE	AKT2/AKT3/AXL/BAX/BCL2/BCL2L11/CCND1/EGFR/EIF4E/FGF2/HGF/HRAS/IGF1/IGF1R/IL6R/JAK1/KDR/MAP2K1/MET/MTOR/MYC/NRAS/PDGFRA/PDGFRB/PIK3R1/PTEN/RAF1/RPS6KB1/STAT3/VEGFA	107	0.1686192469
PID_AJDISS_2PATHWAY	CDC42/CTNNB1/EGFR/FYN/HRAS/IGF1R/MET/RET	108	0.1663179916

REACTOME_VEGFR2_MEDIATED_CELL_PROLIFERATION	HRAS/ITPR1/KDR/NRAS/VEGFA	109	0.1648535565
WP_TNFRELATED_WEAK_INDUCER_OF_APOPTOSIS_TWEAK_SIGNALING_PATHWAY	AKT2/CASP7/CASP8/CHUK/CTNNB1/FADD/MAPK14/MAPK9/RAF1/TRAF5	110	0.1640167364
PID_IL4_2PATHWAY	COL1A2/HMGA1/IRS2/JAK1/MAPK14/MTOR/MYB/PIK3R1/RPS6KB1	111	0.1627150163
WP_TOLLLIKE_RECEPTOR_SIGNALING_PATHWAY	AKT2/AKT3/CASP8/CCL4/CHUK/FADD/IFNB1/MAP2K1/MAPK14/MAPK9/PIK3R1/TAB1/TAB3/TLR4	112	0.1625821877
WP_FOCAL_ADHESION	AKT2/AKT3/BCL2/CCND1/CCND2/CCND3/CDC42/COL1A2/CTNNB1/EGFR/FYN/HGF/HRAS/IGF1/IGF1R/KDR/MAP2K1/MAPK9/MET/PDGFRA/PDGFRB/PIK3R1/PTEN/RAF1/RELN/VEGFA/VEGFC	113	0.1624050829
WP_REGULATORY_CIRCUITS_OF_THE_STAT3_SIGNALING_PATHWAY	EGFR/IL6R/JAK1/MAPK14/MAPK9/MTOR/PDGFRA/PDGFRB/RICTOR/STAT3	114	0.1589958159
REACTOME_SIGNALING_BY_VEGF	AKT2/AKT3/AXL/CDC42/CTNNB1/CYBB/FYN/HRAS/ITPR1/KDR/MAPK14/MTOR/NCKAP1/NRAS/PIK3R1/RICTOR/VEGFA/VEGFC	115	0.1578335658

KEGG_FOCAL_ADHESION	AKT2/AKT3/BCL2/CCND1/CCND2/CCND3/CDC42/COL1A2/COL3A1/CTNNB1/EGFR/FYN/HGF/HRAS/IGF1/IGF1R/KDR/MAP2K1/MAPK9/MET/PDGFRA/PDGFRB/PIK3R1/PTEN/RAF1/RELN/VEGFA/VEGFC	116	0.15720263
PID_IL2_STAT5_PATHWAY	BCL2/CCNA2/CCND2/CCND3/CDK6/JAK1/MYC/PIK3R1	117	0.1558577406
WP_TNFALPHA_SIGNALING_PATHWAY	BAX/BTRC/CASP7/CASP8/CHUK/FADD/HRAS/MAPK9/NRAS/RAF1/TAB1/TAB3	118	0.1551603905
BIOCARTA_TEL_PATHWAY	BCL2/EGFR/IGF1R/MYC/RB1	119	0.1548117155
PID_TRAIL_PATHWAY	CASP8/CHUK/FADD/PIK3R1/TNFRSF10A/TNFRSF10B	120	0.1541143654
KEGG_APOPTOSIS	AKT2/AKT3/BAX/BCL2/CASP7/CASP8/CHUK/FADD/PIK3R1/TNFRSF10A/TNFRSF10B	121	0.15405097
PID_MTOR_4PATHWAY	CCNE1/EIF4E/HRAS/MAP2K1/MTOR/NRAS/RAF1/RB1CC1/RICTOR/RPS6KB1/SREBF1/ULK2/YY1	122	0.1525587383
REACTOME_NEGATIVE_REGULATION_OF_THE_PI3K_AKT_NETWORK	AKT2/AKT3/EGFR/ESR1/FGF2/FYN/HGF/INSR/IRS2/MET/PDGFRA/PDGFRB/PIK3R1/PTEN	123	0.1509264794

REACTOME_DISEASES_OF_SIGNAL_TRANSDUCTION_BY_GROWTH_FACTOR_RECEPTORS_AND_SECOND_MESSENGERS	AKT2/AKT3/APC/BCL2L11/CDK8/CDKN1A/CHUK/CTNNB1/EGFR/ESR1/FBXW7/FGF2/FYN/FZD6/HGF/HRAS/IRS2/JAG1/KAT2B/KDR/MAP2K1/MET/MTOR/MYC/NOTCH1/NRAS/PDGFRA/PDGFRB/PIK3R1/PTEN/RAF1/RICTOR/SMAD2/STAT3/TGFBR1	124	0.1502689779
REACTOME_MAPK_FAMILY_SIGNALING_CASCADES	AGO1/ARL2/CCND3/CDC42/EGFR/FGF2/FYN/HGF/HRAS/IGF2BP1/IL6R/IRS2/JAK1/MAP2K1/MET/MMP2/MYC/NRAS/PDGFRA/PDGFRB/PIK3R1/RAF1/RET/SPTAN1	125	0.1501046025
WP_DNA_DAMAGE_RESPONSE_ONLY_ATM_DEPENDENT	AKT2/AKT3/APC/BAX/BCL2/BCL2L11/CCND1/CCND2/CCND3/CDC42/CDKN1A/CTNNB1/FOSL1/HRAS/INSR/LEF1/MAPK9/MYC/NRAS/PDK1/PIK3R1/PTEN/RBL2/WNT7A	126	0.14958159
REACTOME_C_TYPE_LECTIN_RECEPTORS_CLRS	BTRC/CASP8/CDC34/CHUK/FYN/HRAS/ITPR1/NRAS/RAF1/TAB1/TAB3	127	0.1468238874
WP_REGUCALCIN_IN_PROXIMAL_TUBULE_EPITHELIAL_KIDNEY_CELLS	BAX/CASP8/MTOR/RAF1/SMAD2/TGFBR1/TNFSF11	128	0.1440526001

WP_VEGFAVEGFR2_SIGNALING	BCL2/BIRC5/CCND1/CDC42/CTNNB1/CYBB/EIF4E/EPHA2/EPHB2/FADD/FYN/HRAS/ITCH/JAG1/KDR/MAP2K1/MAPK14/MAPK9/MMP2/MTOR/NOTCH4/PABPC1/PIK3R1/PTGS2/RAF1/RHOC/RICTOR/RPS6KB1/STAT3/VEGFA	129	0.1423988842
BIOCARTA_WNT_PATHWAY	APC/BTRC/CCND1/CTNNB1/MYC/TAB1	130	0.140167364
PID_TCPTP_PATHWAY	CSF1/EGFR/HGF/INSR/JAK1/KDR/KPNA2/MET/PDGFRB/PIK3R1/STAT3/VEGFA	131	0.1391213389
WP_WNT_SIGNALING_PATHWAY	APC/AXIN2/CCND1/CDK6/CTNNB1/LEF1/MAPK9/MTOR/MYC/PPARG	132	0.1355648536
WP_NONALCOHOLIC_FATTY_LIVER_DISEASE	AKT2/AKT3/BAX/BCL2L11/CASP7/CASP8/CDC42/IL6R/INSR/IRS2/ITCH/MAPK9/PIK3R1/RXRA/SMAD7/SREBF1	133	0.1351987448
REACTOME_TOLL_LIKE_RECEPTOR_TLR1_TLR2_CASCADE	APP/BTRC/CASP8/CHUK/MAP2K1/MAPK14/MAPK9/TAB1/TAB3/TLR4	134	0.1330543933
REACTOME_TOLL_LIKE_RECEPTOR_9_TLR9_CASCADE	APP/BTRC/CASP8/CHUK/MAP2K1/MAPK14/MAPK9/TAB1/TAB3/TLR4	134	0.1330543933
REACTOME_INTRINSIC_PATHWAY_FOR_APOPTOSIS	AKT2/AKT3/BAX/BCL2/BCL2L11/BMF/CASP7/CASP8/E2F1/STAT3	135	0.1326359833

KEGG_MTOR_SIGNALING_PATHWAY	AKT2/AKT3/CAB39/EIF4E/HIF1A/IGF1/MTOR/PIK3R1/RICTOR/RPS6KB1/ULK2/VEGFA/VEGFC	136	0.1326037979
PID_EPHRINB_REV_PATHWAY	EFNB2/EPHB1/EPHB2/EPHB4/FYN/PIK3R1	137	0.1317991632
BIOCARTA_TGFB_PATHWAY	APC/MAP2K1/SMAD2/SMAD7/TAB1/TGFBR1	138	0.1317991632
WP_NOTCH_SIGNALING_PATHWAY	CCND1/CDKN1A/FBXW7/HIF1A/ITCH/JAG1/MAPT/MYC/NOTCH1/NOTCH4/PIK3R1/STAT3	139	0.1311018131
WP_ALZHEIMERS_DISEASE	AKT2/AKT3/APC/APP/ATG14/AXIN2/CASP7/CASP8/CHUK/CSF1/CTNNB1/CYBB/FADD/FZD6/HRAS/INSR/IRS2/ITPR1/MAP2K1/MAPK9/MAPT/MTOR/NRAS/PIK3R1/PTGS2/RAF1/RB1CC1/SNCA/ULK2/WNT7A	140	0.129567643
WP_ALZHEIMERS_DISEASE_AND_MIRNA_EFFECTS	AKT2/AKT3/APC/APP/ATG14/AXIN2/CASP7/CASP8/CHUK/CSF1/CTNNB1/CYBB/FADD/FZD6/HRAS/INSR/IRS2/ITPR1/MAP2K1/MAPK9/MAPT/MTOR/NRAS/PIK3R1/PTGS2/RAF1/RB1CC1/SNCA/ULK2/WNT7A	140	0.129567643
REACTOME_ACTIVATION_OF_BH3_ONLY_PROTEINS	AKT2/AKT3/BCL2/BCL2L11/BMF/E2F1	141	0.1290097629
PID_AVB3_INTEGRIN_PATHWAY	CASP8/COL1A2/COL3A1/CSF1/FGF2/IGF1R/KDR/PIK3R1/RPS6KB1/VEGFA	142	0.1288702929

REACTOME_MYD88_INDEPENDENT_TLR4_CASCADE	APP/BTRC/CASP8/CHUK/FADD/MAP2K1/MAPK14/MAPK9/TAB1/TAB3/TLR4	143	0.1285659947
WP_TGFBETA_SIGNALING_PATHWAY	APP/BTRC/CCND1/CDC42/CDKN1A/COL1A2/DAB2/E2F5/ITCH/MAP2K1/MAPK14/MAPK9/MET/MYC/PDK1/PIK3R1/RAF1/RBL1/RBL2/RUNX2/SMAD2/SMAD7/TAB1/TGFBR1/YAP1	144	0.1280334728
WP_TH17_CELL_DIFFERENTIATION_PATHWAY	HIF1A/IL6R/JAK1/MAPK14/MTOR/RXRA/SMAD2/STAT3/TGFBR1/ZAP70	145	0.1276150628
WP_APOPTOSIS	BAX/BCL2/BCL2L11/BCL2L2/BIRC5/CASP7/CASP8/CHUK/FADD/IGF1/IGF1R/MYC/PIK3R1/TNFRSF10B	146	0.1276150628
WP_FOCAL_ADHESION_PI3KAKTMTORSIGNALING_PATHWAY	AKT2/AKT3/CAB39/CDKN1A/COL1A2/COL3A1/CSF1/EGFR/EIF4E/EPHA2/FGF2/HGF/HIF1A/HRAS/IFNB1/IGF1/IGF1R/IL6R/INSR/IRS2/JAK1/KDR/MAP2K1/MET/MTOR/NRAS/PDGFRA/PDGFRB/PIK3R1/PTEN/RAF1/RELN/RPS6KB1/SLC2A3/SLC2A4/SREBF1/VEGFA/VEGFC	147	0.1266240916
PID_PS1_PATHWAY	APC/CCND1/CTNNB1/MYC/NKD1/NOTCH1/TAB1	148	0.1261207412
WP_TROP2_REGULATORY_SIGNALING	BAX/BCL2/CCND1/CCNE1/CDK4/CHEK1/CTNNB1/EGFR/IGF1/IGF1R/NOTCH1/PIK3R1/PTEN/RB1	149	0.1261207412

REACTOME_EPHB_MEDIATED_FORWARD_SIGNALING	CDC42/EFNB2/EPHB1/EPHB2/EPHB4/FYN/HRAS	150	0.1261207412
REACTOME_SIGNALING_BY_INTERLEUKINS	APP/BCL2/BIRC5/BTRC/CASP8/CCL4/CCND1/CDC42/CDKN1A/CHUK/COL1A2/CSF1/FGF2/FYN/HGF/HIF1A/IL6R/IRS2/JAK1/MAP2K1/MAPK14/MAPK9/MMP2/MYC/NANOG/PIK3R1/PTGS2/SOX2/STAT3/TAB1/TAB3/TWIST1/VEGFA	151	0.1257765944
WP_AMPACTIVATED_PROTEIN_KINASE_SIGNALING	AKT2/CAB39/CCNA2/CDKN1A/FASN/HNF4A/INSR/MTOR/PIK3R1/RPS6KB1/SLC2A4/SREBF1	152	0.1251743375
WP_PI3KAKT_SIGNALING_PATHWAY	AKT2/AKT3/BCL2/BCL2L11/BRCA1/CCND1/CCND2/CCND3/CCNE1/CCNE2/CDK4/CDK6/CDKN1A/CHUK/COL1A2/CSF1/EGFR/EIF4E/EPHA2/FGF2/HGF/HRAS/IFNB1/IGF1/IGF1R/IL6R/INSR/JAK1/KDR/MAP2K1/MET/MTOR/MYB/MYC/NRAS/PDGFRA/PDGFRB/PIK3R1/PTEN/RAF1/RBL2/RELN/RPS6KB1/TLR4/VEGFA/VEGFC	153	0.1242495907
WP_TGFBETA_RECEPTOR_SIGNALING	BAMBI/CTNNB1/HRAS/JAK1/LEF1/MAPK9/RUNX2/RUNX3/SMAD1/SMAD2/SMAD7/STAT3/TGFBR1	154	0.1239137432
PID_MYC_REPRESS_PATHWAY	BCL2/BRCA1/CCND1/CDKN1A/COL1A2/MYC/PDGFRB/RBL1/SMAD2	155	0.1236634124

PID_CMYB_PATHWAY	ADORA2B/BCL2/CBX4/CCND1/CDK6/CDKN1A/COL1A2/HRAS/LEF1/MYB/MYC/NRAS/PTGS2/TAB1	156	0.1228332337
REACTOME_INTERLEUKIN_4_AND_INTERLEUKIN_13_SIGNALING	BCL2/BIRC5/CCND1/CDKN1A/COL1A2/FGF2/HGF/HIF1A/IL6R/JAK1/MMP2/MYC/NANOG/PIK3R1/PTGS2/SOX2/STAT3/TWIST1/VEGFA	157	0.1224399912
WP_OVERVIEW_OF_NANOPARTICLE_EFFECTS	AKT3/BAX/BCL2/CCND3/PTGS2	158	0.1188284519
WP_HIPPO_SIGNALING_REGULATION_PATHWAYS	CDC42/CTNNB1/EGFR/EPHA2/IGF1R/INSR/KDR/LEF1/MET/MTOR/PDGFRA/PDGFRB/SMAD2	159	0.1184422272
REACTOME_TRANSCRIPTIONAL_REGULATION_OF_GRANULOPOIESIS	CDK4/CDKN1A/E2F1/IL6R/LEF1/MYB/MYC/RXRA/STAT3	160	0.1129707113
WP_NANOMATERIAL_INDUCED_APOPTOSIS	BAX/BCL2/CASP7/CASP8/FADD	161	0.1121338912
REACTOME_NUCLEOTIDE_BINDING_DOMAIN_LEUCINE_RICH_REPEAT_CONTAINING_RECEPTOR_NLR_SIGNALING_PATHWAYS	APP/BCL2/CASP8/CHUK/ITCH/MAPK14/TAB1/TAB3	162	0.1114016736
WP_NEURAL_CREST_CELL_MIGRATION_DURING_DEVELOPMENT	AKT2/AKT3/EPHB1/EPHB2/EPHB4/MMP2/STAT3/TWIST1	163	0.109832636
PID_AP1_PATHWAY	BCL2L11/CCND1/COL1A2/CTNNB1/ESR1/FOSL1/HIF1A/MYB/MYC/PTEN	164	0.1092050209

REACTOME_SIGNALING_BY_NUCLEAR_RECEPTORS	ABCA1/ABCG1/AGO1/AKT2/AKT3/ARL4C/BCL2/CCND1/EGFR/ESR1/FASN/HRAS/IGF1R/KAT2B/KPNA2/MMP2/MYB/MYC/MYLIP/NRAS/PDK1/PIK3R1/RARG/RDH10/RXRA/SREBF1/YY1	165	0.1080117775
PID_FOXO_PATHWAY	BCL2L11/CHUK/CTNNB1/KAT2B/MAPK9/RBL2/SIRT1	166	0.1075911536
BIOCARTA_DEATH_PATHWAY	BCL2/CASP7/CASP8/CHUK/FADD/SPTAN1	167	0.1073919107
WP_FACTORS_AND_PATHWAYS_AFFECTING_INSULINLIKE_GROWTH_FACTOR_IGF1AKT_SIGNALING	EIF4E/IGF1/IGF1R/MTOR/PDK1/PTEN/RICTOR/RPS6KB1/SMAD2	168	0.1059972106
PID_GLYPICAN_1PATHWAY	APP/FGF2/FYN/SMAD2/TGFBR1/VEGFA	169	0.1046025105
REACTOME_INTRACELLULAR_SIGNALING_BY_SECOND_MESSENGERS	AGO1/AKT2/AKT3/BMI1/CBX4/CDKN1A/CHUK/EGFR/ESR1/EZH2/FGF2/FYN/HGF/INSR/IRS2/ITPR1/KPNA2/MET/MTOR/NR2E1/PDGFRA/PDGFRB/PIK3R1/PPARG/PTEN/RICTOR/SNAI1/SNAI2/SUZ12	170	0.1038811138
WP_ANGIOGENESIS	FGF2/HIF1A/KDR/MAPK14/PDGFRA/SMAD1/VEGFA	171	0.1034070532
WP_WNT_SIGNALING	APC/CCND1/CCND2/CCND3/CTNNB1/FOSL1/FZD6/LEF1/MAPK9/MYC/NKD1/WNT7A	172	0.1032078103

WP_IL18_SIGNALING_PATHWAY	BAX/BCL2/CASP8/CCL4/CCNA2/CHUK/COL1A2/COL3A1/CTNNB1/FADD/FBXW7/GRM7/MAPK9/MMP2/PIK3R1/PTEN/PTGS2/RPS6KB1/RUNX2/TNFSF11/ULBP2/VEGFA	173	0.1027006466
KEGG_TGF_BETA_SIGNALING_PATHWAY	E2F5/MYC/RBL1/RBL2/RPS6KB1/SMAD1/SMAD2/SMAD7/TGFBR1	174	0.1008833101
REACTOME_APOPTOSIS	AKT2/AKT3/APC/BAX/BCL2/BCL2L11/BMF/CASP7/CASP8/CTNNB1/E2F1/FADD/MAPT/SPTAN1/STAT3/TJP1/TLR4/TNFRSF10A/TNFRSF10B	175	0.1008588417
REACTOME_EPH_EPHRIN_SIGNALING	CDC42/EFNB2/EPHA2/EPHB1/EPHB2/EPHB4/FYN/GIT1/HRAS/MMP2	176	0.09958158996
WP_MIRNAS_INVOLVED_IN_DNA_DAMAGE_RESPONSE	CCND1/CCND3/CCNE1/CDC25A/CDK6/CDKN1A/E2F1/MYC	177	0.09937238494
KEGG_ADHERENS_JUNCTION	CDC42/CTNNB1/EGFR/FYN/IGF1R/INSR/LEF1/MET/NECTIN4/SMAD2/SNAI1/SNAI2/TGFBR1/TJP1	178	0.09862522415
REACTOME_PROGRAMMED_CELL_DEATH	AKT2/AKT3/APC/BAX/BCL2/BCL2L11/BMF/CASP7/CASP8/CTNNB1/E2F1/FADD/ITCH/MAPT/SPTAN1/STAT3/TJP1/TLR4/TNFRSF10A/TNFRSF10B	179	0.09790794979
PID_WNT_NONCANONICAL_PATHWAY	CDC42/CTHRC1/FZD6/MAPK9/PPARG/TAB1	180	0.09623430962

KEGG_WNT_SIGNALING_PATHWAY	APC/AXIN2/BTRC/CCND1/CCND2/CCND3/CTNNB1/FOSL1/FZD6/LEF1/MAPK9/MYC/NKD1/SMAD2/WNT7A	181	0.09511854951
REACTOME_FOXO_MEDIATED_TRANSCRIPTION	AKT2/AKT3/BCL2L11/CDKN1A/KAT2B/KLF4/RBL2/SIRT1/SMAD2/SREBF1	182	0.09288702929
PID_ECADHERIN_STABILIZATION_PATHWAY	CTNNB1/EGFR/EPHA2/GIT1/HGF/IGF1R/MET/NCKAP1	183	0.09152719665
PID_BETA_CATENIN_NUC_PATHWAY	APC/AXIN2/CCND1/CCND2/CTNNB1/IGF2BP1/KLF4/LEF1/MMP2/MYC/SNAI2	184	0.09128946367
WP_NEUROINFLAMMATION_AND_GLUTAMATERGIC_SIGNALING	BCL2/FGF2/GRM7/IGF1/IL6R/INSR/JAK1/SLC2A3/SMAD2/SMAD7/STAT3/TGFBR1/TRAF5	185	0.09076279369
WP_CCL18_SIGNALING_PATHWAY	COL1A2/HIF1A/MMP2/MYC/PTEN/SNAI1/TWIST1/VEGFC	186	0.09048117155
WP_NUCLEAR_RECEPTORS_METAPATHWAY	BAX/CCND1/CDK4/CYP2C9/EGFR/EPHA2/ESR1/FASN/HGF/IRS2/KAT2B/MYC/PTGS2/RXRA/SLC2A3/SLC2A4/SNAI2/SREBF1/STAT3	187	0.08940761947
WP_SPINAL_CORD_INJURY	CCND1/CDC42/CDK4/E2F1/E2F5/EFNB2/EGFR/LGALS3/MYC/PTGS2/RB1/RHOC/TLR4	188	0.08883167042
WP_WNT_SIGNALING_PATHWAY_AND_PLURIPOTENCY	APC/AXIN2/CCND1/CCND2/CCND3/CTNNB1/FOSL1/FZD6/LEF1/MAPK9/MYC/NANOG/NKD1/SOX2/WNT7A	189	0.08814504881

PID_CASPASE_PATHWAY	APP/BAX/BCL2/CASP7/CASP8/SPTAN1/SREBF1	190	0.08786610879
WNT_SIGNALING	APC/BTRC/CCND1/CCND2/CCND3/CTNNB1/FOSL1/FZD6/LEF1/MYC/NKD1/WNT7A	191	0.08751743375
REACTOME_TRANSCRIPTION_OF_E2F_TARGETS_UNDER_NEGATIVE_CONTROL_BY_DREAM_COMPLEX	CDC25A/E2F1/E2F5/MYC/RBL1/RBL2	192	0.08647140865
REACTOME_SMAD2_SMAD3_SMAD4_HETEROTRIMER_REGULATES_TRANSCRIPTION	CDK8/COL1A2/E2F5/MYC/RBL1/SMAD2/SMAD7/TGIF2	193	0.08472803347
WP_H19_ACTION_RBE2F1_SIGNALING_AND_CDKBETACATENIN_ACTIVITY	CCND1/CDK4/CDK8/CTNNB1/E2F1/JAG1/RB1	194	0.084279737
PID_RXR_VDR_PATHWAY	ABCA1/BCL2/PPARG/RARG/RPS6KB1/RXRA/SREBF1	195	0.084279737
PID_NOTCH_PATHWAY	CCND1/CDKN1A/FBXW7/ITCH/JAG1/MYC/NOTCH1/NOTCH4/YY1	196	0.08414690841
REACTOME_APOPTOTIC_CLEAVAGE_OF_CELLULAR_PROTEINS	APC/CASP7/CASP8/CTNNB1/MAPT/SPTAN1/TJP1	197	0.08308427974
REACTOME_APOPTOTIC_EXECUTION_PHASE	APC/CASP7/CASP8/CTNNB1/MAPT/SPTAN1/TJP1	197	0.08308427974
REACTOME_PTEN_REGULATION	AGO1/AKT2/AKT3/BMI1/CBX4/EZH2/MTOR/NR2E1/PPARG/PTEN/SNAI1/SNAI2/SUZ12	198	0.08046346958
WP_NEUROGENESIS_REGULATION_IN_THE_OLFACTORY_EPITHELIUM	APP/JAG1/MAPT/MYC/NOTCH1/NTRK3/RELN/RET/SOX2/STAT3	199	0.07991631799
PID_TGFBR_PATHWAY	BAMBI/CTNNB1/DAB2/ITCH/RPS6KB1/SMAD2/SMAD7/TAB1/TGFBR1/YAP1	200	0.07949790795

REACTOME_TRANSCRIPTIONAL_ACTIVITY_OF_SMAD2_SMAD3_SMAD4_HETEROTRIMER	CDK8/COL1A2/E2F5/MYC/RBL1/SMAD2/SMAD7/TGIF2/USP9X	201	0.07717340772
WP_ADIPOGENESIS	CDKN1A/CTNNB1/E2F1/GATA4/HIF1A/HMGA1/IGF1/IRS2/NAMPT/PPARG/RB1/RBL1/RBL2/RXRA/SLC2A4/SREBF1/STAT3/TWIST1	202	0.0760111576
WP_NEOVASCULARISATION_PROCESSES	EPHB2/EPHB4/HIF1A/JAG1/KDR/MAPK9/NOTCH1/NOTCH4/SMAD1/SMAD2/TGFBR1	203	0.0756941803
WP_DYRK1A	APP/CCND1/CDK4/CDK6/E2F5/FGF2/MAPT/MTOR/NOTCH1/RBL1/RBL2/SIRT1	204	0.07531380753
WP_DNA_DAMAGE_RESPONSE	BAX/BRCA1/CASP8/CCND1/CCND2/CCND3/CCNE1/CCNE2/CDC25A/CDK4/CDK6/CDKN1A/CHEK1/E2F1/MYC/RB1/TNFRSF10B	205	0.07457543687
WP_MIRNA_REGULATION_OF_DNA_DAMAGE_RESPONSE	BAX/BRCA1/CASP8/CCND1/CCND2/CCND3/CCNE1/CCNE2/CDC25A/CDK4/CDK6/CDKN1A/CHEK1/E2F1/MYC/RB1/TNFRSF10B	205	0.07457543687
REACTOME_REGULATION_BY_C_FLIP	CASP8/FADD/TNFRSF10A/TNFRSF10B	206	0.07426778243
WP_NEURAL_CREST_DIFFERENTIATION	AXIN2/CTNNB1/FGF2/ISL1/MYB/MYC/NOTCH1/NOTCH4/SMAD1/SNAI1/SNAI2/TWIST1	207	0.07391910739
REACTOME_REGULATION_OF_RUNX1_EXPRESSION_AND_ACTIVITY	AGO1/CCND1/CCND2/CCND3/CDK6	208	0.07364016736

WP_MARKERS_OF_KIDNEY_CELL_LINEAGE	AXIN2/CTNNB1/HNF4A/JAG1/KDR/NOTCH1/PDGFRB/SMAD1	209	0.07322175732
KEGG_CYTOKINE_CYTOKINE_RECEPTOR_INTERACTION	CCL4/CSF1/EGFR/HGF/IFNB1/IL6R/KDR/MET/PDGFRA/PDGFRB/TGFBR1/TNFRSF10A/TNFRSF10B/TNFSF11/VEGFA/VEGFC	210	0.07296025105
REACTOME_SIGNALING_BY_TGF_BETA_RECEPTOR_COMPLEX	BAMBI/CDK8/COL1A2/E2F5/MYC/RBL1/SMAD2/SMAD7/TGFBR1/TGIF2/USP9X	211	0.07265119817
WP_GALANIN_RECEPTOR_PATHWAY	BCL2L11/CDKN1A/PPARG/SLC2A4/VEGFA/YAP1	212	0.07252440725
WP_APOPTOSIS_MODULATION_AND_SIGNALING	BAX/BCL2/BCL2L11/BCL2L2/BIRC5/BMF/CASP7/CASP8/FADD/TNFRSF10A/TNFRSF10B	213	0.07112970711
WP_TRANSCRIPTION_FACTOR_REGULATION_IN_ADIPOGENESIS	INSR/IRS2/PPARG/RXRA/SLC2A4/TWIST1	214	0.07112970711
WP_VITAMIN_D_RECEPTOR_PATHWAY	CCND1/CCNE1/CDC34/CDKN1A/CYP2C9/EPHB4/HIF1A/KLF4/MYC/PRDM1/RXRA/SLC2A4/TNFSF11	215	0.07080785323
REACTOME_SIGNALING_BY_TGFB_FAMILY_MEMBERS	BAMBI/CDK8/COL1A2/E2F5/MYC/RBL1/SMAD1/SMAD2/SMAD7/TGFBR1/TGIF2/USP9X	216	0.07078103208
REACTOME_DEUBIQUITINATION	APC/AXIN2/BRCA1/CCNA2/CDC25A/ESR1/HIF1A/KAT2B/MYC/PTEN/SMAD1/SMAD2/SMAD7/TAB1/TGFBR1/USP9X/YY1	217	0.06916071868
PID_HES_HEY_PATHWAY	E2F1/GATA4/HIF1A/KDR/MYB/NOTCH1/RB1/RUNX2/STAT3/TWIST1/YY1	218	0.06884747052

WP_PLURIPOTENT_STEM_CELL_DIFFERENTIATION_PATHWAY	CSF1/FGF2/HGF/IGF1/IL6R/NOTCH1/TNFSF11/VEGFA	219	0.0679916318
REACTOME_TRANSCRIPTIONAL_REGULATION_OF_PLURIPOTENT_STEM_CELLS	EOMES/FGF2/KLF4/LIN28A/NANOG/SMAD2/SOX2/STAT3	220	0.0679916318
REACTOME_CASPASE_ACTIVATION_VIA_DEATH_RECEPTORS_IN_THE_PRESENCE_OF_LIGAND	CASP8/FADD/TLR4/TNFRSF10A/TNFRSF10B	221	0.06694560669
PID_SMAD2_3NUCLEAR_PATHWAY	CDK4/CDKN1A/COL1A2/E2F5/ESR1/HNF4A/IFNB1/KAT2B/MYC/RBL1/RUNX2/RUNX3/SMAD2/SMAD7/TGIF2	222	0.06610878661
WP_HEART_DEVELOPMENT	CTNNB1/GATA4/ISL1/NOTCH1/SMAD1/VEGFA/VEGFC	223	0.0651524208
WP_SREBF_AND_MIR33_IN_CHOLESTEROL_AND_LIPID_HOMEOSTASIS	ABCA1/FASN/MTOR/SIRT1/SREBF1/SREBF2	224	0.06276150628
REACTOME_OXIDATIVE_STRESS_INDUCED_SENESCENCE	AGO1/BMI1/CBX4/CDK4/CDK6/E2F1/E2F2/E2F3/EZH2/IFNB1/MAPK14/MAPK9/SUZ12	225	0.0624396524
REACTOME_EPH_EPHRIN_MEDIATED_REPULSION_OF_CELLS	EFNB2/EPHA2/EPHB1/EPHB2/EPHB4/FYN/MMP2	226	0.06037059175
WP_STEROL_REGULATORY_ELEMENTBINDING_PROTEINS_SREBP_SIGNALING	CDK8/FASN/MTOR/PPARG/SIRT1/SREBF1/SREBF2/YY1	227	0.06014644351
REACTOME_REGULATION_OF_PTEN_GENE_TRANSCRIPTION	BMI1/CBX4/EZH2/MTOR/NR2E1/PPARG/PTEN/SNAI1/SNAI2/SUZ12	228	0.0589958159
REACTOME_EPHRIN_SIGNALING	EFNB2/EPHB1/EPHB2/EPHB4/FYN/GIT1	229	0.05718270572

REACTOME_TRANSCRIPTIONAL_REGULATION_OF_WHITE_ADIPOCYTE_DIFFERENTIATION	CCND3/CDK4/CDK8/KLF4/PPARG/RXRA/SLC2A4/SREBF1/SREBF2	230	0.05113900511
REACTOME_TRANSCRIPTION_OF_E2F_TARGETS_UNDER_NEGATIVE_CONTROL_BY_P107_RBL1_AND_P130_RBL2_IN_COMPLEX_WITH_HDAC1	CCNA2/E2F1/E2F5/RBL1/RBL2	231	0.05020920502
WP_ID_SIGNALING_PATHWAY	CCNE1/RB1/RBL1/RBL2/SREBF1	232	0.04937238494
REACTOME_TRANSCRIPTIONAL_REGULATION_BY_RUNX1	AGO1/BMI1/CBX4/CCND1/CCND2/CCND3/CDK6/ESR1/ITCH/KAT2B/LGALS3/MYB/RUNX2/TJP1/YAP1	233	0.04574616457
WP_NAD_METABOLISM_SIRTUINS_AND_AGING	HIF1A/NAMPT/PPARG/SIRT1	234	0.04184100418
WP_MESODERMAL_COMMITMENT_PATHWAY	AXIN2/CCND1/EOMES/HMGA2/HNF4A/KLF4/LEF1/NANOG/RARG/SETD2/SMAD1/SMAD2/SNAI1/SOX2/YAP1	235	0.04128312413
WP_NUCLEAR_RECEPTORS	ESR1/HNF4A/NR2C2/NR2E1/PPARG/RARG/RXRA	236	0.03407053198
REACTOME_TRANSCRIPTIONAL_REGULATION_BY_E2F6	BMI1/BRCA1/CHEK1/E2F1/EZH2/SUZ12	237	0.03207810321
REACTOME_NR1H2_AND_NR1H3_MEDIATED_SIGNALING	ABCA1/ABCG1/AGO1/ARL4C/FASN/MYLIP/RXRA/SREBF1	238	0.03138075314
BIOCARTA_PRC2_PATHWAY	BMI1/CBX4/EZH2/SUZ12/YY1	239	0.02761506276

Supplementary material 17: FDA-approved drugs targeting genes regulated by dysregulated miRNAs in AD and T2DM.

		Gene	Drug	Interation score
TARGET GENES FOR DOWNREGULATED MICRORNAS		**AKT1_HUMAN**	CAPIVASERTIB	0.51
			NELFINAVIR	0.12
		**MAPK1**	BENZALKONIUM CHLORIDE	0.14
	TARGET GENES FOR UPREGULATED MICRORNAS	**MAP2K1**	COBIMETINIB	0.56
			TRAMETINIB DIMETHYL SULFOXIDE	0.54
			SELUMETINIB	0.47
			BINIMETINIB	0.40
			DABRAFENIB	0.34
			VEMURAFENIB	0.33
			COBIMETINIB FUMARATE	0.31
			SELUMETINIB SULFATE	0.31
			ENCORAFENIB	0.27
			PANITUMUMAB	0.18
			CETUXIMAB	0.16
		**RAF1_HUMAN**	TOVORAFENIB	0.66
			ENCORAFENIB	0.22
			REGORAFENIB	0.18
			SORAFENIB	0.15
		**PIK3R1**	LENIOLISIB	0.33
			COPANLISIB	0.18
			ALPELISIB	0.11
		**HRAS**	SOTORASIB	0.41
			PRALSETINIB	0.17
			LORLATINIB	0.14
			TRAMETINIB DIMETHYL SULFOXIDE	0.14
			SELUMETINIB	0.11
			VITAMIN E	0.11
			CABOZANTINIB S-MALATE	0.11
